# Synthesis of *N*-Substituted Iminosugar *C*-Glycosides and Evaluation as Promising *α*-Glucosidase Inhibitors

**DOI:** 10.3390/molecules27175517

**Published:** 2022-08-27

**Authors:** Haibo Wang, Senling Tang, Guoqing Zhang, Yang Pan, Wei Jiao, Huawu Shao

**Affiliations:** 1 Natural Products Research Centre, Chengdu Institute of Biology, Chinese Academy of Sciences, Chengdu 610041, China; 2 University of Chinese Academy of Sciences, Beijing 100049, China; 3 Zhejiang Hongyuan Pharmaceutical Co., Ltd., Linhai 317016, China; 4 School of Pharmacy, North Sichuan Medical College, Nanchong 637100, China

**Keywords:** iminosugars, *C*-glycosides, *α*-glucosidase, inhibitors

## Abstract

A series of *N*-substituted iminosugar *C*-glycosides were synthesized and tested for *α*-glucosidase inhibition. The results suggested that **6e** is a promising and potent *α*-glucosidase inhibitor. Enzymatic kinetic assays indicated that compound **6e** may be classified as an uncompetitive inhibitor. The study of structure-activity relationships of those iminosugars provided a starting point for the discovery of new *α*-glucosidase inhibitors.

## 1. Introduction

**Figure 1:**
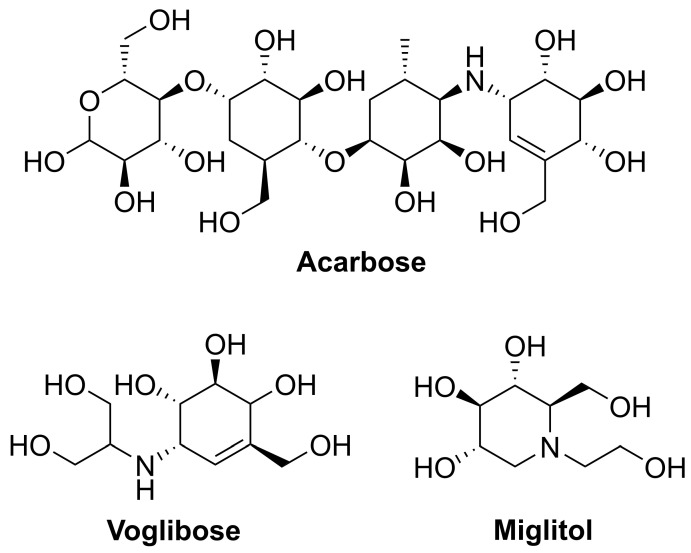
Important clinically used α-glucosidase inhibitors for the treatment of T2DM.

Diabetes mellitus (DM) is one of the most common chronic diseases with 1.5 million deaths each year [1]. According to the International Diabetes Federation (IDF), the prevalence of DM was estimated to be 783 million by 2045 [2]. Type 2 diabetes mellitus (T2DM) is the most common form of this disease and almost 80–90% of all diabetic patients are T2DM [3]. Generally, diabetic pathogeny mostly focuses on genetic, lifestyle, environmental toxins, and advancing age [4]. Current antidiabetic drugs to treat T2DM include incretin mimetic, biguanides, sulfonylureas, thiazolidinediones, dipeptidyl peptidase 4 inhibitors, sodium-glucose co-transporter 2 inhibitors, glucagon-like peptide 1 agonists, and α-glucosidase inhibitors [5,6]. α-Glucosidase (α-D-glucoside glucohydrolase; EC3.2.1.20) is the glycoside hydrolase specifically hydrolyzing 1,4-α-glucopyranosides bond to produce α-glucose, located in the brush border of the small intestine [7]. α-Glucosidase inhibitors are thought to be the most efficient agents to reduce postprandial hyperglycemia [8]. Thus, it has been considered as one of the most popular targets for the treatment of diabetes [9]. At present, α-glucosidase inhibitors have been marketed as therapeutic drugs for T2DM, including acarbose [10], voglibose [10], and miglitol [11], and have been used in reducing plasma glucose levels and postprandial hyperglycemia (Figure 1) [12]. However, their widespread application has been limited by the complicated multistep procedures needed for their synthesis and undesirable side effects such as gastrointestinal intolerability, diarrhoea and flatulence [13]. Therefore, the search for new small molecules possessing potent α-glucosidase inhibitory activity and minimal side effects has attracted significant attention for many years.

Iminosugars [14], formed by the replacement of sugar ring oxygen with nitrogen, are well known for their ability to selectively inhibit glycosidases [15]. This kind of scaffold inhibits glycosidases by mimicking the substrate transition states with oxacarbenium ion character during the hydrolysis reaction catalyzed by glycosidases [16]. In the past two decades, more than 100 iminosugars have been isolated from plants and microorganisms [17,18,19]. Besides, hundreds of their analogues and derivatives were synthesized and evaluation of their biological activity was assayed, especially as glucosidase inhibitors [20]. However, various comparative studies on simple glycolipid analogues have demonstrated a marked dependence of the potency of the inhibitors upon the position of the alkyl chain (1-*C*- or *N*-alkyl derivatives) [21]. Meanwhile, Butters, T. D. and co-workers also found that the presence of a hydrophobic *N*-alkyl chain of iminosugars provided an increase in inhibitory potency to glucosidases [22]. As a part of our continuing interest in the synthesis of novel iminosugars and their α-glucosidase inhibition, we report a library of *N*-substituted iminosugar *C*-glycosides and their structure-activity relationships against α-glucosidase.

## 2. Results and Discussion

### 2.1. Synthesis of Iminosugar C-Glycosides

**Scheme 1:**
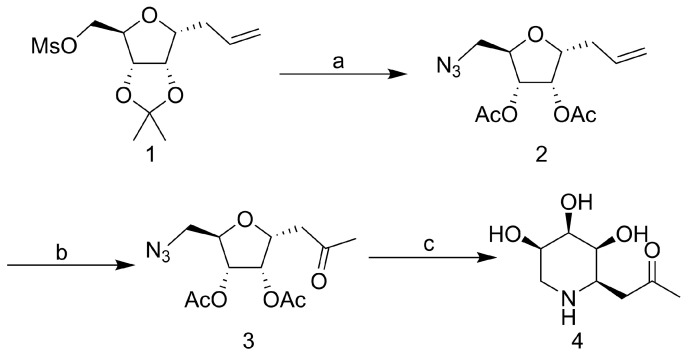
The synthesis of the compound 1-*C*-Acetylmethyl-5-deoxy-5-amino-*α*-D-ribopyranoside. Reagents and conditions: (**a**) NaN_3_, DMF, 80 °C, overnight; 6 N HCl, THF, 50 °C, 18 h; Ac_2_O, DMAP, 5 h. (**b**) Acetone/H_2_O:4/1, Hg(OAc)_2_, Jones reagent, 0 °C–r. t., overnight. (**c**) Pd/C, H_2_, MeOH, 5 h; 1% MeONa/MeOH, 8 h; Saturated MeONa/MeOH, overnight.

The synthesis of the compound 1-*C*-Acetylmethyl-5-deoxy-5-amino-*α*-D-ribopyranoside was based on our PREVIOUS synthetic strategy (Scheme 1) [23]. Nucleophilic substitution of known mesylate **1** with NaN_3_ generated 5-azido-*C*-riboside **2**. Reduction of the azido group to amine after terminal olefin oxidation, which was immediately treated with the saturated sodium methoxide-methanol solution at room temperature overnight. Purified the crude with silica gel flash column chromatography (ethyl acetate/methanol, 2:1, R_f_ = 0.2) to afford the compound **4**.

**Scheme 2:**
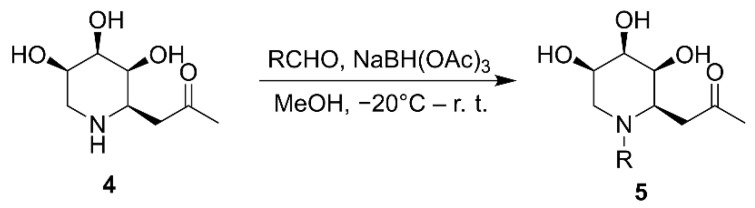
Synthesis of *N*-substituted iminosugar *C*-glycosides.

**Figure 2:**
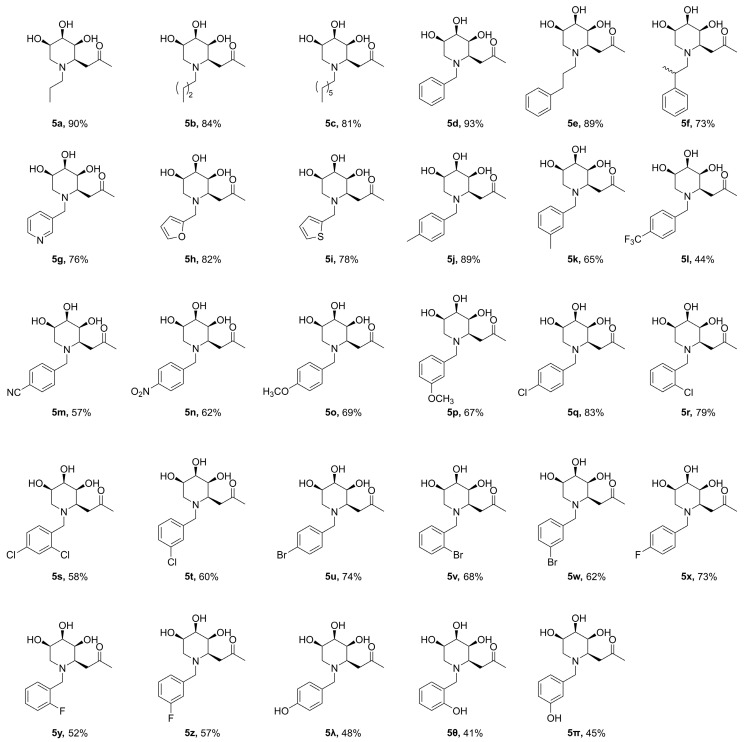
Synthesized *N*-substituted iminosugar *C*-glycosides (**5a**–**5π**).

Based on previous work, the inhibitory potency of *C*-glycosides **4** has been demonstrated for the *α*-glucosidase [23]. To obtain higher activity compounds, the compound **4** was structurally modified by introducing alkyl side chains on its nitrogen atom. The reaction conditions were screened (Table 1). Under the conditions of −20 °C–r. t., NaBH(OAc)_3_ and MeOH, good yield was obtained by reductive amination. Finally, a variety of aldehydes could be attached to the structure **4** through reductive amination and obtained *N*-substituted iminosugar *C*-glycosides **5** (Scheme 2, Figure 2).

### 2.2. α-Glucosidase Inhibitory Assays

**Figure 3:**
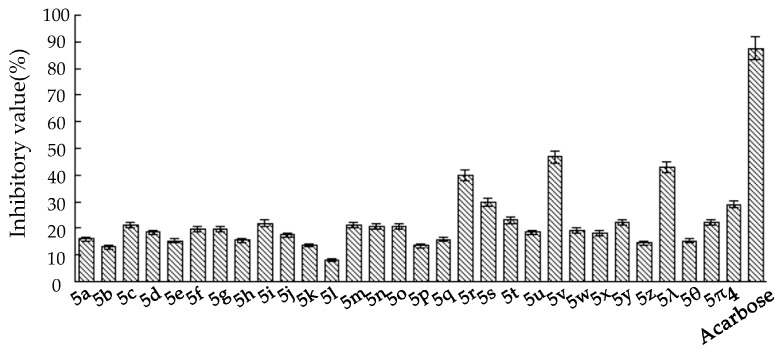
The inhibitory value of the compounds against *α*-glucosidase from yeast at 1 µg/mL.

The inhibitory potency of the synthesized *N*-substituted iminosugar *C*-glycosides **5** was assessed by testing the compounds in assays for the *α*-glucosidase from yeast [24]. Compared to the positive control Acarbose (Glucobay^®^), the lead compound (**4**) suggests weak inhibitory activity against *α*-glucosidase (Figure 3). To improve the inhibitory potency against *α*-glucosidase of the lead compound, construction of the *N*-functionalized iminosugars was carried out. Installation of the N atom with aliphatic chain (**5a**, **5b**, **5c**) showed no improvement of *α*-glucosidase inhibition, where shortened or lengthened *N*-alkylated chain was invalid. Alternatively, N atom was modified with various benzaldehydes, giving a library of iminosugar *C*-glycosides containing phenyl. It was fascinating to discover that the introduction of a hydroxyl group at C4-position (**5λ**) of the aromatic ring improved the enzyme inhibition, whereas introducing a hydroxyl at C2 or C3 (**5θ**, **5π**) reduced the inhibitory potency significantly. This indicates that a hydroxy in C4-position of the phenyl contributed to improving the enzyme inhibitory activity. These findings demonstrate that subtle changes in the iminosugar *N*-functionalized region may result in remarkable enzyme specificity. To identify more potent inhibitors, we also introduced halogen atoms onto the aromatic ring, and discovered that positioning Cl or Br at C2 (**5r**, **5v**) enhanced the enzyme inhibition. However, the location of the halogen atom at C3 or C4-position (**5q**, **5t**, **5u**, **5w**, **5x**, **5y**, **5z**) of the phenyl doesn’t show any significant improvement for the inhibitory activity on the enzyme. The same result was obtained when introducing electron-donating groups as CH_3_(**5j**, **5k**), OCH_3_(**5o**, **5p**) or electron-withdrawing groups such as CF_3_(**5l**), NO_2_(**5n**), CN(**5m**).

**Scheme 3:**
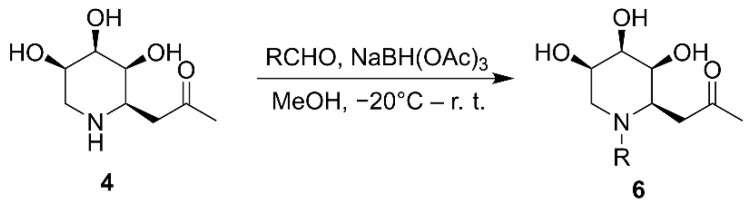
Synthesis of *N*-substituted iminosugar *C*-glycosides.

**Figure 4:**
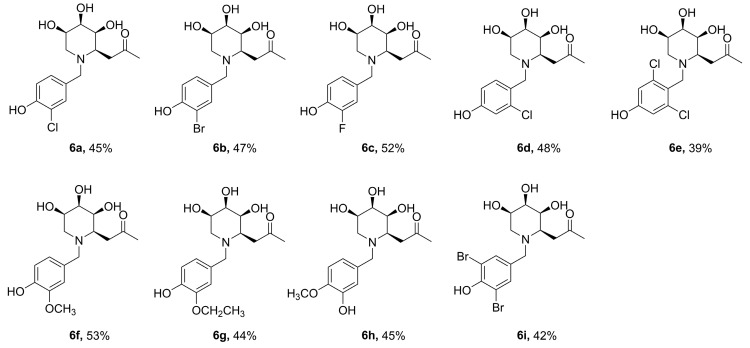
Synthesized *N*-substituted iminosugar *C*-glycosides (**6a**–**6i**).

Given the above results, and in order to ascertain whether hydroxyl and halogen atoms would have a synergistic effect on the enhancement of the enzyme inhibition, we synthesized a series of iminosugar *C*-glycosides **6** containing hydroxyl at C4-position on phenyl and introducing halogen atoms at different positions (Scheme 3, Figure 4). The assay of their inhibition against *α*-glucosidase was listed in Table 2. Compared with compound 5**λ**, the installation of halogen atoms at C3-position (**6a**, **6b**, **6c** and **6i**) can slightly improve the inhibition potency on the basis of the presence of hydroxyl at C4 position. However, introducing an alkoxyl at C3-position (**6f**, **6g**) severely diminishes the inhibition, which may be due to the fact that the substituents are too large to allow the guest to fit into the enzyme pocket. Clearly, iminosugars substituted with chlorine at the C2, 6-position (or at C2) provide greater enhancement of inhibition than others, such as **6e**, which has a 2-fold stronger inhibitory potency than the positive control Acarbose. The reason for this improvement in inhibition provided by additional chlorine atoms at C2, 6-position (or C2-position) and hydroxyl at C4-position on phenyl is not known but may be due to structural and spatial features of the enzyme, which allow the hydroxyl to bind with active site and chlorine atoms matching the pocket well.

**Figure 5:**
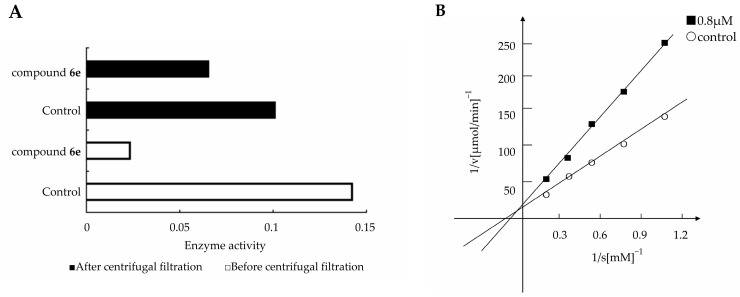
(**A**) Recovery of enzymatic activity by elimination of **6e** by centrifugal filtration. (**B**) Lineweaver–Burk plots of *α*-glucosidase without (○) and with (■) **6e**.

The inhibitory mechanisms of glucosidases were divided into two distinct mechanisms: reversible inhibitors that have a high affinity for the enzyme, and irreversible inhibitors that react with carboxylic acid of the active site of the enzyme [25]. To determine which mechanism **6e** belonged to, we removed the unreacted inhibitor from the enzyme solution by ultrafiltration [26], and examined whether the activity would be recovered or not by the method described in the literatures. When an enzyme solution containing **6e** was subjected to ultrafiltration and then redissolved with the same concentration, the *α*-glucosidase activity was substantially recovered (Figure 5A). From this result, we can conclude that **6e** is a reversible inhibitor against *α*-glucosidase.

Further analysis of the compound **6e**, in order to reveal the type of enzyme inhibition on *α*-glucosidase, was done with different substrate concentration [7]. Based on Lineweaver-Burk plots (Figure 5B), compound **6e** may be classified as an uncompetitive inhibitor with K*i* = 8.6 µM. These results indicate that **6e** may reversibly combine with only the E–S (enzyme–substrate) complex and inhibit the activity of this enzyme. It is specifically mentioned that uncompetitive inhibitors have a benefit over competitive inhibitors such as therapeutic drugs, since the inhibition is not overcome even when the substrate concentration reaches saturation.

### 2.3. Homology Modeling and Molecular Docking

Since the three-dimensional structure of *α*-glucosidase MAL12 in Saccharomyces cerevisiae is unknown, the FASTA format sequence of *α*-glucosidase is downloaded from the NCBI database (https://www.ncbi.nlm.nih.gov/ (accessed on 28 December 2021)). Furthermore, the three-dimensional structure of *α*-glucosidase was obtained by SWISS-MODEL [27,28,29,30,31] homology modeling method using 3AXH_A [32] (72% similarity) as template. The ligand was optimized using B3LYP [33,34] functional in gas phase, which was performed with in Gaussian 16 program [35]. Next, molecular docking was achieved employing AutoDock Vina [36]. PyMol and Ligplot [37] were applied to visualize the interactions between the ligand and the protein.

**Figure 6:**
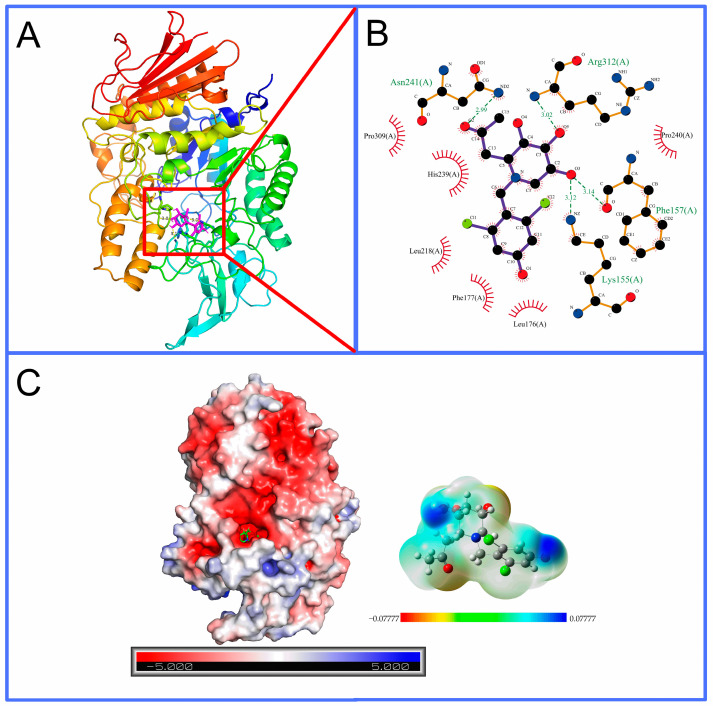
Binding interactions results of molecular docking between compound **6e** and *α*-glucosidase. (**A**) The ligand and protein are shown as magenta-colored stick models and colored cartoons, respectively. (**B**) Interactions between compound **6e** and its surrounding amino acid residues. (**C**) The electrostatic potential (ESP) map of α-glucosidase and compound **6e**. The scale of the ESP of α-glucosidase is −5.000 (negative, red) to 5.000 (positive, blue). The isovalue of 0.0004 electron/bohr^3^ was selected for the definition of the density surface of **6e** with a scale of −0.07777 (red) to 0.07777 hartree (blue).

As shown in Figure 6, compound **6e** forms hydrogen bonds with amino acid residues Lys155, Phe157, Asn241 and Arg312, as well as hydrophobic interactions with amino acid residues Leu176, Phe177, Leu218, His239, Pro240 and Pro309, which were close to the catalytic active site (Asp214) [38] of *α*-glucosidase, supporting the experimental conclusion that it is a noncompetitive inhibitor.

### 2.4. In Silico Analysis

Because some compounds showed good inhibitory activities, we chose to conduct in silico researches to evaluate their drug-likeness and pharmacokinetic properties that were carried out using the SwissADME [39] and the admetSAR [40] platforms. Satisfyingly, all compounds were found to have excellent obedience (75–100%) with different drug-likeness filters (Lipinski [41], Ghose [42], Veber [43], Egan [44], and Muegge [45]) (Table 3). In addition, the compounds **6d** and **6e** were found to have great average ADMET scores [46,47] (0.77, 0.79) in respect to human intestinal absorption, blood-brain barrier penetration, Caco-2 permeability, Ames mutagenicity, carcinogenicity, and acute oral toxicity class (Table 4).

## 3. Experimental

### 3.1. Chemistry

#### 3.1.1. General Procedures

All reactions sensitive to air or moisture were carried out under nitrogen or argon with anhydrous solvents. All reagents were purchased from commercial suppliers and used without further purification unless otherwise noted. TLC was performed by using silica gel GF254 precoated plates (0.20–0.25 mm thickness) with a fluorescent indicator. Visualization of TLC plates was achieved by UV light (254 nm) and a typical TLC indicator solution (10 % sulfuric acid/ethanol solution). Column chromatography was performed on silica gel 90, 200–300 mesh. Optical rotations were measured with a Perkin–Elmer M341 digital polarimeter. ^1^H and ^13^C NMR spectra (600 and 150 MHz, respectively) were recorded with a Bruker Avance 600 spectrometer. ^1^H NMR chemical shifts are reported in ppm (δ) relative to tetramethylsilane (TMS) with the solvent resonance employed as the internal standard (DMSO-*d*_6_ or CD_3_OD). Data are reported as follows: chemical shift, multiplicity (s = singlet, d = doublet, t = triplet, q = quartet, m = multiplet), integration, and coupling constants [Hz]. ^13^C NMR chemical shifts are reported in ppm from TMS with the solvent resonance as the internal standard (DMSO-*d*_6_ or CD_3_OD). ESI-HRMS data were recorded with a BioTOF Q instrument.

#### 3.1.2. Synthetic Procedures

A suspension of compound **4** (40 mg, 0.2 mmol) containing activated 4Å molecular sieves in anhydrous methanol (3 mL) was stirred at room temperature for 10 min. After cooling to −20 °C, the corresponding aldehydes (0.6 mmol, 3eq) and NaBH(OAc)_3_ (0.6 mmol, 3eq) were added, and the solution was stirred for 3 h under an argon atmosphere. The reaction solution was concentrated in vacuo and purified by silica gel flash column chromatography (dichloromethane/methanol, 25:1→5:1) to afford colorless syrupy products.

Detailed physicochemical properties of novel *N*-substituted iminosugar *C*-glycosides (see the supporting information for details):

**(1R, 2S, 3R, 4R)-1-Acetylmethyl-2, 3, 4-trihydroxyl-*N*-*n*-propylpiperidine (5a)**

colorless syrup, yield 90%, ${\left[\alpha \right]}_{20}^{D}$ −55.0 (*c* 0.08, CH_2_Cl_2_). ^1^H NMR (600 MHz, DMSO-*d*_6_) δ 3.67 (s, 1H), 3.51–3.43 (m, 2H), 3.09 (dd, *J* = 8.5, 2.4 Hz, 1H), 2.92 (d, *J* = 5.7 Hz, 1H), 2.62 (dd, *J* = 16.7, 4.7 Hz, 1H), 2.47–2.41 (m, 1H), 2.37–2.28 (m, 1H), 2.28–2.23 (m, 1H), 2.15–2.10 (m, 1H), 2.09 (s, 3H), 1.37–1.28 (m, 2H), 0.78–0.72 (m, 3H). ^13^C NMR (150 MHz, DMSO-*d*_6_) δ 208.2, 72.3, 70.7, 67.7, 56.9, 54.5, 52.0, 40.5, 30.6, 19.7, 12.1. ESI-HRMS: *m/z* calcd for C_11_H_22_NO_4_ [M+H]^+^: 232.1543; found: 232.1551.

**(1R, 2S, 3R, 4R)-1-Acetylmethyl-2, 3, 4-trihydroxyl-*N*-*n*-butylpiperidine (5b)**

colorless syrup, yield 84%, ${\left[\alpha \right]}_{20}^{D}$ −52.0 (*c* 0.01, CH_2_Cl_2_). ^1^H NMR (600 MHz, DMSO-*d*_6_) δ 3.66 (s, 1H), 3.45 (dd, *J* = 15.7, 9.9 Hz, 2H), 3.09 (d, *J* = 8.5 Hz, 1H), 2.91 (s, 1H), 2.63 (dd, *J* = 16.7, 4.6 Hz, 1H), 2.47–2.24 (m, 4H), 2.09 (s, 3H), 1.33–1.26 (m, 2H), 1.17 (ddd, *J* = 28.3, 14.0, 7.4 Hz, 2H), 0.83 (t, *J* = 7.3 Hz, 3H). ^13^C NMR (150 MHz, DMSO-*d*_6_) δ 208.2, 72.3, 70.7, 67.7, 57.0, 52.3, 52.0, 40.5, 30.6, 28.8, 20.4, 14.3. ESI-HRMS: *m/z* calcd for C_12_H_24_O_4_NNa [M+Na]^+^: 246.1700; found: 246.1712.

**(1R, 2S, 3R, 4R)-1-Acetylmethyl-2, 3, 4-trihydroxyl-*N*-*n*-heptylpiperidine (5c)**

colorless syrup, yield 81%, ${\left[\alpha \right]}_{20}^{D}$ −31.3 (*c* 0.16, CH_2_Cl_2_). ^1^H NMR (600 MHz, DMSO-*d*_6_) δ 3.66 (s, 1H), 3.44 (s, 1H), 3.08 (d, *J* = 7.1 Hz, 1H), 2.90 (s, 1H), 2.62 (dd, *J* = 16.7, 4.3 Hz, 1H), 2.46–2.40 (m, 1H), 2.38–2.28 (m, 2H), 2.08 (d, *J* = 10.7 Hz, 3H), 1.37–1.09 (m, 12H), 0.84 (t, *J* = 7.0 Hz, 3H). ^13^C NMR (150 MHz, DMSO-*d*_6_) δ 208.1, 72.3, 70.7, 67.7, 57.0, 52.6, 52.0, 40.6, 31.7, 30.6, 29.0, 27.2, 26.5, 22.5, 14.4. ESI-HRMS: *m/z* calcd for C_15_H_29_O_4_NNa [M+Na]^+^: 310.1989; found: 310.1998.

**(1R, 2S, 3R, 4R)-1-Acetylmethyl-2, 3, 4-trihydroxyl-*N*-benzylpiperidine (5d)**

colorless syrup, yield 93%, ${\left[\alpha \right]}_{20}^{D}$ −34.4 (*c* 0.34, CH_2_Cl_2_). ^1^H NMR (600 MHz, DMSO-*d*_6_) δ 7.42–7.15 (m, 5H), 3.69 (d, *J* = 15.0 Hz, 1H), 3.65 (d, *J* = 13.5 Hz, 1H), 3.41 (s, 1H), 3.19 (d, *J* = 8.5 Hz, 1H), 3.11 (d, *J* = 13.5 Hz, 1H), 3.00 (s, 1H), 2.80 (dd, *J* = 17.0, 3.7 Hz, 1H), 2.62 (dd, *J* = 17.0, 6.4 Hz, 1H), 2.30 (dd, *J* = 11.0, 3.9 Hz, 1H), 2.22 (t, *J* = 10.4 Hz, 1H), 2.08 (d, *J* = 10.2 Hz, 3H). ^13^C NMR (150 MHz, DMSO-*d*_6_) δ 208.3, 140.2, 128.9, 128.9, 128.6, 128.6, 127.2, 72.0, 72.0, 67.4, 57.3, 57.0, 52.1, 40.5, 30.6. ESI-HRMS: *m/z* calcd for C_15_H_22_NO_4_ [M+H]^+^: 280.1543; found: 280.1552.

**(1R, 2S, 3R, 4R)-1-Acetylmethyl-2, 3, 4-trihydroxyl-*N*-3-benzylpropylpiperidine (5e)**

colorless syrup, yield 89%, ${\left[\alpha \right]}_{20}^{D}$ −25.5 (*c* 0.40, CH_2_Cl_2_). ^1^H NMR (600 MHz, DMSO-*d*_6_) δ 7.25 (t, *J* = 7.5 Hz, 2H), 7.21–7.10 (m, 3H), 3.66 (s, 1H), 3.46 (s, 1H), 3.08 (d, *J* = 7.7 Hz, 1H), 2.91 (s, 1H), 2.59 (dd, *J* = 17.0, 4.3 Hz, 1H), 2.50-2.45 (m, 2H), 2.40–2.27 (m, 3H), 2.12 (dt, *J* = 15.7, 8.4 Hz, 1H), 1.97 (d, *J* = 17.3 Hz, 3H), 1.71–1.57 (m, 2H). ^13^C NMR (150 MHz, DMSO-*d*_6_) δ 208.1, 142.5, 128.8, 128.8, 128.7, 128.7, 126.1, 72.3, 70.6, 67.9, 56.8, 56.8, 51.9, 40.5, 33.0, 30.5, 28.2. ESI-HRMS: *m/z* calcd for C_17_H_26_NO_4_ [M+Na]^+^: 308.1856; found: 308.1863.

**(1R, 2S, 3R, 4R, 2’R/S)-1-Acetylmethyl-2, 3, 4-trihydroxyl-*N*-2’-benzyl-2’-methylethylpiperidine (5f)**

colorless syrup, yield 73%, ${\left[\alpha \right]}_{20}^{D}$ −53.3 (*c* 0.02, CH_2_Cl_2_). **(R)** ^1^H NMR (600 MHz, DMSO-*d*_6_) δ 7.25 (t, *J* = 7.5 Hz, 2H), 7.16 (dd, *J* = 7.5, 4.7 Hz, 2H), 3.65 (s, 1H), 3.52–3.44 (m, 1H), 3.14 (dd, *J* = 15.6, 8.4 Hz, 2H), 3.02 (s, 1H), 2.79 (dd, *J* = 13.9, 7.0 Hz, 1H), 2.65–2.58 (m, 2H), 2.56–2.39 (m, 2H), 2.35 (dd, *J* = 12.5, 9.9 Hz, 1H), 2.23 (dd, *J* = 12.9, 7.1 Hz, 1H), 2.07 (d, *J* = 2.1 Hz, 3H), 1.11 (d, *J* = 6.9 Hz, 2H). **(S)** ^1^H NMR (600 MHz, DMSO-*d*_6_) δ 7.25 (t, *J* = 7.5 Hz, 2H), 7.16 (dd, *J* = 7.5, 4.7 Hz, 2H), 3.59 (s, 1H), 3.41 (s, 1H), 3.20 (m, 2H), 2.91 (s, 1H), 2.79 (dd, *J* = 13.9, 7.0 Hz, 1H), 2.72–2.65 (m, 2H), 2.56–2.39 (m, 2H), 2.30 (t, *J* = 10.3 Hz, 1H), 2.15 (dd, *J* = 12.5, 5.0 Hz, 1H), 2.05 (s, 3H), 1.00 (d, *J* = 6.1 Hz, 2H). ESI-HRMS: *m/z* calcd for C_17_H_25_O_4_NNa [M+Na]^+^: 330.1676; found: 330.1685.

**(1R, 2S, 3R, 4R)-1-Acetylmethyl-2, 3, 4-trihydroxyl-*N*-3-pyridinemethylenepiperidine (5g)**

colorless syrup, yield 76%, ${\left[\alpha \right]}_{20}^{D}$ −10.8 (*c* 0.12, CH_2_Cl_2_). ^1^H NMR (600 MHz, DMSO-*d*_6_) δ 8.47–8.38 (m, 2H), 7.65 (d, *J* = 7.8 Hz, 1H), 7.31 (dd, *J* = 7.7, 4.8 Hz, 1H), 3.70 (s, 1H), 3.66 (d, *J* = 13.8 Hz, 1H), 3.42 (dd, *J* = 6.8, 4.3 Hz, 1H), 3.19 (dd, *J* = 8.4, 6.1 Hz, 2H), 3.03 (s, 1H), 2.80 (dd, *J* = 17.1, 4.3 Hz, 1H), 2.65 (dd, *J* = 17.1, 6.3 Hz, 1H), 2.25 (d, *J* = 6.9 Hz, 2H), 2.10 (s, 3H). ^13^C NMR (150 MHz, DMSO-*d*_6_) δ 208.3, 150.3, 148.5, 136.7, 135.5, 123.8, 71.9, 70.7, 67.3, 57.2, 54.1, 51.9, 40.5, 30.6. ESI-HRMS: *m/z* calcd for C_14_H_20_O_4_N_2_Na [M+Na]^+^: 303.1326; found: 303.1315.

**(1R, 2S, 3R, 4R)-1-Acetylmethyl-2, 3, 4-trihydroxyl-*N*-2-furanmethylenepiperidine (5h)**

colorless syrup, yield 82%, ${\left[\alpha \right]}_{20}^{D}$ −38.8 (*c* 0.26, CH_2_Cl_2_). ^1^H NMR (600 MHz, DMSO-*d*_6_) δ 7.54 (s, 1H), 6.36 (d, *J* = 1.9 Hz, 1H), 6.23 (d, *J* = 3.0 Hz, 1H), 3.67 (s, 1H), 3.57 (t, *J* = 12.3 Hz, 1H), 3.44–3.10 (m, 3H, overlap), 3.13 (dd, *J* = 9.1, 2.6 Hz, 1H), 2.89–2.80 (m, 1H), 2.66 (dd, *J* = 9.8, 5.3 Hz, 1H), 2.39 (dd, *J* = 11.0, 4.5 Hz, 1H), 2.32 (t, *J* = 10.5 Hz, 1H), 2.09 (s, 3H). ^13^C NMR (150 MHz, CDCl_3_) δ 208.2, 152.7, 142.6, 110.7, 109.0, 72.3, 71.0, 67.4, 56.6, 52.5, 49.5, 40.5, 30.7. ESI-HRMS: *m/z* calcd for C_13_H_19_O_5_NNa [M+Na]^+^: 292.1165; found: 292.1155.

**(1R, 2S, 3R, 4R)-1-Acetylmethyl-2, 3, 4-trihydroxyl-*N*-2-thiophenemethylenepiperidine (5i)**

colorless syrup, yield 78%, ${\left[\alpha \right]}_{20}^{D}$ −40.0 (*c* 0.16, CH_2_Cl_2_). ^1^H NMR (600 MHz, DMSO-*d*_6_) δ 7.37 (t, *J* = 10.4 Hz, 1H), 6.98–6.87 (m, 2H), 3.77 (d, *J* = 14.3 Hz, 1H), 3.70 (s, 1H), 3.50 (d, *J* = 14.3 Hz, 1H), 3.43 (s, 1H), 3.21–3.12 (m, 1H), 2.97 (d, *J* = 4.4 Hz, 1H), 2.76 (dd, *J* = 16.9, 4.4 Hz, 1H), 2.65–2.55 (m, 1H), 2.43 (dd, *J* = 11.0, 4.2 Hz, 1H), 2.29 (t, *J* = 10.4 Hz, 1H), 2.08 (d, *J* = 14.7 Hz, 3H). ^13^C NMR (150 MHz, DMSO-*d*_6_) δ 208.2, 143.8, 126.9, 126.0, 125.5, 72.0, 70.8, 67.3, 56.8, 52.2, 51.7, 44.6, 30.6. ESI-HRMS: *m/z* calcd for C_13_H_20_NO_4_S [M+H]^+^: 286.1108; found: 286.1106.

**(1R, 2S, 3R, 4R)-1-Acetylmethyl-2, 3, 4-trihydroxyl-*N*-4-methylbenzylpiperidine (5j)**

colorless syrup, yield 89%, ${\left[\alpha \right]}_{20}^{D}$ −31.8 (*c* 0.27, CH_2_Cl_2_). ^1^H NMR (600 MHz, DMSO-*d*_6_) δ 7.10 (dd, *J* = 24.3, 7.8 Hz, 4H), 3.69 (s, 1H), 3.59 (d, *J* = 13.3 Hz, 1H), 3.42–3.36 (m, 1H), 3.23–3.15 (m, 1H), 3.05 (d, *J* = 13.3 Hz, 1H), 2.96 (s, 1H), 2.78 (dd, *J* = 17.0, 3.9 Hz, 1H), 2.61 (dd, *J* = 17.0, 6.3 Hz, 1H), 2.28 (dd, *J* = 16.1, 6.9 Hz, 1H), 2.25 (s, 3H), 2.18 (t, *J* = 10.5 Hz, 1H), 2.08 (d, *J* = 10.5 Hz, 3H). ^13^C NMR (150 MHz, DMSO-*d*_6_) δ 208.3, 137.0, 136.2, 129.1, 129.1, 129.0, 129.0, 71.9, 70.8, 67.3, 57.3, 56.7, 51.9, 44.6, 30.6, 21.1. ESI-HRMS: *m/z* calcd for C_16_H_24_NO_4_ [M+H]^+^: 294.1700; found: 294.1714.

**(1R, 2S, 3R, 4R)-1-Acetylmethyl-2, 3, 4-trihydroxyl-*N*-3-methylbenzylpiperidine (5k)**

colorless syrup, yield 65%, ${\left[\alpha \right]}_{20}^{D}$ −56.6 (*c* 0.06, CH_2_Cl_2_). ^1^H NMR (600 MHz, DMSO-*d*_6_) δ 7.16 (t, *J* = 7.5 Hz, 1H), 7.03 (dd, *J* = 18.8, 9.4 Hz, 3H), 3.69 (d, *J* = 23.2 Hz, 1H), 3.61 (d, *J* = 13.3 Hz, 1H), 3.40 (s, 1H), 3.18 (t, *J* = 7.1 Hz, 1H), 3.05 (d, *J* = 13.4 Hz, 1H), 2.97 (s, 1H), 2.80 (dd, *J* = 17.2, 4.0 Hz, 1H), 2.61 (dd, *J* = 17.0, 6.4 Hz, 1H), 2.29 (dd, *J* = 12.1, 5.8 Hz, 2H), 2.26 (s, 3H), 2.09 (s, 3H). ^13^C NMR (150 MHz, DMSO-*d*_6_) δ 208.3, 140.1, 137.6, 129.6, 128.4, 127.8, 126.1, 72.1, 71.0, 67.4, 57.3, 57.1, 52.1, 44.8, 30.6, 21.5. ESI-HRMS: *m/z* calcd for C_16_H_24_NO_4_ [M+H]^+^: 294.1700; found: 294.1705.

**(1R, 2S, 3R, 4R)-1-Acetylmethyl-2, 3, 4-trihydroxyl-*N*-4-trifluoromethylbenzylpiperidine (5l)**

colorless syrup, yield 44%, ${\left[\alpha \right]}_{20}^{D}$ −40.0 (*c* 0.02, CH_2_Cl_2_). ^1^H NMR (600 MHz, DMSO-*d*_6_) δ 7.60 (s, 1H), 7.57 (d, *J* = 7.8 Hz, 2H), 7.54–7.50 (m, 1H), 3.72 (d, *J* = 14.2 Hz, 2H), 3.44 (s, 1H), 3.28 (d, *J* = 14.1 Hz, 1H, overlap), 3.22 (d, *J* = 6.4 Hz, 1H), 3.05 (t, *J* = 9.5 Hz, 1H), 2.80 (dd, *J* = 17.1, 4.2 Hz, 1H), 2.64 (dd, *J* = 17.1, 6.4 Hz, 1H), 2.30–2.24 (m, 2H), 2.08 (s, 3H). ^13^C NMR (150 MHz, DMSO-d6) δ 208.2, 142.0, 133.0, 133.0, 129.6, 129.6, 125.2, 123.9, 72.0, 70.7, 67.4, 57.2, 56.2, 52.2, 44.2, 40.5, 30.6. ESI-HRMS: *m/z* calcd for C_16_H_20_NF_3_O_4_Na [M+Na]^+^: 370.1237; found: 370.1246.

**(1R, 2S, 3R, 4R)-1-Acetylmethyl-2, 3, 4-trihydroxyl-*N*-4-cyanobenzylpiperidine (5m)**

colorless syrup, yield 57%, ${\left[\alpha \right]}_{20}^{D}$ 0 (*c* 0.09, CH_2_Cl_2_). ^1^H NMR (600 MHz, DMSO-*d*_6_) δ 7.58 (d, *J* = 8.1 Hz, 2H), 7.47 (d, *J* = 8.2 Hz, 2H), 3.70 (s, 1H), 3.64 (s, 1H), 3.44 (s, 1H), 3.21 (d, *J* = 8.5 Hz, 1H), 3.10 (d, *J* = 7.3 Hz, 1H), 3.04 (s, 1H), 2.79 (dd, *J* = 17.2, 4.5 Hz, 1H), 2.60 (dd, *J* = 17.0, 6.4 Hz, 1H), 2.26 (dt, *J* = 11.1, 9.0 Hz, 2H), 2.08 (s, 3H). ^13^C NMR (150 MHz, DMSO-*d*_6_) δ 208.3, 146.7, 145.9, 132.7, 132.7, 132.5, 132.5, 129.7, 73.4, 72.0, 71.3, 56.4, 52.3, 49.1, 40.5, 30.6. ESI-HRMS: *m/z* calcd for C_16_H_20_N_2_O_4_Na [M+Na]^+^: 327.1315; found: 327.1329.

**(1R, 2S, 3R, 4R)-1-Acetylmethyl-2, 3, 4-trihydroxyl-*N*-4-nitrobenzylpiperidine (5n)**

colorless syrup, yield 62%, ${\left[\alpha \right]}_{20}^{D}$ −30.0 (*c* 0.10, CH_2_Cl_2_). ^1^H NMR (600 MHz, DMSO-*d*_6_) δ 7.59 (d, *J* = 8.1 Hz, 2H), 7.48 (d, *J* = 8.2 Hz, 2H), 3.70 (s, 1H), 3.64 (s, 1H), 3.44 (s, 1H), 3.21 (d, *J* = 8.5 Hz, 1H), 3.10 (d, *J* = 7.3 Hz, 1H), 3.04 (s, 1H), 2.79 (dd, *J* = 17.2, 4.5 Hz, 1H), 2.60 (dd, *J* = 17.0, 6.4 Hz, 1H), 2.26 (dt, *J* = 11.1, 9.0 Hz, 2H), 2.08 (s, 3H). ^13^C NMR (150 MHz, DMSO-*d*_6_) δ 208.2, 148.1, 147.8, 129.8, 129.7, 123.9, 123.7, 73.4, 72.7, 58.6, 57.2, 56.2, 49.1, 40.5, 30.6. ESI-HRMS: *m/z* calcd for C_15_H_20_N_2_O_6_Na [M+Na]^+^: 347.1315; found: 347.1328.

**(1R, 2S, 3R, 4R)-1-Acetylmethyl-2, 3, 4-trihydroxyl-*N*-4-methoxylbenzylpiperidine (5o)**

colorless syrup, yield 69%, ${\left[\alpha \right]}_{20}^{D}$ +10.0 (*c* 0.12, CH_2_Cl_2_). ^1^H NMR (600 MHz, DMSO-*d*_6_) δ 7.15 (t, *J* = 8.6 Hz, 2H), 6.84 (d, *J* = 8.5 Hz, 2H), 3.73 (s, 1H), 3.70 (m, 1H, overlap), 3.57 (d, *J* = 13.3 Hz, 1H), 3.36 (d, *J* = 11.8 Hz, 1H), 3.17 (d, *J* = 8.5 Hz, 1H), 3.03 (d, *J* = 13.1 Hz, 1H), 2.96 (s, 1H), 2.78 (dd, *J* = 17.0, 4.0 Hz, 1H), 2.62 (dd, *J* = 17.0, 6.4 Hz, 1H), 2.36 (s, 1H), 2.28 (dd, *J* = 11.1, 4.1 Hz, 1H), 2.17 (t, *J* = 10.4 Hz, 1H), 2.08 (s, 3H). ^13^C NMR (150 MHz, DMSO-*d*_6_) δ 208.3, 158.6, 131.8, 131.8, 131.8, 114.0, 114.0, 79.6, 73.0, 71.9, 57.3, 56.3, 55.5, 51.8, 40.5, 30.6. ESI-HRMS: *m/z* calcd for C_16_H_24_NO_5_ [M+H]^+^: 310.1649; found: 310.1659.

**(1R, 2S, 3R, 4R)-1-Acetylmethyl-2, 3, 4-trihydroxyl-*N*-3-methoxylbenzylpiperidine (5p)**

colorless syrup, yield 67%, ${\left[\alpha \right]}_{20}^{D}$ −47.5 (*c* 0.08, CH_2_Cl_2_). ^1^H NMR (600 MHz, DMSO-*d*_6_) δ 7.20 (dd, *J* = 18.7, 11.0 Hz, 1H), 6.82 (d, *J* = 8.6 Hz, 2H), 6.77 (d, *J* = 7.6 Hz, 1H), 3.72 (s, 1H), 3.71 (s, 2H), 3.61 (d, *J* = 13.3 Hz, 1H), 3.43 (s, 1H), 3.20 (s, 1H), 3.15 (d, *J* = 5.0 Hz, 1H), 3.11 (d, *J* = 13.6 Hz, 1H), 3.00 (s, 1H), 2.79 (d, *J* = 13.6 Hz, 1H), 2.66–2.55 (m, 1H), 2.31 (d, *J* = 7.4 Hz, 1H), 2.23 (d, *J* = 10.2 Hz, 1H), 2.09 (s, 3H). ^13^C NMR (150 MHz, DMSO-*d*_6_) δ 208.3, 159.7, 141.9, 129.6, 121.1, 114.4, 112.5, 72.1, 70.8, 67.5, 57.3, 56.9, 55.4, 52.1, 49.1, 30.6. ESI-HRMS: *m/z* calcd for C_16_H_24_NO_5_ [M+H]^+^: 310.1649; found: 310.1658.

**(1R, 2S, 3R, 4R)-1-Acetylmethyl-2, 3, 4-trihydroxyl-*N*-4-chlorobenzylpiperidine (5q)**

colorless syrup, yield 83%, ${\left[\alpha \right]}_{20}^{D}$ −9.6 (*c* 0.26, CH_2_Cl_2_). ^1^H NMR (600 MHz, DMSO-*d*_6_) δ 7.33 (d, *J* = 8.3 Hz, 2H), 7.27 (d, *J* = 8.3 Hz, 2H), 3.70 (s, 1H), 3.62 (d, *J* = 13.8 Hz, 1H), 3.42 (d, *J* = 4.8 Hz, 1H), 3.20 (d, *J* = 8.1 Hz, 1H), 3.17–3.11 (m, 1H), 3.00 (t, *J* = 9.6 Hz, 1H), 2.79 (dd, *J* = 17.1, 4.3 Hz, 1H), 2.61 (dd, *J* = 17.1, 6.3 Hz, 1H), 2.27–2.19 (m, 2H), 2.09 (s, 3H). ^13^C NMR (150 MHz, DMSO-*d*_6_) δ 208.3, 139.6, 131.6, 130.7, 130.7, 128.5, 128.5, 79.6, 73.2, 72.0, 67.4, 56.1, 52.0, 40.5, 30.6. ESI-HRMS: *m/z* calcd for C_15_H_20_O_4_NClNa [M+Na]^+^: 336.0973; found: 336.0985.

**(1R, 2S, 3R, 4R)-1-Acetylmethyl-2, 3, 4-trihydroxyl-*N*-2-chlorobenzylpiperidine (5r)**

colorless syrup, yield 79%, ${\left[\alpha \right]}_{20}^{D}$ −31.2 (*c* 0.24, CH_2_Cl_2_). ^1^H NMR (600 MHz, DMSO-*d*_6_) δ 7.46 (t, *J* = 8.0 Hz, 1H), 7.35 (d, *J* = 7.9 Hz, 1H), 7.28 (t, *J* = 7.3 Hz, 1H), 7.22 (t, *J* = 7.6 Hz, 1H), 3.70 (s, 1H), 3.63 (d, *J* = 14.4 Hz, 1H), 3.42–3.36 (m, 1H), 3.20 (d, *J* = 11.1 Hz, 1H), 3.09 (d, *J* = 21.0 Hz, 1H), 2.80 (dd, *J* = 17.3, 4.0 Hz, 1H), 2.62 (dd, *J* = 17.3, 6.3 Hz, 1H), 2.35–2.29 (m, 2H), 2.06 (s, 3H). ^13^C NMR (150 MHz, DMSO-*d*_6_) δ 208.1, 137.3, 133.5, 131.1, 129.5, 128.8, 127.3, 72.1, 72.0, 67.5, 57.6, 54.1, 52.2, 40.5, 30.6. ESI-HRMS: *m/z* calcd for C_15_H_21_NClO_4_ [M+H]^+^: 314.1154; found: 314.1163.

**(1R, 2S, 3R, 4R)-1-Acetylmethyl-2, 3, 4-trihydroxyl-*N*-2,4-dichlorobenzylpiperidine (5s)**

colorless syrup, yield 58%, ${\left[\alpha \right]}_{20}^{D}$ +16.7 (*c* 0.09, CH_2_Cl_2_). ^1^H NMR (600 MHz, DMSO-*d*_6_) δ 7.52 (d, *J* = 2.1 Hz, 1H), 7.50 (d, *J* = 8.3 Hz, 1H), 7.39 (dd, *J* = 8.3, 2.0 Hz, 1H), 3.70 (s, 1H), 3.64–3.59 (m, 1H), 3.44 (s, 1H), 3.32 (d, *J* = 7.9 Hz, 1H), 3.19 (d, *J* = 11.0 Hz, 1H), 3.12–3.03 (m, 1H), 2.78 (dt, *J* = 17.2, 8.7 Hz, 1H), 2.61 (dd, *J* = 17.2, 6.2 Hz, 1H), 2.34 (dd, *J* = 20.5, 9.3 Hz, 1H), 2.28 (dd, *J* = 11.2, 4.0 Hz, 1H), 2.08 (d, *J* = 5.8 Hz, 3H). ^13^C NMR (150 MHz, DMSO-*d*_6_) δ 208.2, 136.7, 134.3, 134.1, 132.4, 128.9, 127.6, 73.6, 72.8, 72.1, 55.3, 53.5, 52.3, 40.5, 30.6. ESI-HRMS: *m/z* calcd for C_15_H_19_O_4_NCl_2_Na [M+Na]^+^: 370.0583; found: 370.0599.

**(1R, 2S, 3R, 4R)-1-Acetylmethyl-2, 3, 4-trihydroxyl-*N*-3-chlorobenzylpiperidine (5t)**

colorless syrup, yield 60%, ${\left[\alpha \right]}_{20}^{D}$ −31.3 (*c* 0.08, CH_2_Cl_2_). ^1^H NMR (600 MHz, DMSO-*d*_6_) δ 7.31 (d, *J* = 4.0 Hz, 2H), 7.26 (d, *J* = 8.2 Hz, 1H), 7.21 (d, *J* = 7.4 Hz, 1H), 3.71 (d, *J* = 3.0 Hz, 1H), 3.64 (d, *J* = 13.9 Hz, 1H), 3.49–3.41 (m, 1H), 3.23–3.17 (m, 2H), 3.01 (d, *J* = 13.1 Hz, 1H), 2.79 (dd, *J* = 17.1, 4.3 Hz, 1H), 2.62 (dd, *J* = 17.2, 6.4 Hz, 1H), 2.30–2.22 (m, 2H), 2.09 (s, 3H). ^13^C NMR (150 MHz, DMSO-*d*_6_) δ 208.2, 143.1, 133.4, 130.4, 128.5, 127.6, 127.1, 72.0, 70.8, 67.4, 57.2, 56.2, 52.1, 49.1, 30.6. ESI-HRMS: *m/z* calcd for C_15_H_21_ClNO_4_ [M+ H]^+^: 314.1154; found: 314.1155.

**(1R, 2S, 3R, 4R)-1-Acetylmethyl-2, 3, 4-trihydroxyl-*N*-4-bromobenzylpiperidine (5u)**

colorless syrup, yield 74%, ${\left[\alpha \right]}_{20}^{D}$ −19.5 (*c* 0.20, CH_2_Cl_2_). ^1^H NMR (600 MHz, DMSO-*d*_6_) δ 7.47 (d, *J* = 8.3 Hz, 2H), 7.21 (d, *J* = 8.2 Hz, 2H), 3.70 (s, 1H), 3.59 (t, *J* = 14.2 Hz, 1H), 3.39 (d, *J* = 23.1 Hz, 1H), 3.18 (t, *J* = 12.2 Hz, 1H), 3.12 (d, *J* = 14.4 Hz, 1H), 3.03–2.97 (m, 1H), 2.79 (dd, *J* = 17.1, 4.4 Hz, 1H), 2.61 (dd, *J* = 17.1, 6.3 Hz, 1H), 2.28–2.20 (m, 1H), 2.09 (s, 3H). ^13^C NMR (150 MHz, DMSO-*d*_6_) δ 208.3, 139.8, 131.4, 131.4, 131.1, 131.1, 120.1, 72.0, 72.0, 67.4, 57.3, 56.1, 52.1, 40.5, 30.6. ESI-HRMS: *m/z* calcd for C_15_H_21_O_4_NBrNa [M+Na]^+^: 358.0648; found: 358.0649.

**(1R, 2S, 3R, 4R)-1-Acetylmethyl-2, 3, 4-trihydroxyl-*N*-2-bromobenzylpiperidine (5v)**

colorless syrup, yield 68%, ${\left[\alpha \right]}_{20}^{D}$ −19.2 (*c* 0.12, CH_2_Cl_2_). ^1^H NMR (600 MHz, DMSO-*d*_6_) δ 7.55 (t, *J* = 7.8 Hz, 1H), 7.47 (d, *J* = 7.4 Hz, 1H), 7.34 (t, *J* = 7.4 Hz, 1H), 7.16 (t, *J* = 7.5 Hz, 1H), 3.70 (d, *J* = 12.1 Hz, 1H), 3.61 (d, *J* = 14.4 Hz, 1H), 3.44 (s, 1H), 3.31 (d, *J* = 8.1 Hz, 2H), 3.21 (s, 1H), 3.16 (s, 1H), 3.08 (s, 1H), 2.80 (dd, *J* = 17.2, 4.1 Hz, 1H), 2.64 (dd, *J* = 17.3, 6.3 Hz, 1H), 2.07 (d, *J* = 15.6 Hz, 3H). ^13^C NMR (150 MHz, DMSO-*d*_6_) δ 208.2, 138.9, 132.8, 131.3, 129.2, 128.0, 124.0, 72.2, 70.6, 67.7, 57.6, 56.6, 55.4, 52.3, 30.6. ESI-HRMS: *m/z* calcd for C_15_H_20_NBrO_5_Na [M+Na]^+^: 380.0468; found: 380.0459.

**(1R, 2S, 3R, 4R)-1-Acetylmethyl-2, 3, 4-trihydroxyl-*N*-3-bromobenzylpiperidine (5w)**

colorless syrup, yield 62%, ${\left[\alpha \right]}_{20}^{D}$ −16.0 (*c* 0.10, CH_2_Cl_2_). ^1^H NMR (600 MHz, DMSO-*d*_6_) δ 7.45 (s, 1H), 7.41–7.38 (m, 1H), 7.25 (d, *J* = 5.7 Hz, 2H), 3.70 (s, 1H), 3.64 (t, *J* = 11.4 Hz, 1H), 3.45–3.40 (m, 1H), 3.19 (dd, *J* = 14.9, 6.3 Hz, 2H), 3.02 (dd, *J* = 25.2, 7.1 Hz, 1H), 2.79 (dd, *J* = 17.1, 4.2 Hz, 1H), 2.62 (dd, *J* = 17.1, 6.4 Hz, 1H), 2.32–2.20 (m, 2H), 2.09 (s, 3H). ^13^C NMR (150 MHz, DMSO-*d*_6_) δ 208.2, 143.4, 131.4, 130.7, 130.0, 128.0, 122.1, 72.0, 70.8, 67.4, 57.2, 56.2, 52.2, 49.1, 30.6. ESI-HRMS: *m/z* calcd for C_15_H_21_BrNO_4_ [M+ H]^+^: 358.0648; found: 358.0659.

**(1R, 2S, 3R, 4R)-1-Acetylmethyl-2, 3, 4-trihydroxyl-*N*-4-fluorobenzylpiperidine (5x)**

colorless syrup, yield 73%, ${\left[\alpha \right]}_{20}^{D}$ −25.9 (*c* 0.22, CH_2_Cl_2_). ^1^H NMR (600 MHz, DMSO-*d*_6_) δ 7.27 (dd, *J* = 8.4, 5.8 Hz, 2H), 7.09 (t, *J* = 8.7 Hz, 2H), 3.68 (d, *J* = 20.6 Hz, 1H), 3.61 (d, *J* = 13.5 Hz, 1H), 3.43–3.39 (m, 1H), 3.19 (dd, *J* = 8.7, 2.3 Hz, 1H), 3.12 (d, *J* = 13.4 Hz, 1H), 3.00 (dd, *J* = 11.8, 7.4 Hz, 1H), 2.79 (dd, *J* = 17.0, 4.4 Hz, 1H), 2.62 (dd, *J* = 17.1, 6.4 Hz, 1H), 2.27 (dd, *J* = 11.1, 4.3 Hz, 1H), 2.21 (t, *J* = 10.4 Hz, 1H), 2.09 (s, 3H). ^13^C NMR (150 MHz, DMSO-*d*_6_) δ 208.3, 162.4, 136.3, 130.7, 130.7, 115.3, 115.2, 79.6, 71.9, 67.3, 57.3, 56.0, 51.9, 40.5, 30.6. ESI-HRMS: *m/z* calcd for C_15_H_21_NFO_4_ [M+H]^+^: 298.1449; found: 298.1459.

**(1R, 2S, 3R, 4R)-1-Acetylmethyl-2, 3, 4-trihydroxyl-*N*-2-fluorobenzylpiperidine (5y)**

colorless syrup, yield 52%, ${\left[\alpha \right]}_{20}^{D}$ −30.0 (*c* 0.06, CH_2_Cl_2_). ^1^H NMR (600 MHz, DMSO-*d*_6_) δ 7.39 (t, *J* = 7.0 Hz, 1H), 7.27 (dd, *J* = 13.3, 5.8 Hz, 1H), 7.20–7.05 (m, 2H), 3.69 (d, *J* = 19.8 Hz, 1H), 3.62 (d, *J* = 13.8 Hz, 1H), 3.41 (s, 1H), 3.22 (d, *J* = 13.7 Hz, 1H), 3.20–3.14 (m, 1H), 3.04–2.95 (m, 1H), 2.81 (dd, *J* = 17.2, 4.3 Hz, 1H), 2.64 (dd, *J* = 17.2, 6.2 Hz, 1H), 2.35–2.23 (m, 2H), 2.09 (d, *J* = 15.9 Hz, 3H). ^13^C NMR (150 MHz, DMSO-*d*_6_) δ 208.2, 162.0, 131.6, 129.2, 126.5, 124.6, 115.4, 72.1, 70.8, 67.4, 57.4, 52.1, 49.8, 40.6, 30.6. ESI-HRMS: *m/z* calcd for C_15_H_21_FNO_4_ [M+H]^+^: 298.1449; found: 298.1455.

**(1R, 2S, 3R, 4R)-1-Acetylmethyl-2, 3, 4-trihydroxyl-*N*-3-fluorobenzylpiperidine (5z)**

colorless syrup, yield 57%, ${\left[\alpha \right]}_{20}^{D}$ −35.0 (*c* 0.10, CH_2_Cl_2_). ^1^H NMR (600 MHz, DMSO-*d*_6_) δ 7.31 (dd, *J* = 14.2, 7.8 Hz, 1H), 7.11–7.06 (m, 2H), 7.02 (t, *J* = 7.5 Hz, 1H), 3.69 (d, *J* = 18.6 Hz, 1H), 3.65 (d, *J* = 14.0 Hz, 1H), 3.43 (d, *J* = 5.5 Hz, 1H), 3.20 (dd, *J* = 17.3, 8.7 Hz, 2H), 3.03 (d, *J* = 5.4 Hz, 1H), 2.79 (dd, *J* = 17.1, 4.2 Hz, 1H), 2.61 (dd, *J* = 17.1, 6.3 Hz, 1H), 2.32–2.22 (m, 2H), 2.08 (s, 3H). ^13^C NMR (150 MHz, DMSO-*d*_6_) δ 208.2, 161.9, 143.6, 130.4, 124.8, 115.2, 113.8, 72.0, 70.8, 67.4, 57.2, 56.3, 52.2, 49.1, 30.6. ESI-HRMS: *m/z* calcd for C_15_H_21_FNO_4_ [M+H]^+^: 298.1449; found: 298.1457.

**(1R, 2S, 3R, 4R)-1-Acetylmethyl-2, 3, 4-trihydroxyl-*N*-4-hydroxylbenzylpiperidine (5** **λ** **)**

colorless syrup, yield 48%, ${\left[\alpha \right]}_{20}^{D}$ +22.2 (*c* 0.18, CH_2_Cl_2_). ^1^H NMR (600 MHz, DMSO-*d*_6_) δ 7.03 (t, *J* = 15.2 Hz, 2H), 6.67 (t, *J* = 8.7 Hz, 2H), 3.70 (d, *J* = 14.8 Hz, 1H), 3.51 (t, *J* = 13.2 Hz, 1H), 3.38 (s, 1H), 3.17 (t, *J* = 14.1 Hz, 1H), 3.02–2.89 (m, 1H), 2.77 (d, *J* = 21.1 Hz, 1H), 2.64–2.55 (m, 1H), 2.29 (dd, *J* = 11.0, 4.2 Hz, 1H), 2.15 (t, *J* = 10.5 Hz, 1H), 2.08 (d, *J* = 12.3 Hz, 3H). ^13^C NMR (150 MHz, DMSO-*d*_6_) δ 208.4, 156.6, 130.1, 130.1, 130.1, 130.1, 115.3, 79.6, 72.0, 67.5, 57.3, 56.5, 51.8, 40.6, 30.6. ESI-HRMS: *m/z* calcd for C_15_H_22_NO_5_ [M+H]^+^: 296.1492; found: 296.1496.

**(1R, 2S, 3R, 4R)-1-Acetylmethyl-2, 3, 4-trihydroxyl-*N*-2-hydroxylbenzylpiperidine (5** **θ** **)**

colorless syrup, yield 41%, ${\left[\alpha \right]}_{20}^{D}$ −34.4 (*c* 0.16, CH_2_Cl_2_). ^1^H NMR (600 MHz, DMSO-*d*_6_) δ 7.08–7.00 (m, 2H), 6.73–6.66 (m, 2H), 3.75 (d, *J* = 13.9 Hz, 1H), 3.70 (s, 1H), 3.46 (s, 1H), 3.41–3.10 (m, 2H, overlap),3.04 (s, 1H), 2.71 (dd, *J* = 20.8, 5.8 Hz, 1H), 2.60 (s, 1H), 2.43 (dd, *J* = 11.4, 3.8 Hz, 1H), 2.34 (dd, *J* = 20.1, 10.6 Hz, 1H), 2.07 (s, 3H). ^13^C NMR (150 MHz, DMSO-*d*_6_) δ 207.9, 157.2, 129.5, 128.5, 123.6, 119.2, 115.8, 79.6, 71.6, 67.5, 57.3, 54.8, 52.0, 40.5, 30.6. ESI-HRMS: *m/z* calcd for C_15_H_2_1O_5_NNa [M+Na]^+^: 318.1318; found: 318.1312.

**(1R, 2S, 3R, 4R)-1-Acetylmethyl-2, 3, 4-trihydroxyl-*N*-3-hydroxylbenzylpiperidine (5** **π** **)**

colorless syrup, yield 45%, ${\left[\alpha \right]}_{20}^{D}$ +21.1 (*c* 0.08, MeOH). ^1^H NMR (600 MHz, CD_3_OD) δ 7.13 (t, *J* = 8.0 Hz, 1H), 6.80 (d, *J* = 6.6 Hz, 2H), 6.70 (d, *J* = 7.6 Hz, 1H), 3.91 (s, 1H), 3.80 (d, *J* = 13.0 Hz, 1H), 3.72 (s, 1H), 3.51 (s, 1H), 3.41 (d, *J* = 13.6 Hz, 1H), 2.89 (d, *J* = 5.4 Hz, 1H), 2.70–2.62 (m, 1H), 2.57 (s, 1H), 2.19 (s, 3H). ^13^C NMR (150 MHz, CD_3_OD) δ 209.0, 157.3, 129.1, 129.1, 120.1, 115.7, 114.2, 71.3, 67.2, 63.7, 57.6, 57.2, 50.8, 48.4, 29.0. ESI-HRMS: *m/z* calcd for C_15_H_21_NO_4_Na [M+Na]^+^: 318.1312; found: 318.1308.

**(1R, 2S, 3R, 4R)-1-Acetylmethyl-2, 3, 4-trihydroxyl-*N*-3-chloro-4-hydroxylbenzylpiperidine (6a)**

colorless syrup, yield 45%, ${\left[\alpha \right]}_{20}^{D}$ +13.3 (*c* 0.12, MeOH). ^1^H NMR (600 MHz, CD_3_OD) δ 7.25 (d, *J* = 1.9 Hz, 1H), 7.05 (dd, *J* = 8.2, 1.9 Hz, 1H), 6.84 (d, *J* = 8.3 Hz, 1H), 3.86 (s, 1H), 3.64 (dd, *J* = 10.9, 7.4 Hz, 2H), 3.42 (d, *J* = 8.0 Hz, 1H), 3.26–3.15 (m, 2H), 2.89–2.82 (m, 1H), 2.78 (dd, *J* = 17.2, 5.7 Hz, 1H), 2.54 (dd, *J* = 11.5, 4.0 Hz, 1H), 2.47–2.39 (m, 1H), 2.18 (s, 3H). ^13^C NMR (150 MHz, CD_3_OD) δ 209.2, 152.0, 130.9, 130.0, 128.2, 120.1, 115.9, 72.0, 70.1, 67.6, 57.3, 56.0, 50.9, 48.4, 28.9. ESI-HRMS: *m/z* calcd for C_15_H_21_ClNO_5_ [M+H]^+^: 330.1103; found: 330.1108.

**(1R, 2S, 3R, 4R)-1-Acetylmethyl-2, 3, 4-trihydroxyl-*N*-3-bromo-4-hydroxylbenzylpiperidine (6b)**

colorless syrup, yield 47%, ${\left[\alpha \right]}_{20}^{D}$ +11.5 (*c* 0.13, MeOH). ^1^H NMR (600 MHz, CD_3_OD) δ 7.42 (d, *J* = 1.9 Hz, 1H), 7.09 (dd, *J* = 8.2, 1.9 Hz, 1H), 6.83 (d, *J* = 8.2 Hz, 1H), 3.86 (s, 1H), 3.64 (t, *J* = 10.9 Hz, 2H), 3.42 (d, *J* = 5.7 Hz, 1H), 3.25–3.17 (m, 2H), 2.88–2.83 (m, 1H), 2.78 (dd, *J* = 17.2, 5.8 Hz, 1H), 2.55 (dd, *J* = 11.6, 4.0 Hz, 1H), 2.47–2.38 (m, 1H), 2.18 (s, 3H). ^13^C NMR (150 MHz, CD_3_OD) δ 209.2, 153.1, 133.1, 131.3, 128.9, 115.6, 109.3, 72.0, 70.1, 67.6, 57.2, 55.9, 50.9, 48.2, 29.0. ESI-HRMS: *m/z* calcd for C_15_H_21_BrNO_5_ [M+H]^+^: 374.0598; found: 374.0598.

**(1R, 2S, 3R, 4R)-1-Acetylmethyl-2, 3, 4-trihydroxyl-*N*-3-fluoro-4-hydroxylbenzylpiperidine (6c)**

colorless syrup, yield 52%, ${\left[\alpha \right]}_{20}^{D}$ +13.6 (*c* 0.16, MeOH). ^1^H NMR (600 MHz, CD_3_OD) δ 7.06–7.01 (m, 1H), 6.90 (d, *J* = 8.3 Hz, 1H), 6.83 (t, *J* = 8.6 Hz, 1H), 3.85 (d, *J* = 14.6 Hz, 1H), 3.65 (t, *J* = 10.1 Hz, 2H), 3.47–3.39 (m, 1H), 3.25 (d, *J* = 13.4 Hz, 1H), 3.20 (d, *J* = 19.1 Hz, 1H), 2.85 (dd, *J* = 17.4, 5.3 Hz, 1H), 2.78 (dd, *J* = 17.1, 5.7 Hz, 1H), 2.55 (dd, *J* = 11.6, 4.0 Hz, 1H), 2.48–2.40 (m, 1H), 2.17 (s, 3H). ^13^C NMR (150 MHz, CD_3_OD) δ 209.8, 154.5, 150.7, 142.9, 130.5, 126.2, 117.9, 71.9, 70.0, 67.6, 56.1, 53.8, 50.9, 48.5, 28.9. ESI-HRMS: *m/z* calcd for C_15_H_20_NFO_5_Na [M+Na]^+^: 336.1218; found: 336.1209.

**(1R, 2S, 3R, 4R)-1-Acetylmethyl-2, 3, 4-trihydroxyl-*N*-2-chloro-4-hydroxylbenzylpiperidine (6d)**

colorless syrup, yield 48%, ${\left[\alpha \right]}_{20}^{D}$ +14.2 (*c* 0.14, MeOH). ^1^H NMR (600 MHz, CD_3_OD) δ 7.27 (d, *J* = 8.4 Hz, 1H), 6.79 (d, *J* = 2.4 Hz, 1H), 6.71–6.68 (m, 1H), 3.85 (s, 1H), 3.72–3.67 (m, 1H), 3.66–3.61 (m, 1H), 3.43 (d, *J* = 5.3 Hz, 1H), 3.39 (d, *J* = 13.6 Hz, 1H), 3.27 (d, *J* = 6.8 Hz, 1H), 2.85 (d, *J* = 5.6 Hz, 2H), 2.58 (dd, *J* = 11.6, 4.0 Hz, 1H), 2.54–2.47 (m, 1H), 2.18 (s, 3H). ^13^C NMR (150 MHz, CD_3_OD) δ 209.2, 157.2, 134.3, 131.8, 126.4, 115.7, 113.8, 71.9, 69.9, 67.7, 57.6, 53.5, 50.9, 48.5, 28.9. ESI-MS: *m/z* calcd for C_15_H_20_NClO_5_Na [M+ Na]^+^: 352.0922; found: 352.0924.

**(1R, 2S, 3R, 4R)-1-Acetylmethyl-2, 3, 4-trihydroxyl-*N*-2, 6-dichloro-4-hydroxylbenzylpiperidine (6e)**

colorless syrup, yield 39%, ${\left[\alpha \right]}_{20}^{D}$ +6.67 (*c* 0.06, MeOH). ^1^H NMR (600 MHz, DMSO-*d*_6_) δ 6.80 (s, 1H), 6.78 (s, 1H), 3.71 (s, 1H), 3.65 (s, 1H), 3.56 (dd, *J* = 9.6, 5.8 Hz, 1H), 3.51 (d, *J* = 12.5 Hz, 1H), 3.15 (s, 1H), 3.13–3.04 (m, 2H), 2.99 (s, 1H), 2.80 (d, *J* = 6.5 Hz, 1H), 2.39–2.27 (m, 2H), 2.14 (s, 3H). ^13^C NMR (150 MHz, DMSO-*d*_6_) δ 208.3, 158.3, 136.8, 124.5, 118.4, 116.1, 115.2, 72.9, 71.5, 68.0, 58.9, 51.9, 51.1, 48.5, 30.6. ESI-MS: *m/z* calcd for C_15_H_24_Cl_2_NO_5_ [M+H]^+^: 364.0713; found: 364.0704.

**(1R, 2S, 3R, 4R)-1-Acetylmethyl-2, 3, 4-trihydroxyl-*N*-4-hydroxyl-methoxylbenzylpiperidine (6f)**

colorless syrup, yield 53%, ${\left[\alpha \right]}_{20}^{D}$ +3.3 (*c* 0.24, MeOH). ^1^H NMR (600 MHz, CD_3_OD) δ 6.93 (s, 1H), 6.73 (s, 2H), 3.87 (s, 1H), 3.83 (d, *J* = 8.4 Hz, 2H), 3.71 (t, *J* = 14.8 Hz, 1H), 3.69–3.63 (m, 1H), 3.45 (d, *J* = 6.0 Hz, 1H), 3.34 (s, 3H), 3.24 (d, *J* = 20.5 Hz, 1H), 2.85 (t, *J* = 7.7 Hz, 1H), 2.61 (dd, *J* = 11.6, 4.0 Hz, 1H), 2.48 (t, *J* = 10.4 Hz, 1H), 2.18 (s, 3H). ^13^C NMR (150 MHz, CD_3_OD) δ 209.1, 147.6, 145.6, 129.1, 121.6, 114.5, 112.4, 71.8, 70.0, 67.4, 56.9, 55.0, 55.0, 50.8, 48.5, 29.0. ESI-HRMS: *m/z* calcd for C_16_H_23_NO_6_Na [M+ Na]^+^: 348.1418; found: 348.1410.

**(1R, 2S, 3R, 4R)-1-Acetylmethyl-2, 3, 4-trihydroxyl-*N*-3-ethyoxyl-4-hydroxylbenzylpiperidine (6g)**

colorless syrup, yield 44%, ${\left[\alpha \right]}_{20}^{D}$ +11.0 (*c* 0.10, MeOH). ^1^H NMR (600 MHz, DMSO-*d*_6_) δ 6.78 (s, 1H), 6.71–6.65 (m, 1H), 6.60 (d, *J* = 7.5 Hz, 1H), 3.96 (dd, *J* = 13.0, 6.2 Hz, 2H), 3.69 (s, 1H), 3.52 (d, *J* = 13.2 Hz, 1H), 3.41 (s, 1H), 3.18 (s, 1H), 3.06–2.92 (m, 2H), 2.76 (dd, *J* = 16.9, 4.2 Hz, 1H), 2.61 (dd, *J* = 16.8, 6.2 Hz, 1H), 2.32 (dd, *J* = 11.2, 4.0 Hz, 1H), 2.18 (t, *J* = 10.2 Hz, 1H), 2.09 (s, 3H), 1.30 (t, *J* = 6.9 Hz, 3H). ^13^C NMR (150 MHz, DMSO-*d*_6_) δ 208.3, 146.9, 146.1, 130.7, 121.4, 115.6, 114.5, 72.1, 67.6, 64.3, 57.4, 56.7, 56.7, 51.8, 44.5, 30.6, 15.3. ESI-HRMS: *m/z* calcd for C_17_H_25_NO_6_Na [M+Na]^+^: 362.1574; found: 362.1573.

**(1R, 2S, 3R, 4R)-1-Acetylmethyl-2, 3, 4-trihydroxyl-*N*-3-hydroxyl-4-methoxylbenzylpiperidine (6h)**

colorless syrup, yield 45%, ${\left[\alpha \right]}_{20}^{D}$ +21.6 (*c* 0.12, MeOH). ^1^H NMR (600 MHz, CD_3_OD) δ 6.86 (d, *J* = 8.2 Hz, 1H), 6.82 (d, *J* = 1.8 Hz, 1H), 6.75 (d, *J* = 8.2 Hz, 1H), 3.88 (s, 1H), 3.83 (s, 3H), 3.72 (d, *J* = 13.1 Hz, 1H), 3.66 (dd, *J* = 9.1, 3.4 Hz, 1H), 3.48–3.43 (m, 1H), 3.28 (d, *J* = 13.3 Hz, 1H), 3.26–3.17 (m, 1H), 2.89 (dd, *J* = 17.3, 5.0 Hz, 1H), 2.84 (dd, *J* = 17.3, 5.6 Hz, 1H), 2.61 (dd, *J* = 11.6, 4.0 Hz, 1H), 2.52–2.44 (m, 1H), 2.19 (s, 3H). ^13^C NMR (150 MHz, CD_3_OD) δ 209.2, 147.1, 146.2, 130.5, 120.2, 115.8, 111.2, 71.6, 68.7, 67.3, 57.4, 56.8, 55.1, 50.8, 48.2, 29.0. ESI-MS: *m/z* calcd for C_16_H_23_NO_6_Na [M+Na]^+^: 348.1418; found: 348.1410.

**(1R, 2S, 3R, 4R)-1-Acetylmethyl-2, 3, 4-trihydroxyl-*N*-3, 5-dibromo-4-hydroxylbenzylpiperidine (6i)**

colorless syrup, yield 42%, ${\left[\alpha \right]}_{20}^{D}$ +13.8 (*c* 0.08, MeOH). ^1^H NMR (600 MHz, DMSO-*d*_6_) δ 7.40 (s, 2H), 3.69 (s, 1H), 3.53 (d, *J* = 13.7 Hz, 1H), 3.42 (s, 1H), 3.18 (s, 1H), 3.08 (d, *J* = 13.8 Hz, 1H), 2.97 (s, 1H), 2.77 (d, *J* = 17.3 Hz, 1H), 2.62 (dd, *J* = 17.1, 6.4 Hz, 1H), 2.28 (d, *J* = 7.5 Hz, 1H), 2.24 (d, *J* = 9.9 Hz, 1H), 2.08 (d, *J* = 6.7 Hz, 3H). ^13^C NMR (150 MHz, DMSO-*d*_6_) δ 208.2, 170.8, 149.8, 132.6, 132.6, 112.2, 112.2, 73.2, 72.0, 67.6, 60.2, 55.4, 51.9, 49.1, 30.6. ESI-HRMS: *m/z* calcd for C_15_H_19_NBr_2_O_5_Na [M+Na]^+^: 473.9522; found: 473.9515.

### 3.2. α-Glucosidase Inhibitory Activity Test

The extracts of each sample were dissolved in distilled water to prepare a certain concentration. 150 µL of the sample solution was mixed with 150 µL of maltose solution (prepared with 0.2 M, pH = 6.8 phosphate buffer) at a certain concentration, and cultured at 37 °C for 10 min. Then 150 µL of the enzyme solution at a certain concentration was added and mixed. The reaction was carried out at 37 °C for 60 min. The reaction was terminated in a boiling water bath for 10 min and centrifuged at 3000 r/min for 10 min. The supernatant was taken to determine the content of glucose with Glu kit. Acarbose solution was used as positive control, and blank control (distilled water instead of enzyme solution and sample solution) and negative control (distilled water instead of sample solution) were set. Enzyme activity inhibition rate (%) = (negative-sample)/(negative-blank) × 100%.

Enzyme kinetics experiment: The principle of the experimental method is the same as that of the above method, in which the micro-membrane centrifuge tube with a pore diameter of 3K is used for small molecule centrifugal separation. In the enzyme kinetics experiment, the concentration of enzyme was set at a fixed concentration, and five concentration gradients were designed for the solution of substrate maltose. The linear relationship was plotted using the obtained results combined with the Michaelis-Menten equation.

## 4. Conclusions

In summary, a series of *N*-substituted iminosugar *C*-glycosides were synthesized, and their *α*-glucosidase inhibition was assessed. Compound **6e** showed stronger inhibitory potency than positive control Acarbose. Besides, the inhibition of compound **6d** was equivalent to positive control Acarbose. Enzymatic kinetic assays indicated that compound **6e** may be classified as a reversible and uncompetitive inhibitor. The study of structure-activity relationships provided a starting point for the discovery of new more promising *α*-glucosidase inhibitors. Moreover, the experimental result was in good agreement with the docking result that presented evidence for the inhibition mechanism of compound **6e**. Most importantly, compounds **6d** and **6e** were identified to have excellent performance in drug-likeness and pharmacokinetic properties in Silico Analysis, which revealed excellent prospects for the development of new *α*-glucosidase inhibitors. In addition, the influence of the iminosugar core on the inhibitory activity against *α*-glucosidase is being carried out.

## Figures and Tables

**Table 1:** Screening of synthesis conditions of *N*-substituted iminosugar *C*-glycosides.

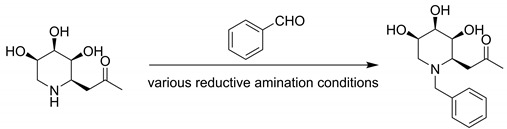				
**Reducing Agent**	**Temperature**	**Solvent**	**Time**	**Yield**
NaBH_3_CN	r. t.	MeOH	8 h	N. R.
NaBH_3_CN	0 °C	MeOH	8 h	N. R.
NaBH_3_CN	−40 °C	MeOH	8 h	N. R.
NaBH_3_CN	−78 °C	MeOH	8 h	N. R.
NaBH_3_CN	−20 °C–r. t.	HOAc, MeOH	8 h	N. R.
NaBH(OAc)_3_	0 °C–r. t.	MeOH	3 h	67%
NaBH(OAc)_3_	−20 °C–r. t.	MeOH	3 h	93%

**Table 2:** The IC_50_ value of the compounds against *α*-glucosidase from yeast.

Compound	IC_50_ (µM)	Compound	IC_50_ (µM)
**5r**	12.0	**6d**	2.4
**5λ**	7.4	**6e**	0.9
**5θ**	>20.0	**6f**	>20.0
**5π**	>20.0	**6g**	>20.0
**6a**	5.6	**6h**	>20.0
**6b**	5.3	**6i**	5.5
**6c**	3.3	**4**	>20.0
Acarbose	2.0		

**Table 3:** Physiochemical properties of the compounds predicted by SwissADME and admetSAR.

Compound Name	MW (g/mol)	nAtoms	nRings	nCarbon	nHetero Atoms	RB	HBA	HBD	MR	TPSA (Å sqr)	XlogP	WlogP	MlogP
**5r**	313.78	41	2	15	6	4	5	3	83.26	81.00	0.03	0.05	0.40
**5λ**	295.33	42	2	15	6	4	6	4	80.27	101.23	−0.96	−0.89 *	−0.66
**5θ**	295.33	42	2	15	6	4	6	4	80.27	101.23	−0.96	−0.89 *	−0.66
**5π**	295.33	42	2	15	6	4	6	4	80.27	101.23	−0.96	−0.89 *	−0.66
**6a**	329.78	42	2	15	7	4	6	4	85.28	101.23	−0.33	−0.24	−0.14
**6b**	374.23	42	2	15	7	4	6	4	87.97	101.23	−0.26	−0.13	−0.02
**6c**	313.32	42	2	15	7	4	7	4	80.23	101.23	−0.86	−0.34	−0.27
**6d**	329.78	42	2	15	7	4	6	4	85.28	101.23	−0.33	−0.24	−0.14
**6e**	364.22	42	2	15	8	4	6	4	90.29	101.23	0.30	0.41	0.37
**6f**	325.36	46	2	16	7	5	7	4	86.76	110.46	−0.98	−0.89 *	−0.94
**6g**	339.38	49	2	17	7	6	7	4	91.57	110.46	−0.62	−0.50 *	−0.70
**6h**	325.36	46	2	16	7	5	7	4	86.76	110.46	−0.98	−0.89 *	−0.94
**6i**	453.12	42	2	15	8	4	6	4	95.67	101.23	0.43	0.63	0.61

(RB: Rotatable Bonds; HBA: H-Bond Acceptor; HBD: H-Bond Donor; MR: Molecular Refractivity. *: Did not comply with “−0.4 ≤ WlogP ≤ 5.6” as specified in the Ghose rule.).

**Table 4:** ADMET score for human intestinal absorption, Caco-2 permeability, blood brain barrier, carcinogenicity, ames mutagenesis and acute oral toxicity as calculated by admetSAR.

Compound Name	Human Intestinal Absorption	Caco-2 Permeability	Blood Brain Barrier	Carcinogenicity	Ames Mutagenesis	Acute Oral Toxicity	Average Score
**6d**	0.9666	0.6029	0.9472	0.8429	0.5900	0.6726	0.7704
**6e**	0.9666	0.6770	0.9472	0.8429	0.6500	0.6726	0.7927

## Data Availability

The details of the data supporting the report results in this research, were included in the paper and Supplementary Materials.

## Supplementary Materials

The following supporting information can be downloaded at: https://www.mdpi.com/article/10.3390/molecules27175517/s1. Figures S1–S117: ^1^H NMR and ^13^C NMR spectra of *N*-substituted iminosugar *C*-glycosides (**5a**–**5π**); Figures S118–S153: ^1^H NMR and ^13^C NMR spectra of *N*-substituted iminosugar *C*-glycosides (**6a**–**6i**).
