# Supplementary material for: Synthesis of N-Substituted Iminosugar C-Glycosides and Evaluation as Promising α-Glucosidase Inhibitors
Source: Molecules. 2022 Aug 27;27(17):5517. doi: 10.3390/molecules27175517 (PMC9458058; doi:10.3390/molecules27175517)
Supplement: Supplementary file 1 [file molecules-27-05517-s001.zip › Supplementary information.pdf]

Synthesis of *N*-substituted iminosugar *C*-glycosides and evaluation as promising *α*-glucosidase inhibitors

Haibo Wang[1,2,3,†], Senling Tang[1,2,†], Guoqing Zhang[1,4], Yang Pan[1,2], Wei Jiao[1,*] and Huawu Shao[1,*]

[1] Natural Products Research Centre, Chengdu Institute of Biology, Chinese Academy of Sciences, Chengdu 610041, China
[2] University of Chinese Academy of Sciences, Beijing 100049, China
[3] Zhejiang Hongyuan Pharmaceutical Co., Ltd., Linhai 317016, China
[4] School of Pharmacy, North Sichuan Medical College, Nanchong 637100, China
[*] Correspondence: jiaowei@cib.ac.cn (W.J.); shaohw@cib.ac.cn (H.S.)
[†] These authors contributed equally to this work.

Contents:

1. ^{1}H NMR and ^{13}C NMR spectra of *N*-substituted iminosugar *C*-glycosides(**5a**-**5π**) ……………………………………………………………………………….…2
2. ^{1}H NMR and ^{13}C NMR spectra of *N*-substituted iminosugar *C*-glycosides(**6a**-**6i**) ………………………………………………………………...………...…..119

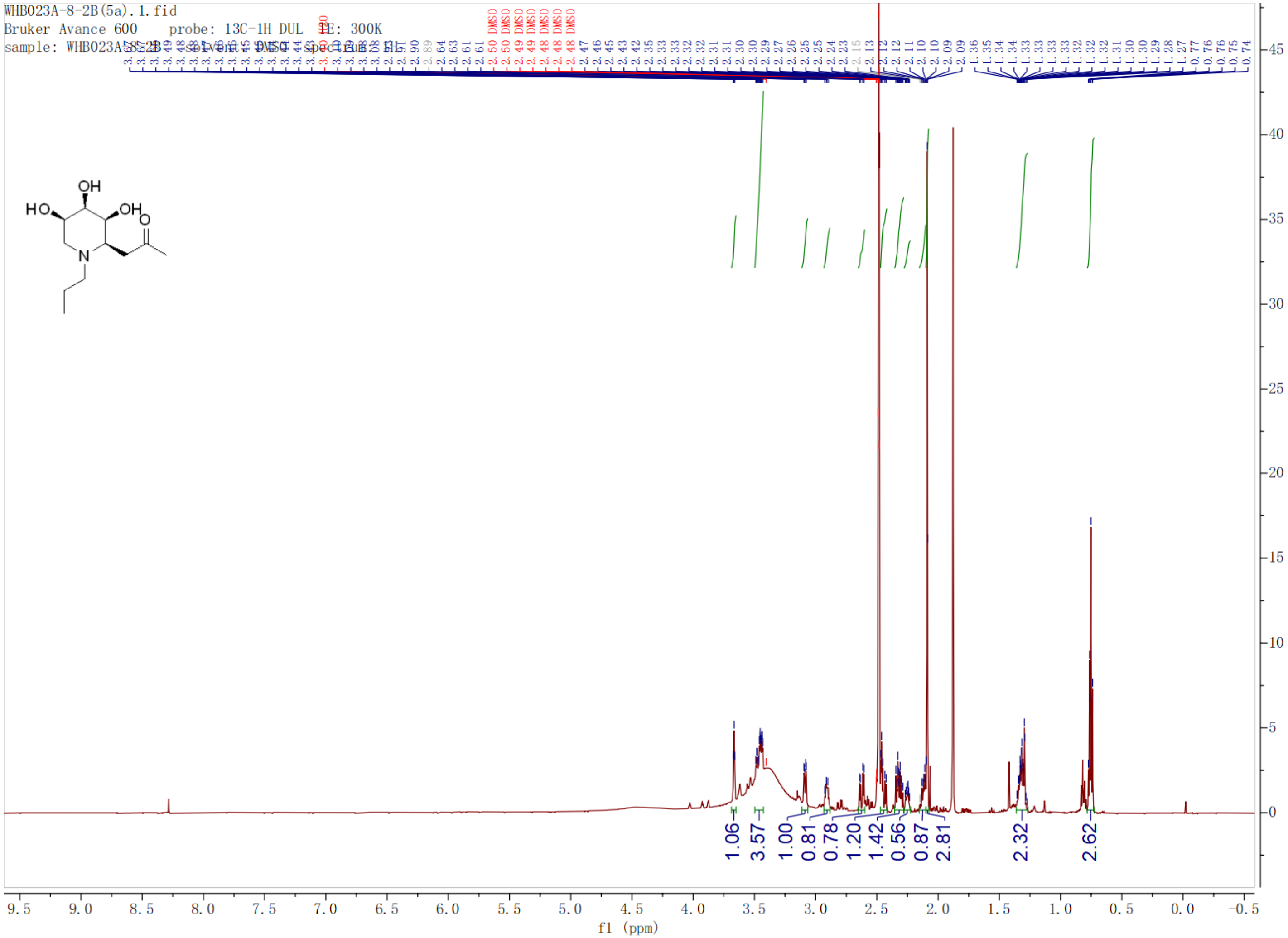

Figure S1. The ^{1}H NMR of compound **5a**.

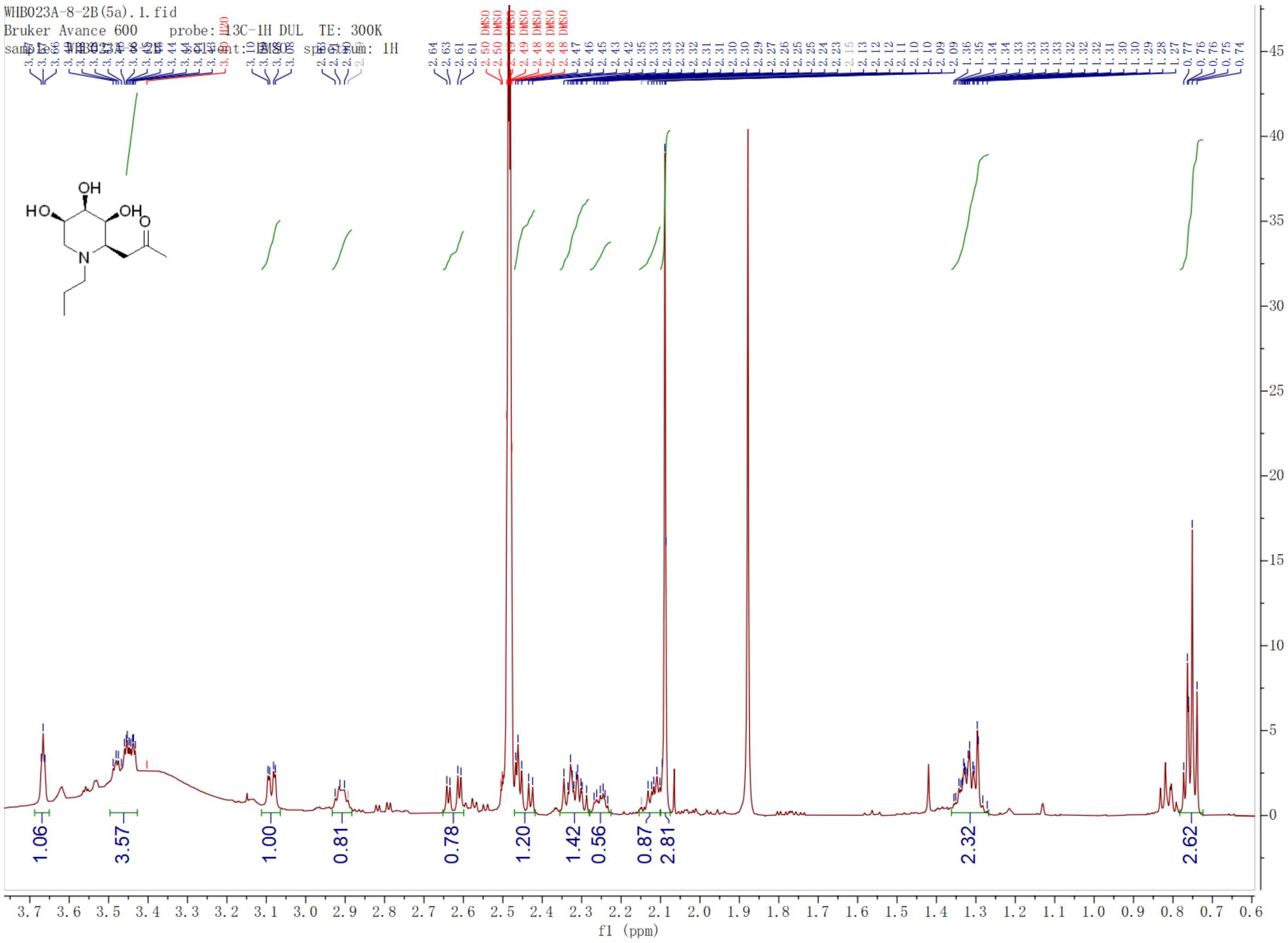

Figure S2. Partial region amplification of the ^{1}H NMR of compound **5a**.

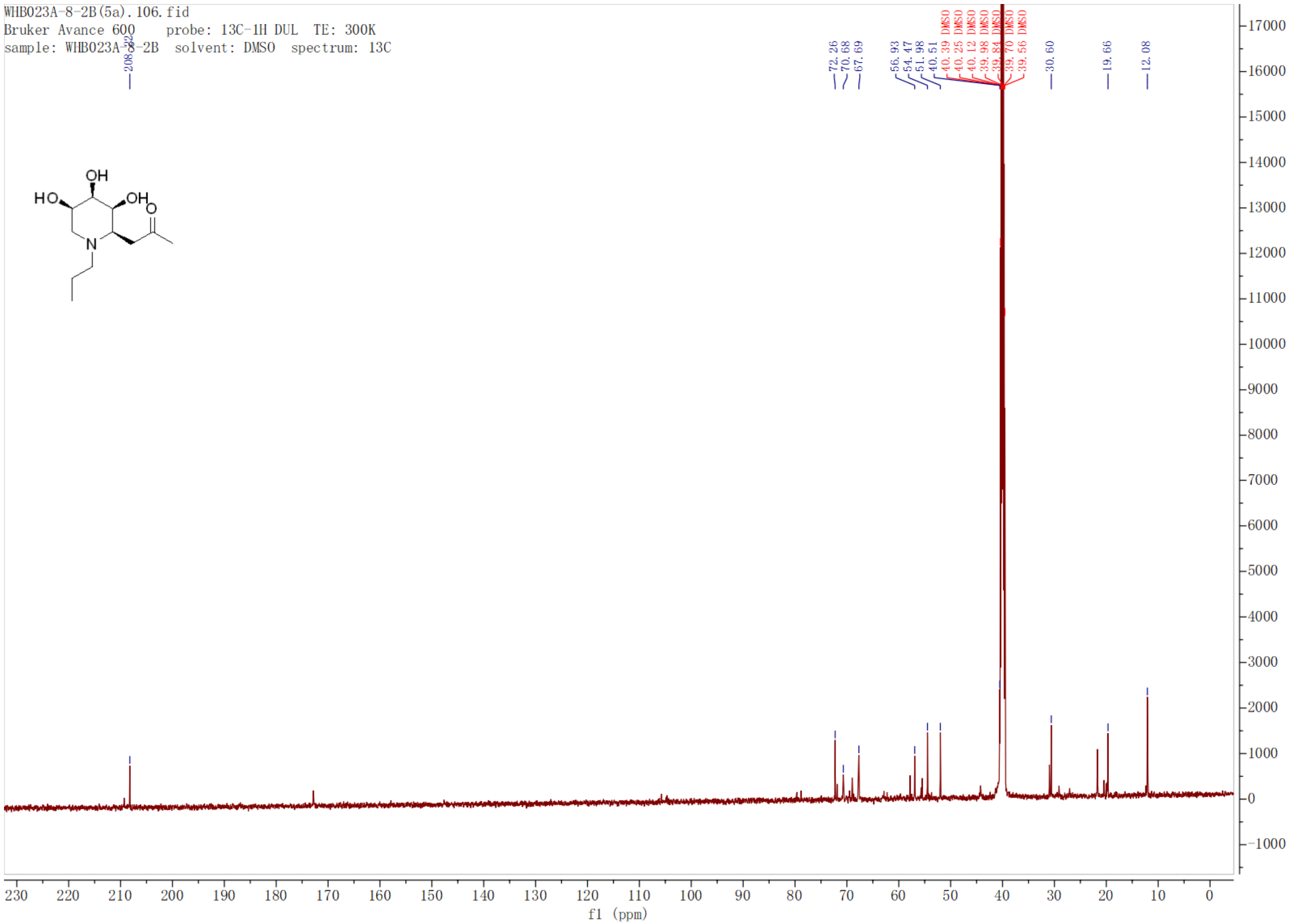

Figure S3. The ^{13}C NMR of compound **5a**.

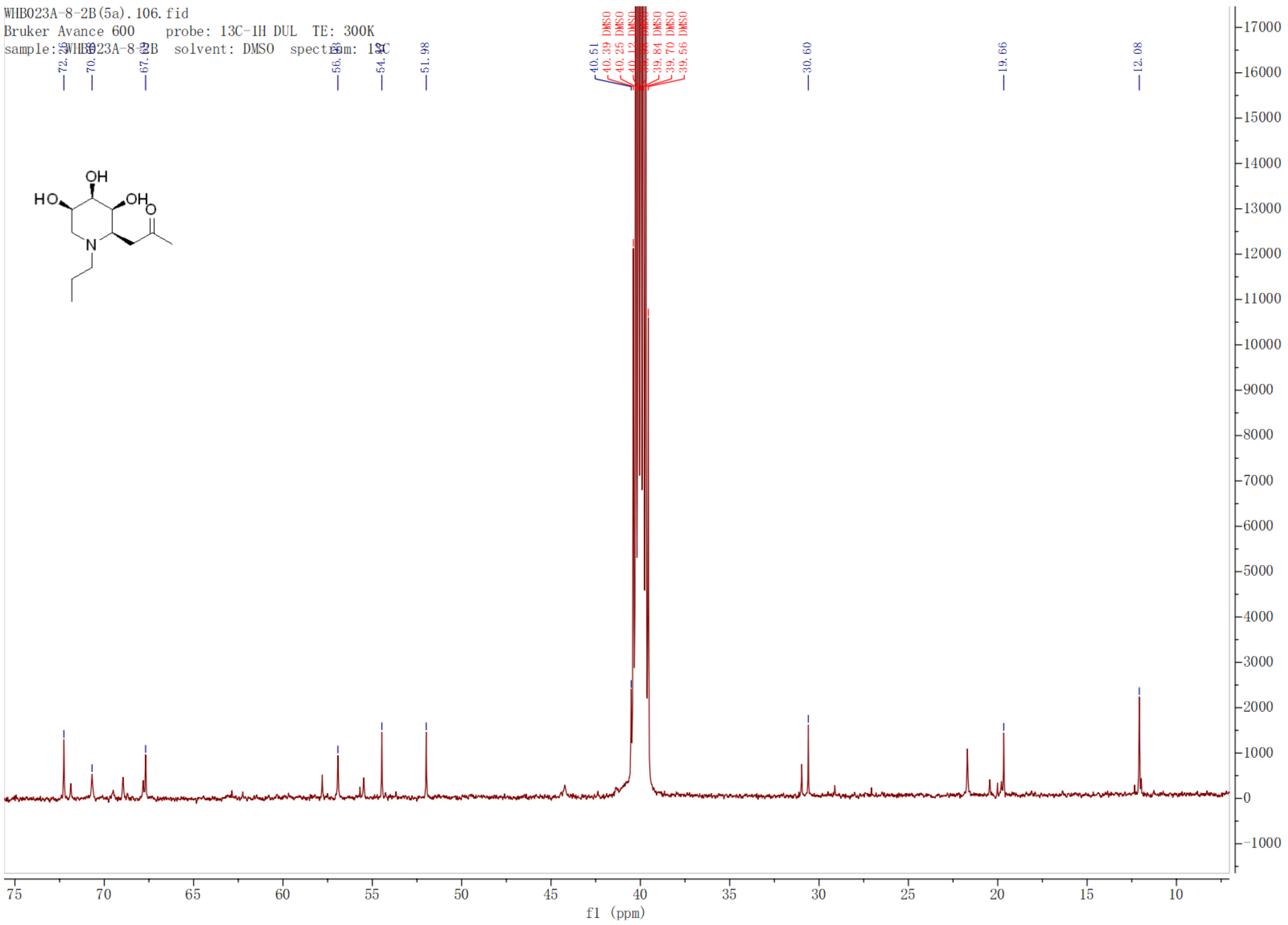

Figure S4. Partial region amplification of the ^{13}C NMR of compound **5a**.

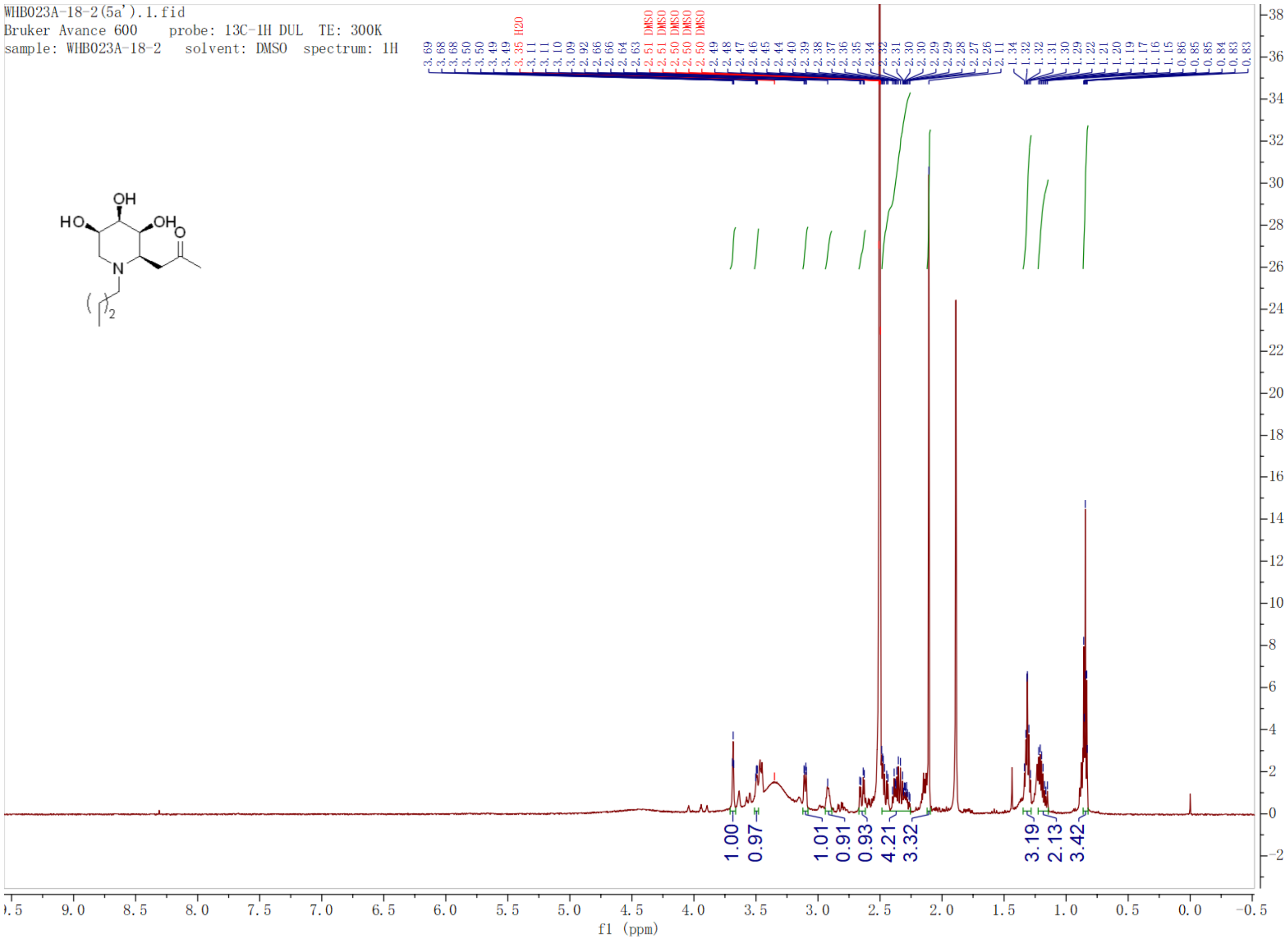

Figure S5. The ^{1}H NMR of compound **5b**.

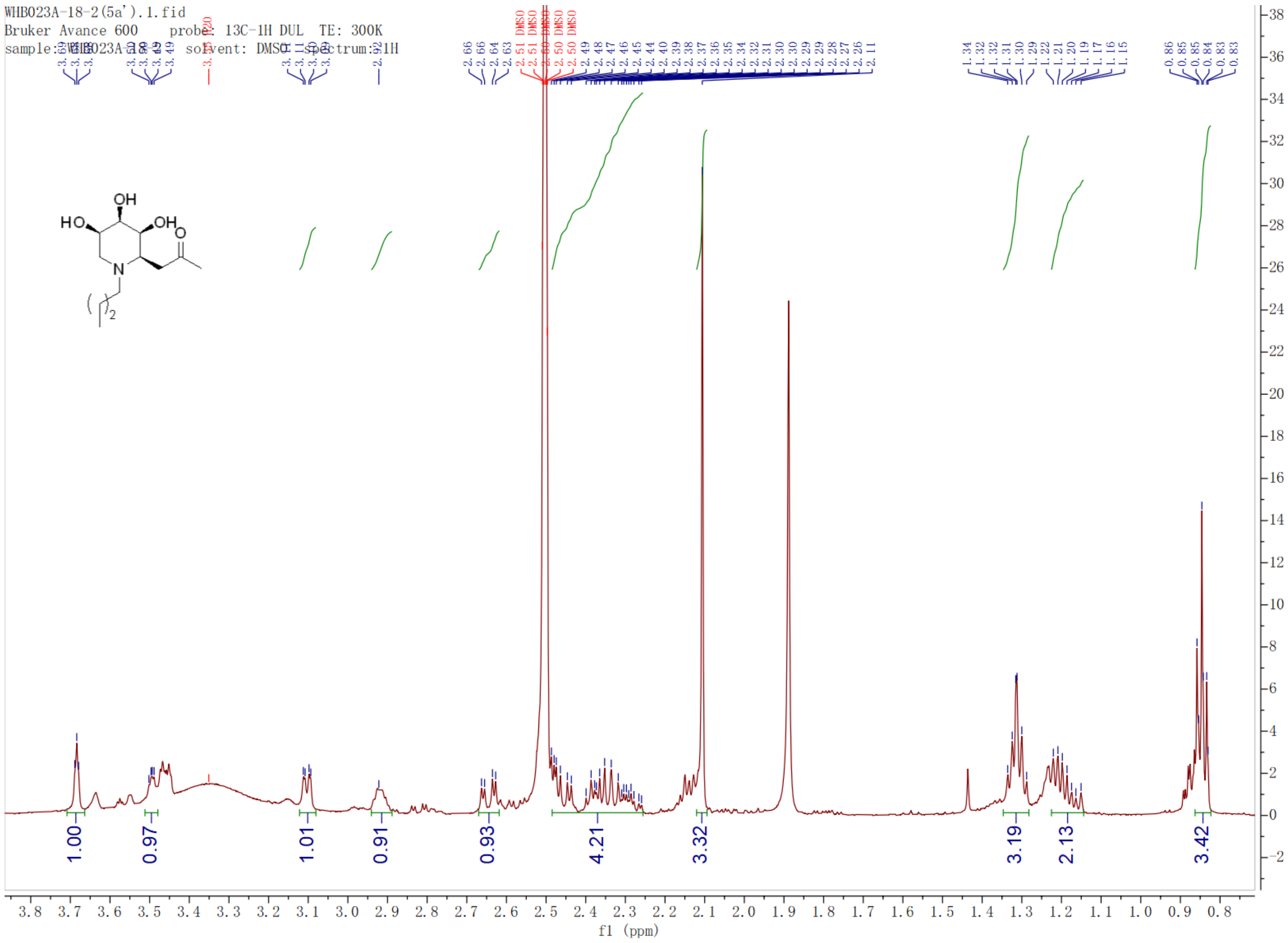

Figure S6. Partial region amplification of the ^{1}H NMR of compound **5b**.

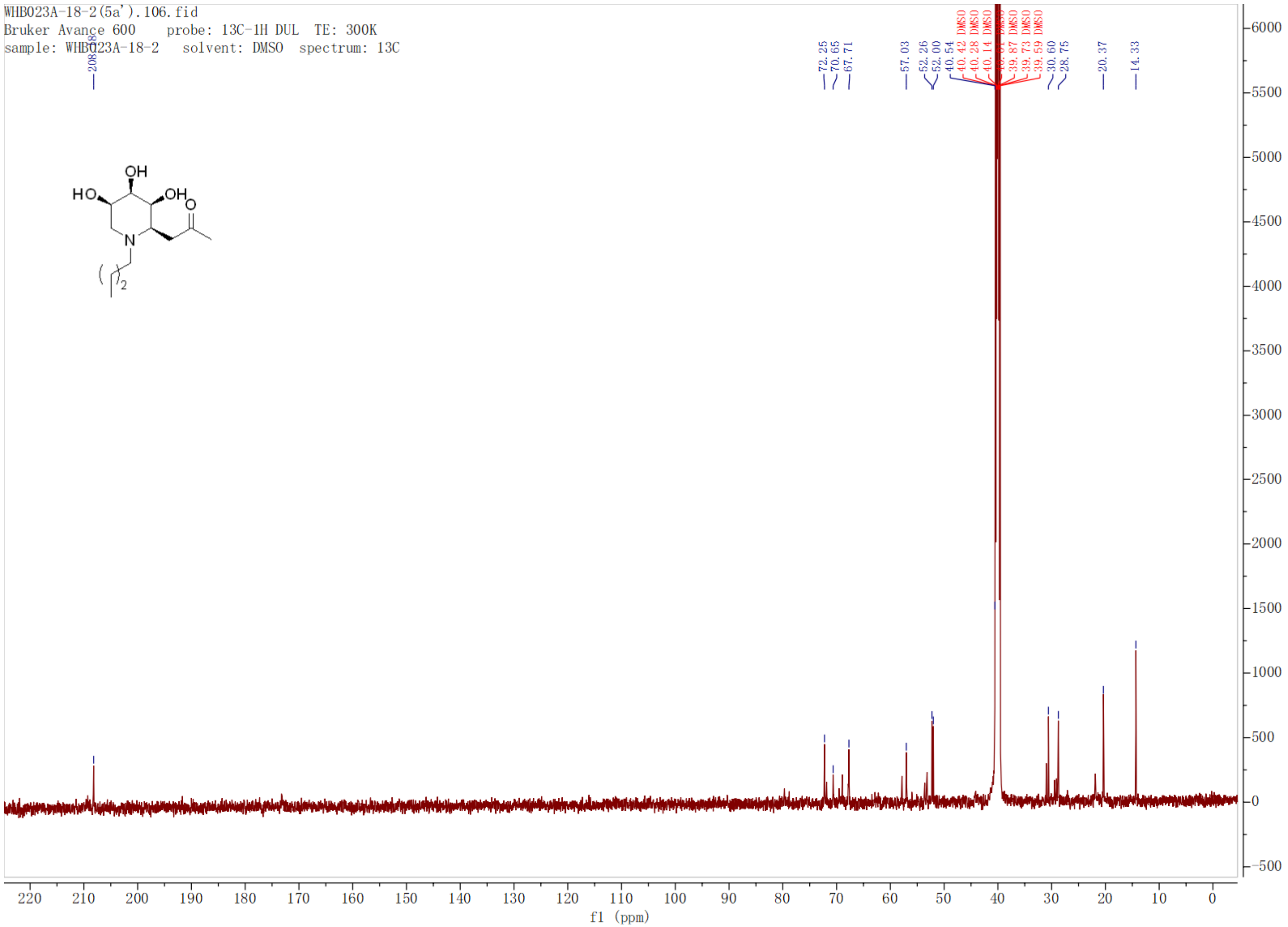

Figure S7. The ^{13}C NMR of compound **5b**.

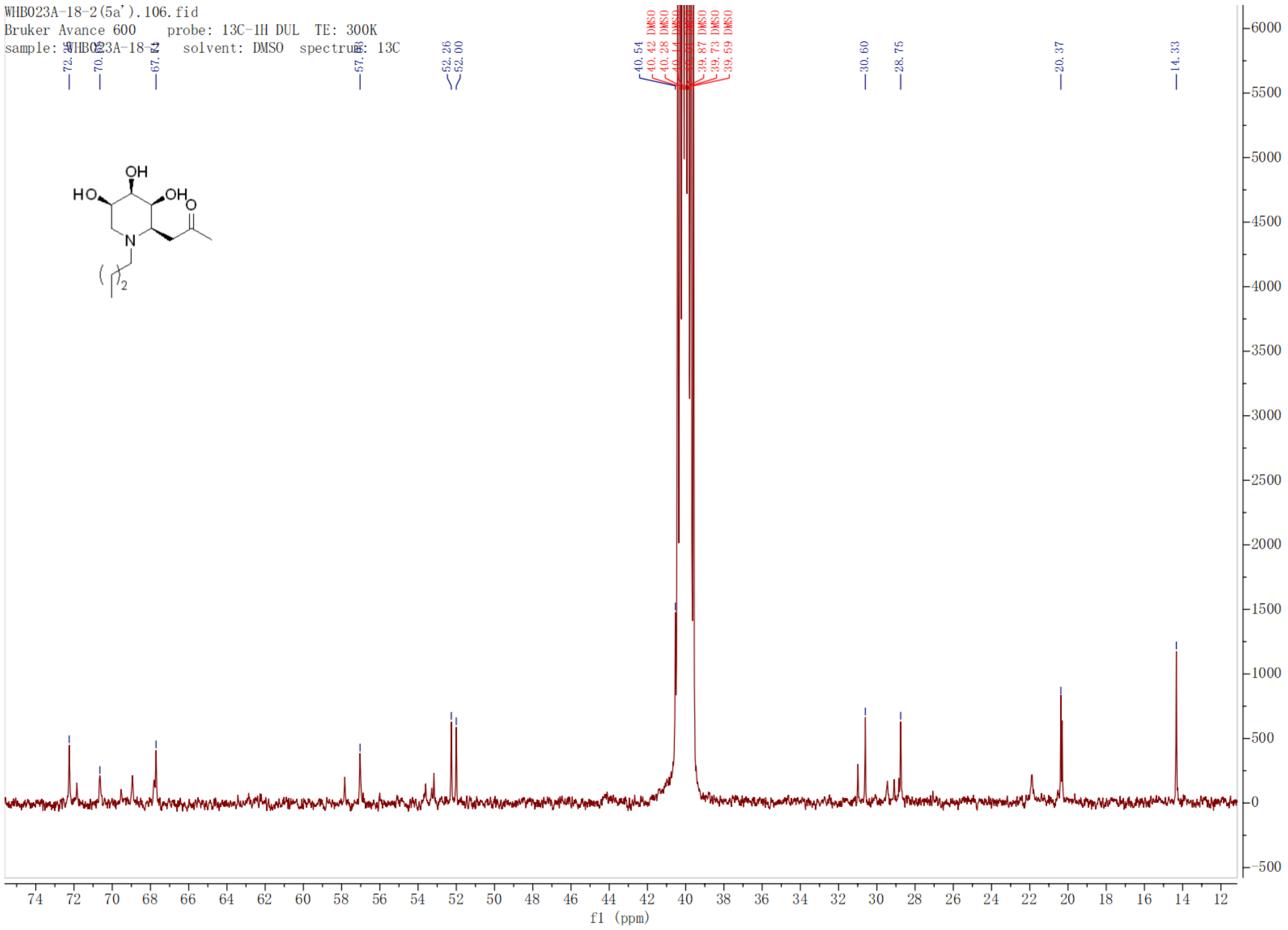

Figure S8. Partial region amplification of the ^{13}C NMR of compound **5b**.

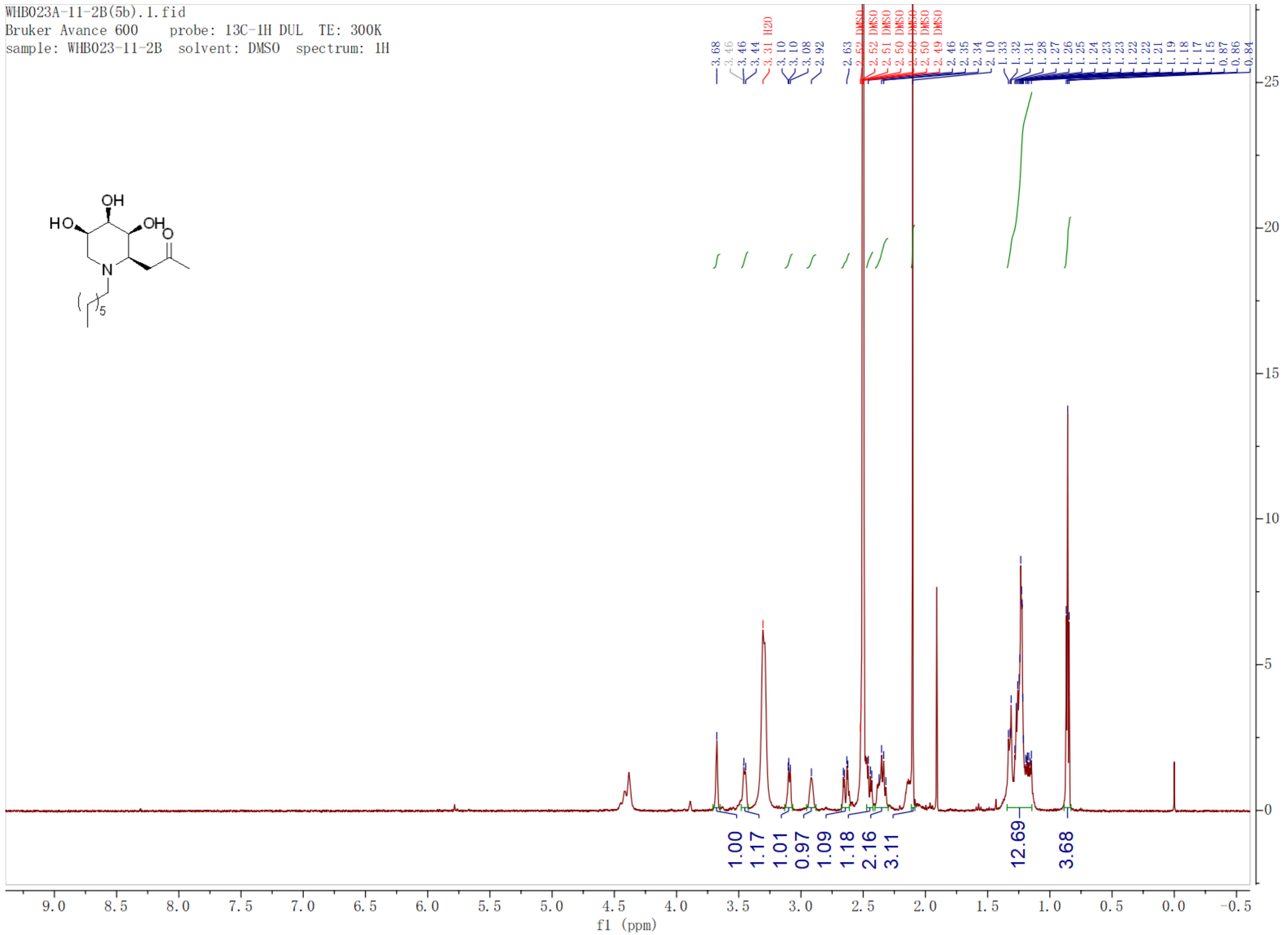

Figure S9. The ^{1}H NMR of compound **5c**.

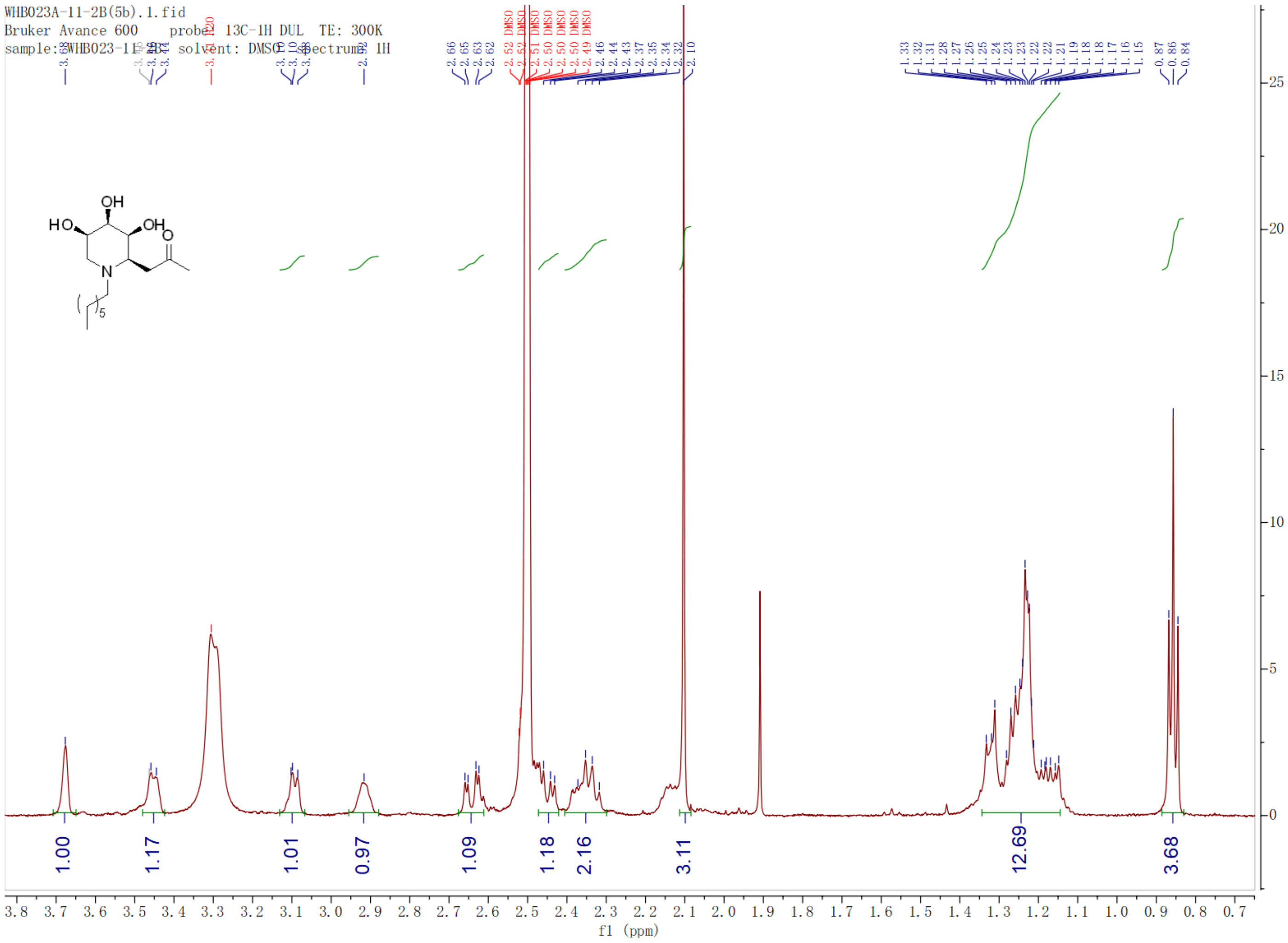

Figure S10. Partial region amplification of the ^{1}H NMR of compound **5c**.

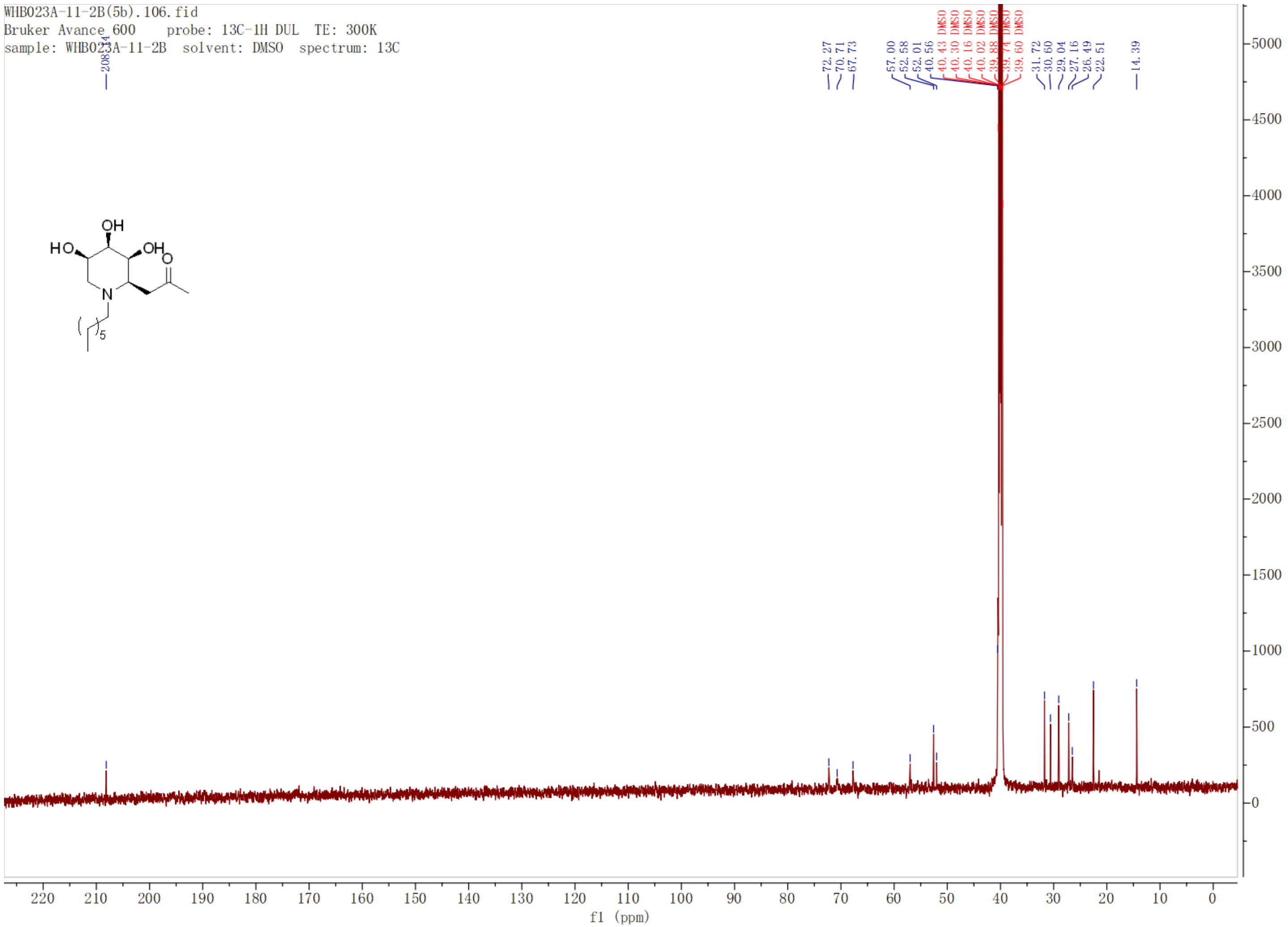

Figure S11. The ^{13}C NMR of compound **5c**.

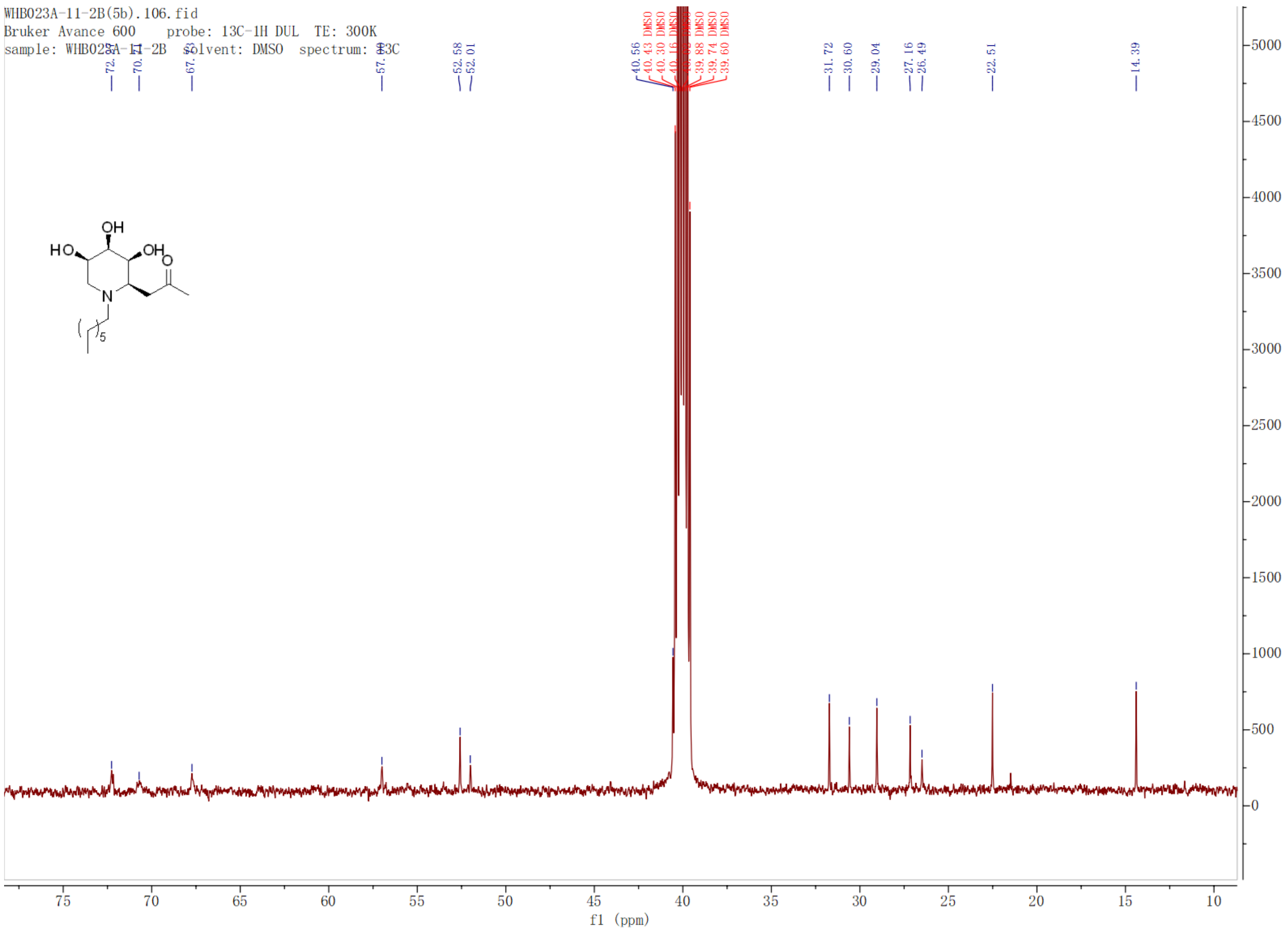

Figure S12. Partial region amplification of the ^{13}C NMR of compound **5c**.

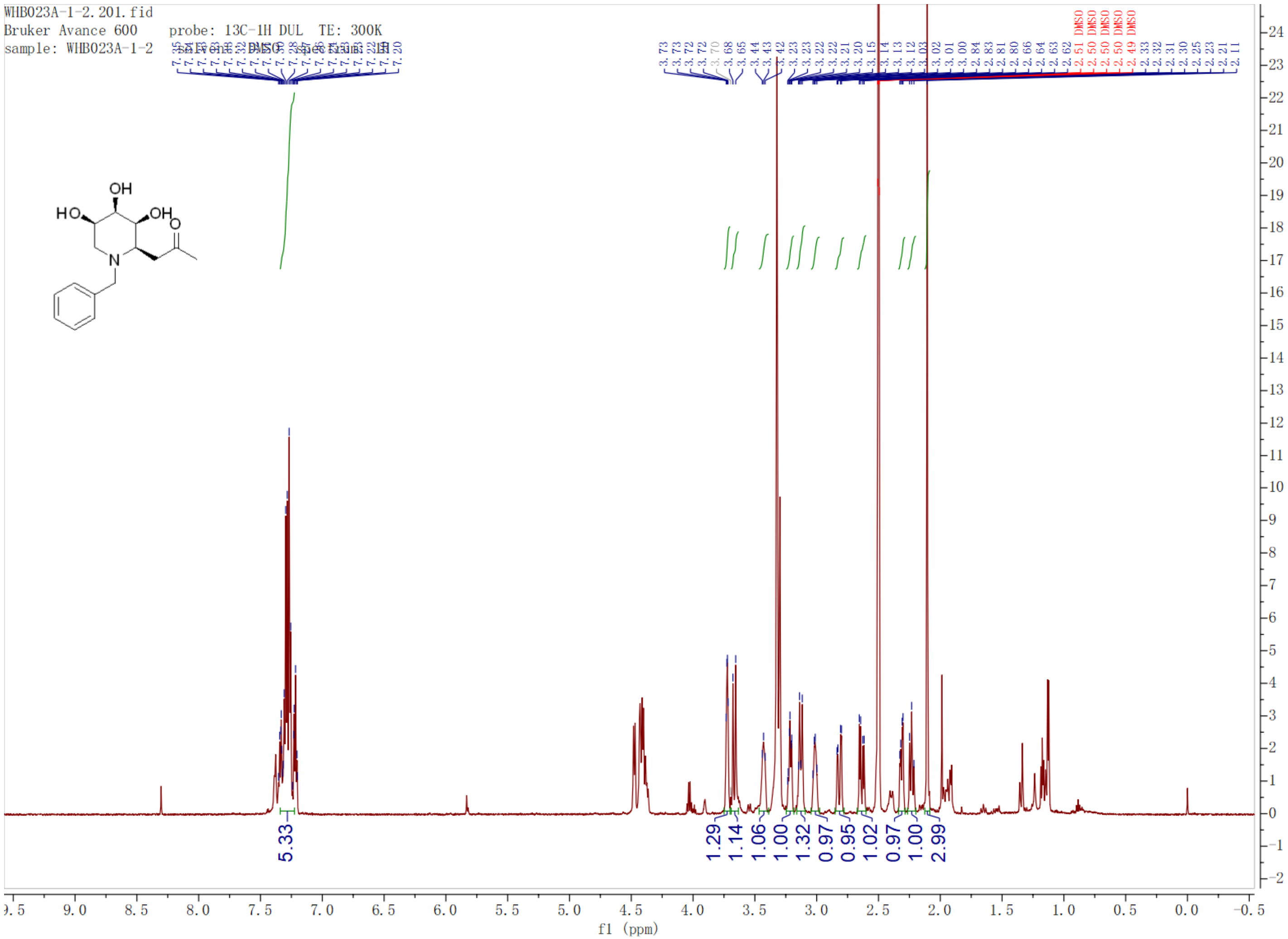

Figure S13. The ^{1}H NMR of compound **5d**.

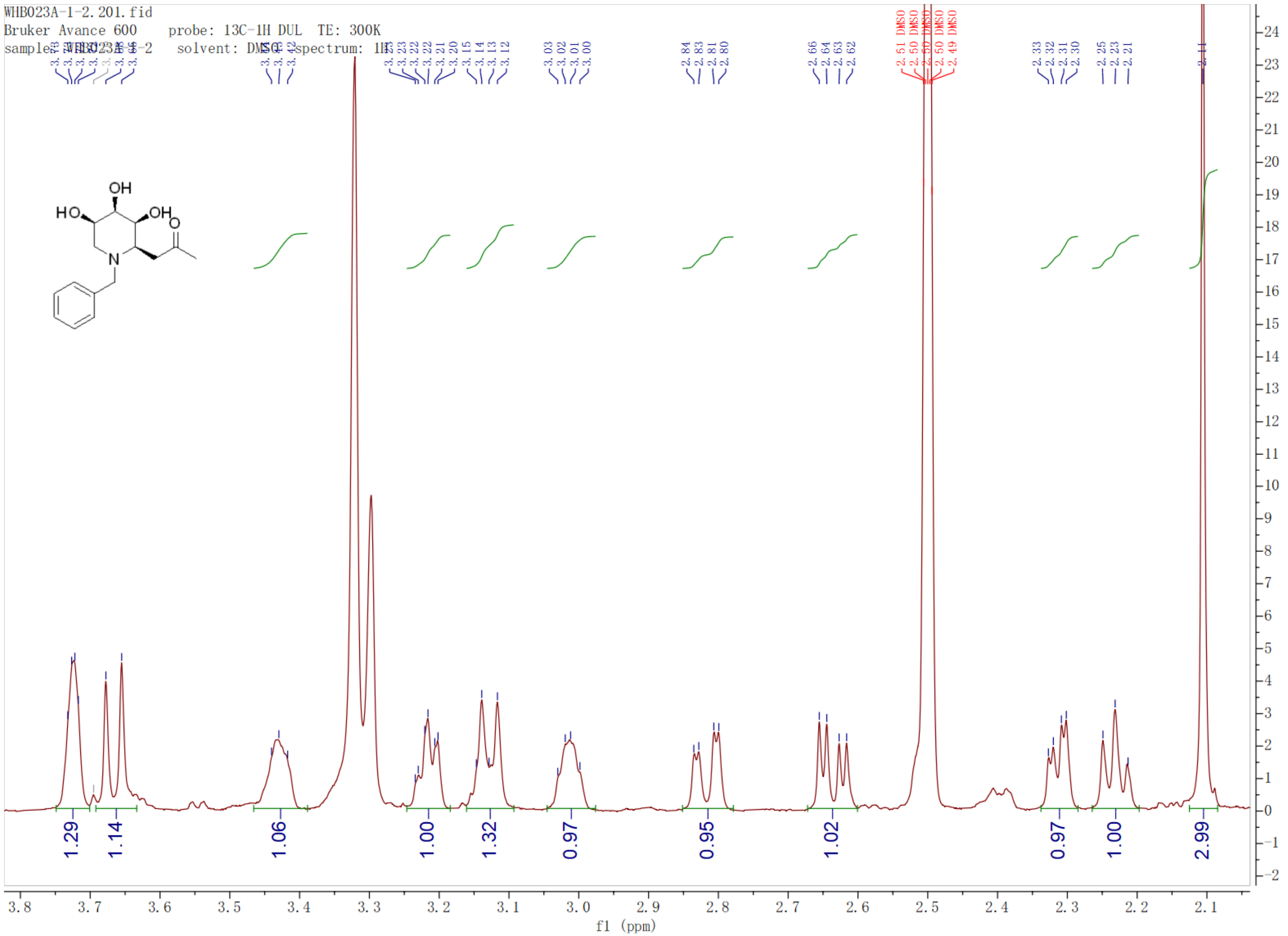

Figure S14. Partial region amplification of the ^{1}H NMR of compound **5d**.

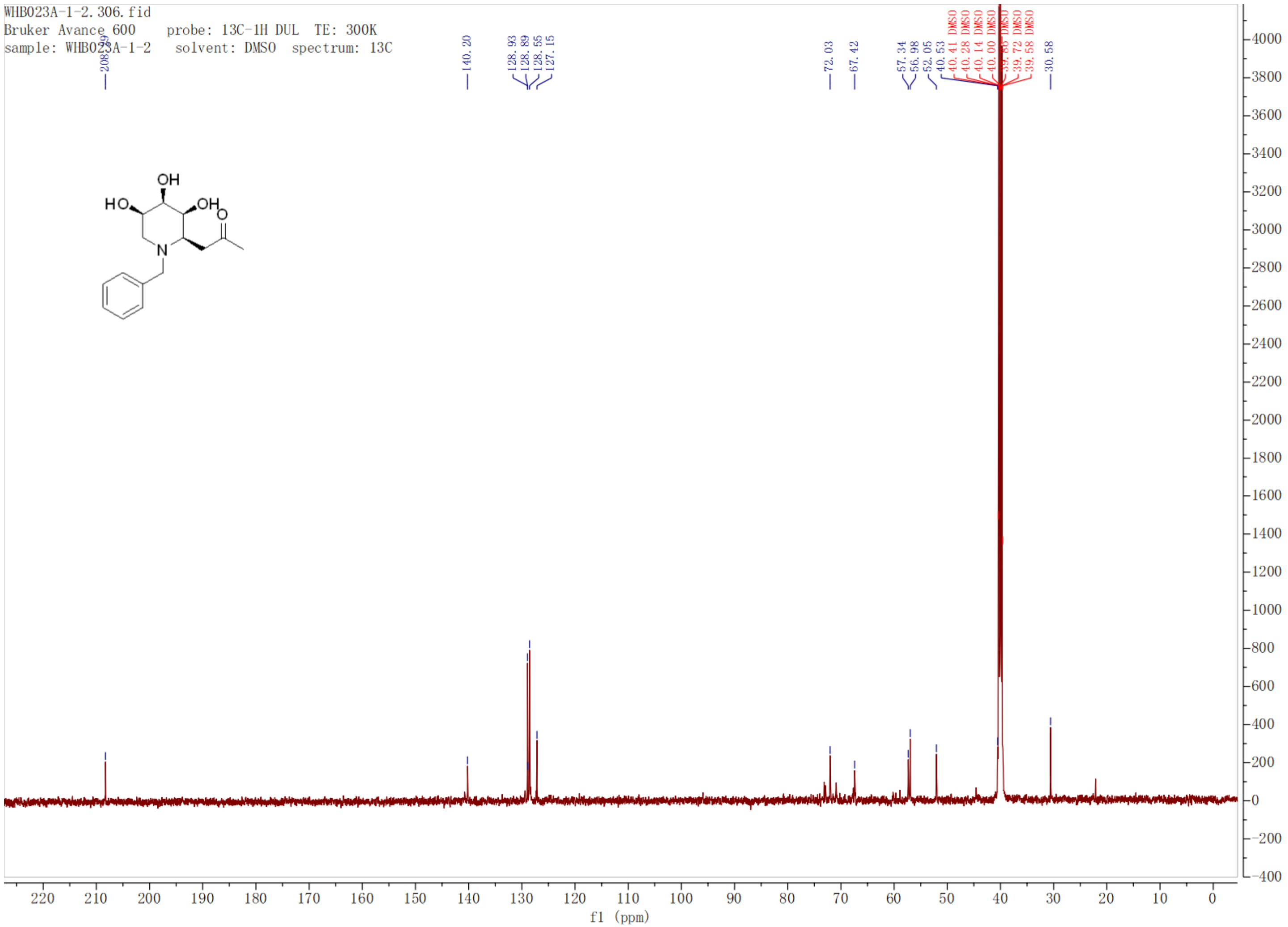

Figure S15. The ^{13}C NMR of compound **5d**.

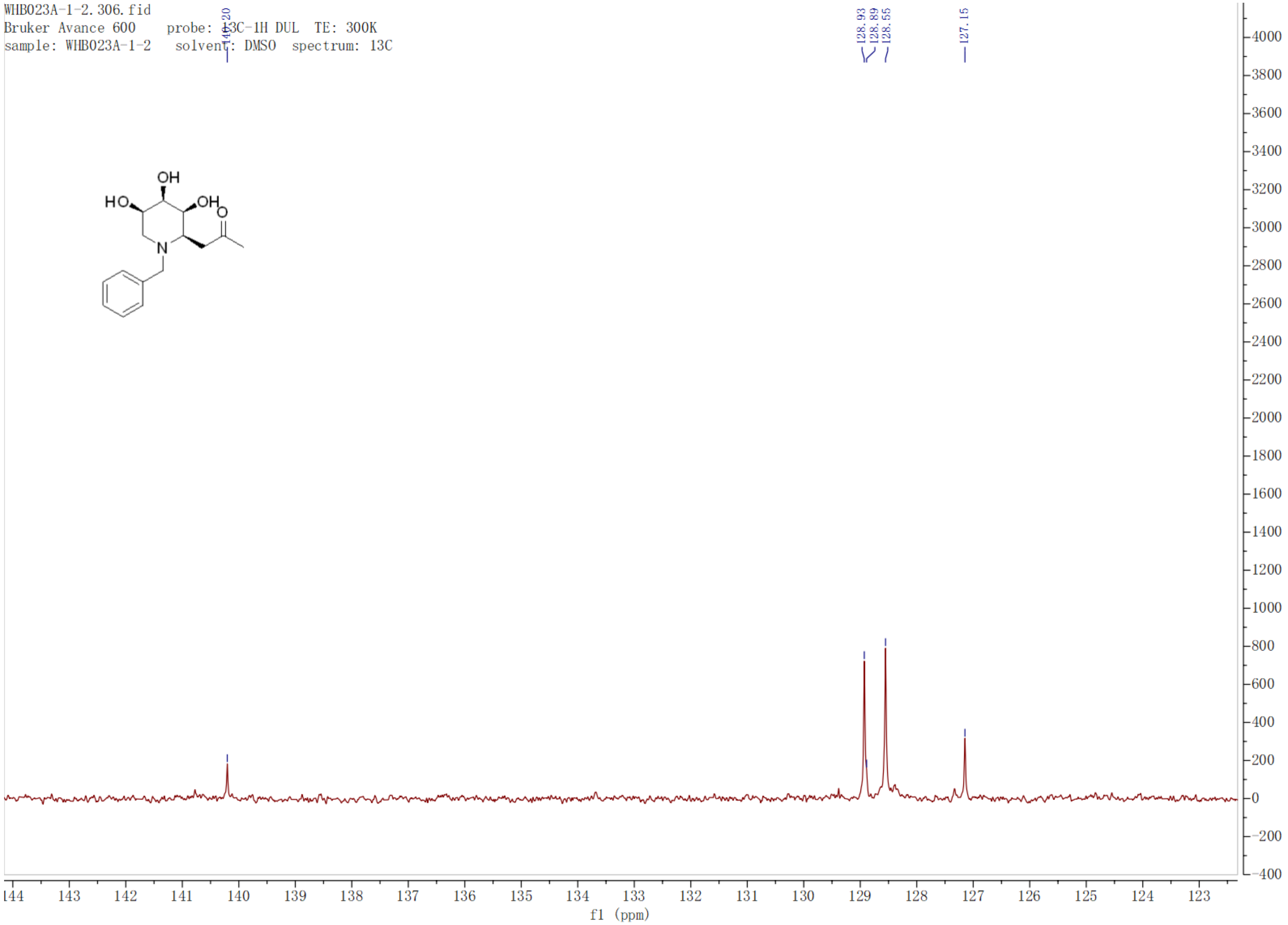

Figure S16. Partial region amplification of the ^{13}C NMR of compound **5d**.

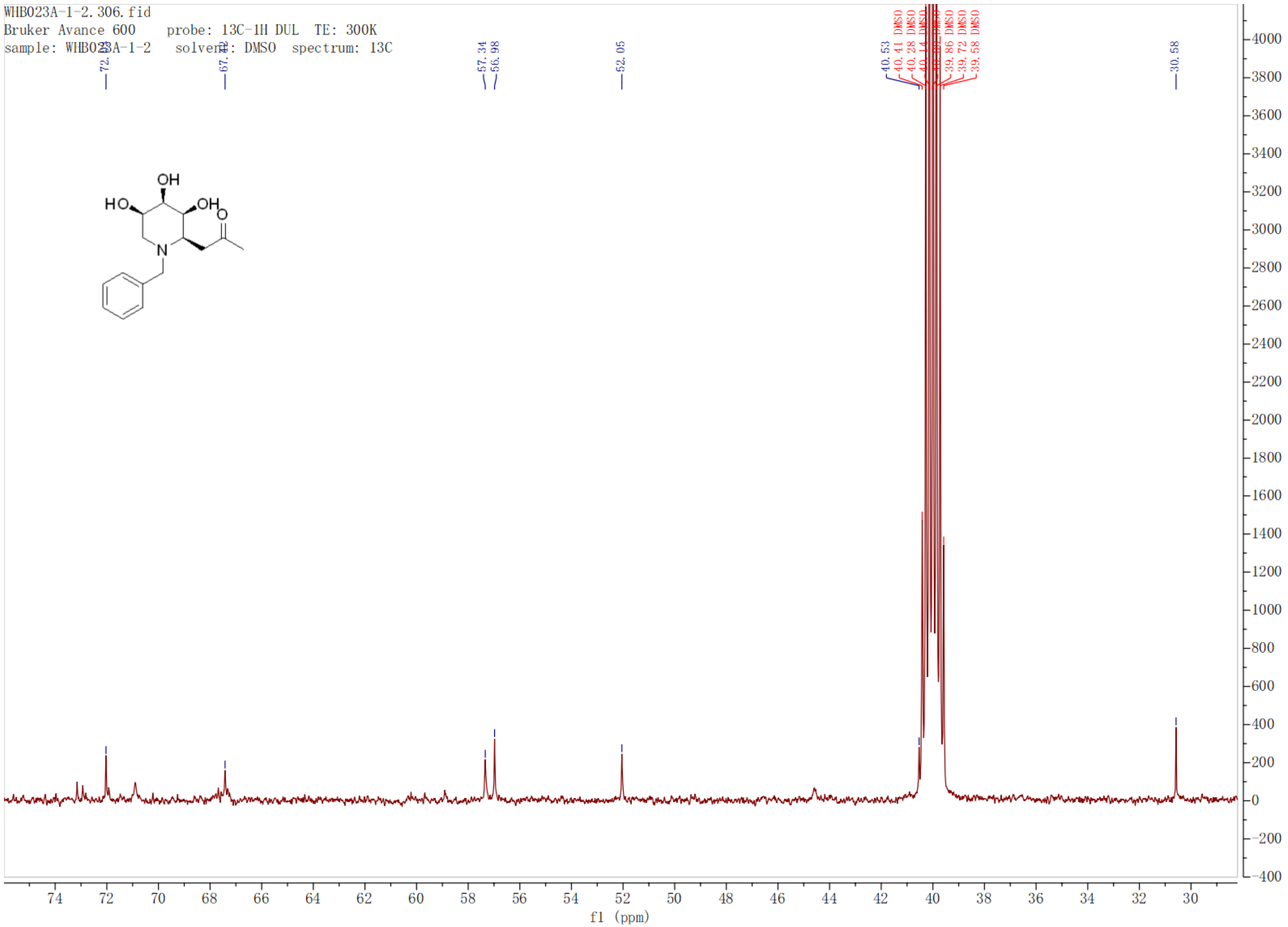

Figure S17. Partial region amplification of the ^{13}C NMR of compound **5d**.

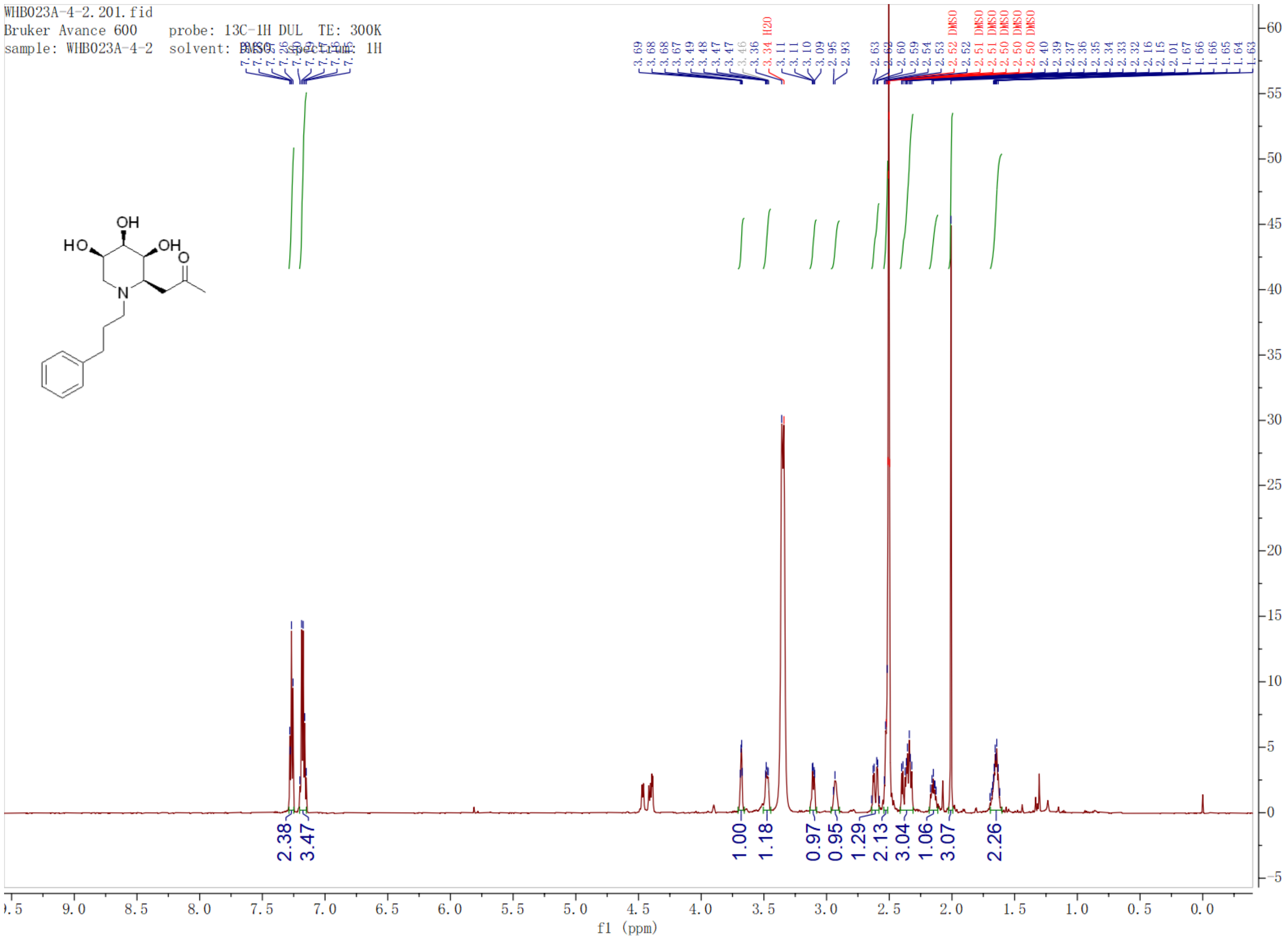

Figure S18. The ^{1}H NMR of compound **5e**.

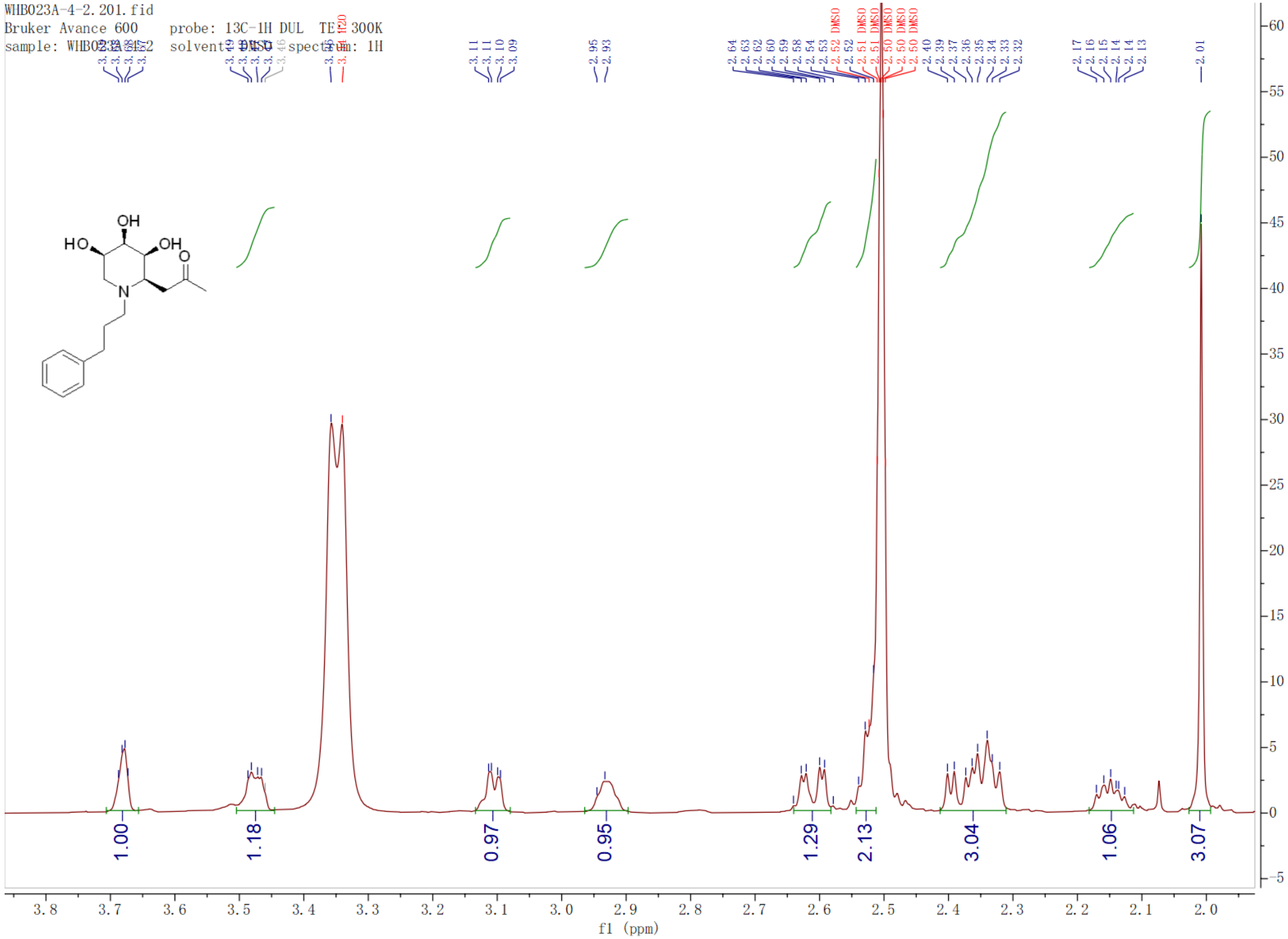

Figure S19. Partial region amplification of the ^{1}H NMR of compound **5e**.

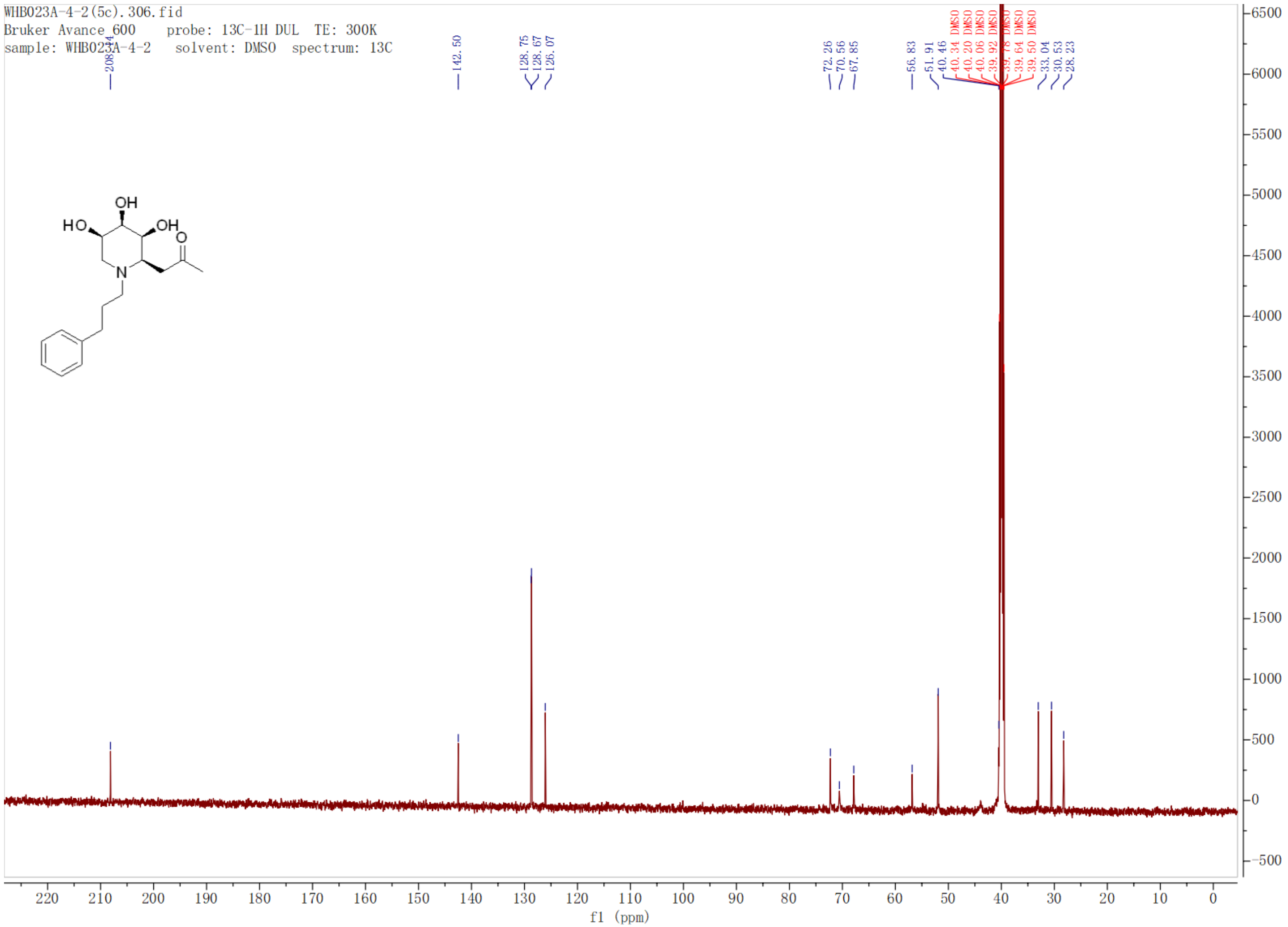

Figure S20. The ^{13}C NMR of compound **5e**.

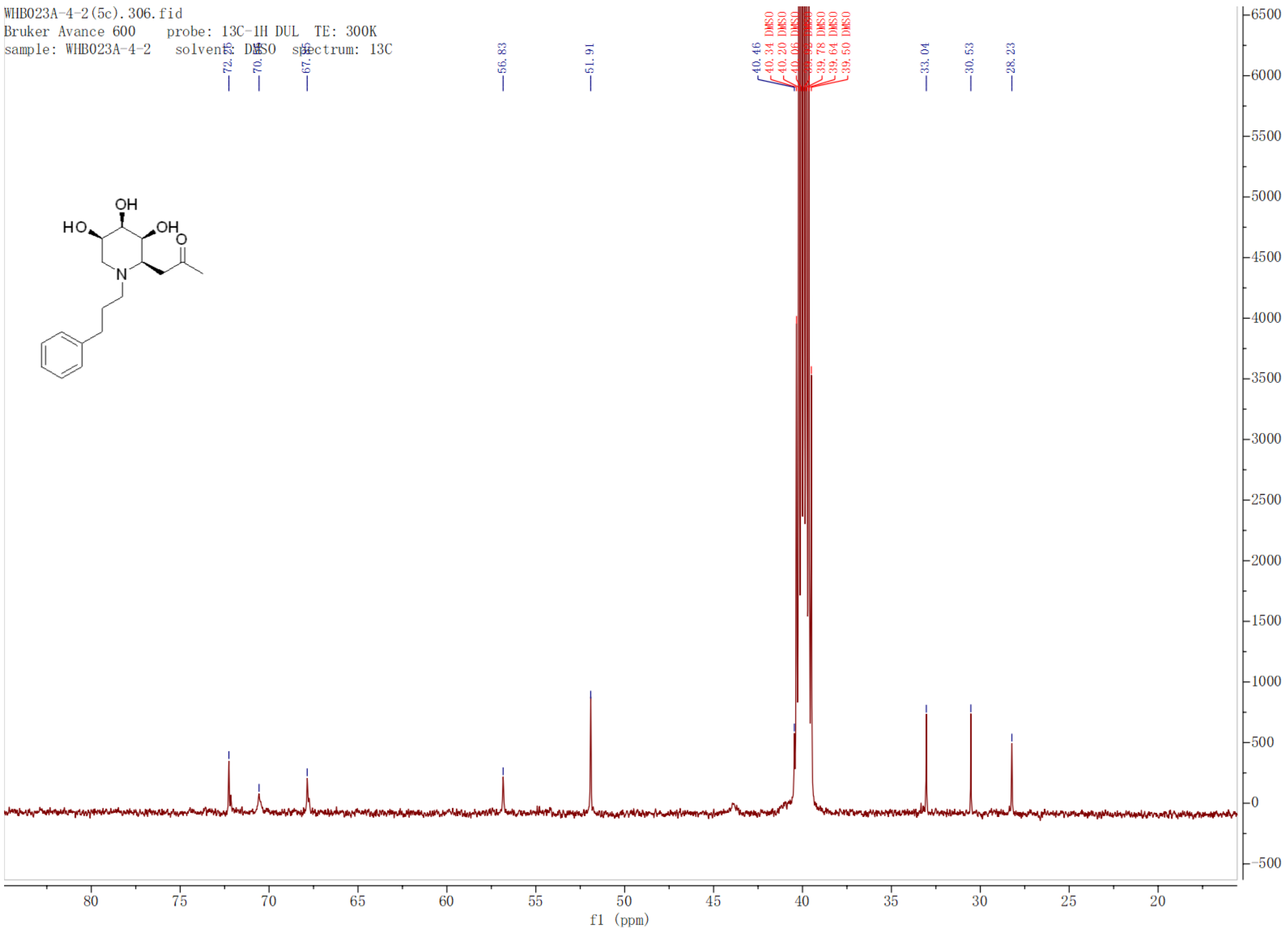

Figure S21. Partial region amplification of the ^{13}C NMR of compound **5e**.

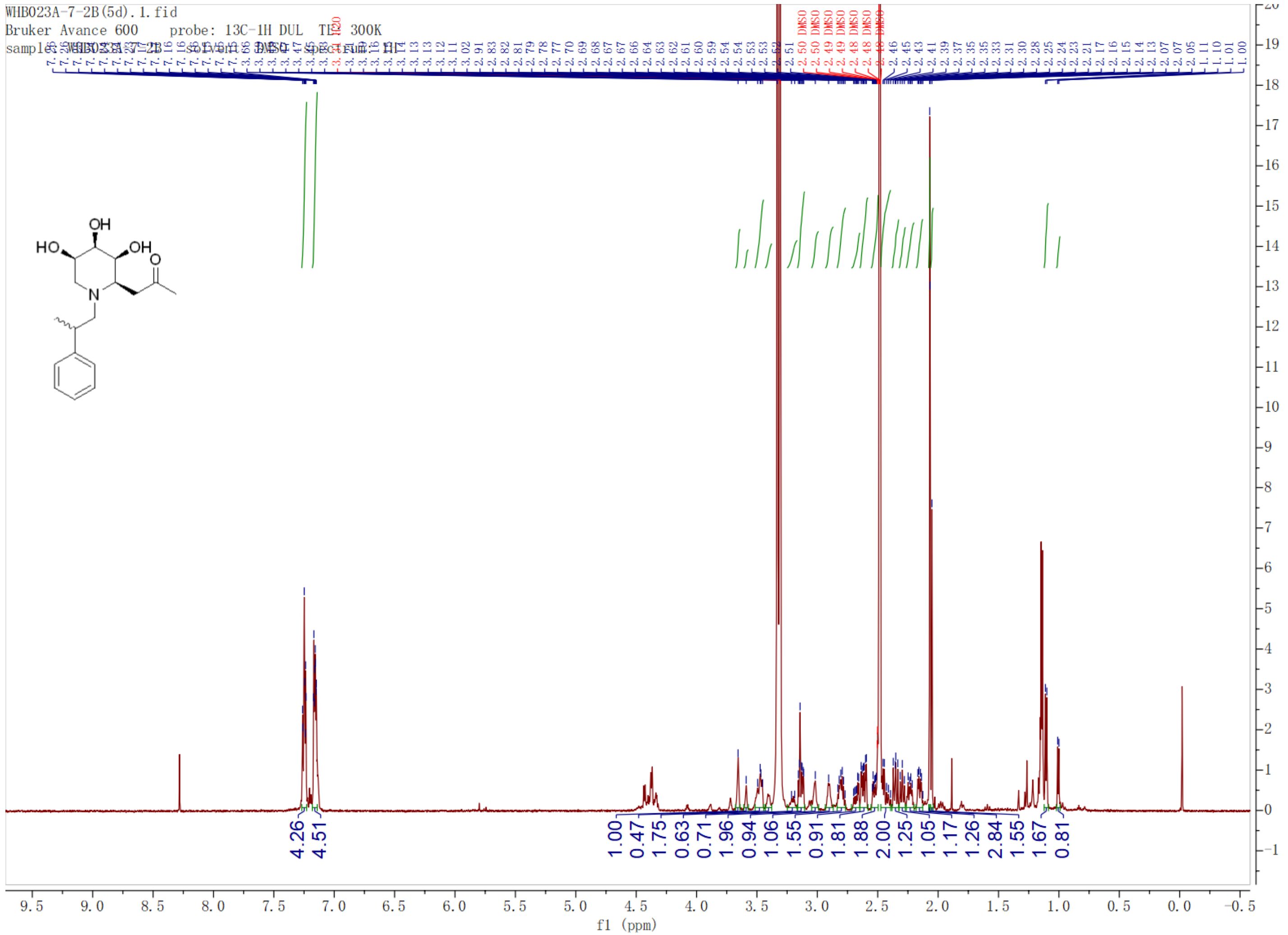

Figure S22. The ^{1}H NMR of compound **5f**.

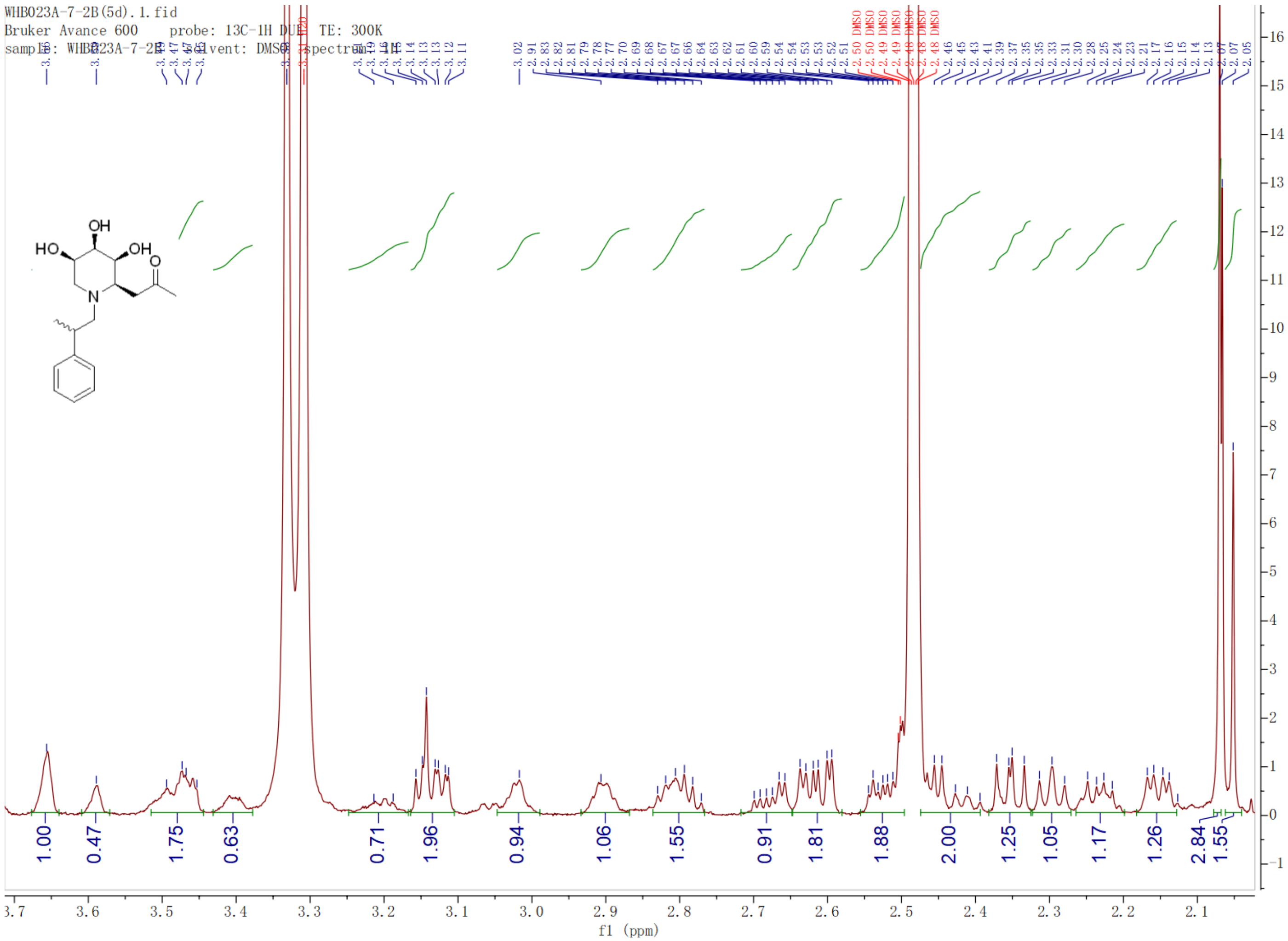

Figure S23. Partial region amplification of the ^1H NMR of compound **5f**.

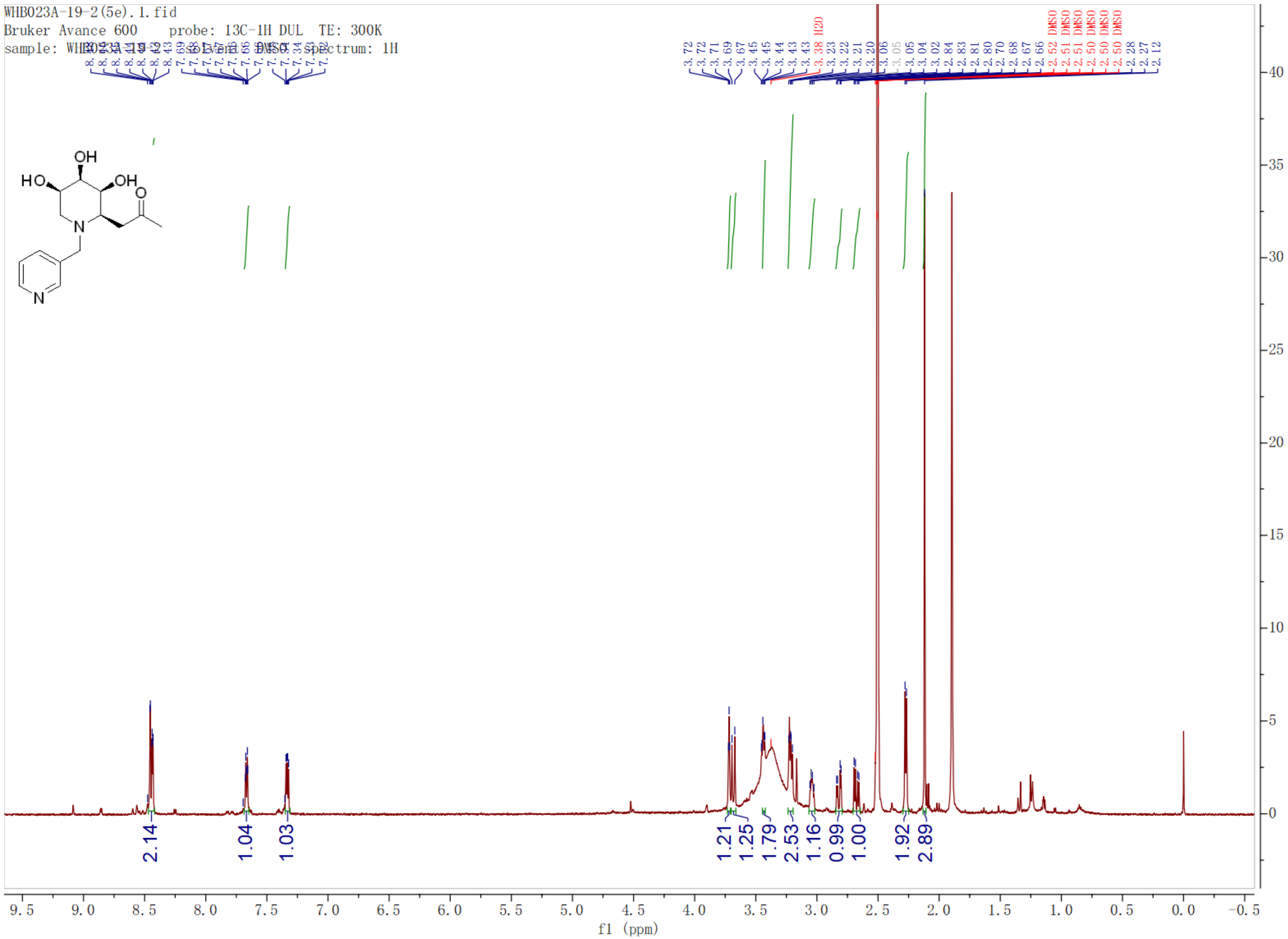

Figure S24. The ^{1}H NMR of compound **5g**.

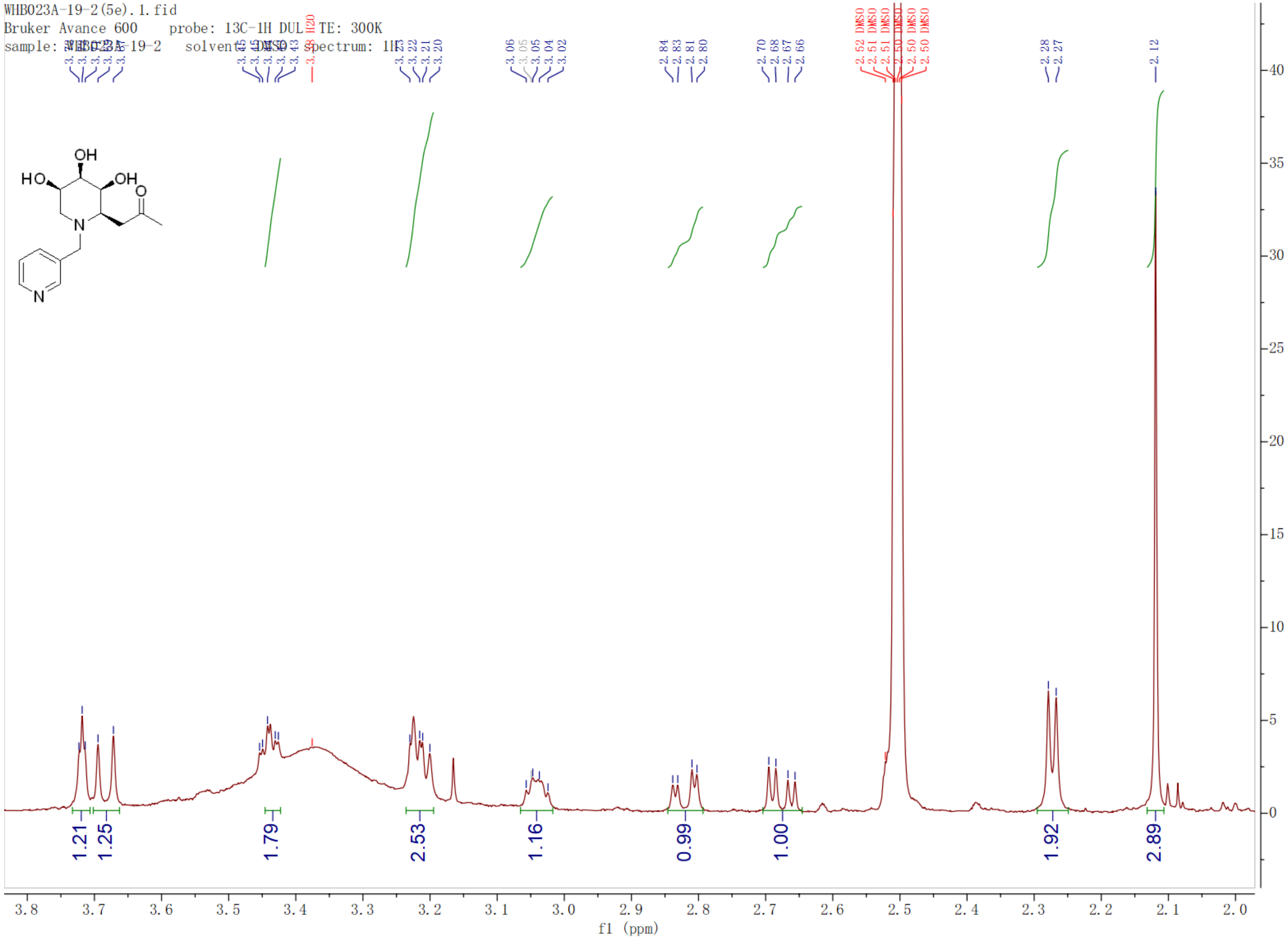

Figure S25. Partial region amplification of the ^{1}H NMR of compound **5g**.

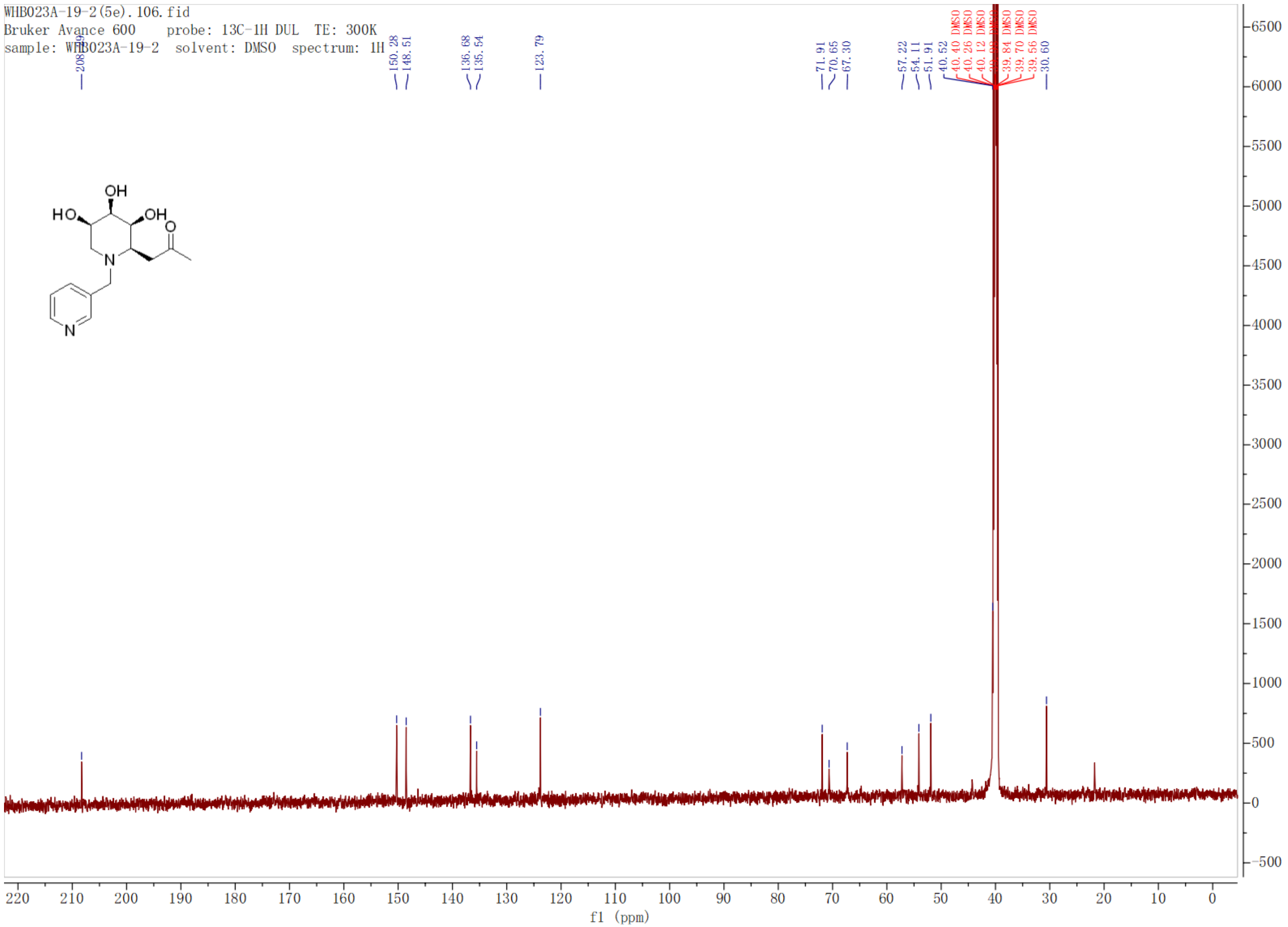

Figure S26. The ^{13}C NMR of compound **5g**.

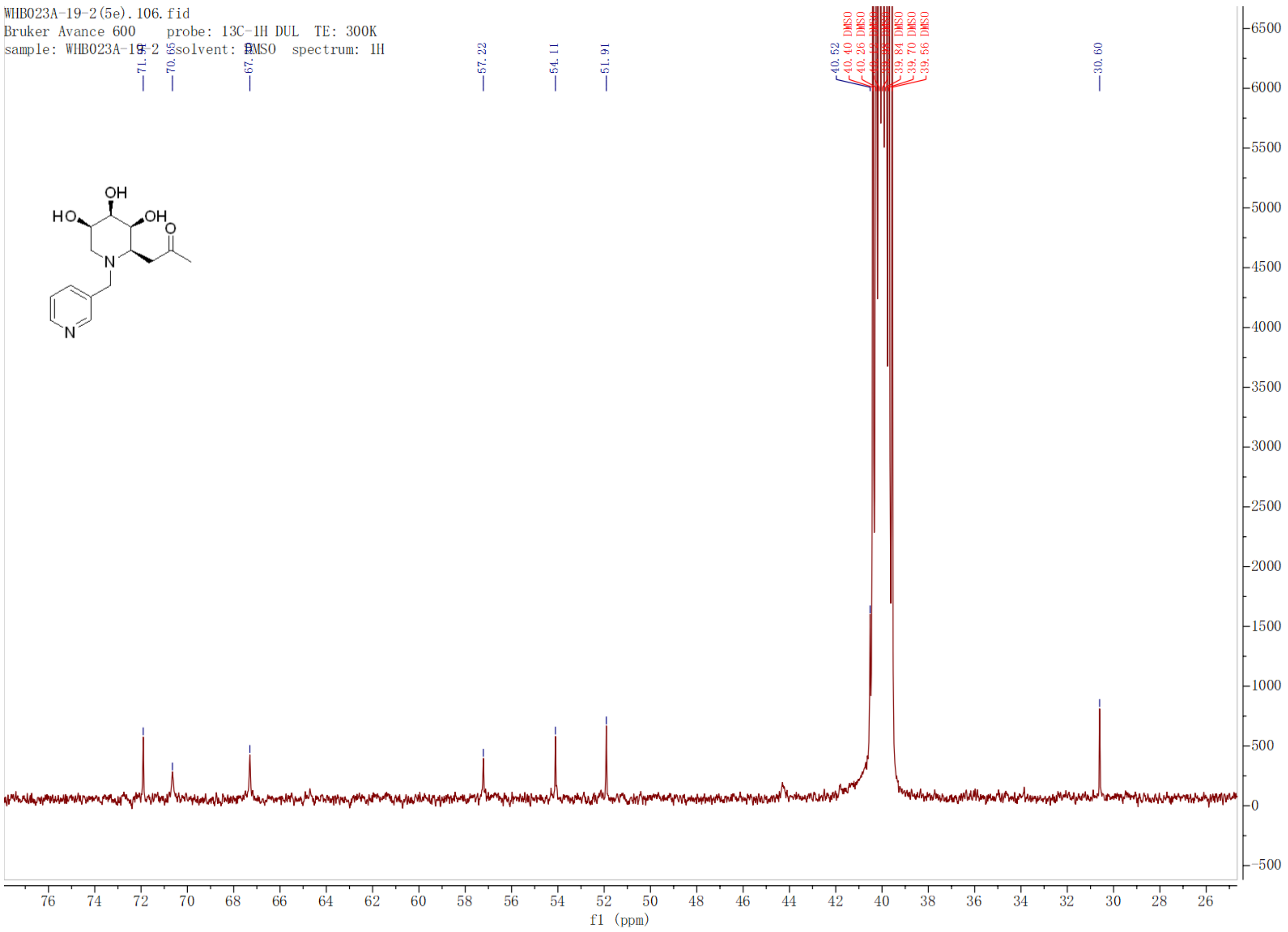

Figure S27. Partial region amplification of the ^{13}C NMR of compound **5g**.

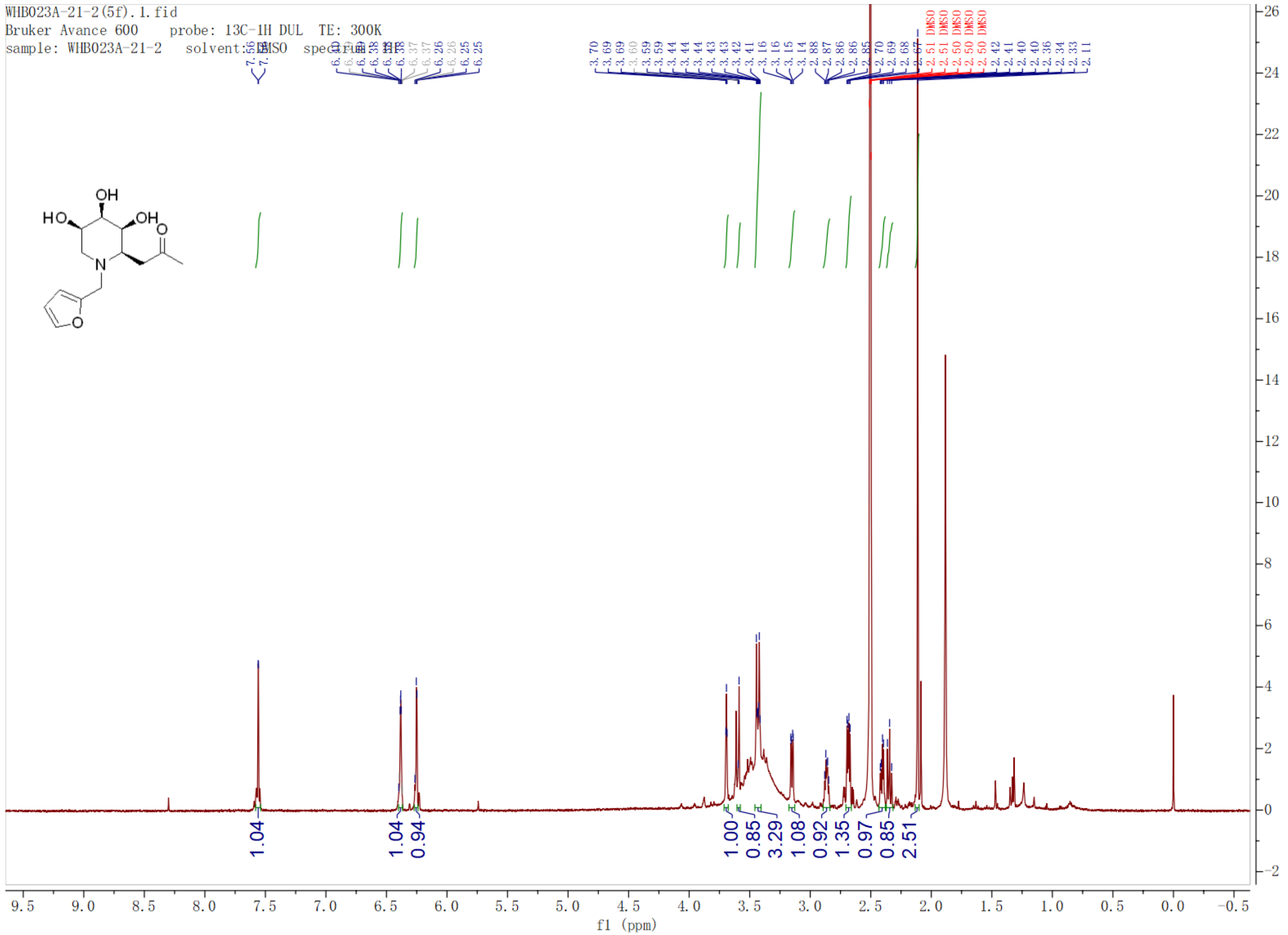

Figure S28. The ^{1}H NMR of compound **5h**.

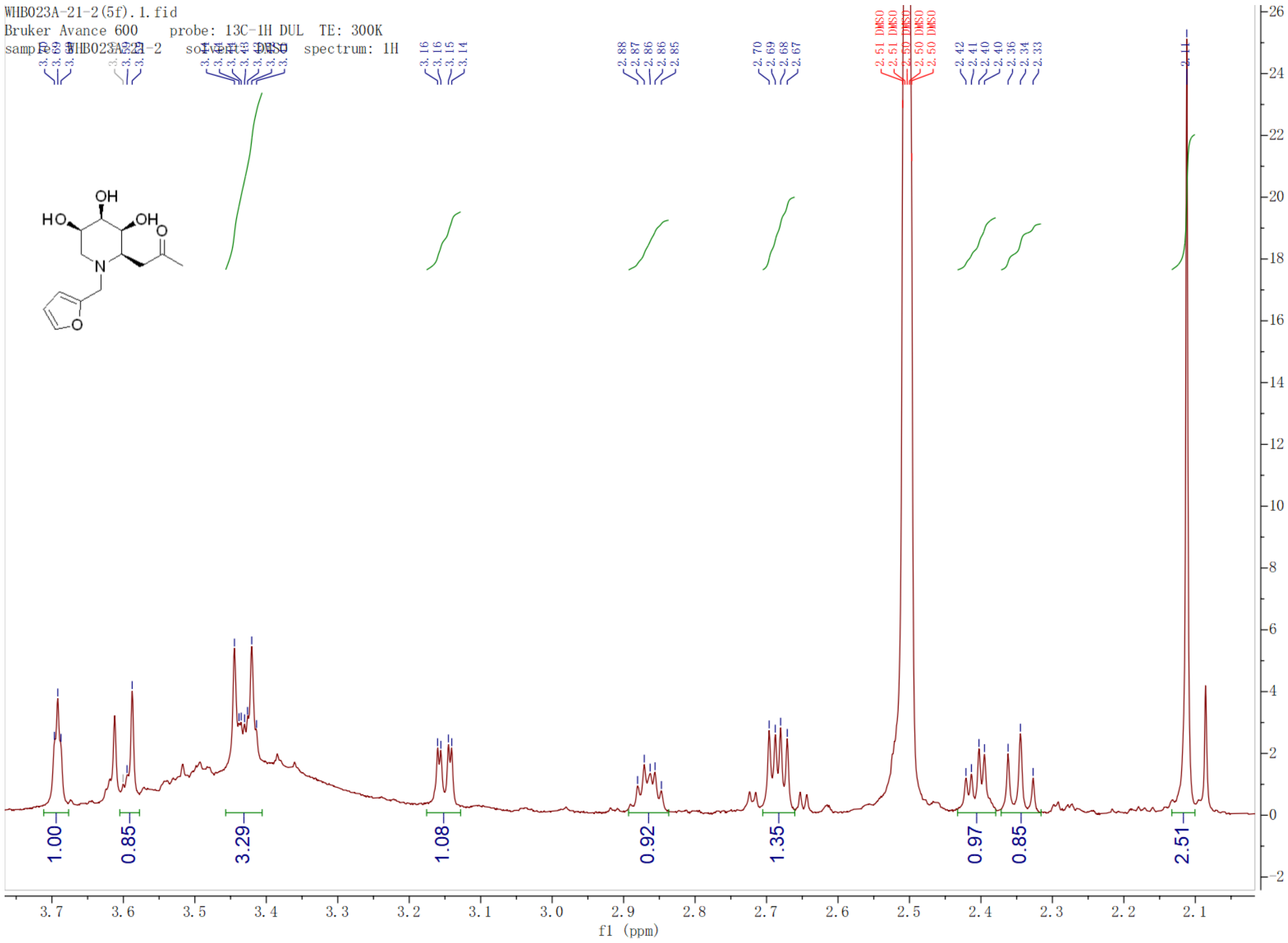

Figure S29. Partial region amplification of the ^{1}H NMR of compound **5h**.

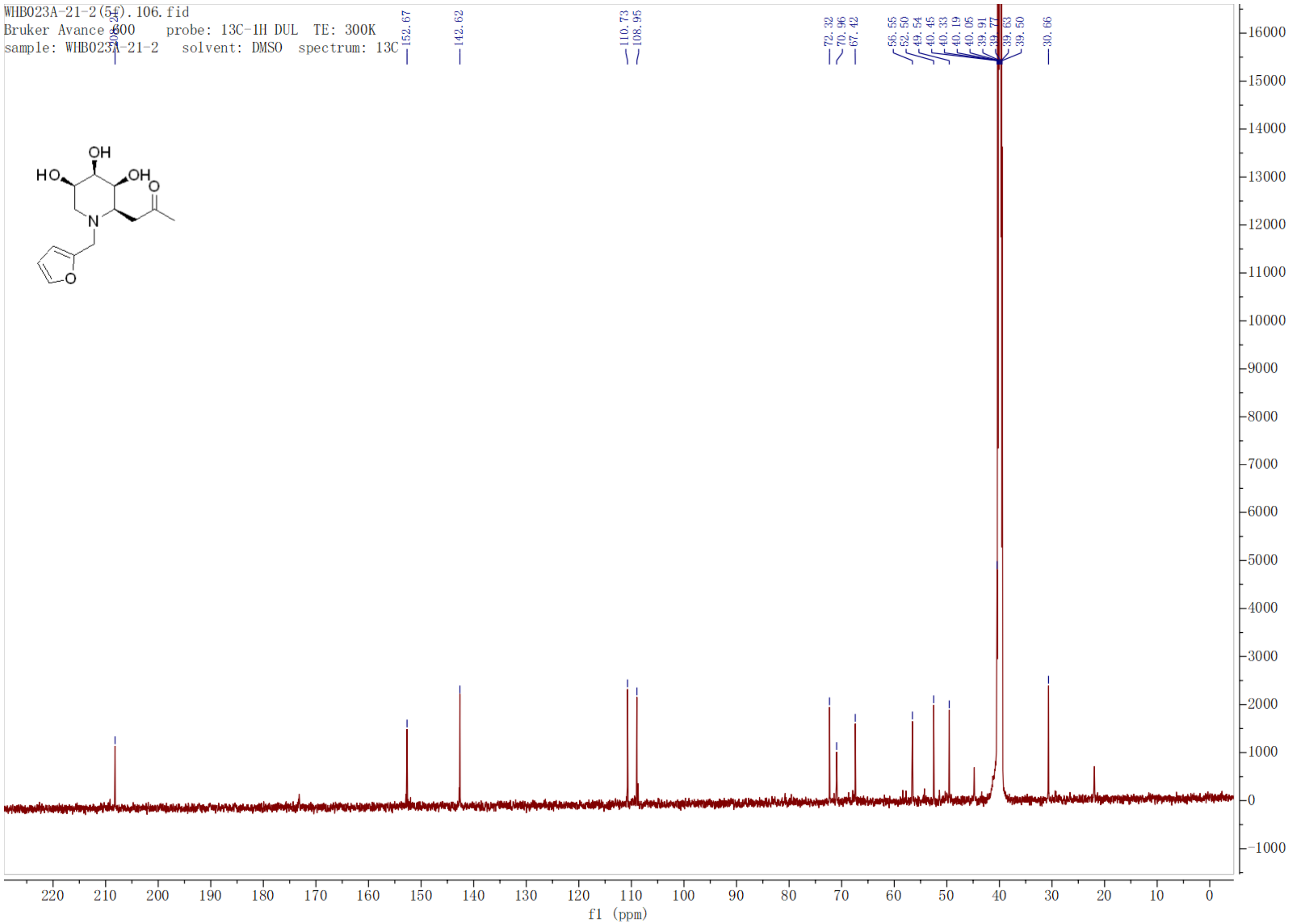

Figure S30. The ^{13}C NMR of compound **5h**.

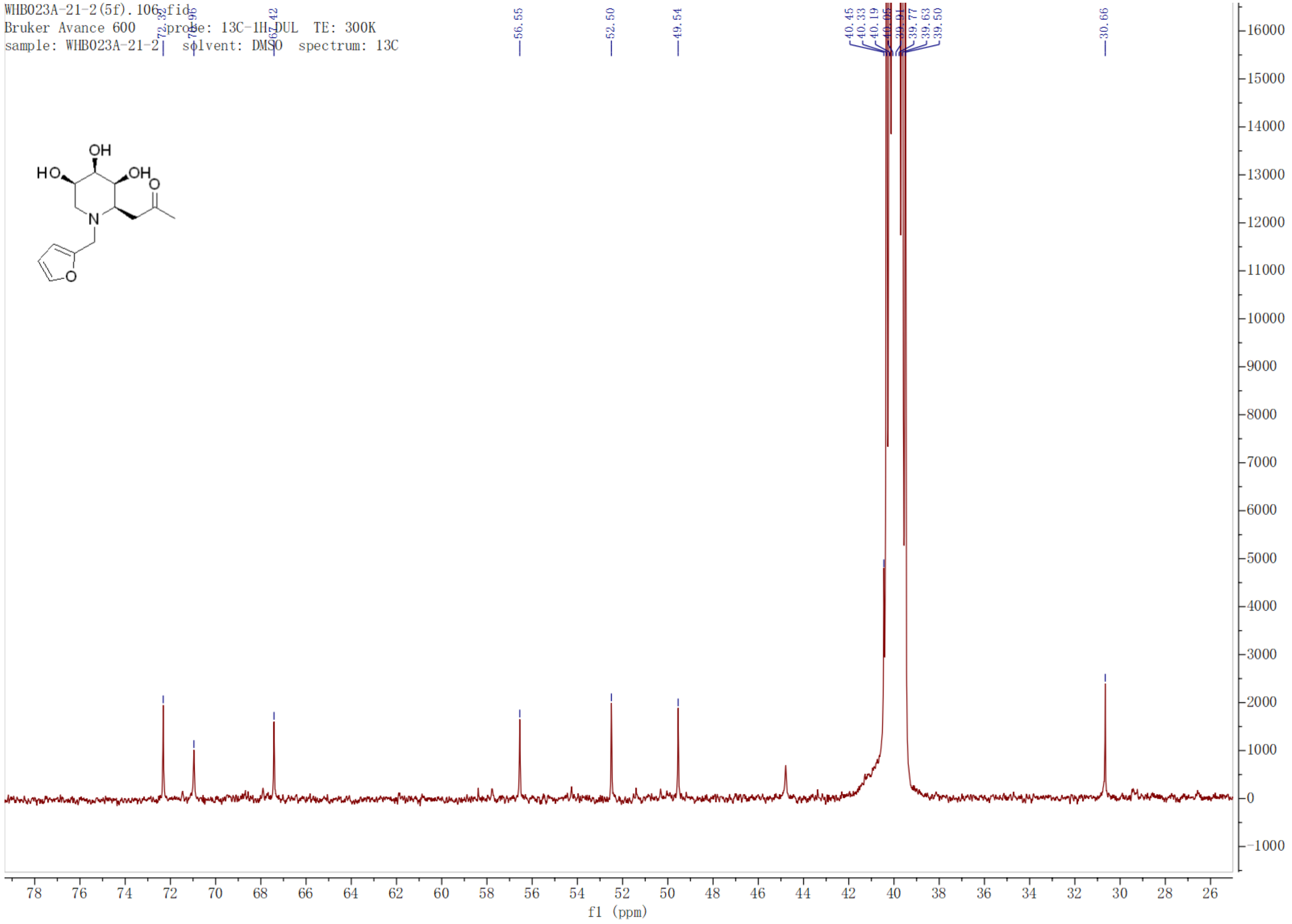

Figure S31. Partial region amplification of the ^{13}C NMR of compound **5h**.

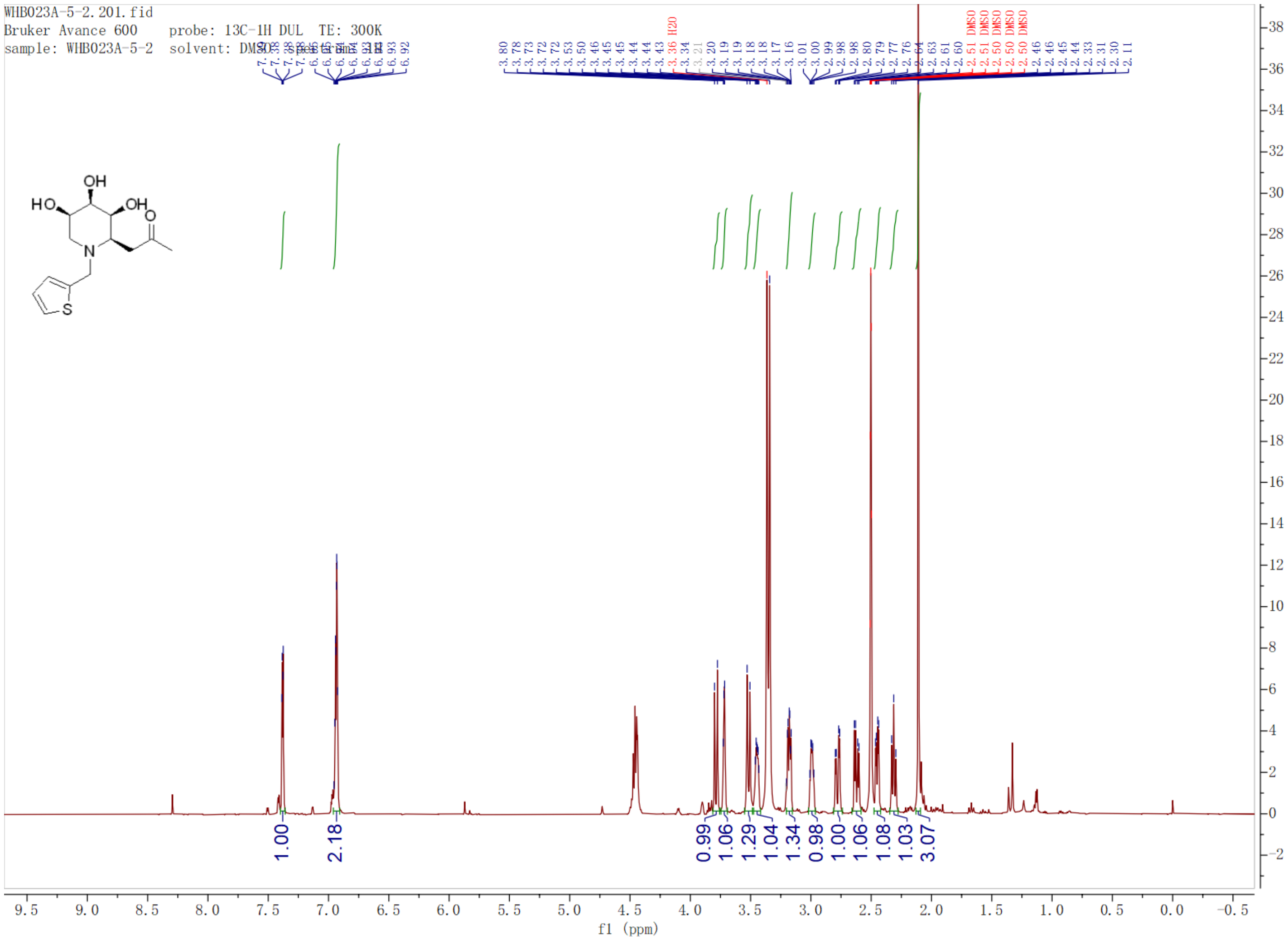

Figure S32. The ^{1}H NMR of compound **5i**.

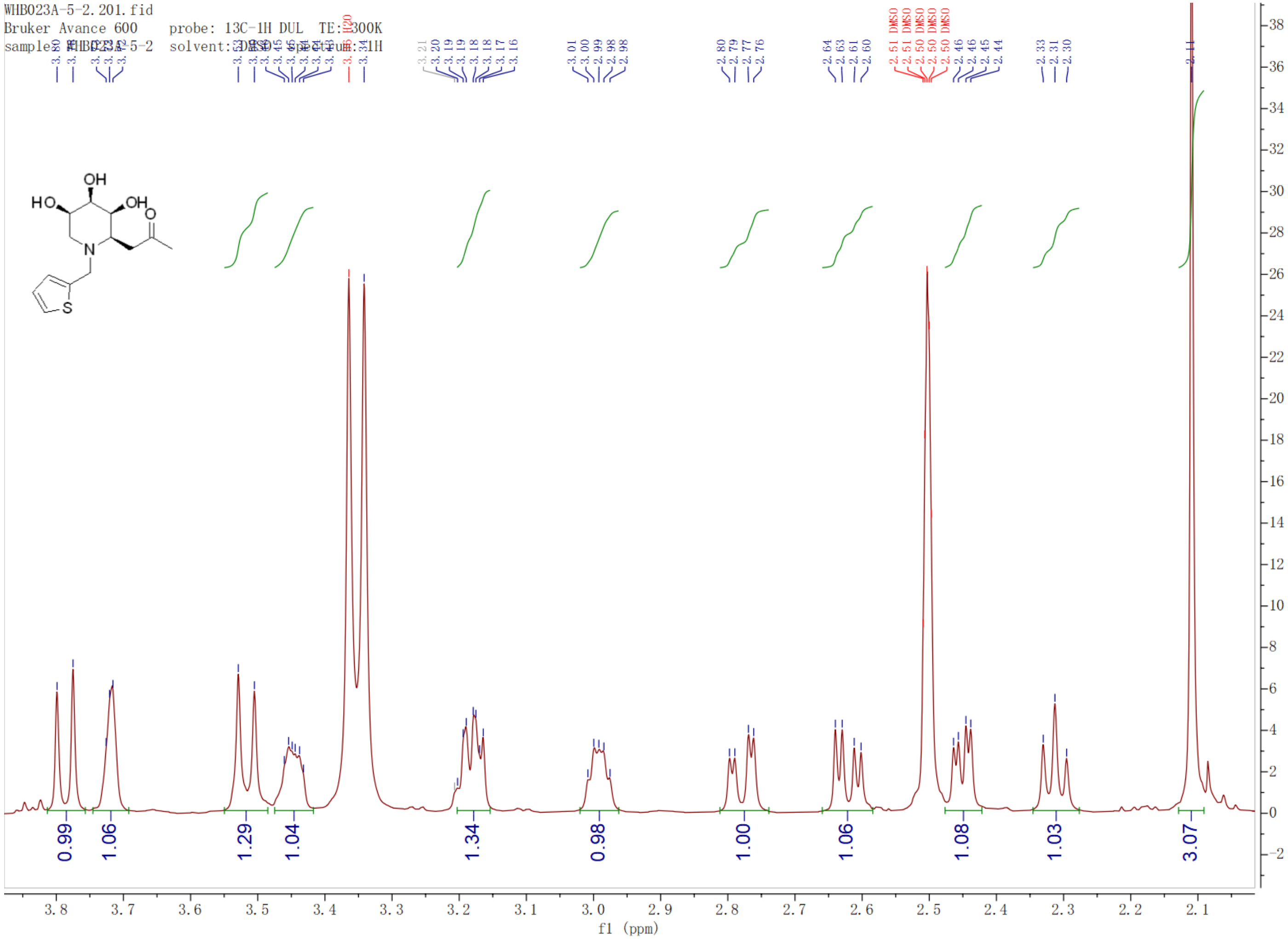

Figure S33. Partial region amplification of the ^1H NMR of compound **5i**.

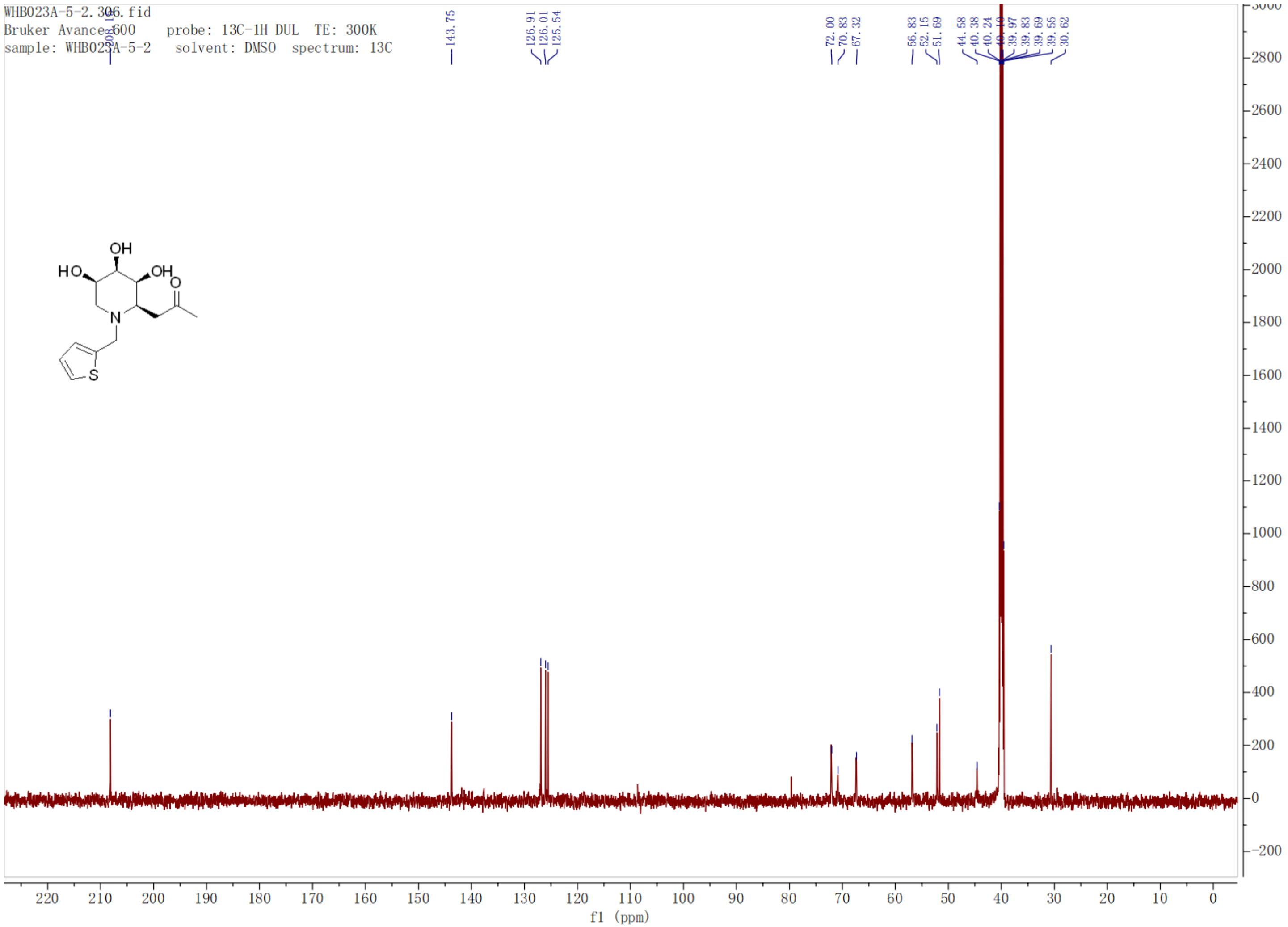

Figure S34. The ^{13}C NMR of compound **5i**.

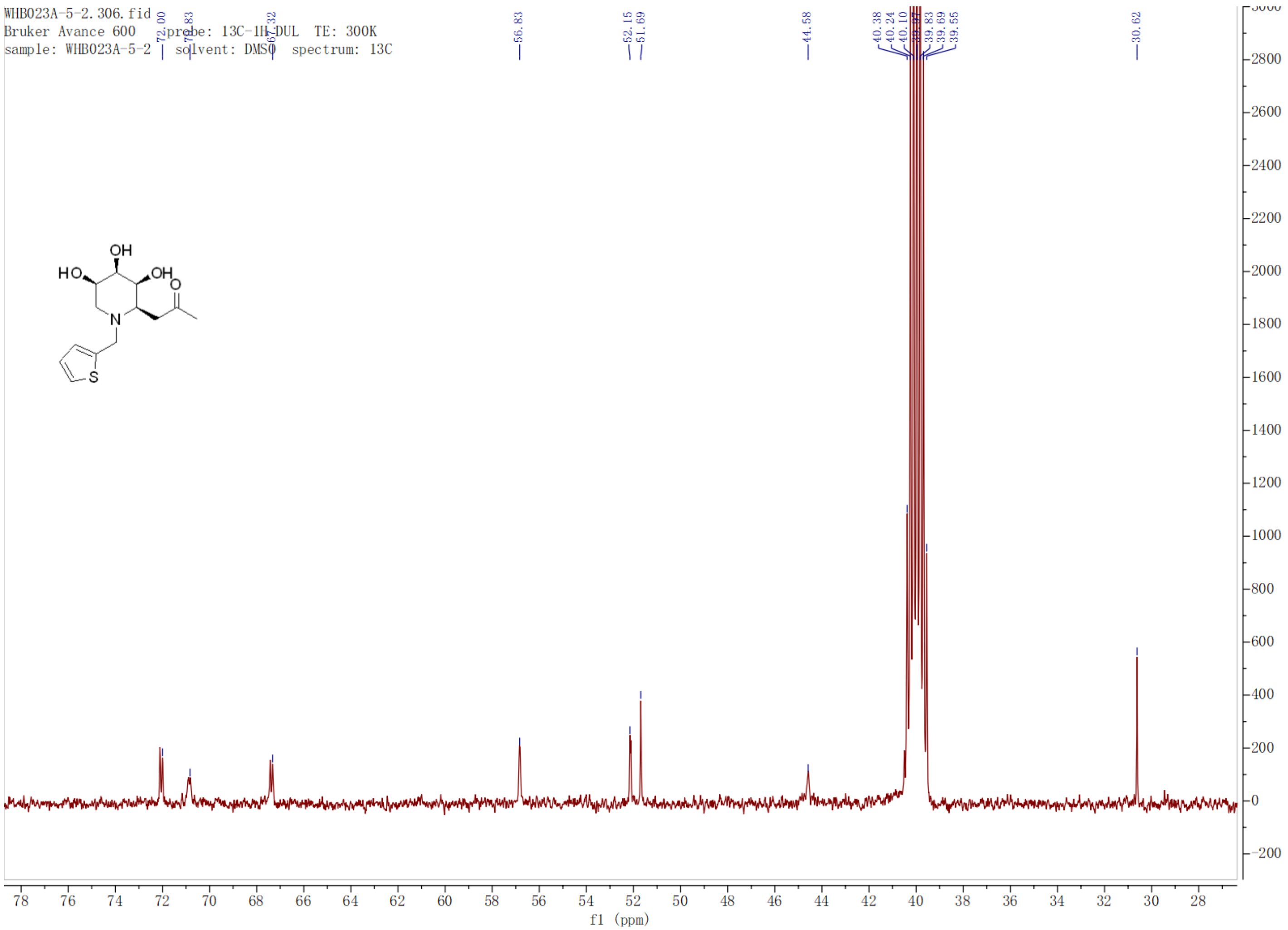

Figure S35. Partial region amplification of the ^{13}C NMR of compound **5i**.

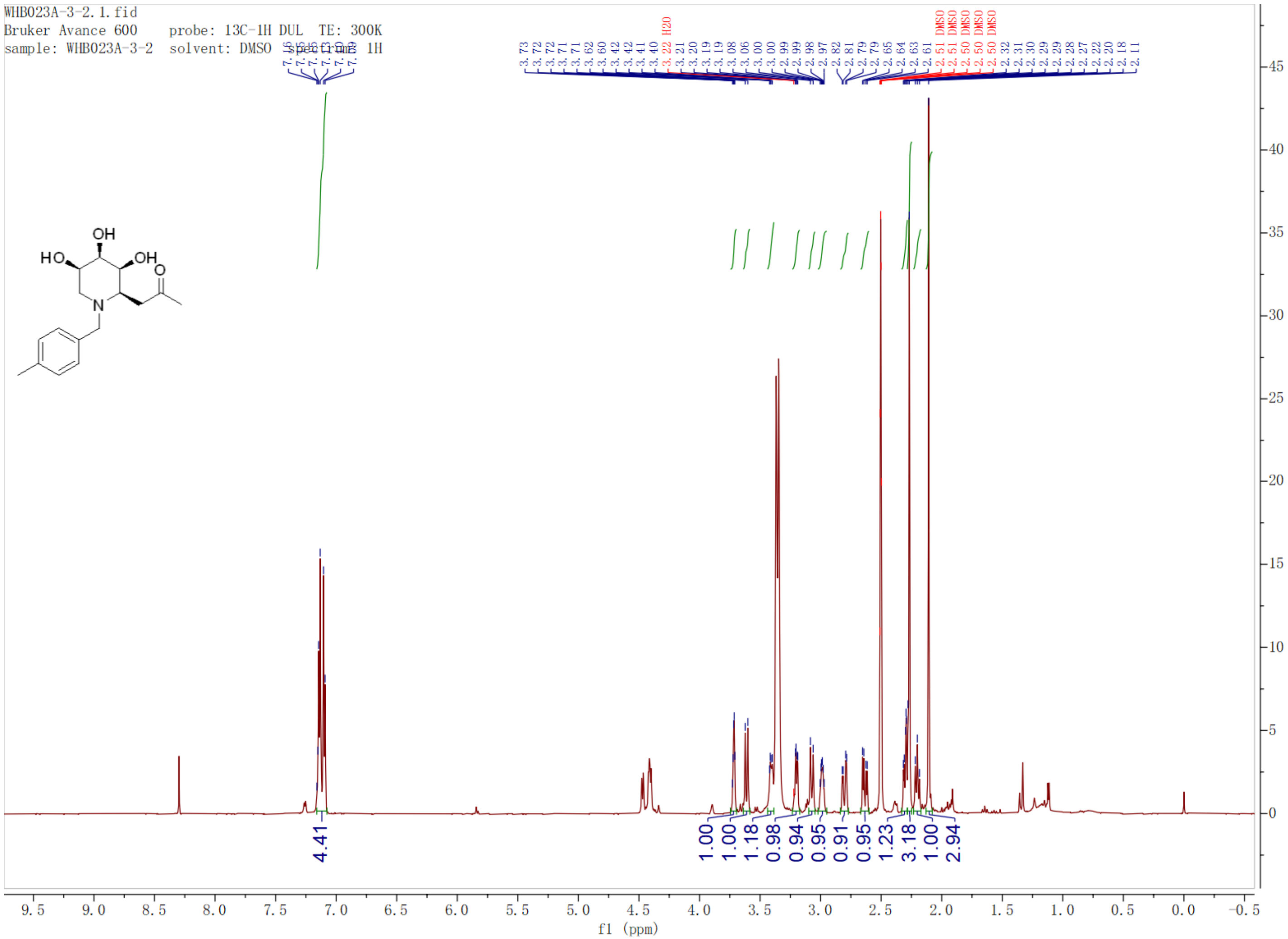

Figure S36. The ^{1}H NMR of compound **5j**.

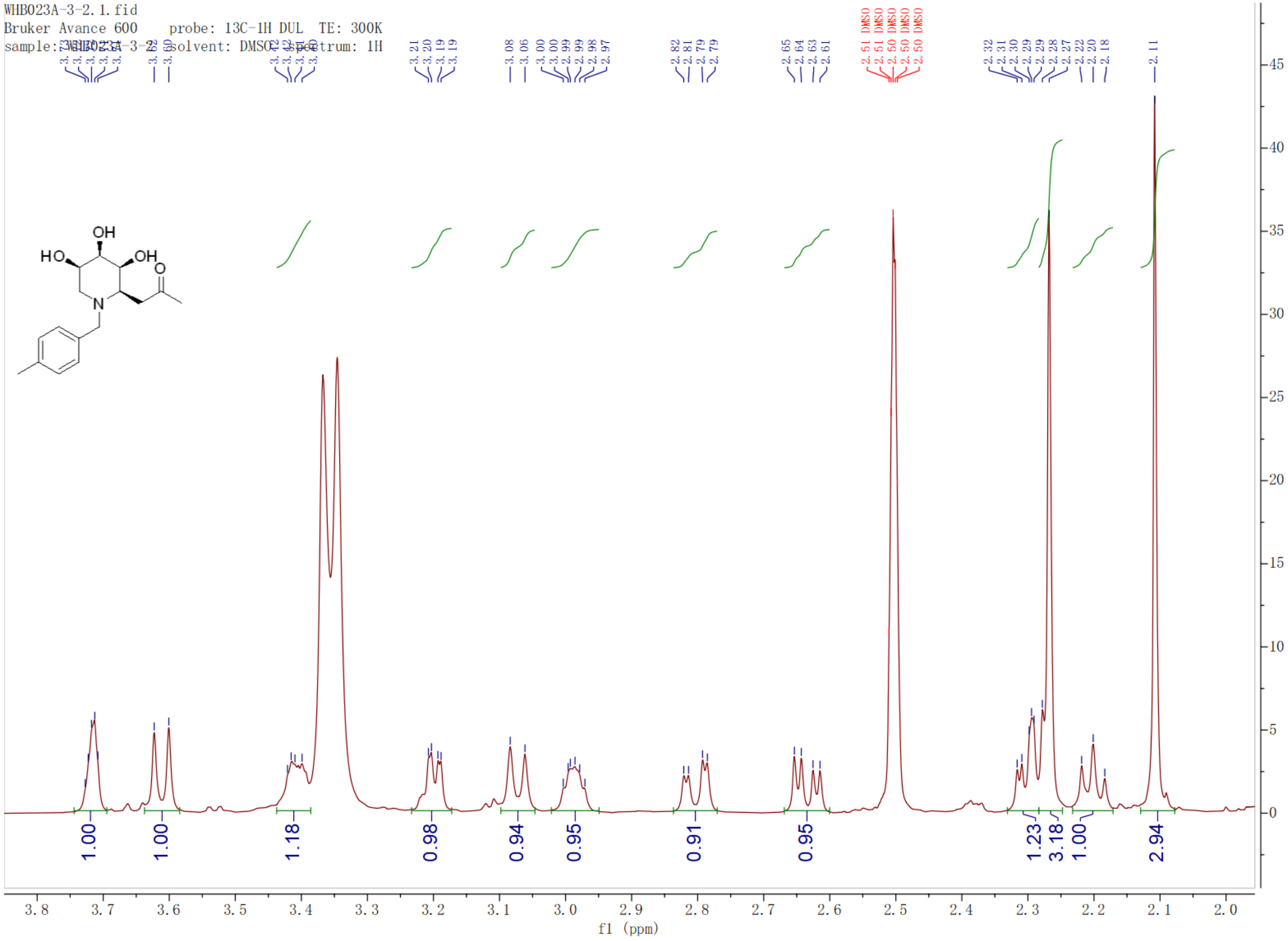

Figure S37. Partial region amplification of the ^{1}H NMR of compound **5j**.

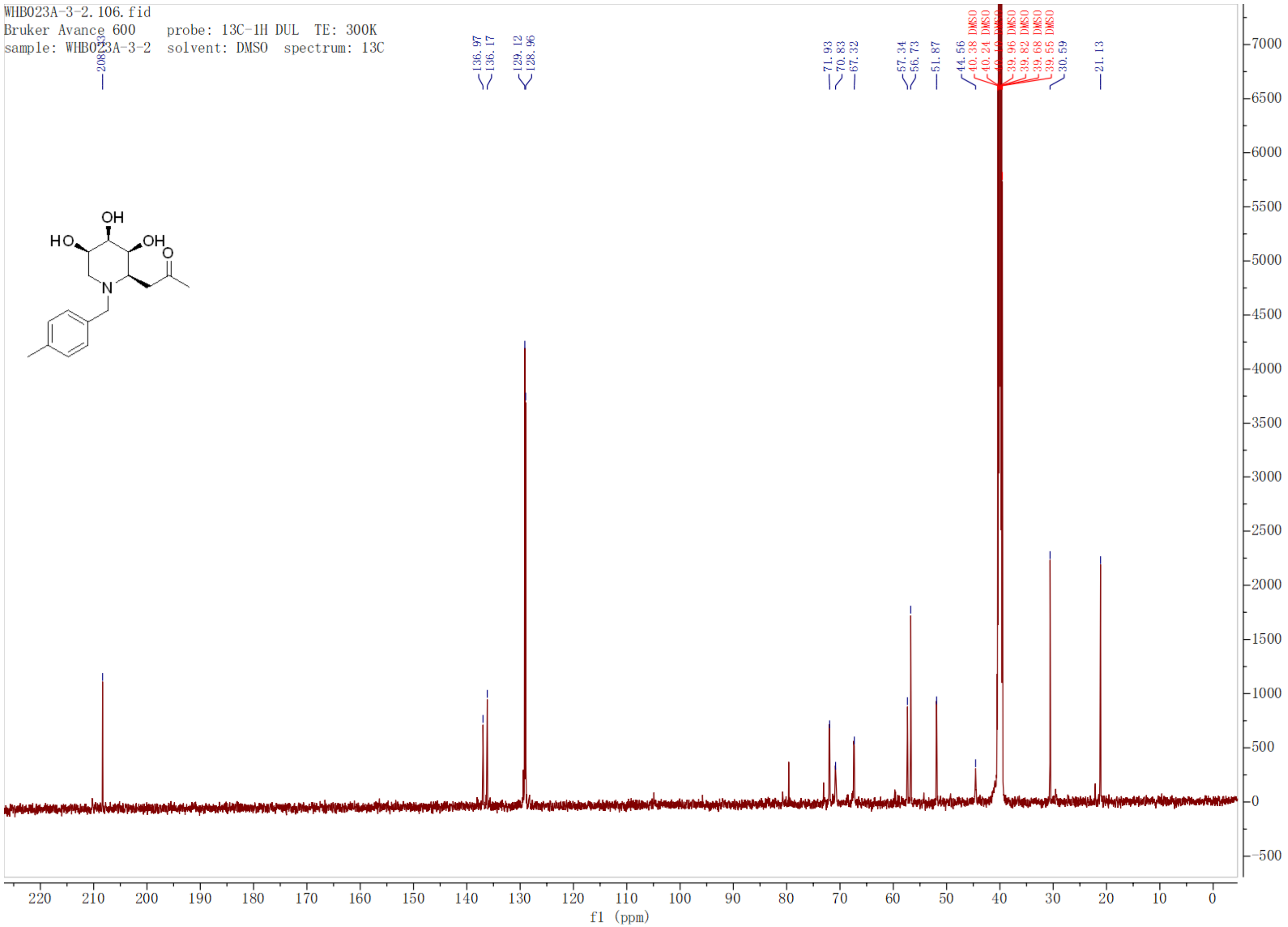

Figure S38. The ^{13}C NMR of compound **5j**.

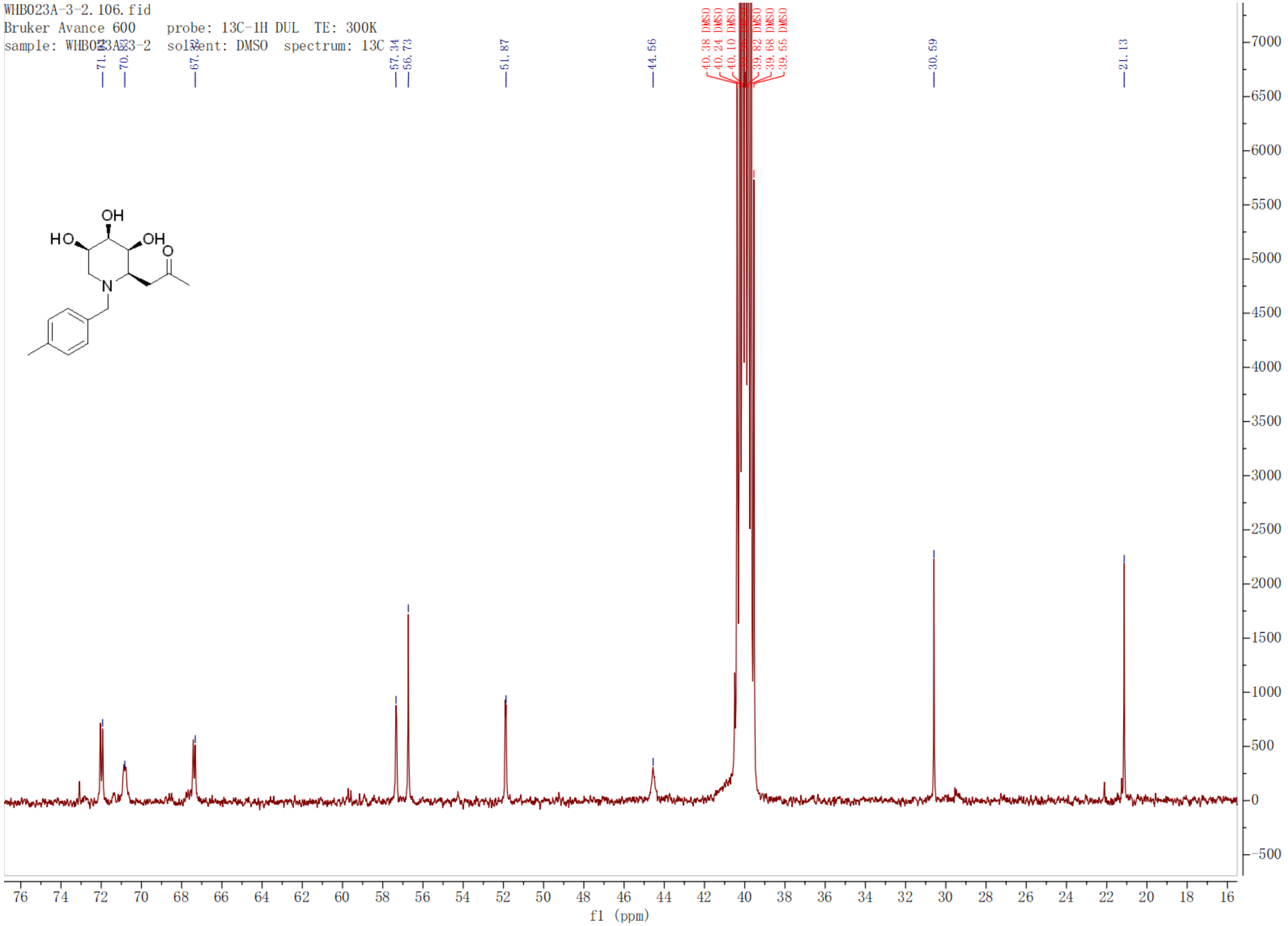

Figure S39. Partial region amplification of the ^{13}C NMR of compound **5j**.

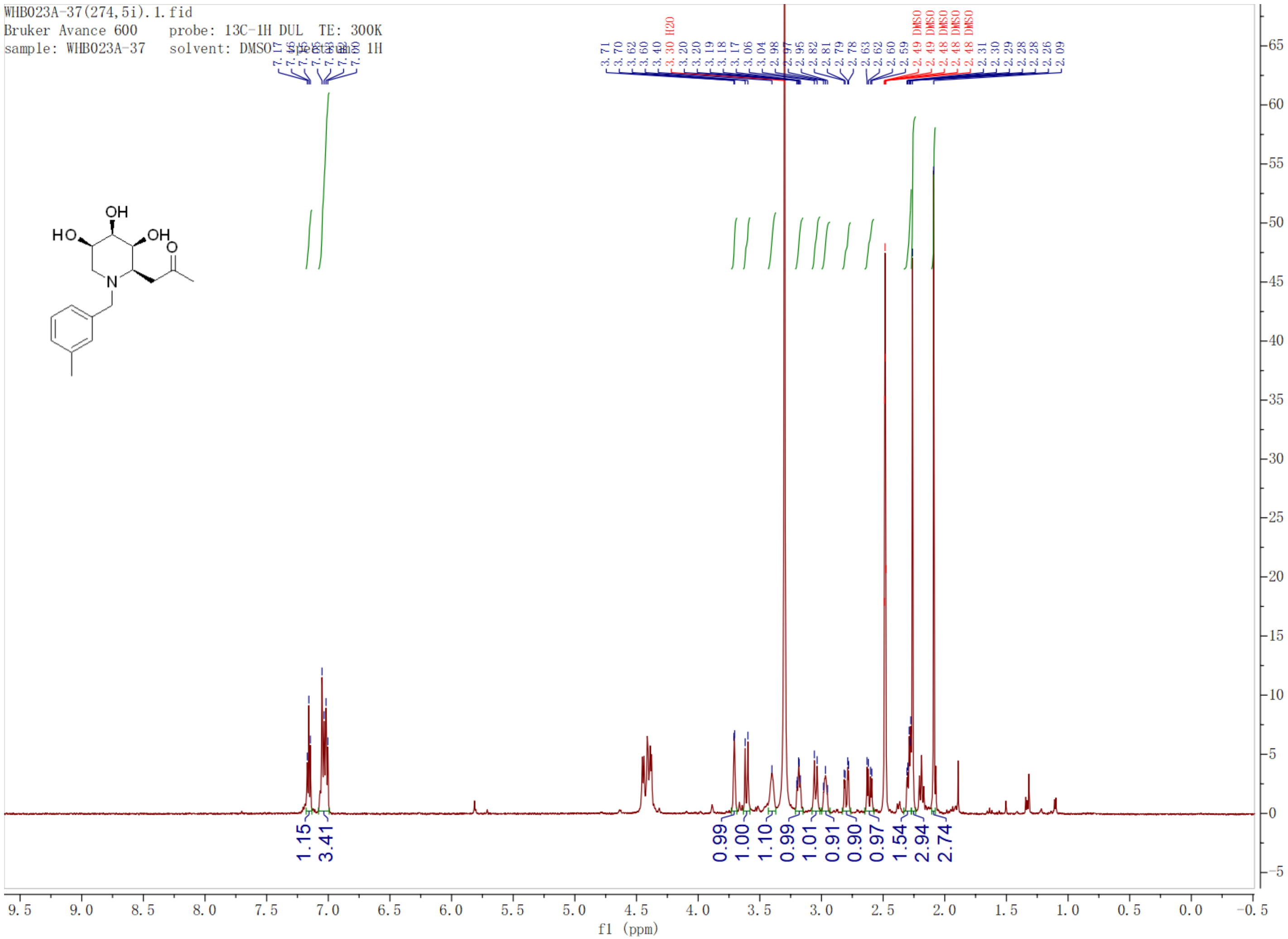

Figure S40. The ^{1}H NMR of compound **5k**.

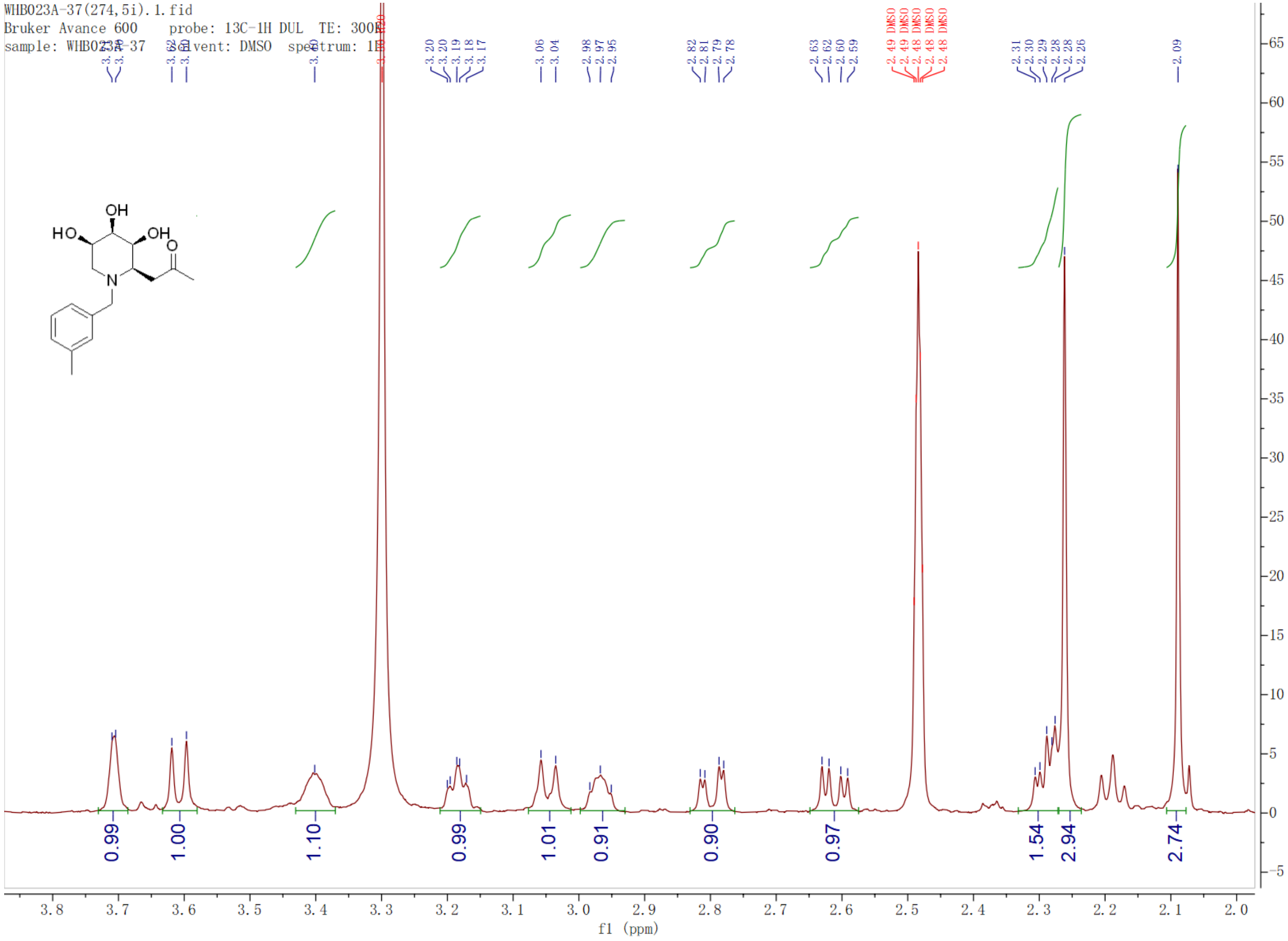

Figure S41. Partial region amplification of the ^{1}H NMR of compound **5k**.

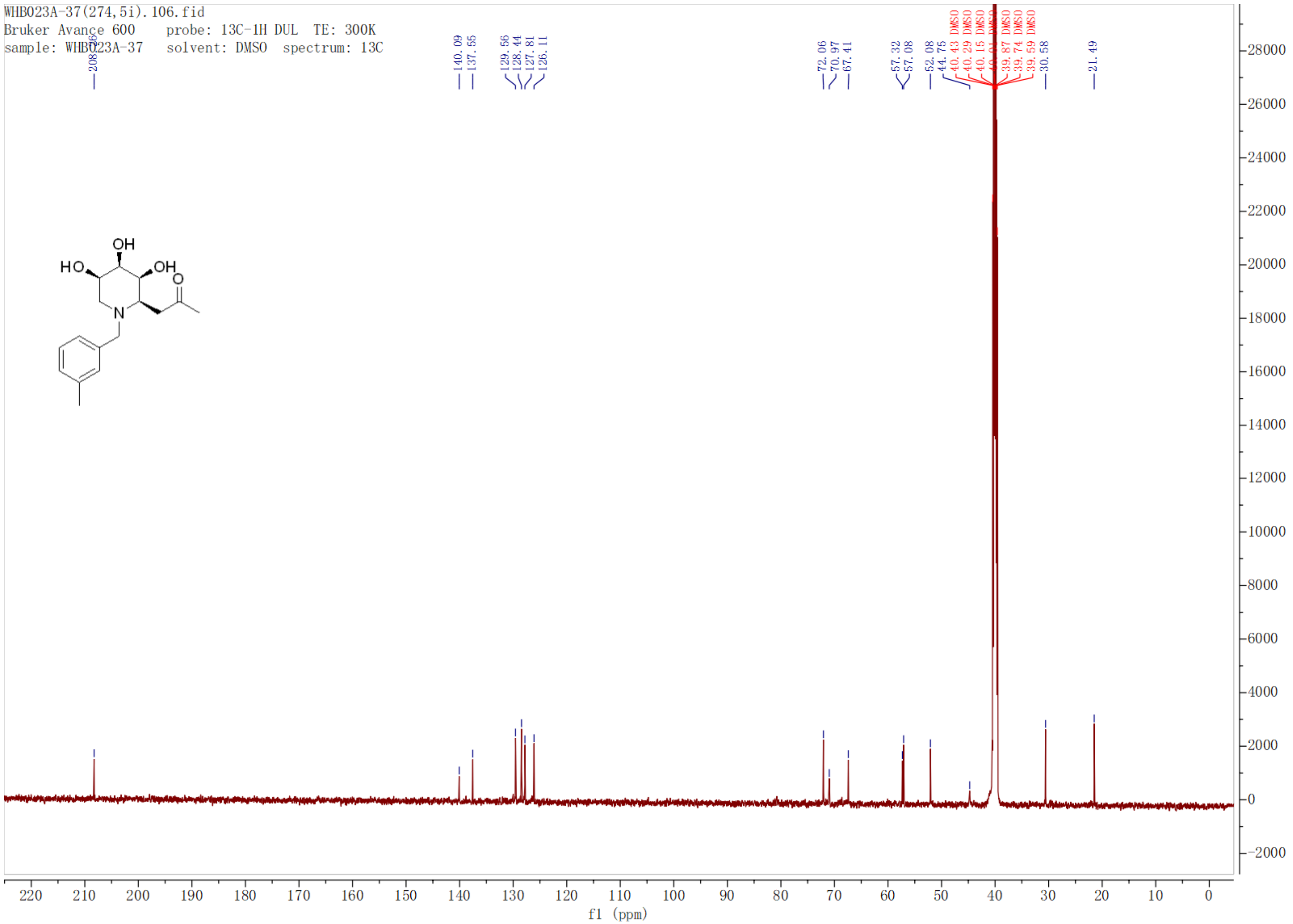

Figure S42. The ^{13}C NMR of compound **5k**.

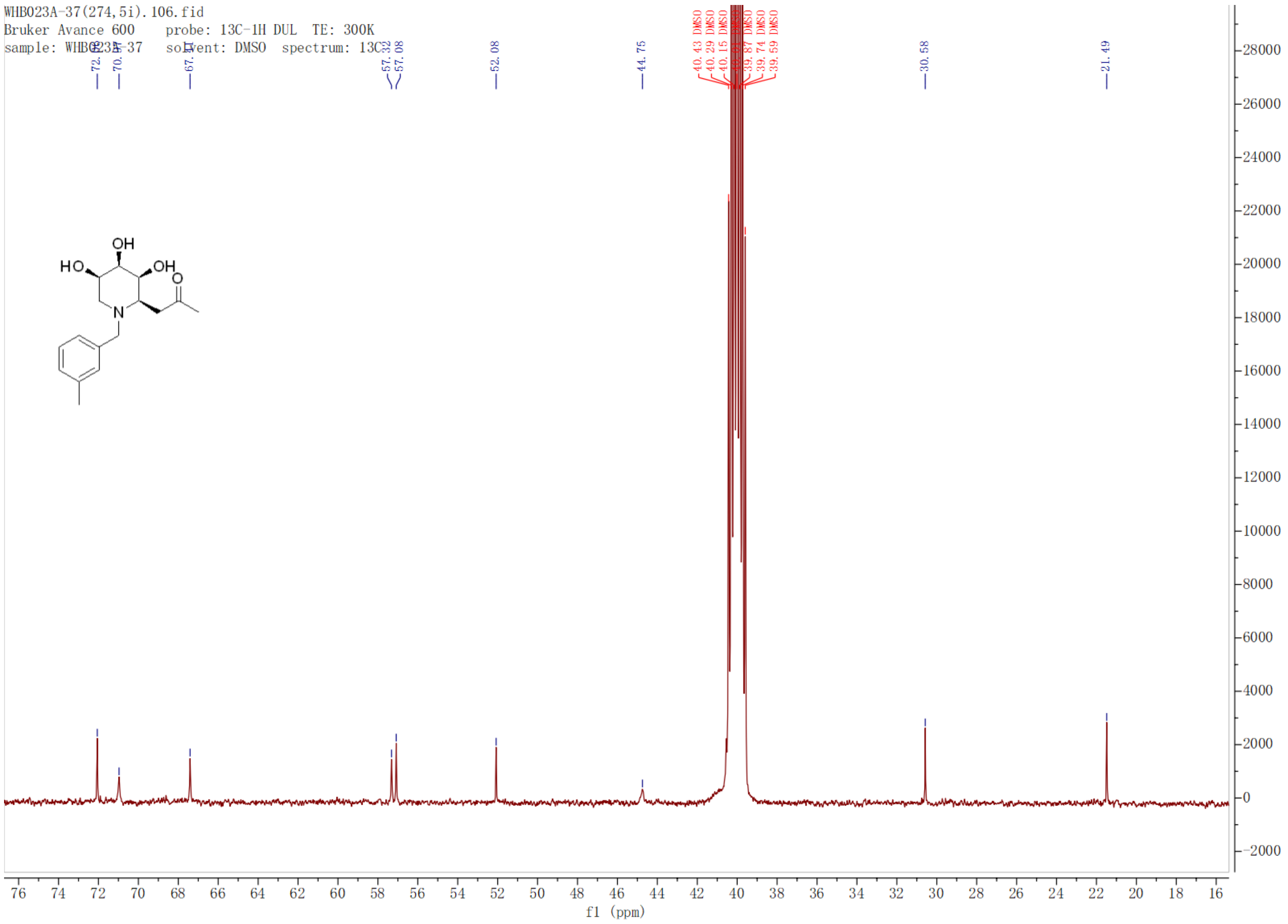

Figure S43. Partial region amplification of the ^{13}C NMR of compound **5k**.

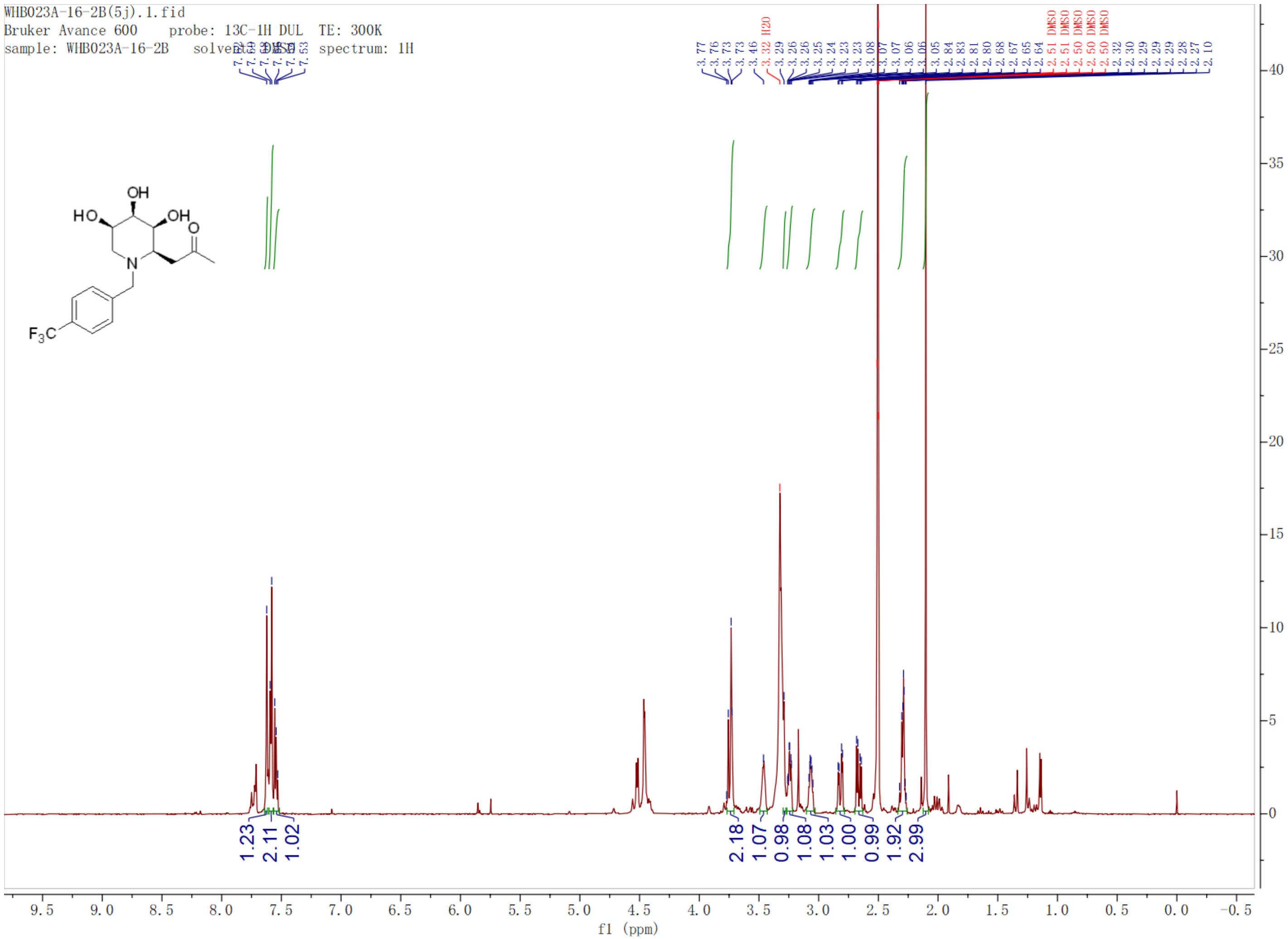

Figure S44. The ^{1}H NMR of compound **5l**.

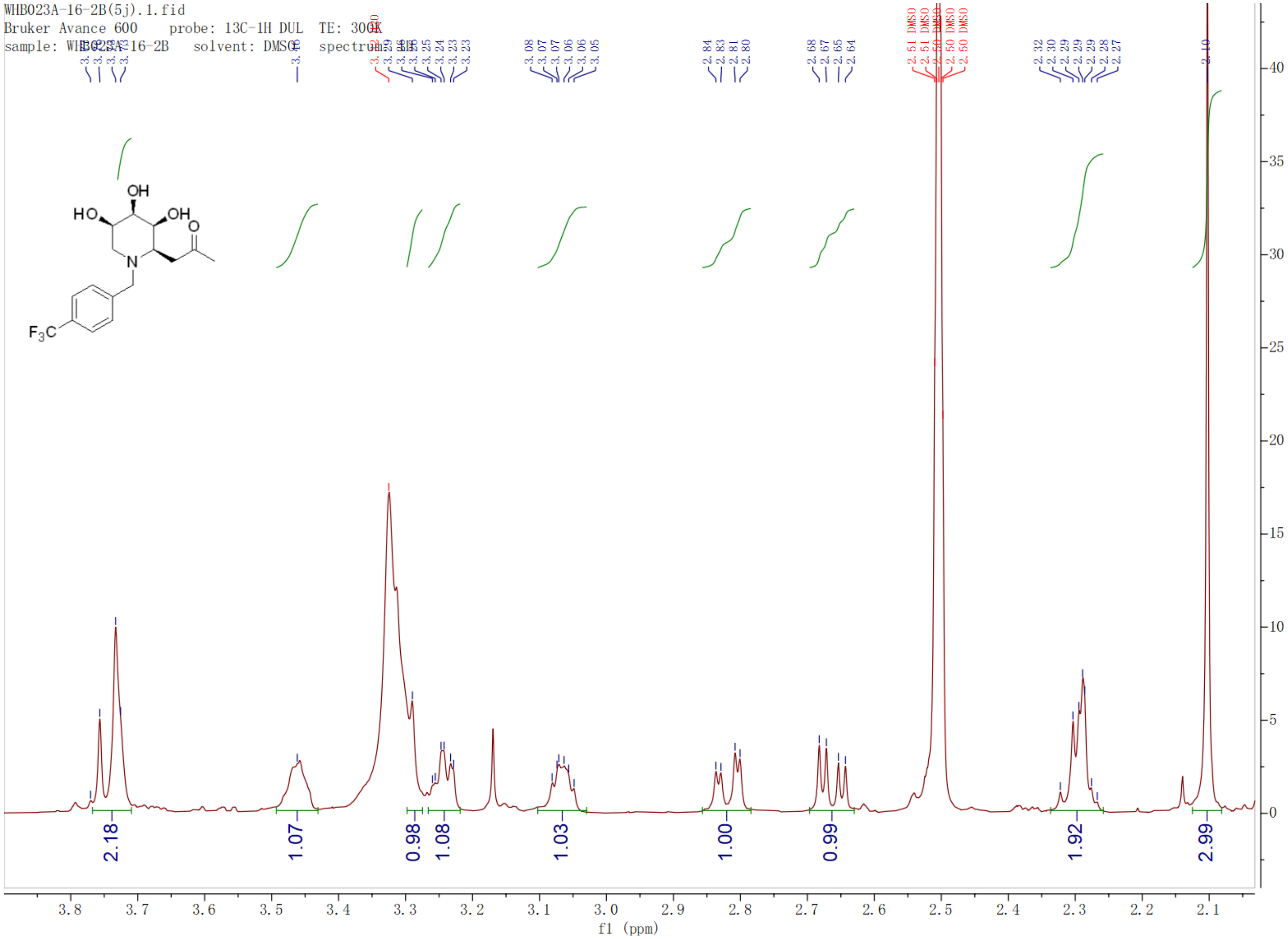

Figure S45. Partial region amplification of the ^{1}H NMR of compound **5l**.

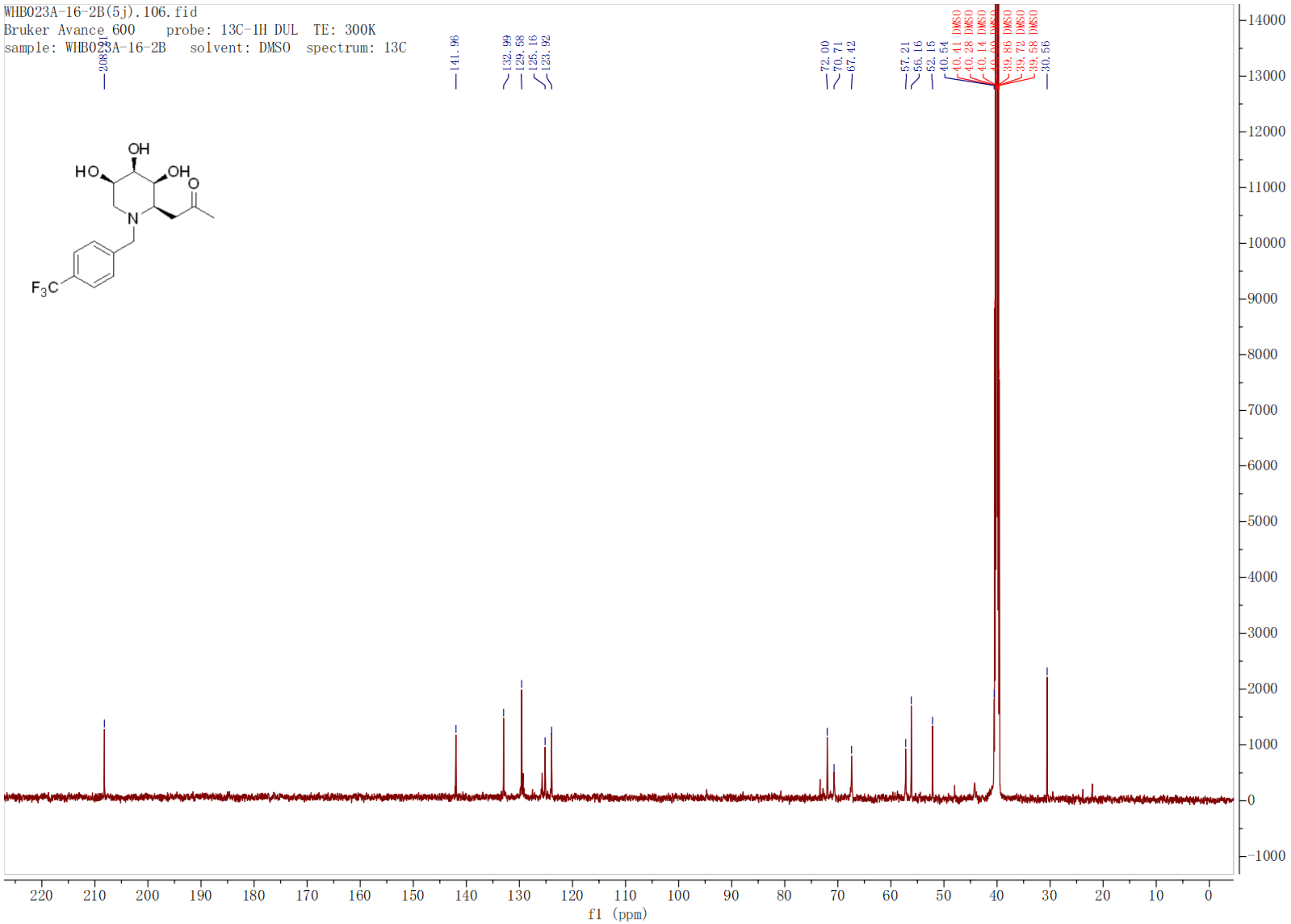

Figure S46. The ^{13}C NMR of compound **5l**.

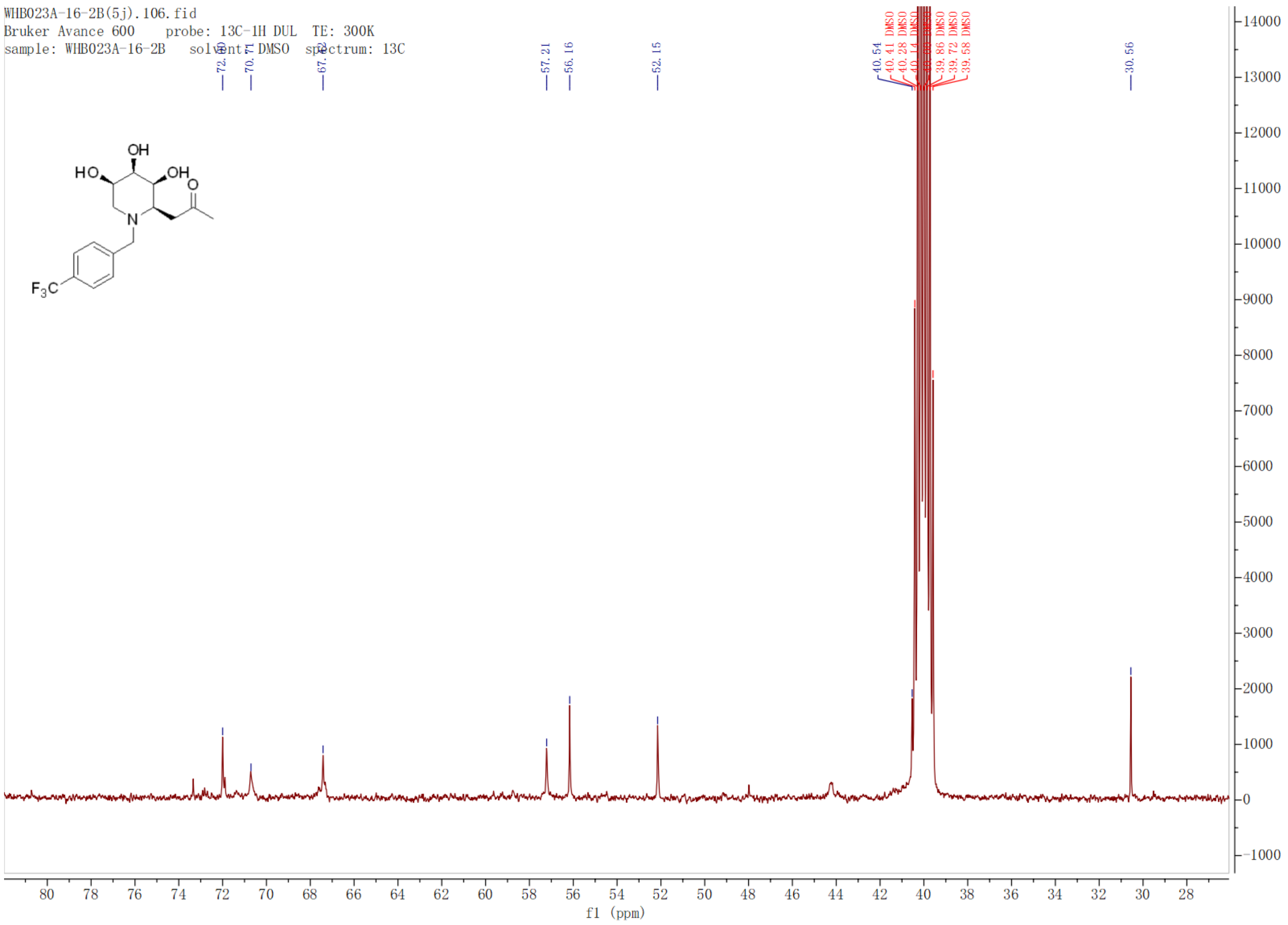

Figure S47. Partial region amplification of the ^{13}C NMR of compound **5l**.

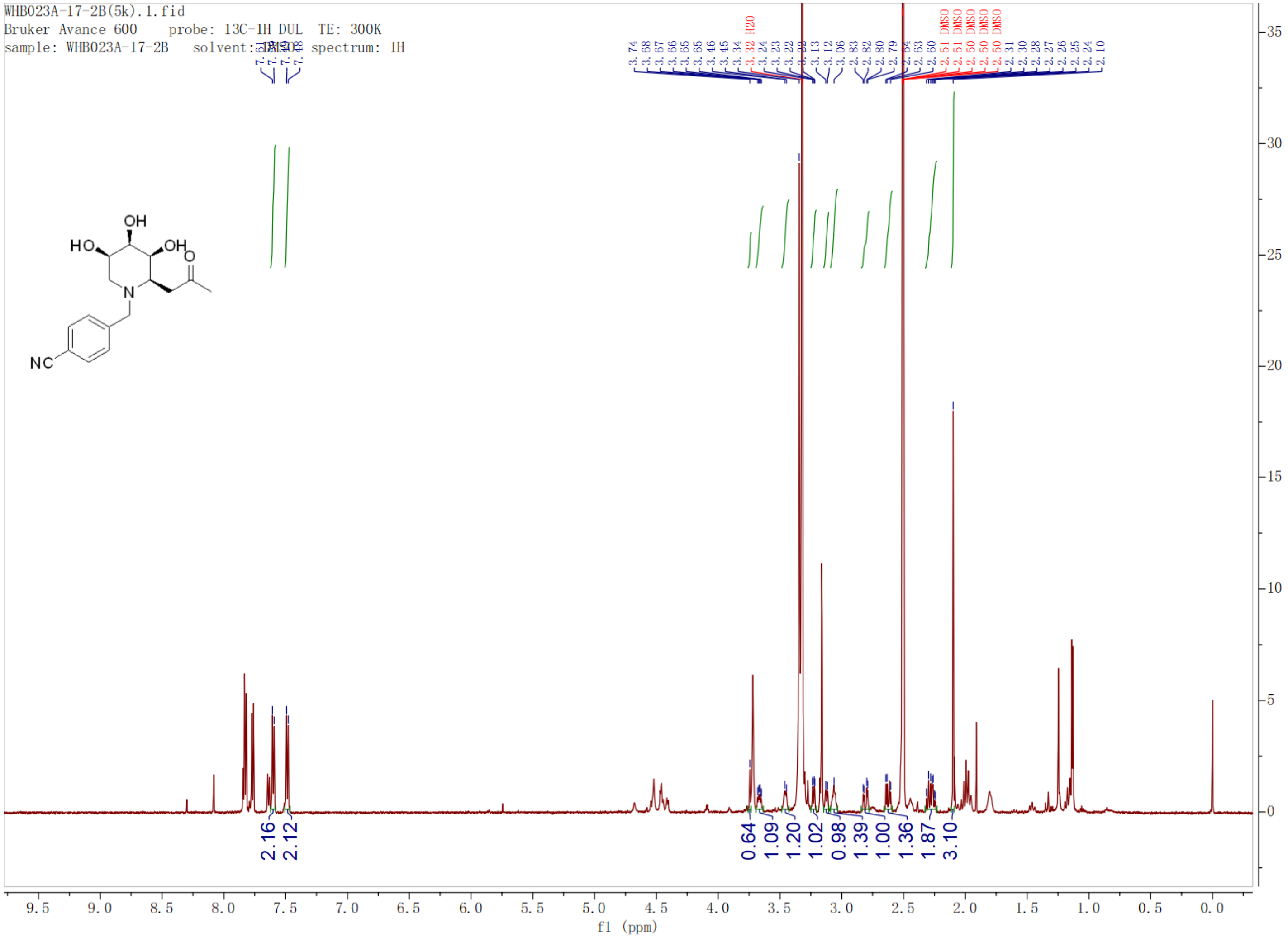

Figure S48. The ^{1}H NMR of compound **5m**.

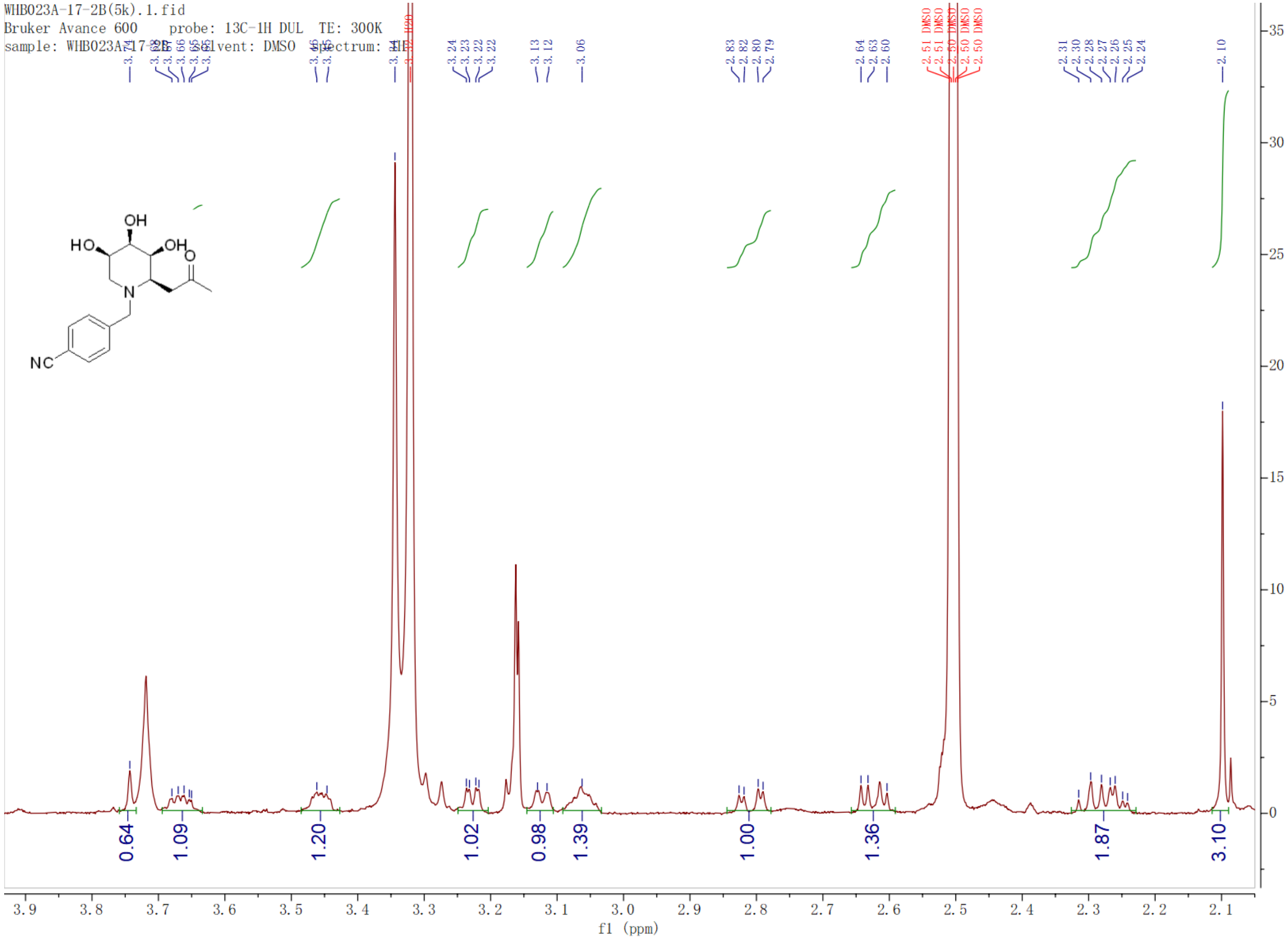

Figure S49. Partial region amplification of the ^{1}H NMR of compound **5m**.

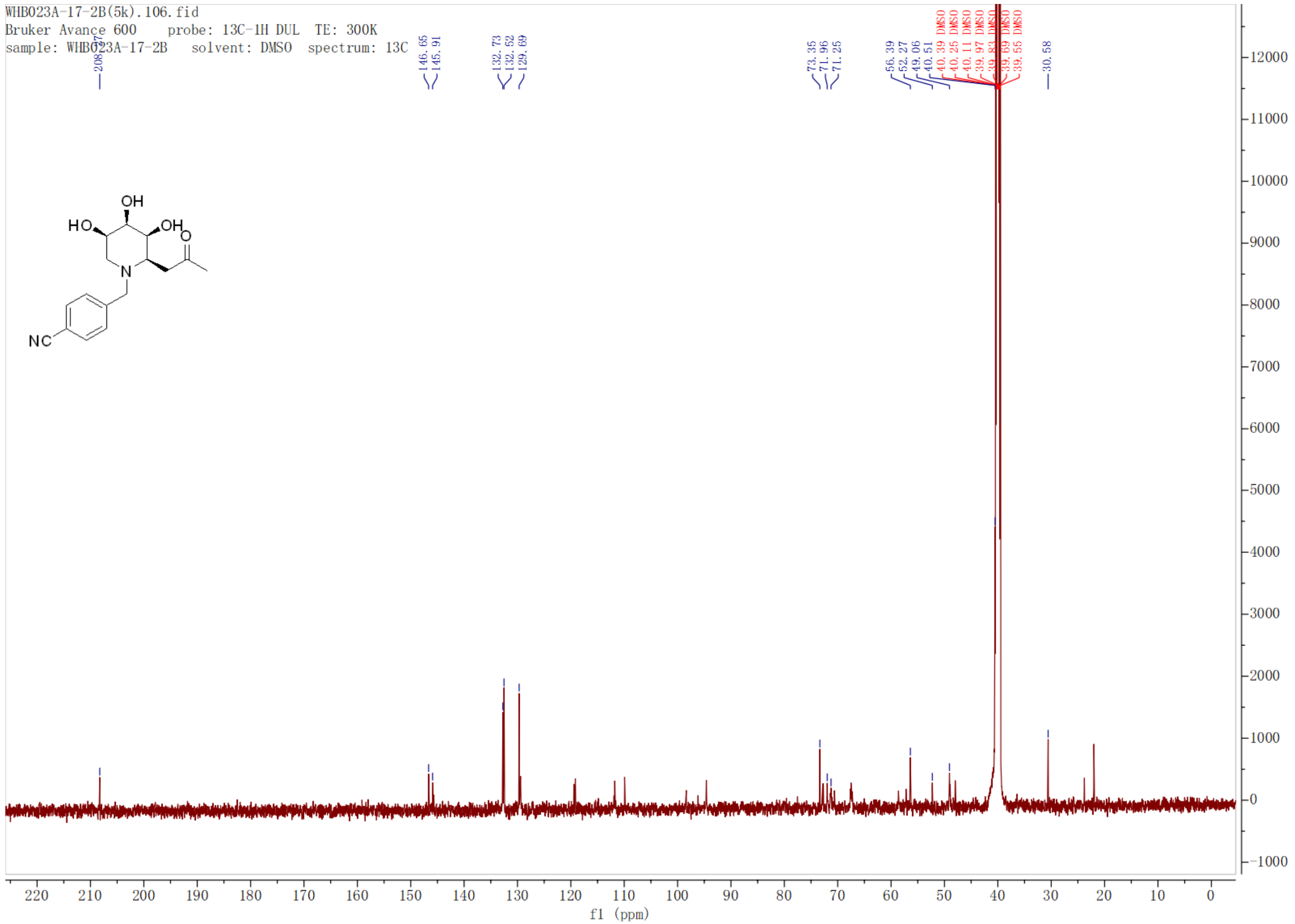

Figure S50. The ^{13}C NMR of compound **5m**.

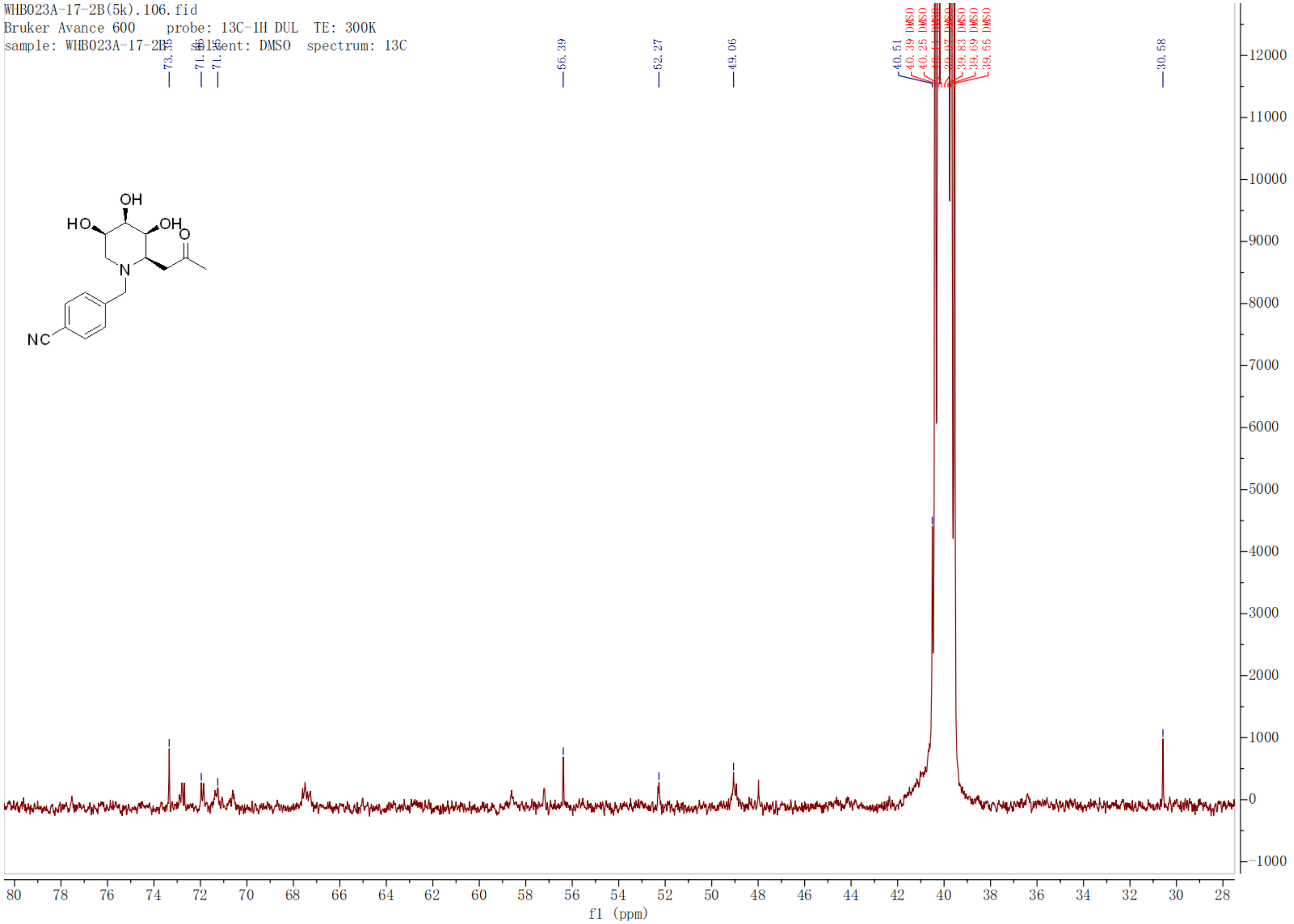

Figure S51. Partial region amplification of the ^{13}C NMR of compound **5m**.

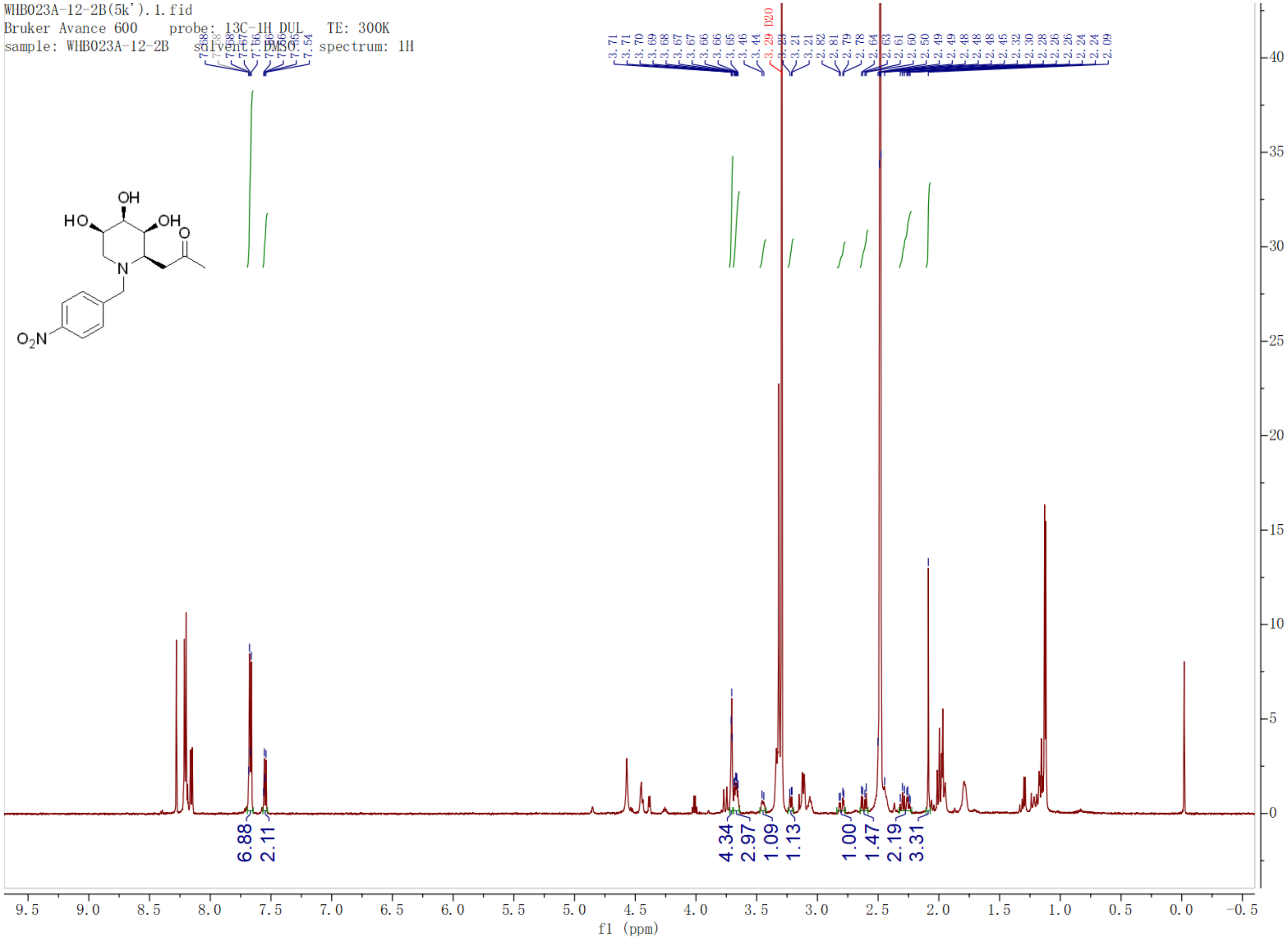

Figure S52. The ^{1}H NMR of compound **5n.**

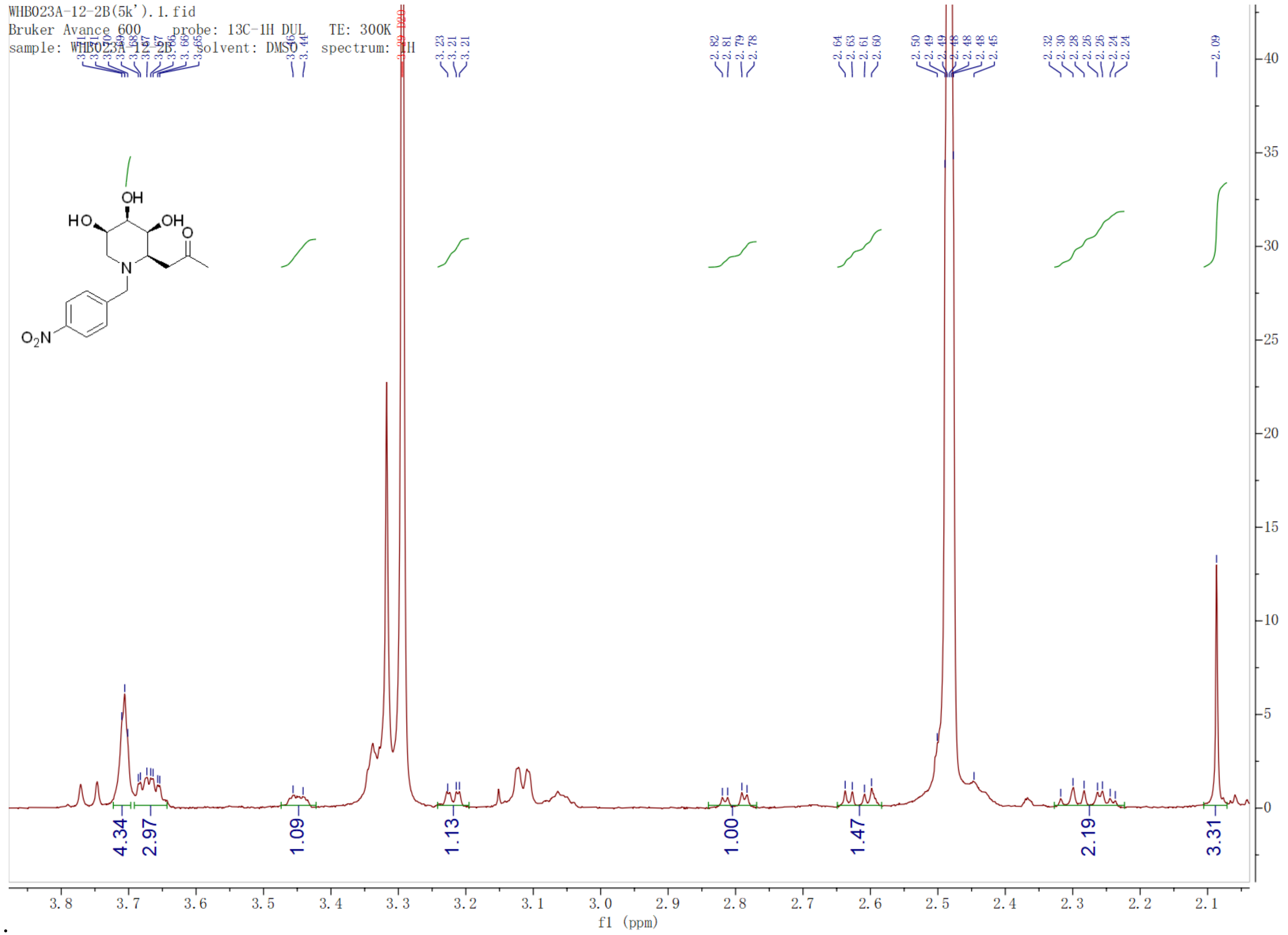

Figure S53. Partial region amplification of the ^{1}H NMR of compound **5n**.

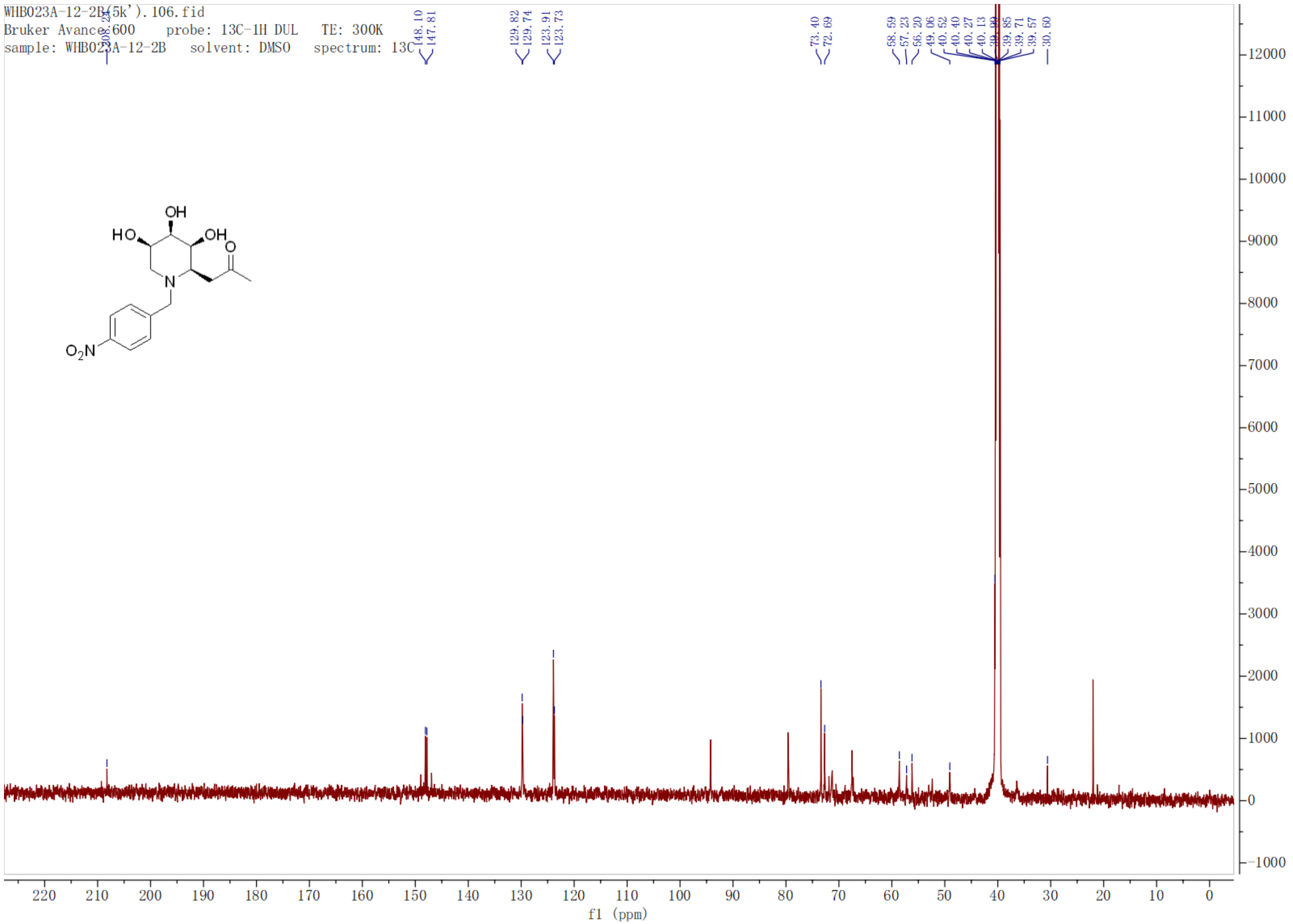

Figure S54. The ^{13}C NMR of compound **5n**.

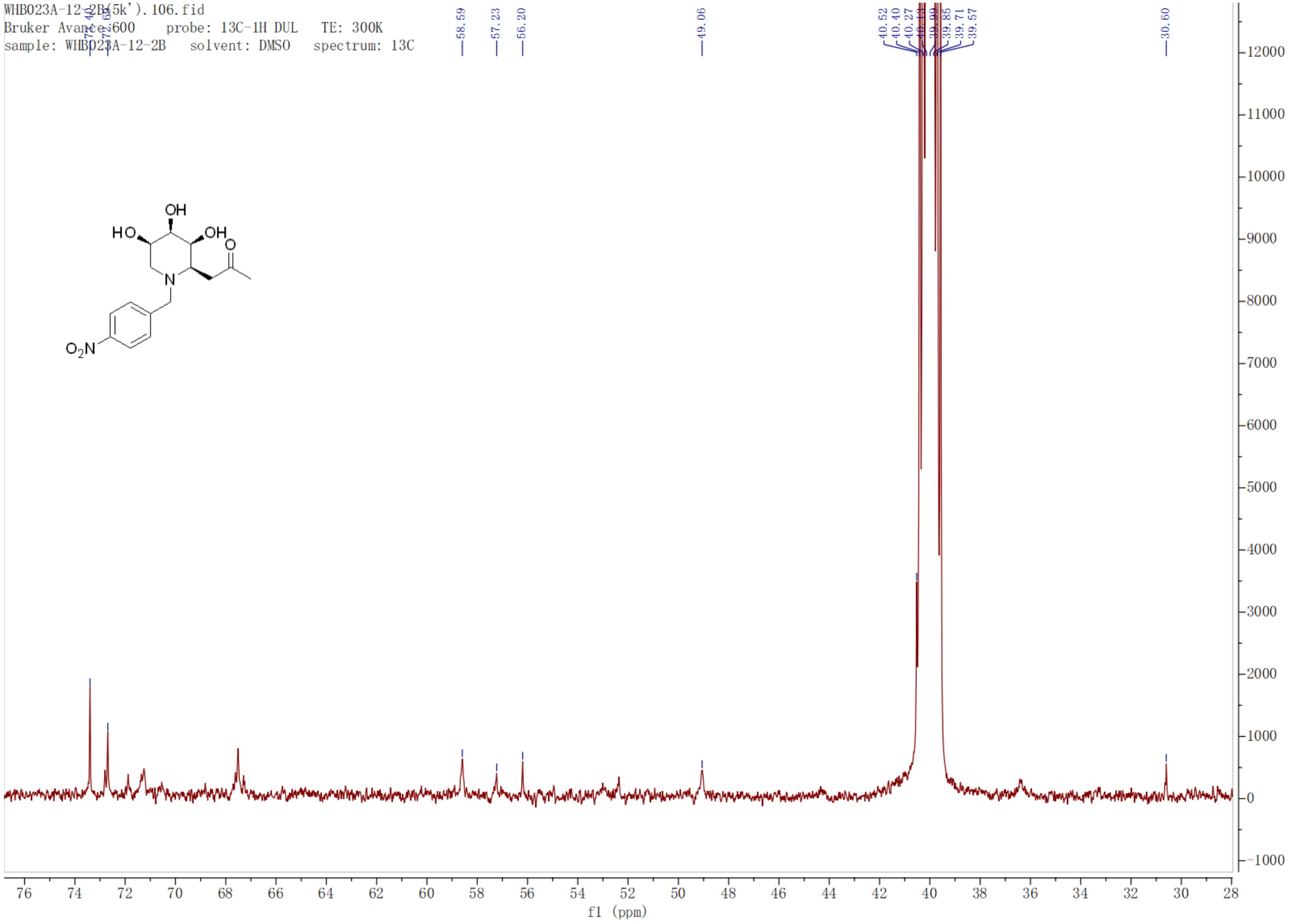

Figure S55. Partial region amplification of the ^{13}C NMR of compound **5n**.

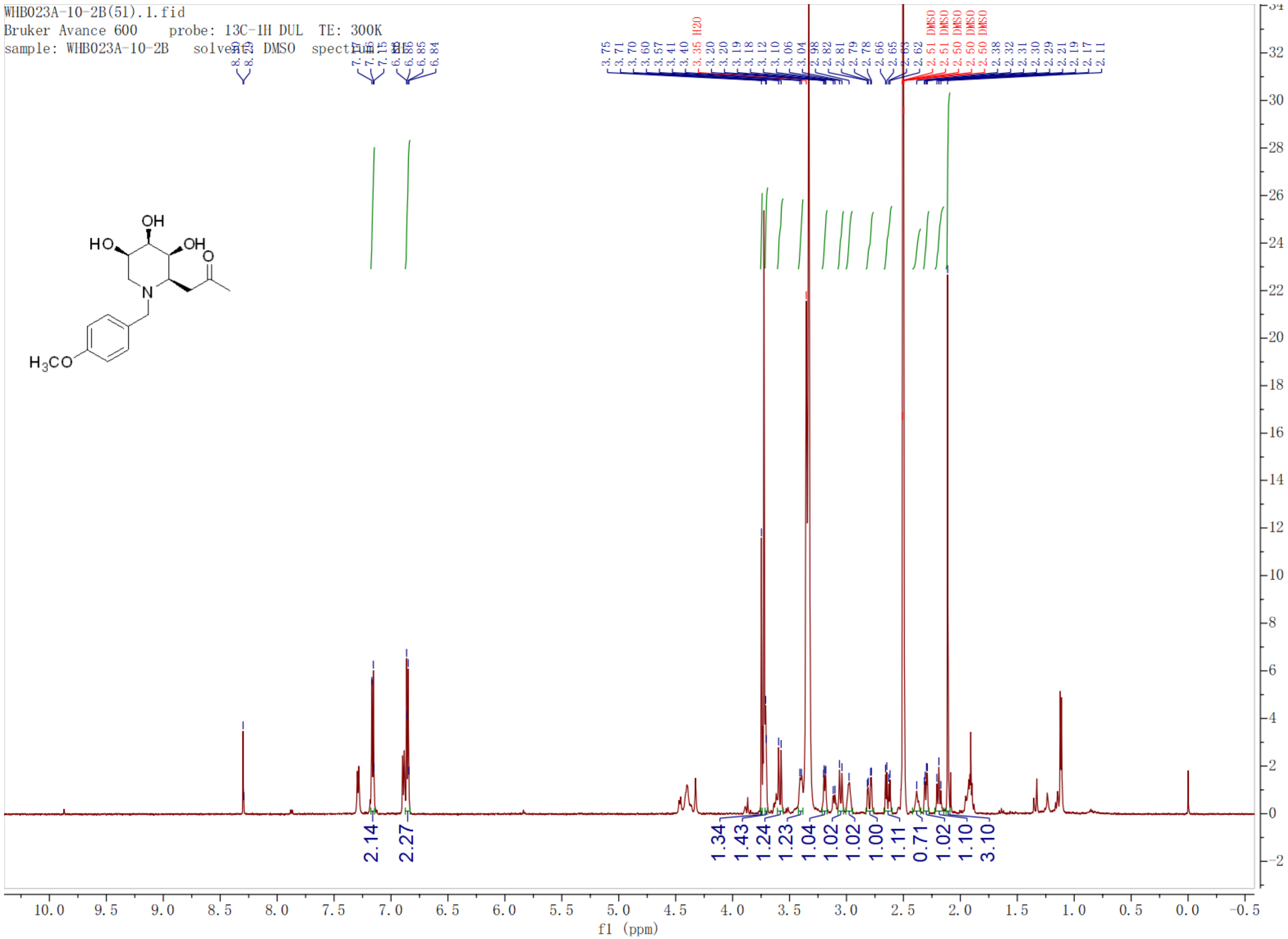

Figure S56. The ^1H NMR of compound **5o**.

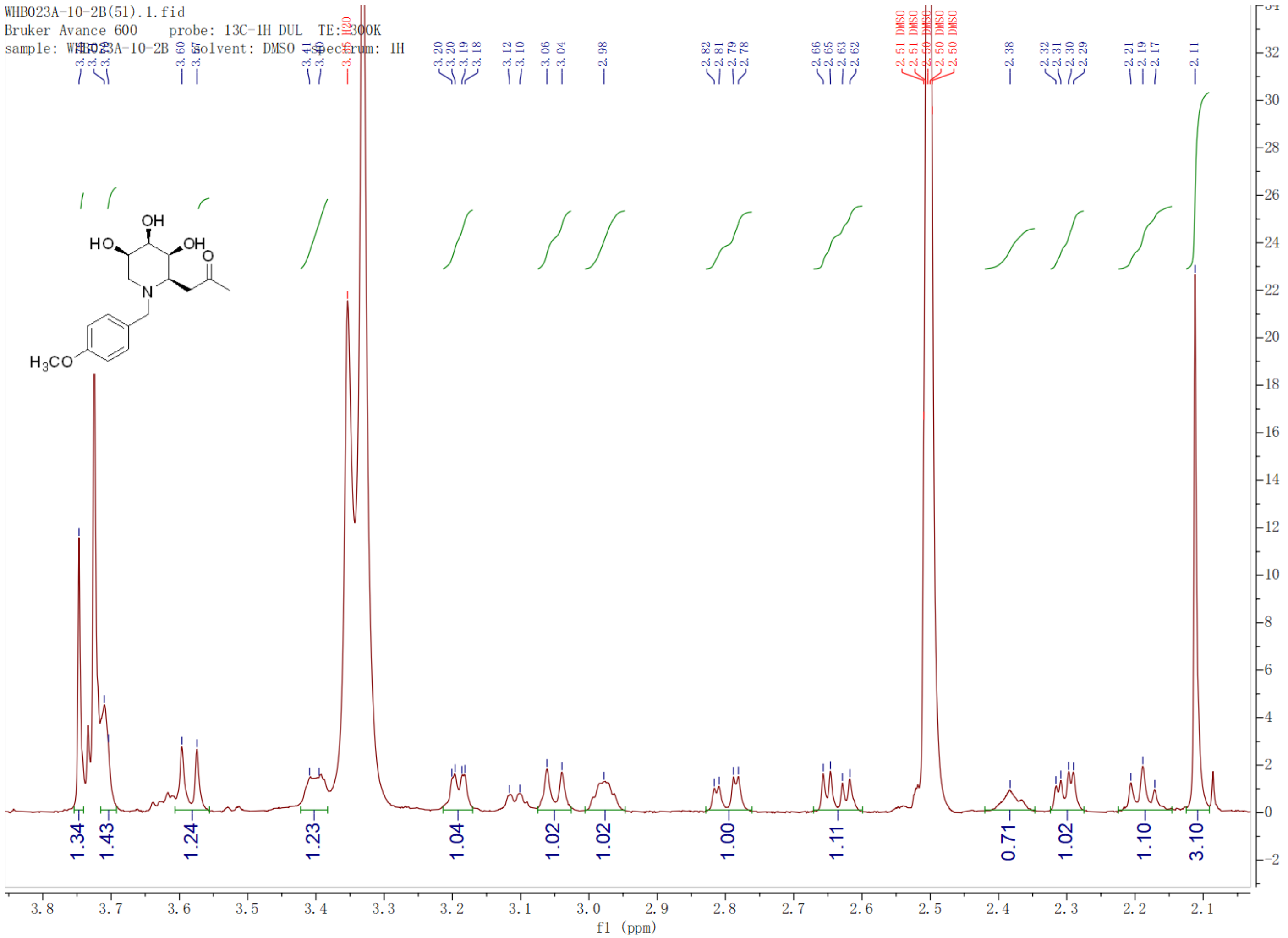

Figure S57. Partial region amplification of the ^{1}H NMR of compound **5o**.

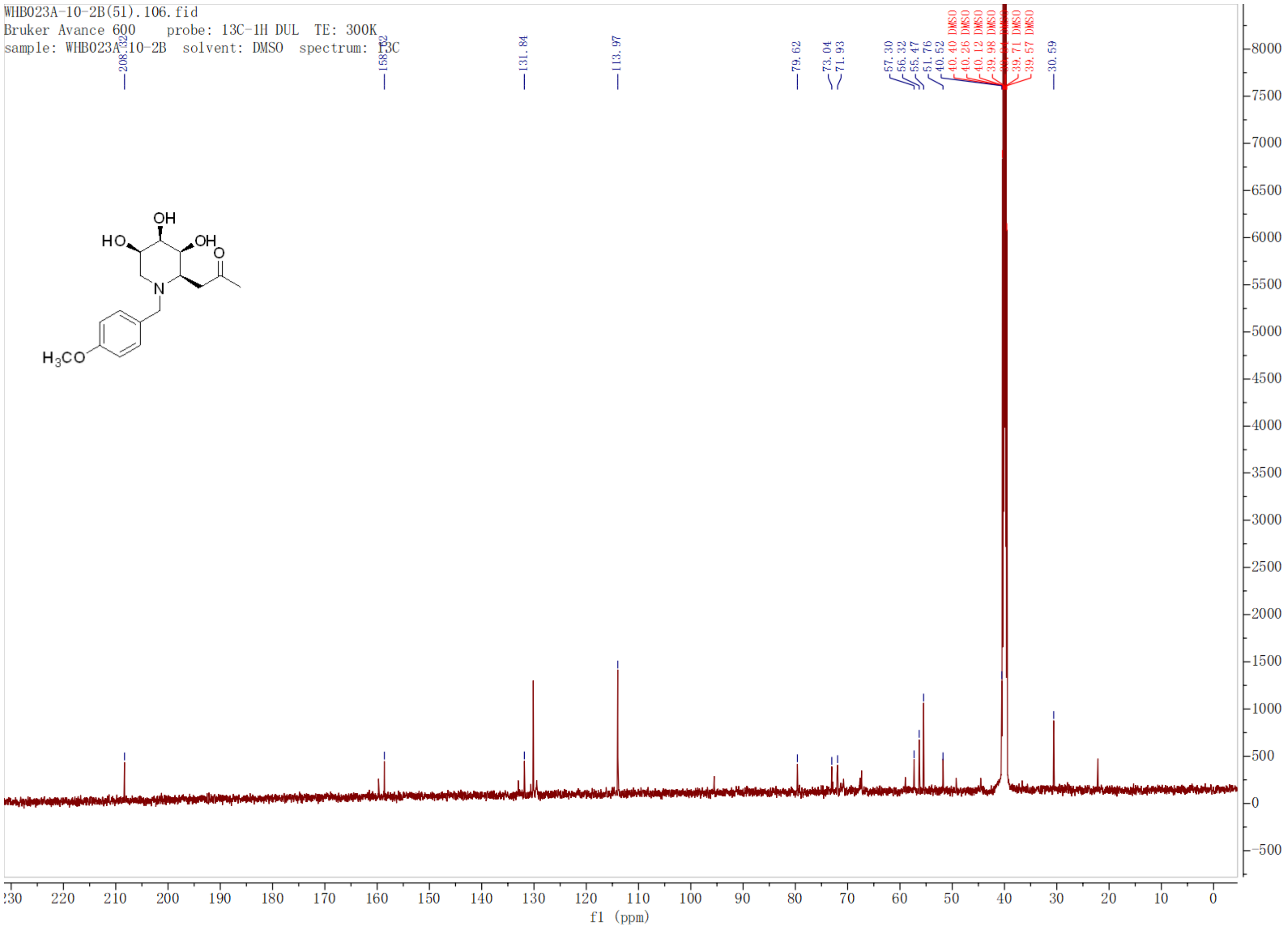

Figure S58. The ^{13}C NMR of compound **5o**.

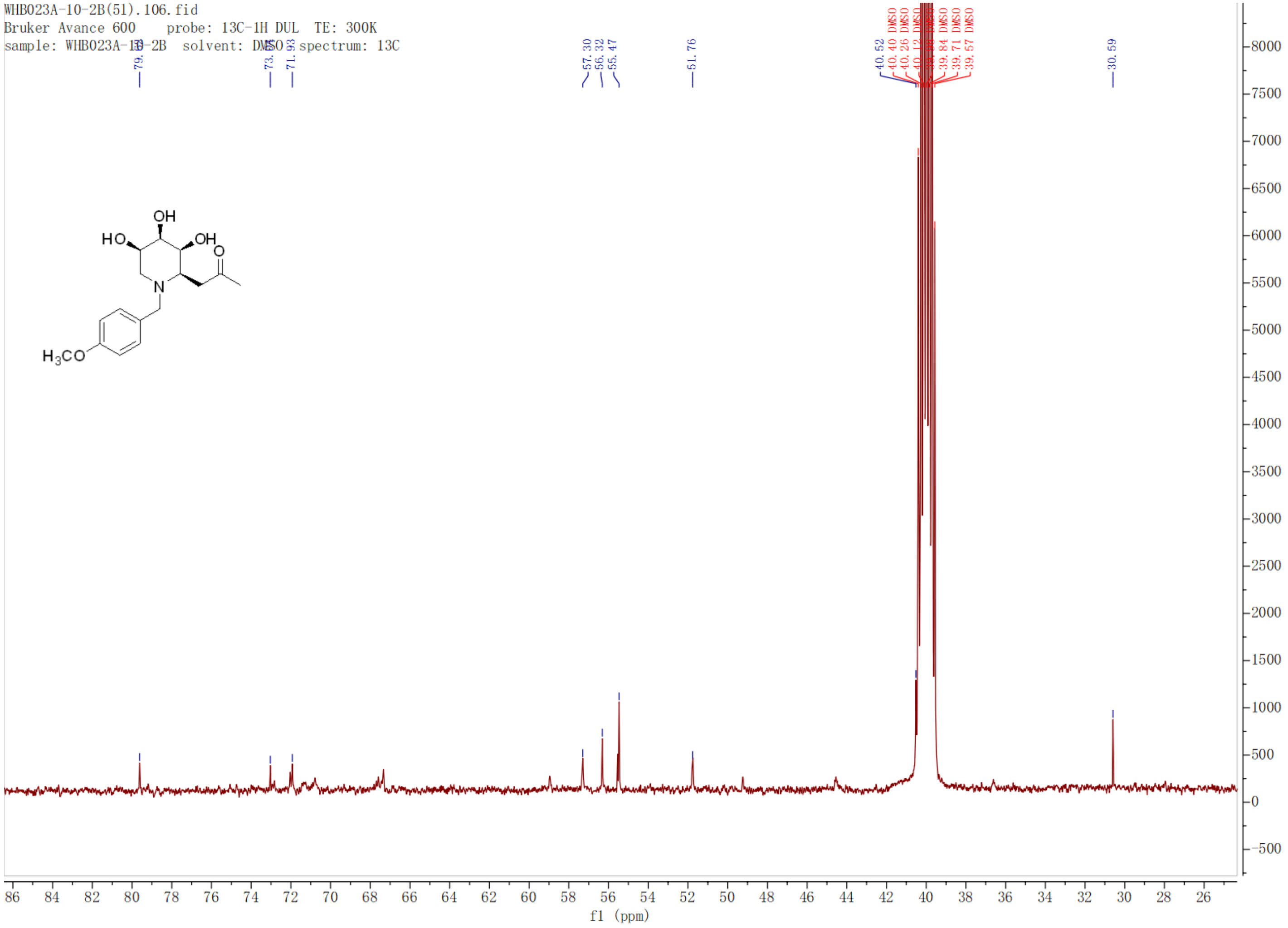

Figure S59. Partial region amplification of the ^{13}C NMR of compound **5o**.

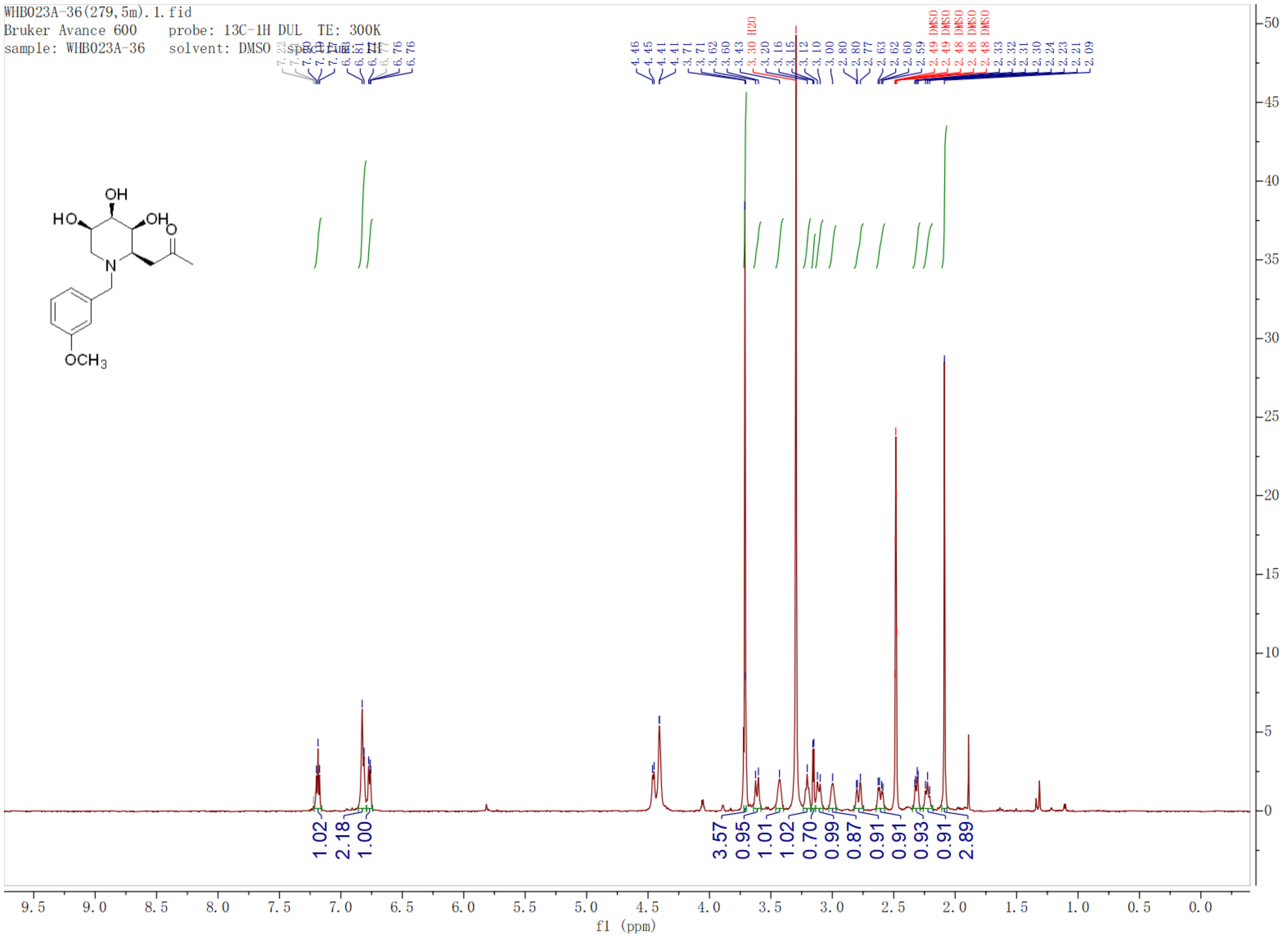

Figure S60. The ^{1}H NMR of compound **5p**.

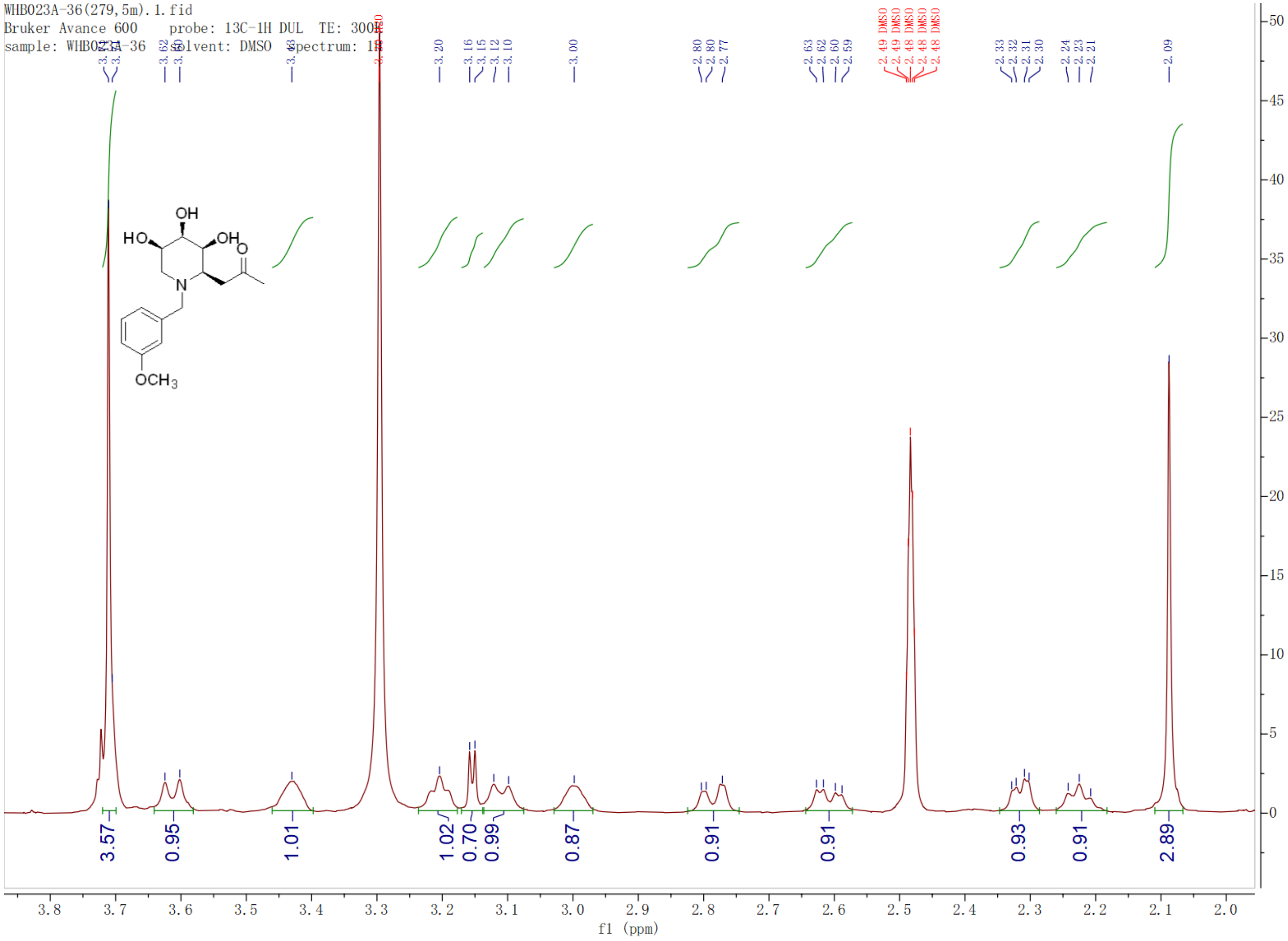

Figure S61. Partial region amplification of the ^{1}H NMR of compound **5p**.

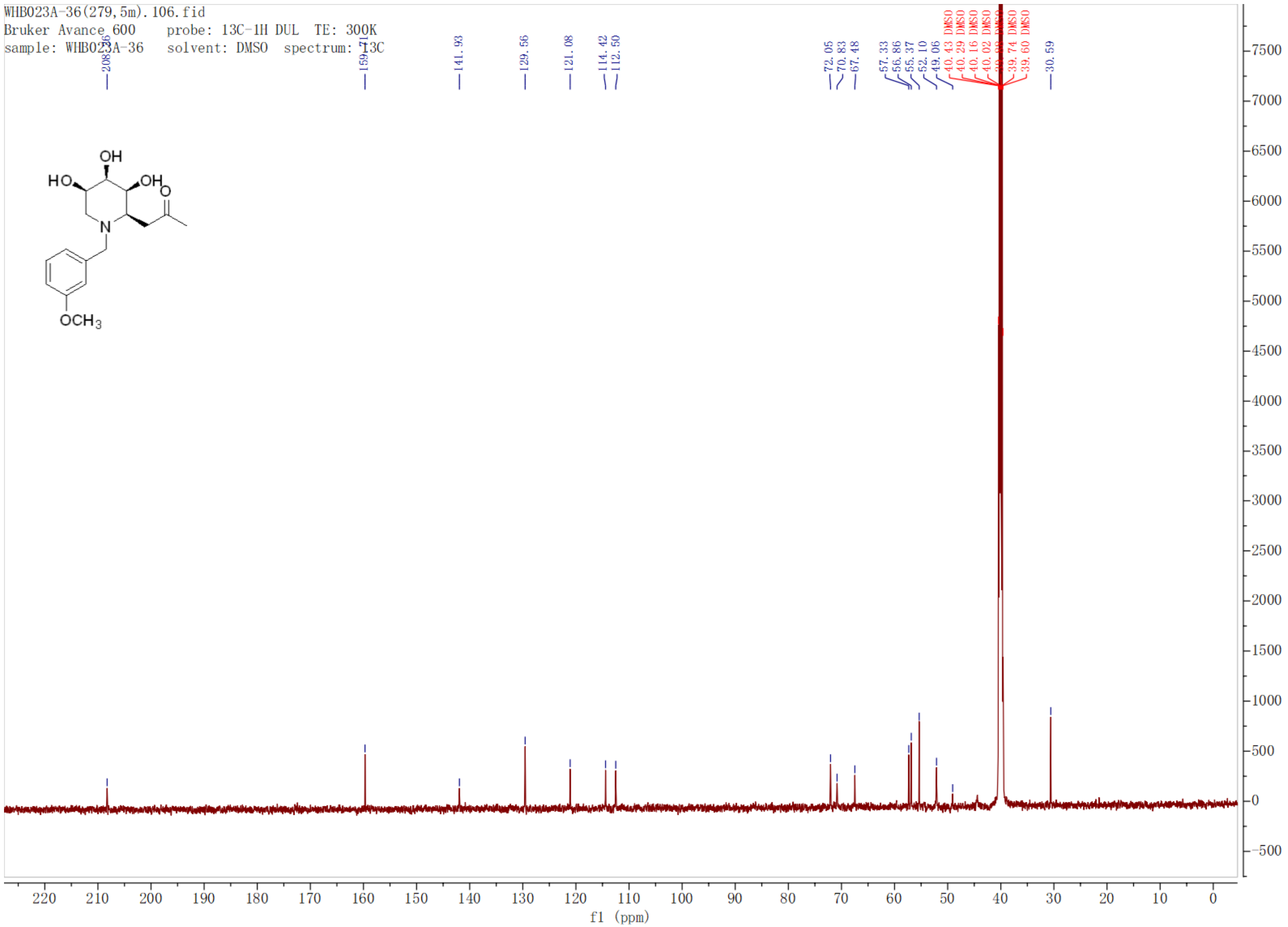

Figure S62. The ^{13}C NMR of compound **5p**.

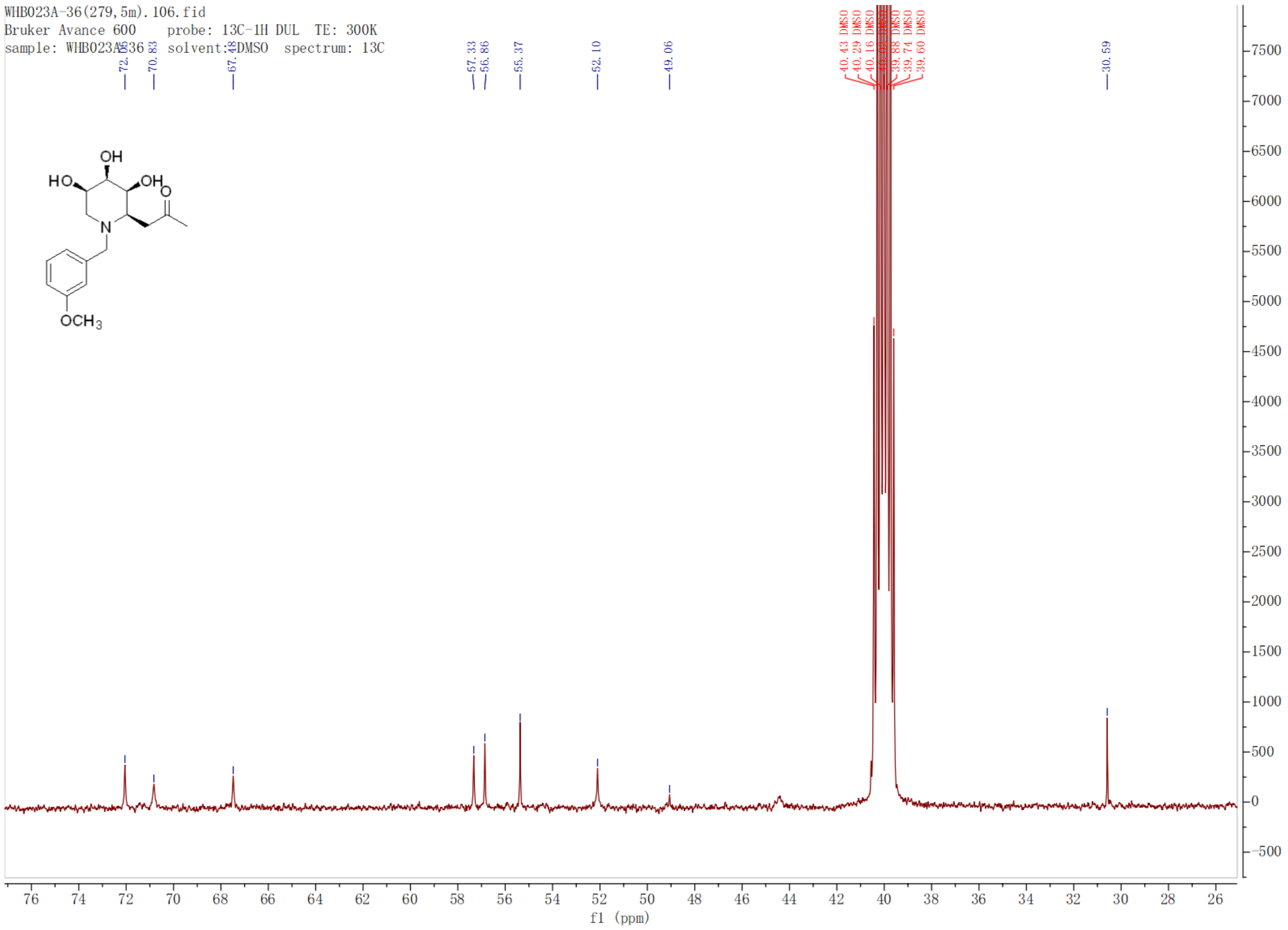

Figure S63. Partial region amplification of the ^{13}C NMR of compound **5p**.

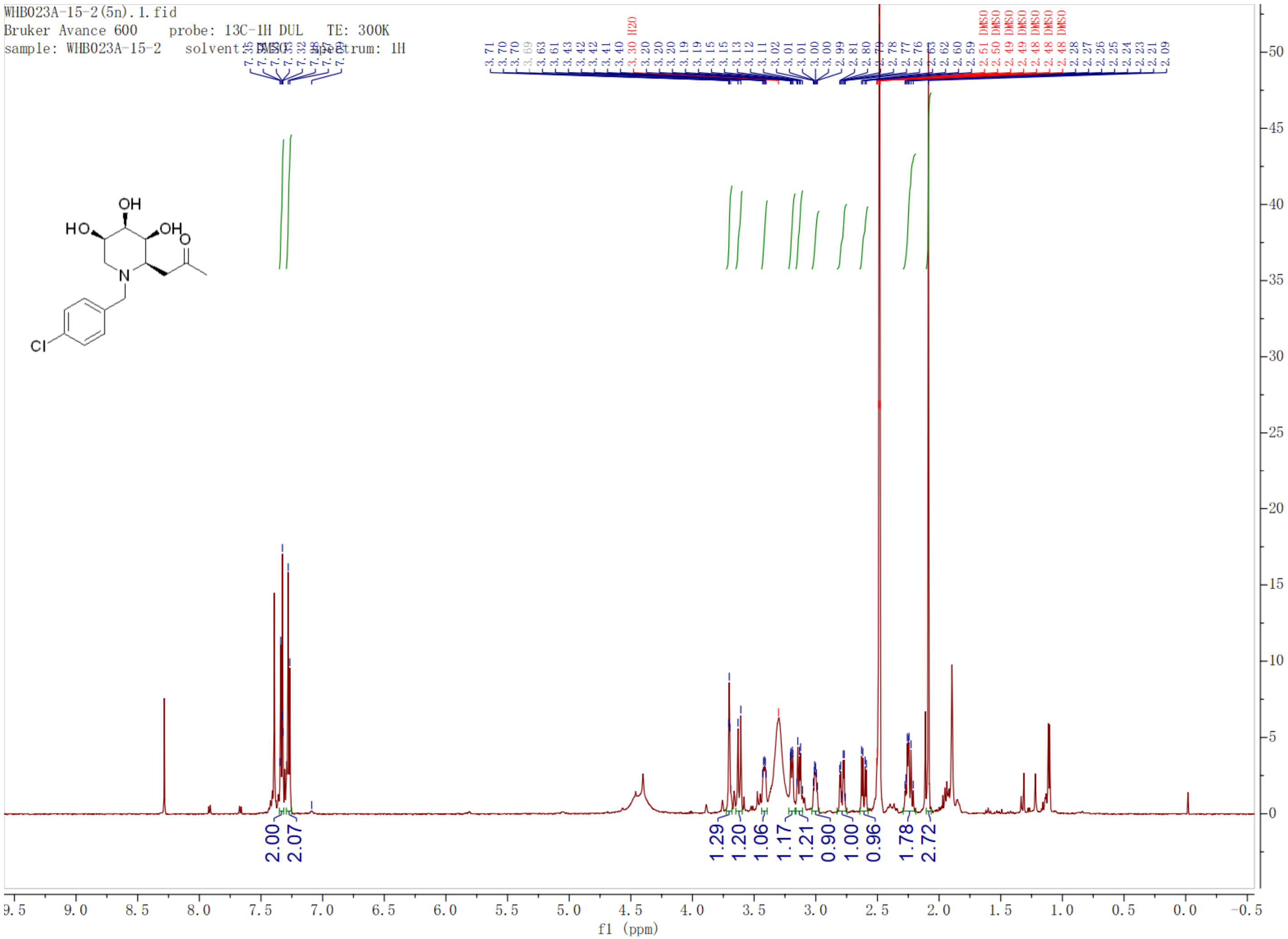

Figure S64. The ^{1}H NMR of compound **5q**.

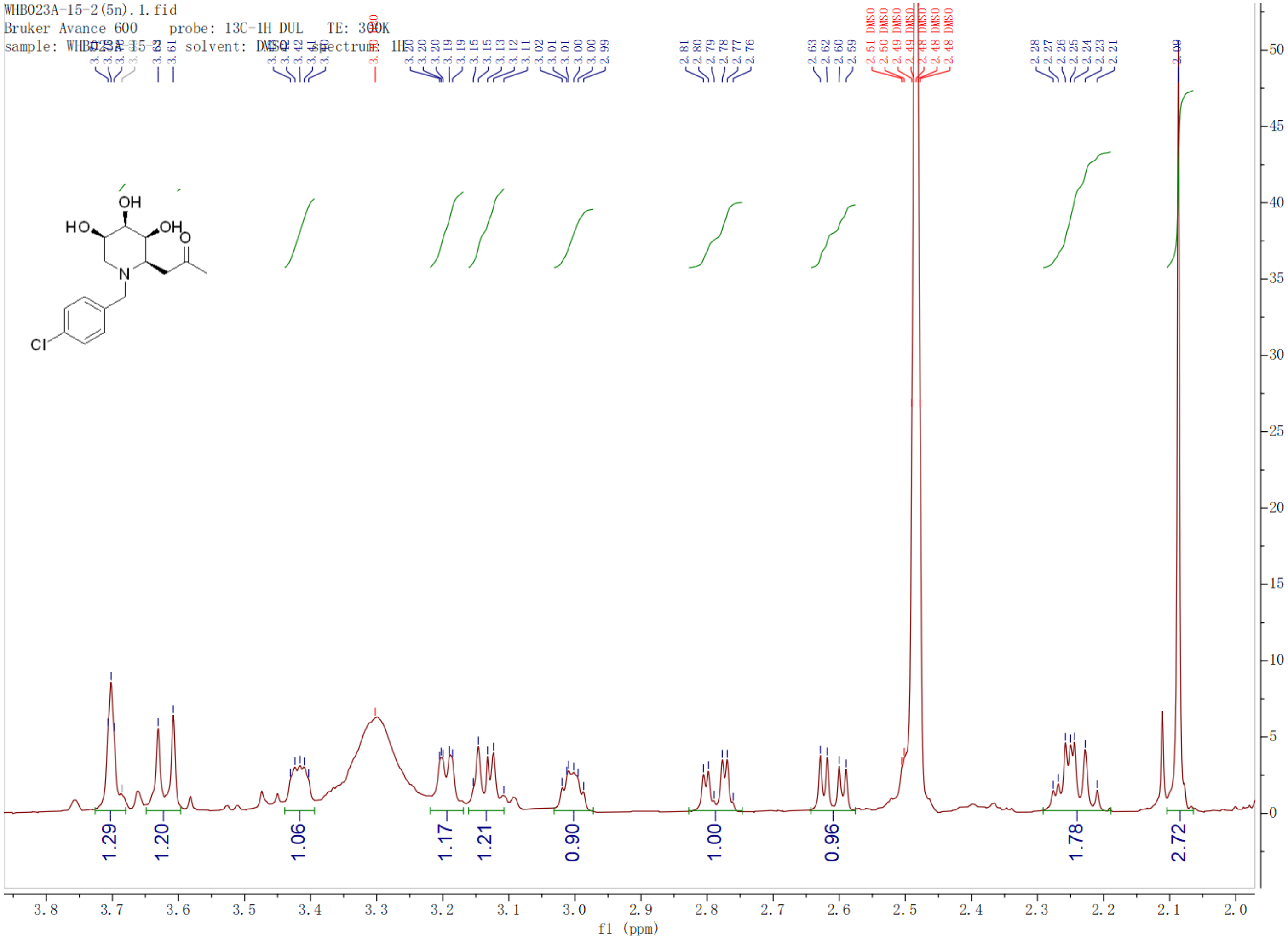

Figure S65. Partial region amplification of the ^{1}H NMR of compound **5q**.

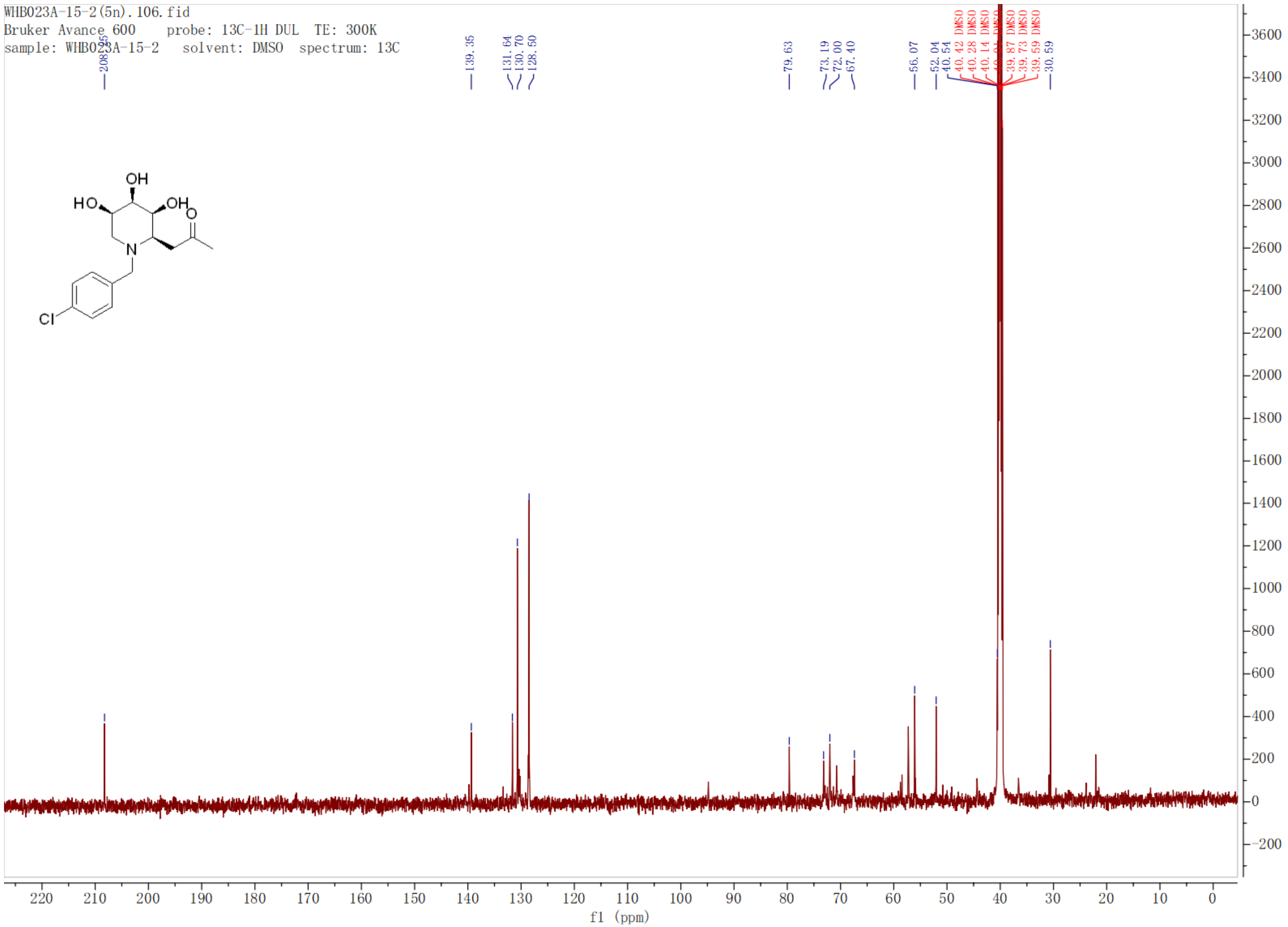

Figure S66. The ¹³C NMR of compound **5q**.

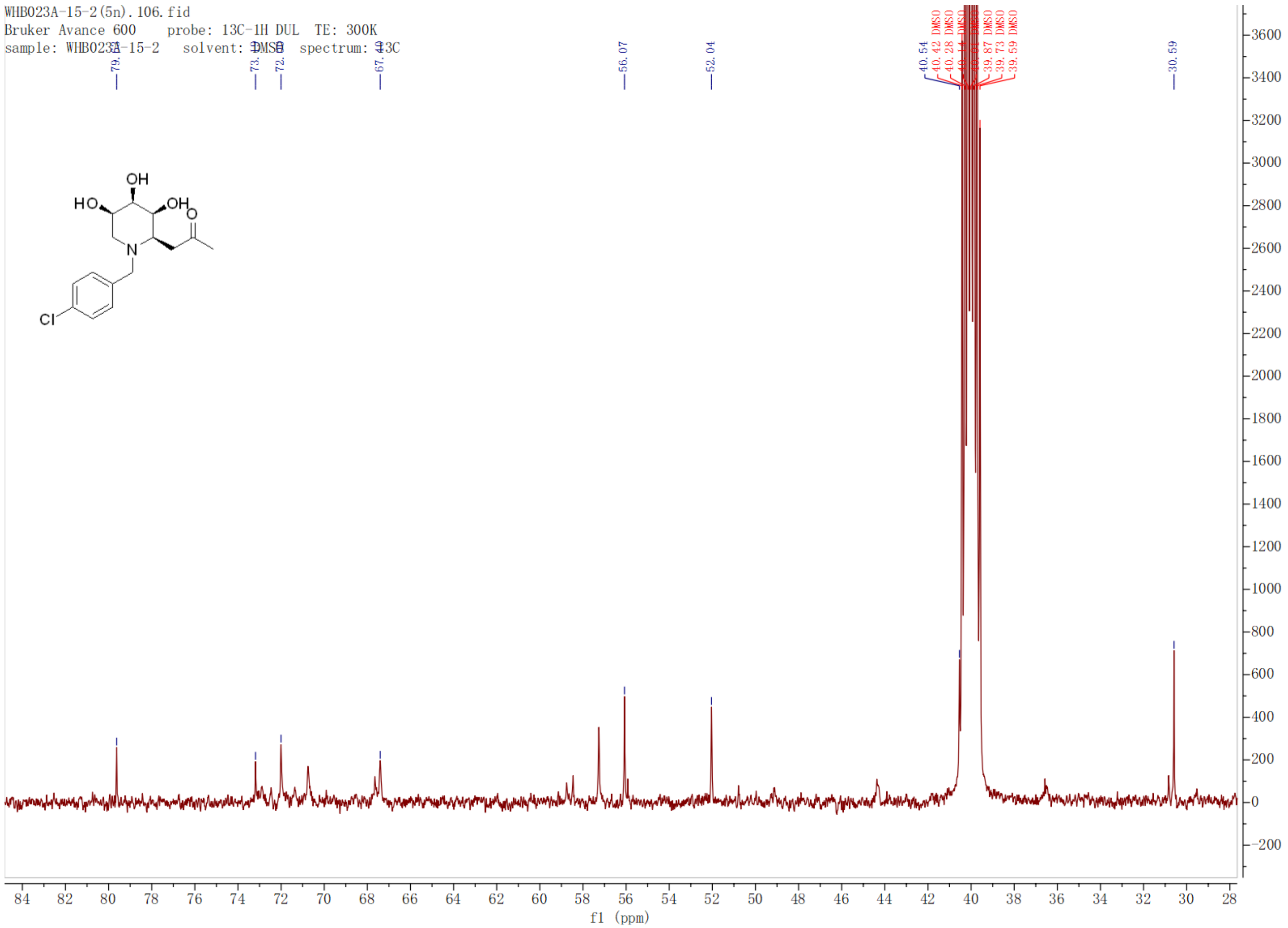

Figure S67. Partial region amplification of the ^{13}C NMR of compound **5q**.

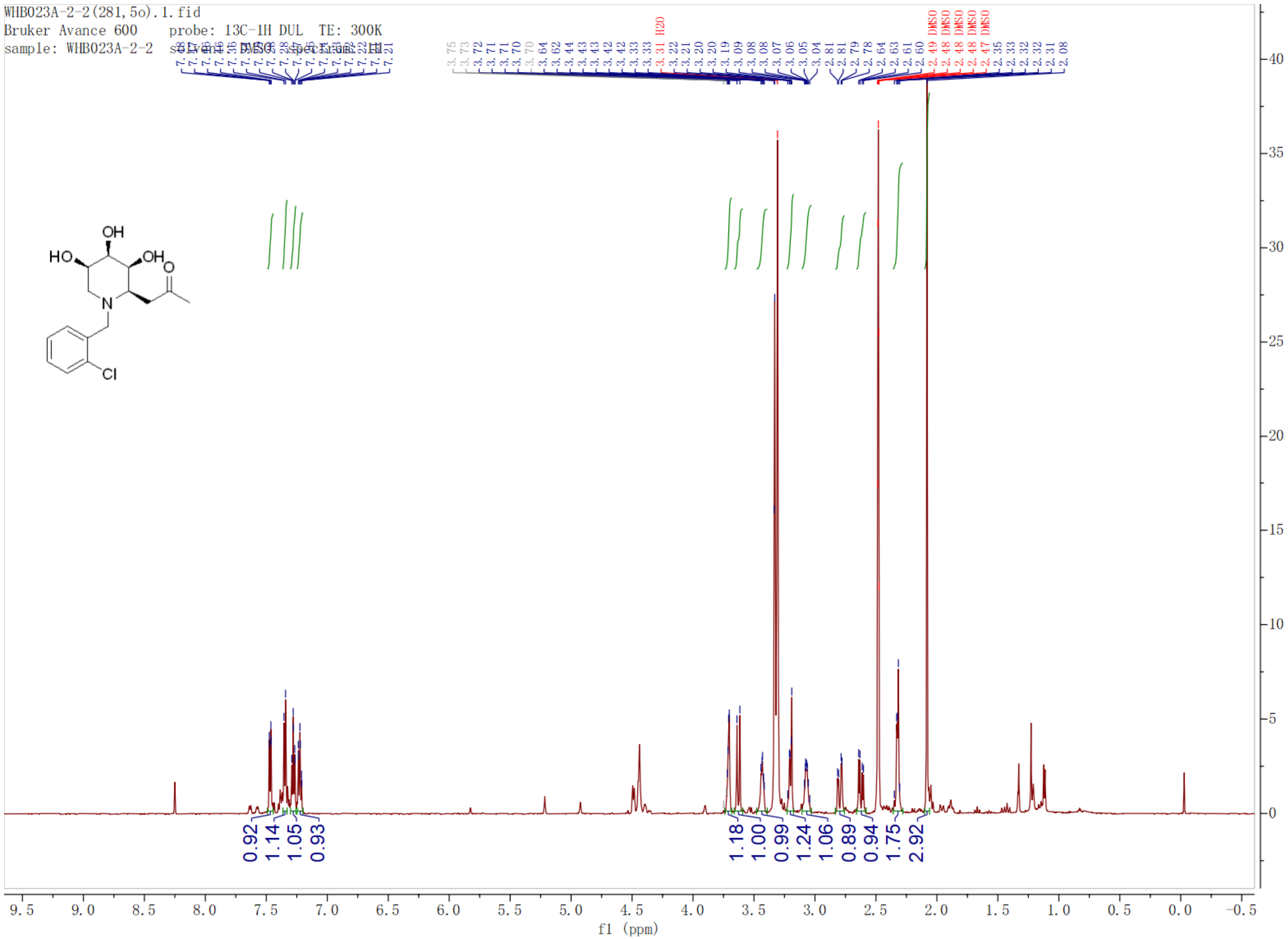

Figure S68. The ¹H NMR of compound **5r**.

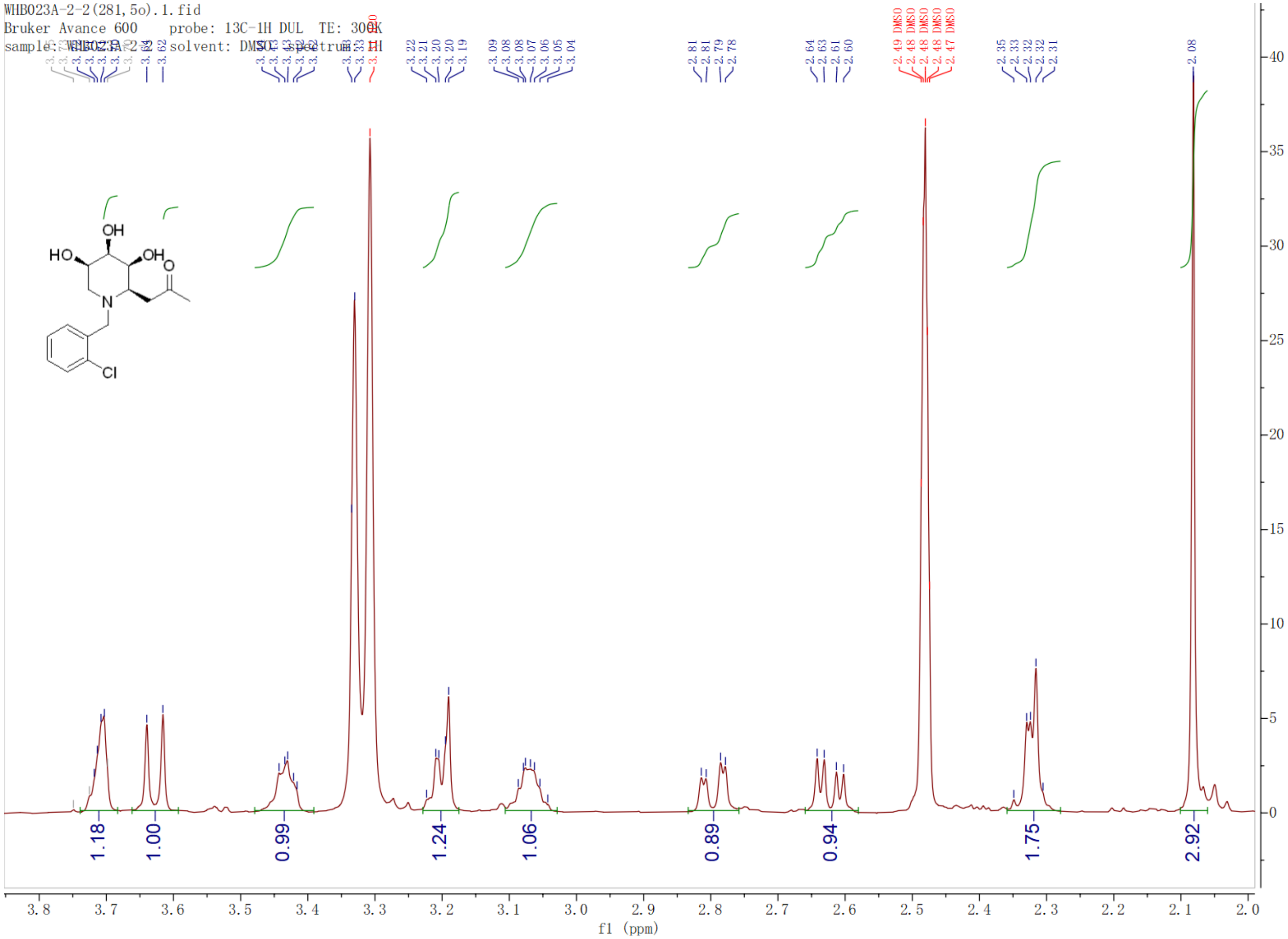

Figure S69. Partial region amplification of the ^1H NMR of compound **5r**.

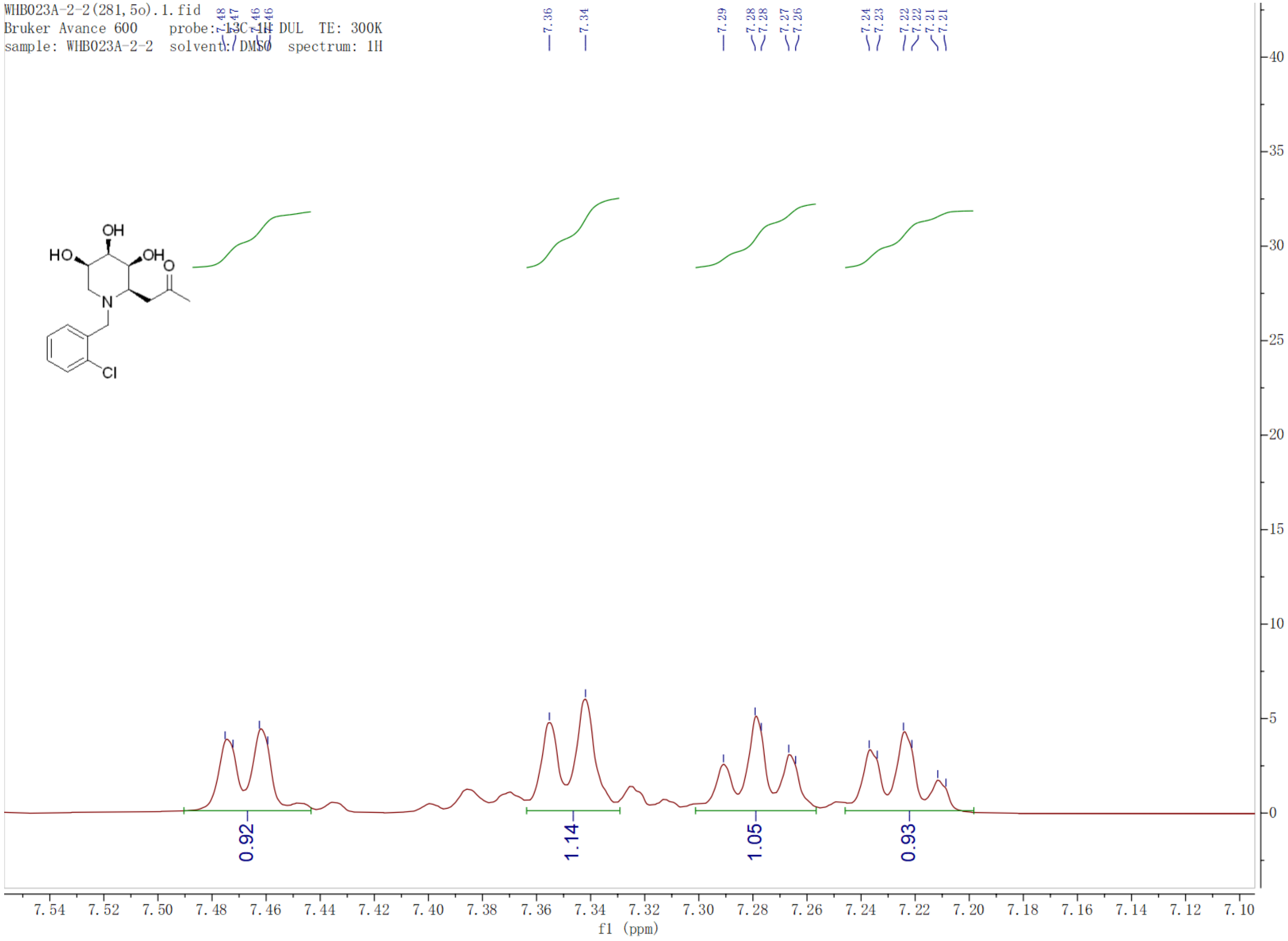

Figure S70. Partial region amplification of the ^{1}H NMR of compound **5r**.

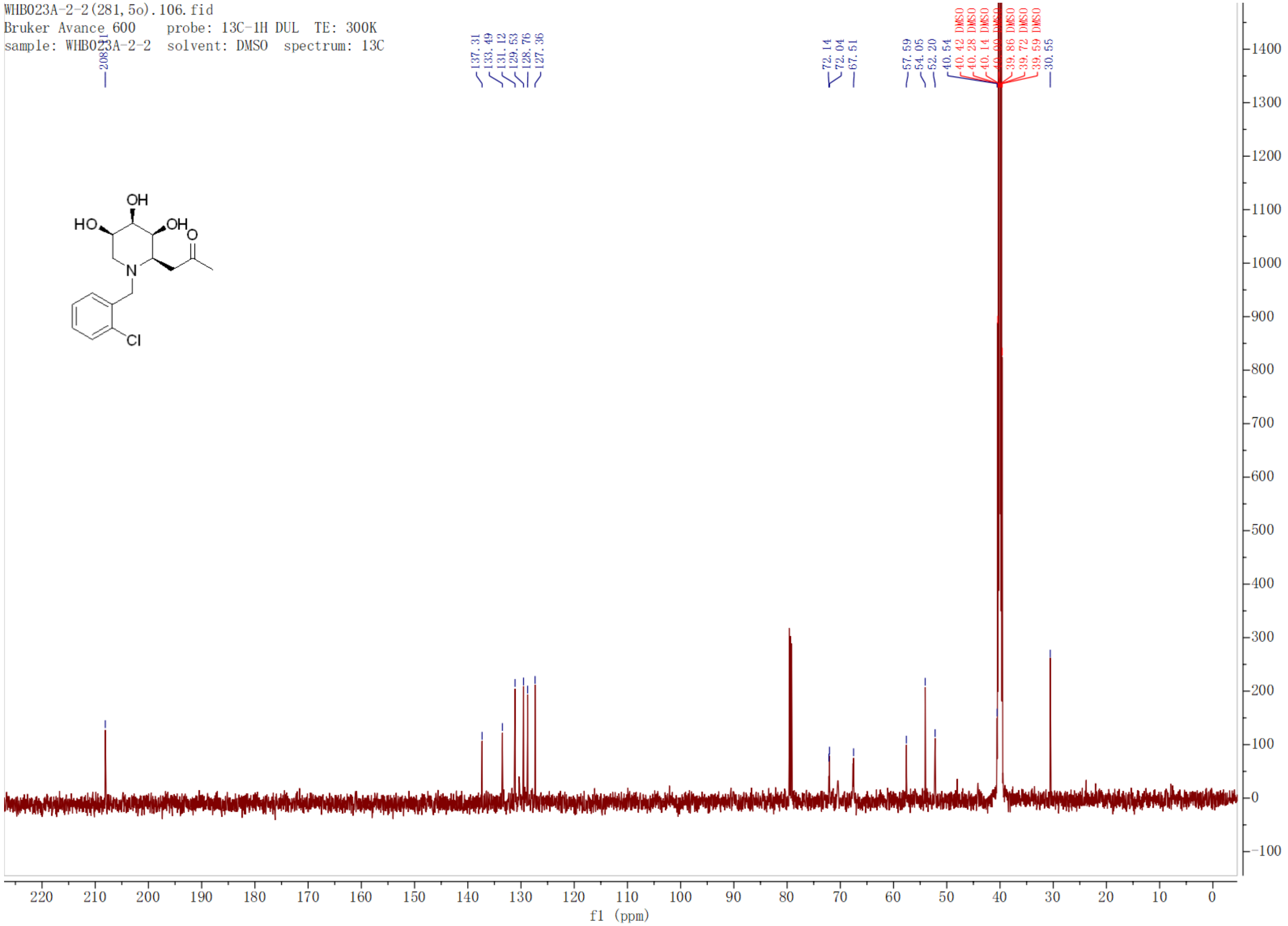

Figure S71. The ^{13}C NMR of compound **5r**.

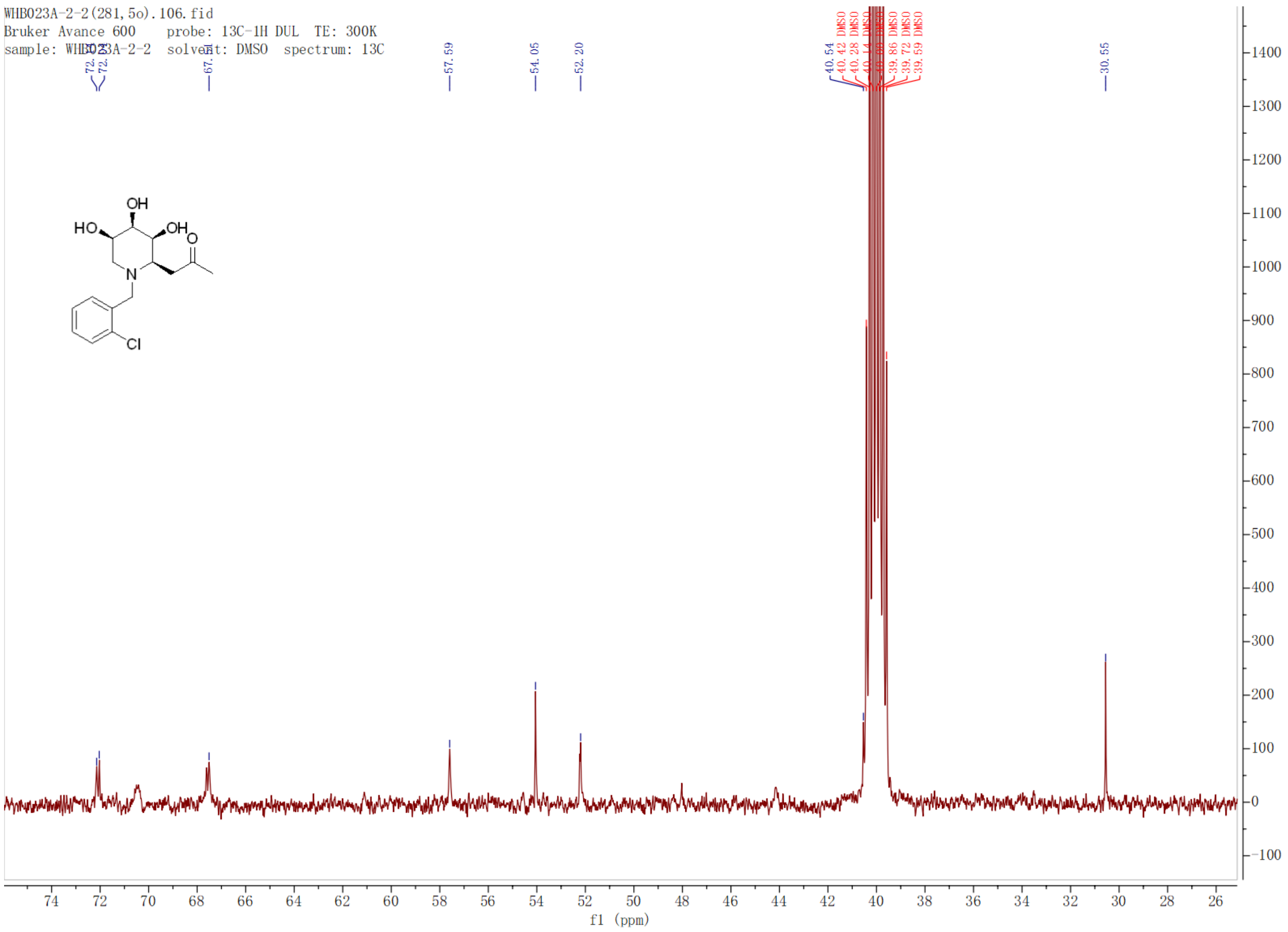

Figure S72. Partial region amplification of the ^{13}C NMR of compound **5r**.

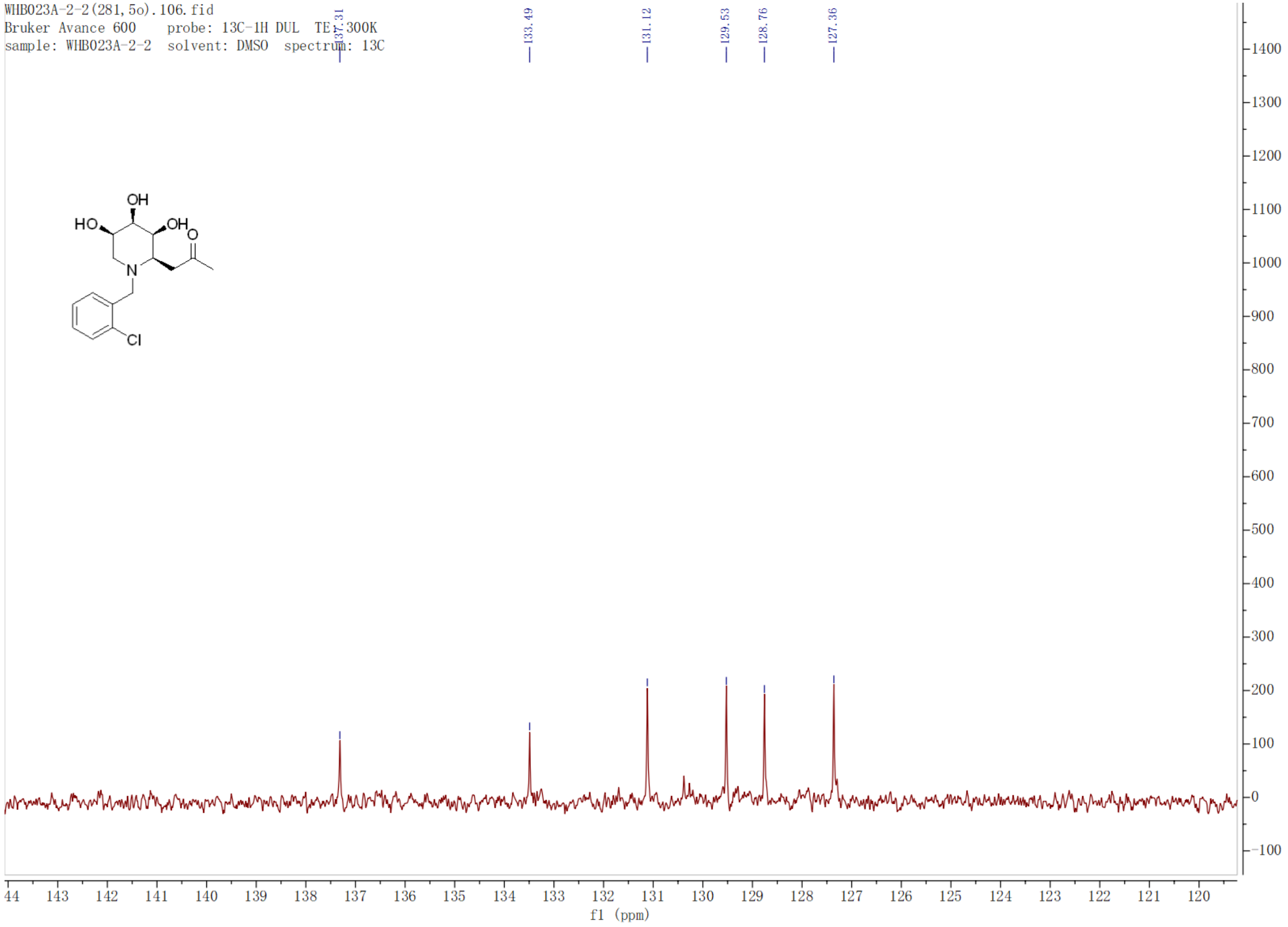

Figure S73. Partial region amplification of the ^{13}C NMR of compound **5r**.

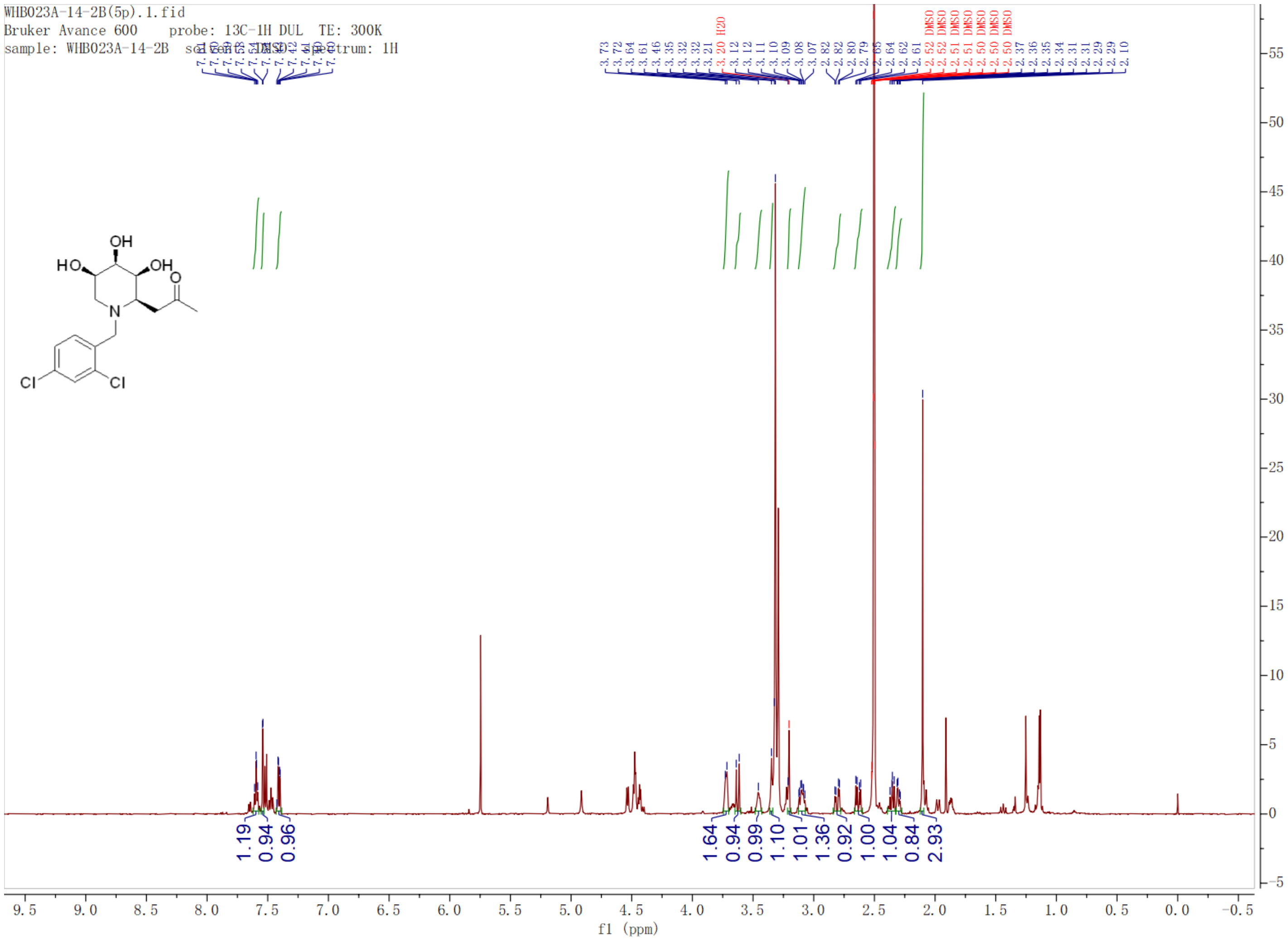

Figure S74. The ^{1}H NMR of compound **5s**.

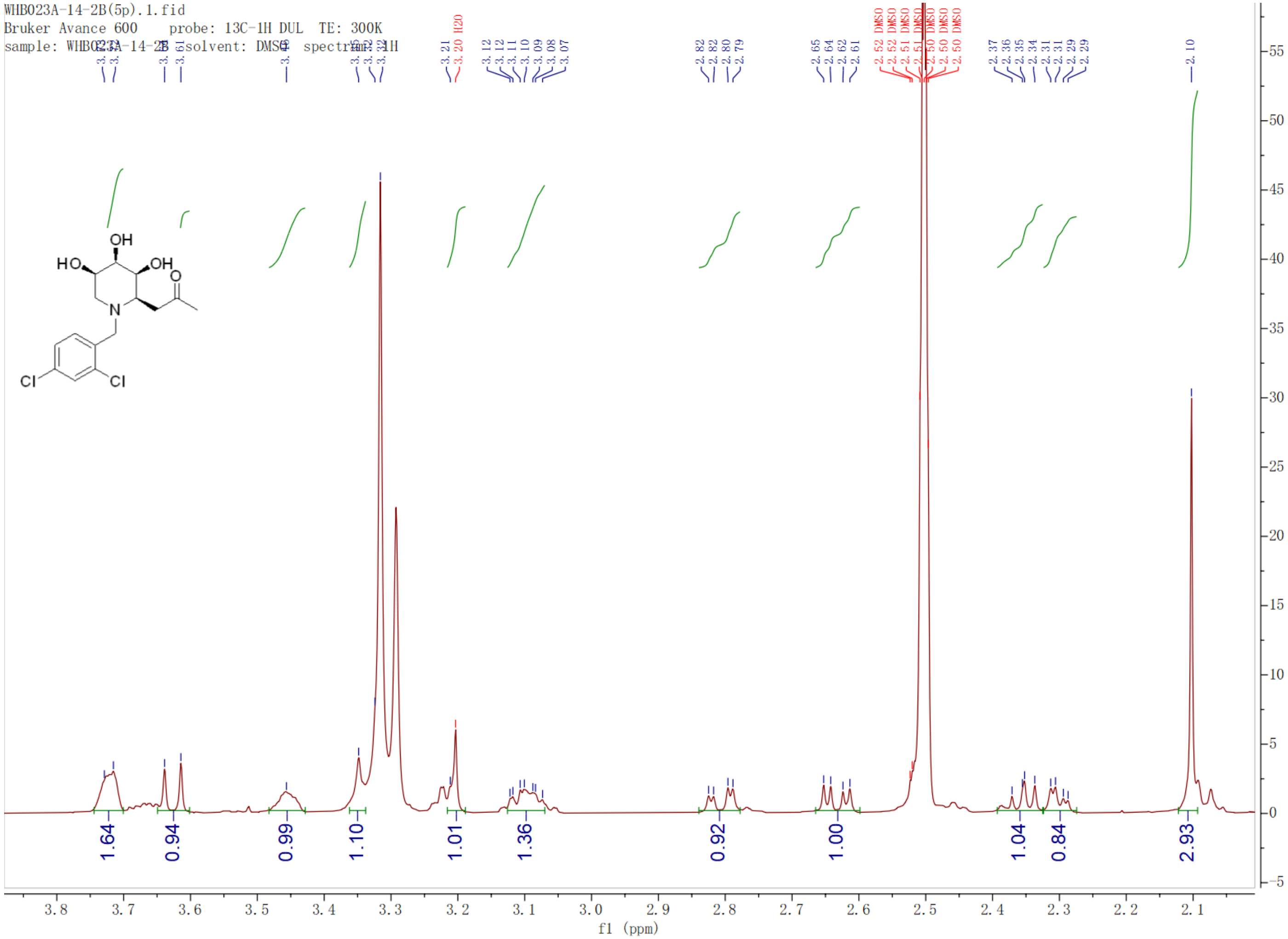

Figure S75. Partial region amplification of the ^{1}H NMR of compound **5s**.

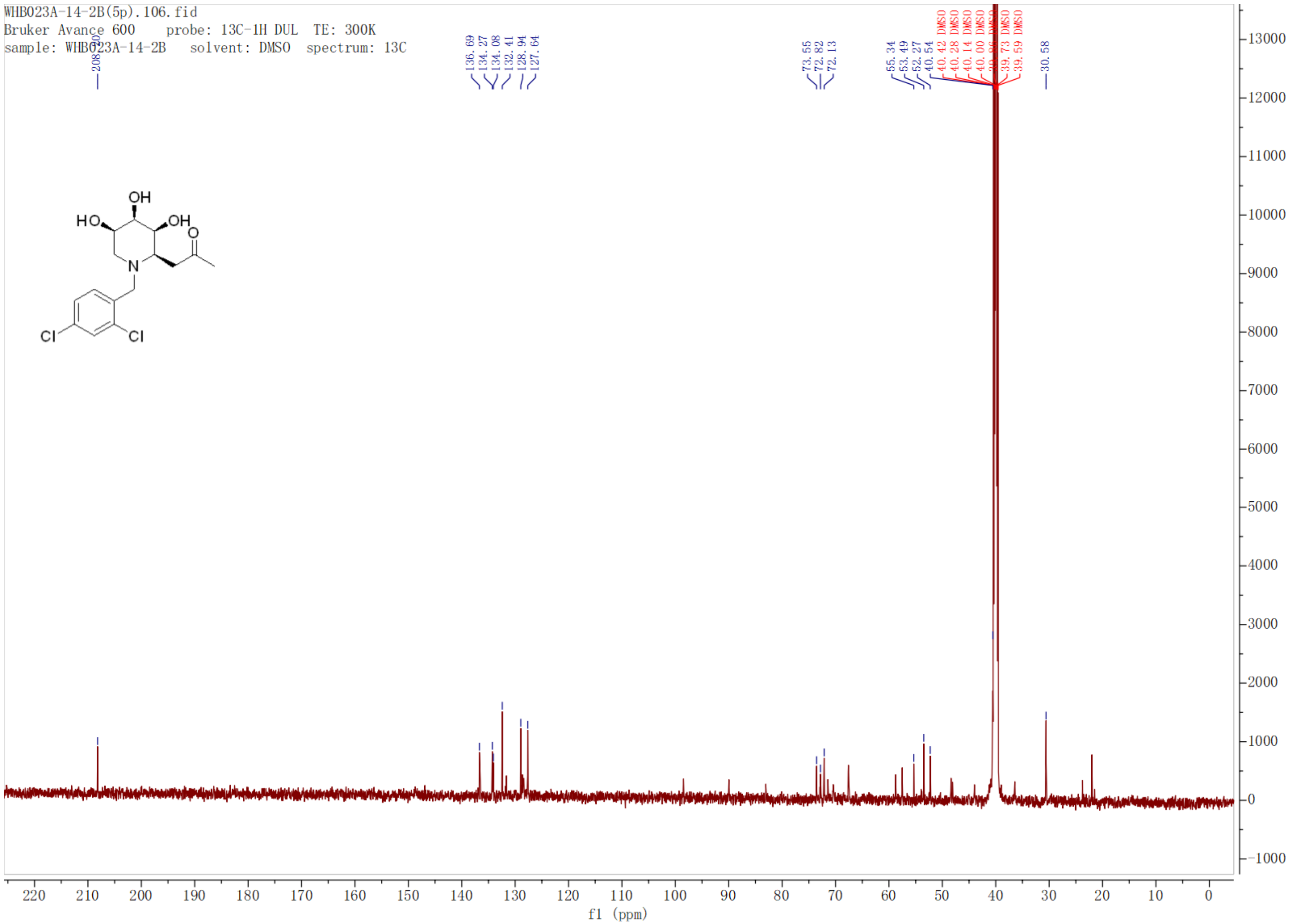

Figure S76. The ^{13}C NMR of compound **5s**.

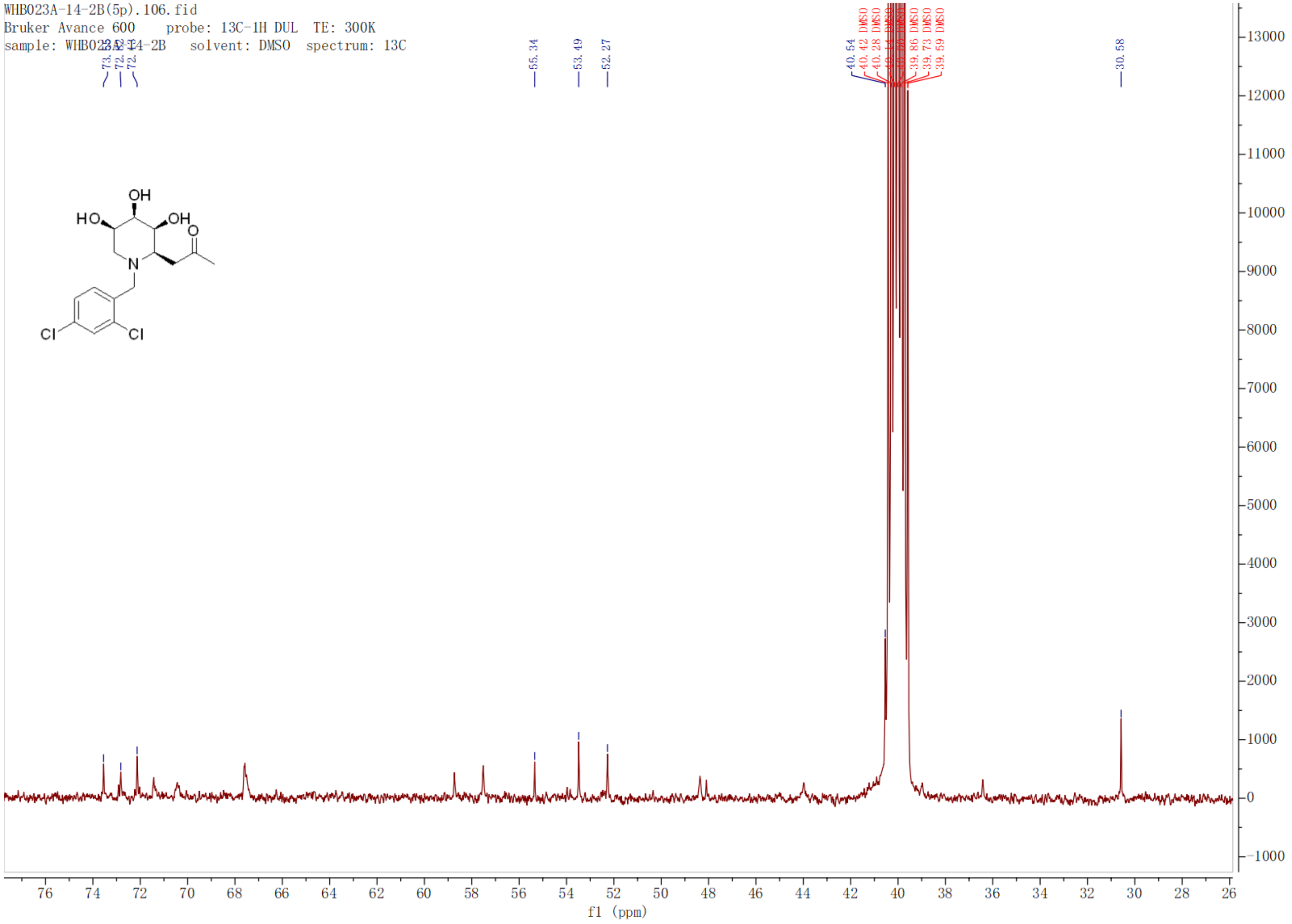

Figure S77. Partial region amplification of the ^{13}C NMR of compound **5s**.

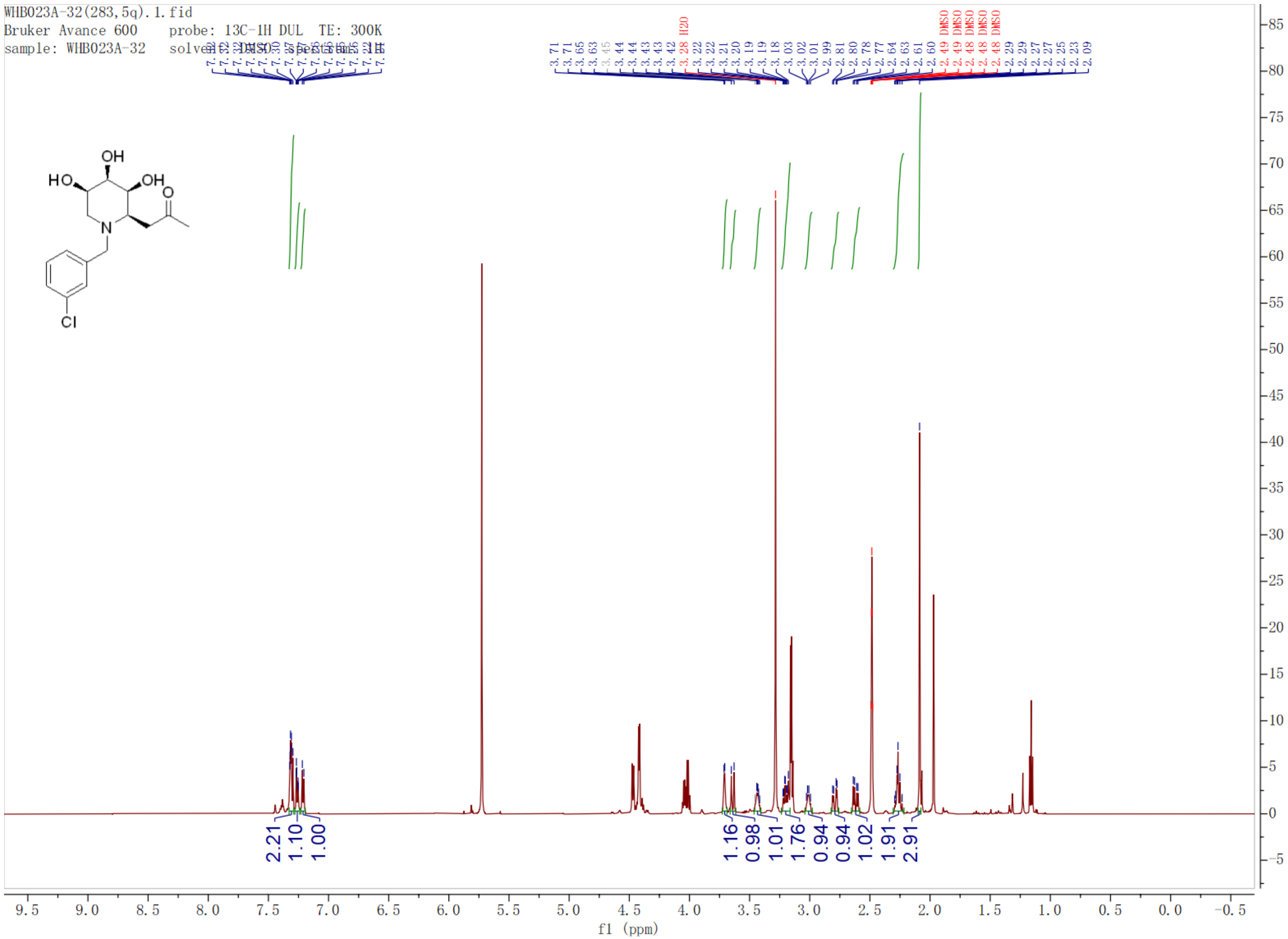

Figure S78. The ^{1}H NMR of compound **5t**.

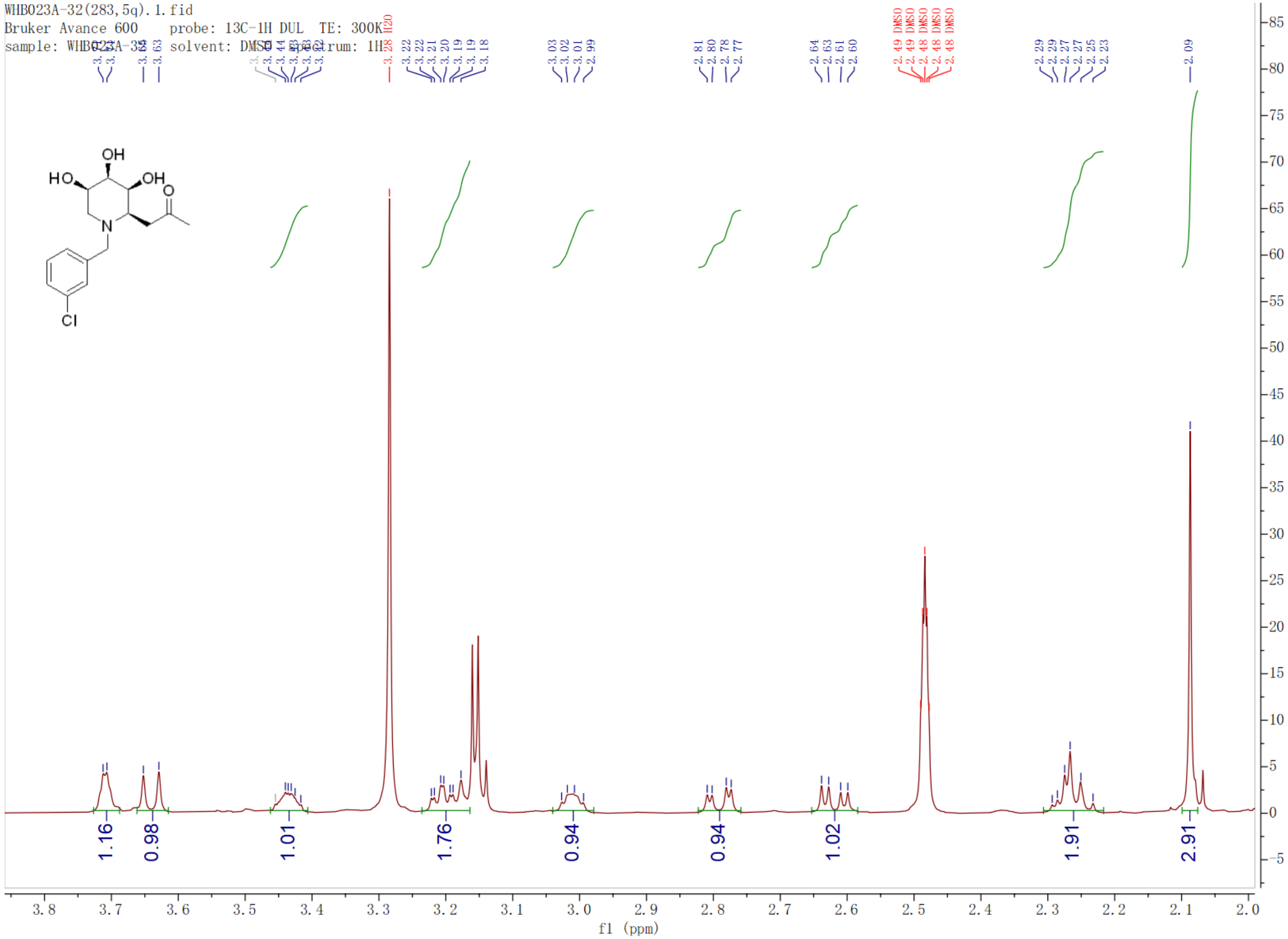

Figure S79. Partial region amplification of the ^{1}H NMR of compound **5t**.

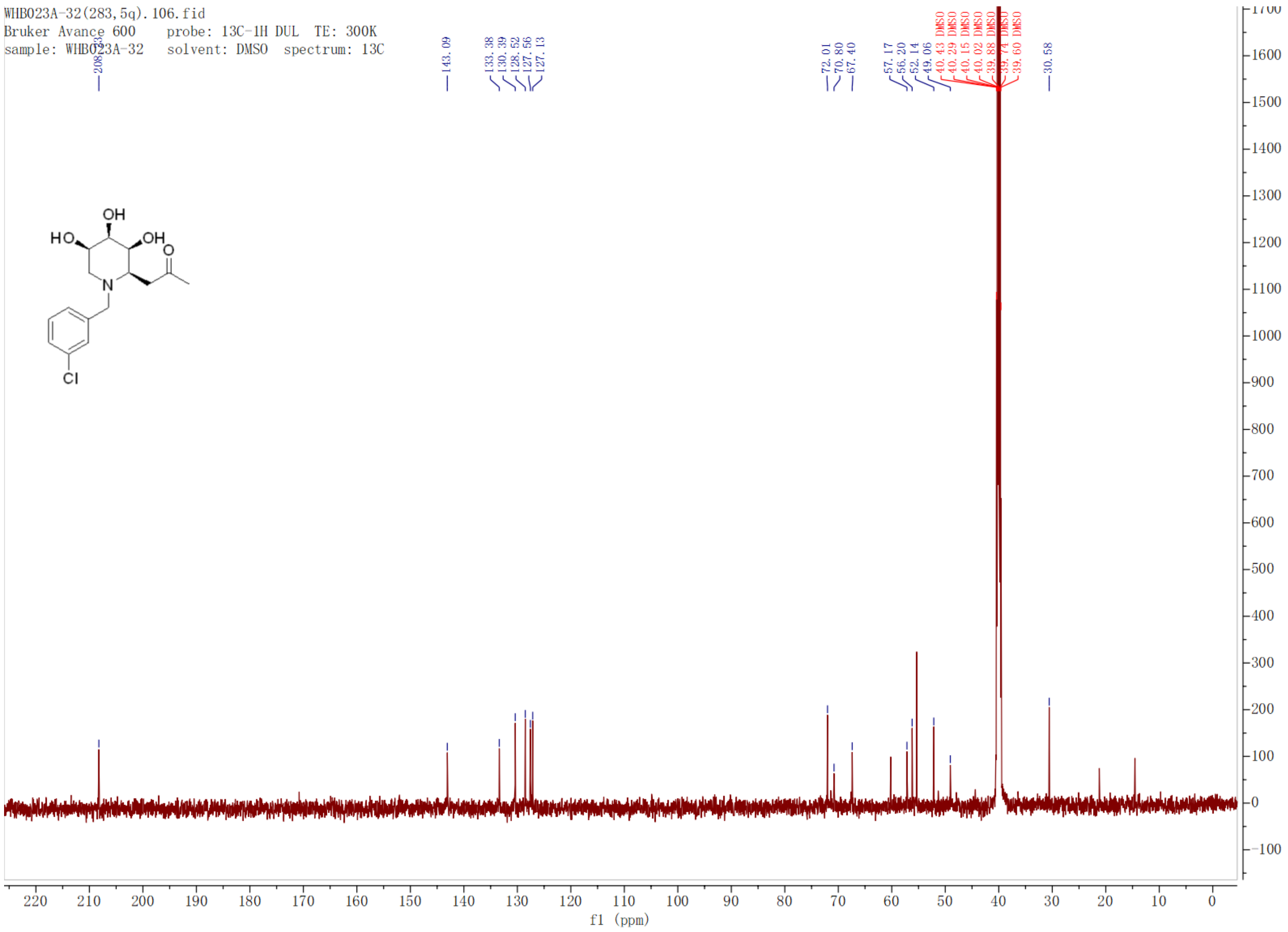

Figure S80. The ^{13}C NMR of compound **5t**.

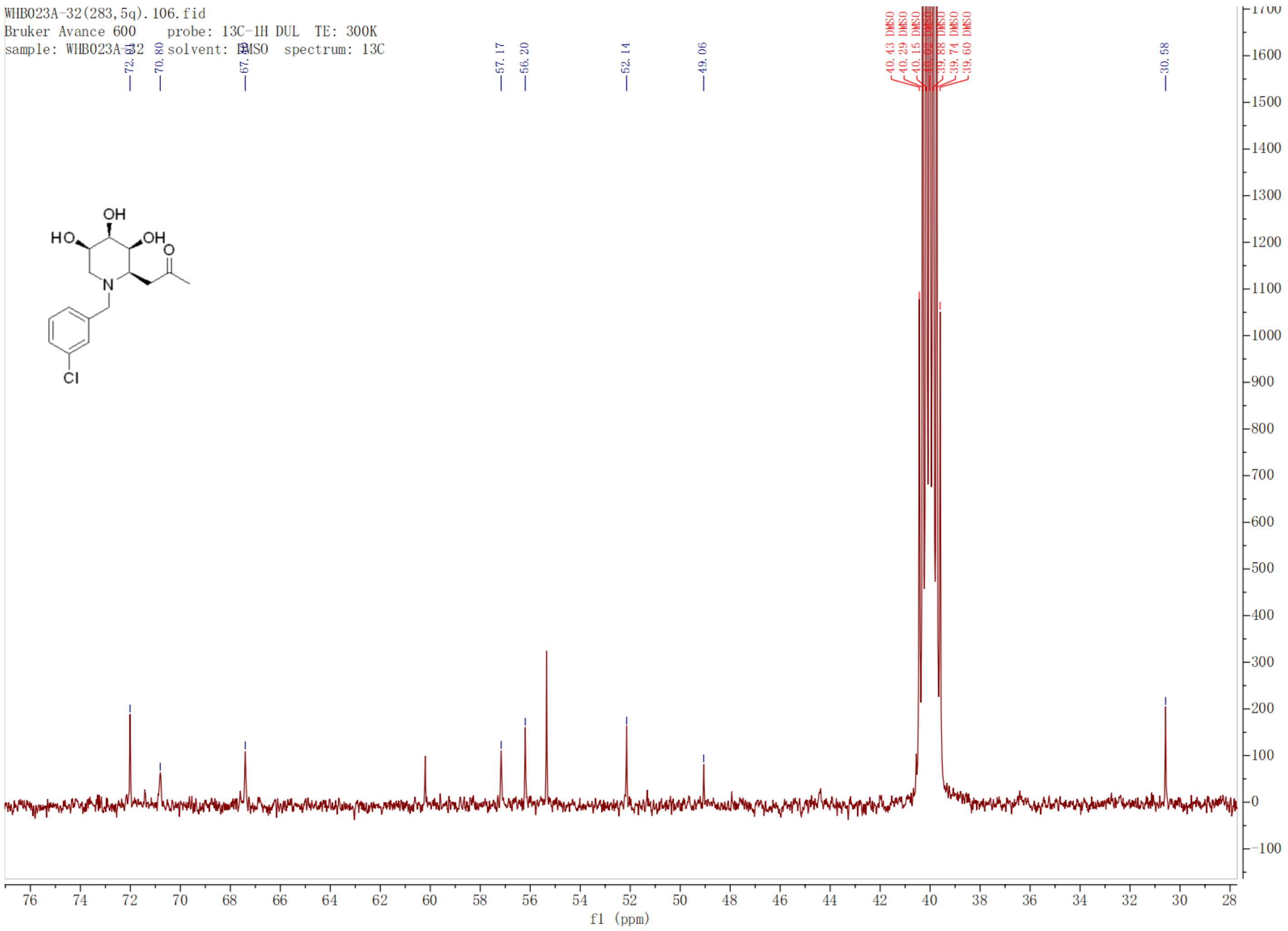

Figure S81. Partial region amplification of the ^{13}C NMR of compound **5t**.

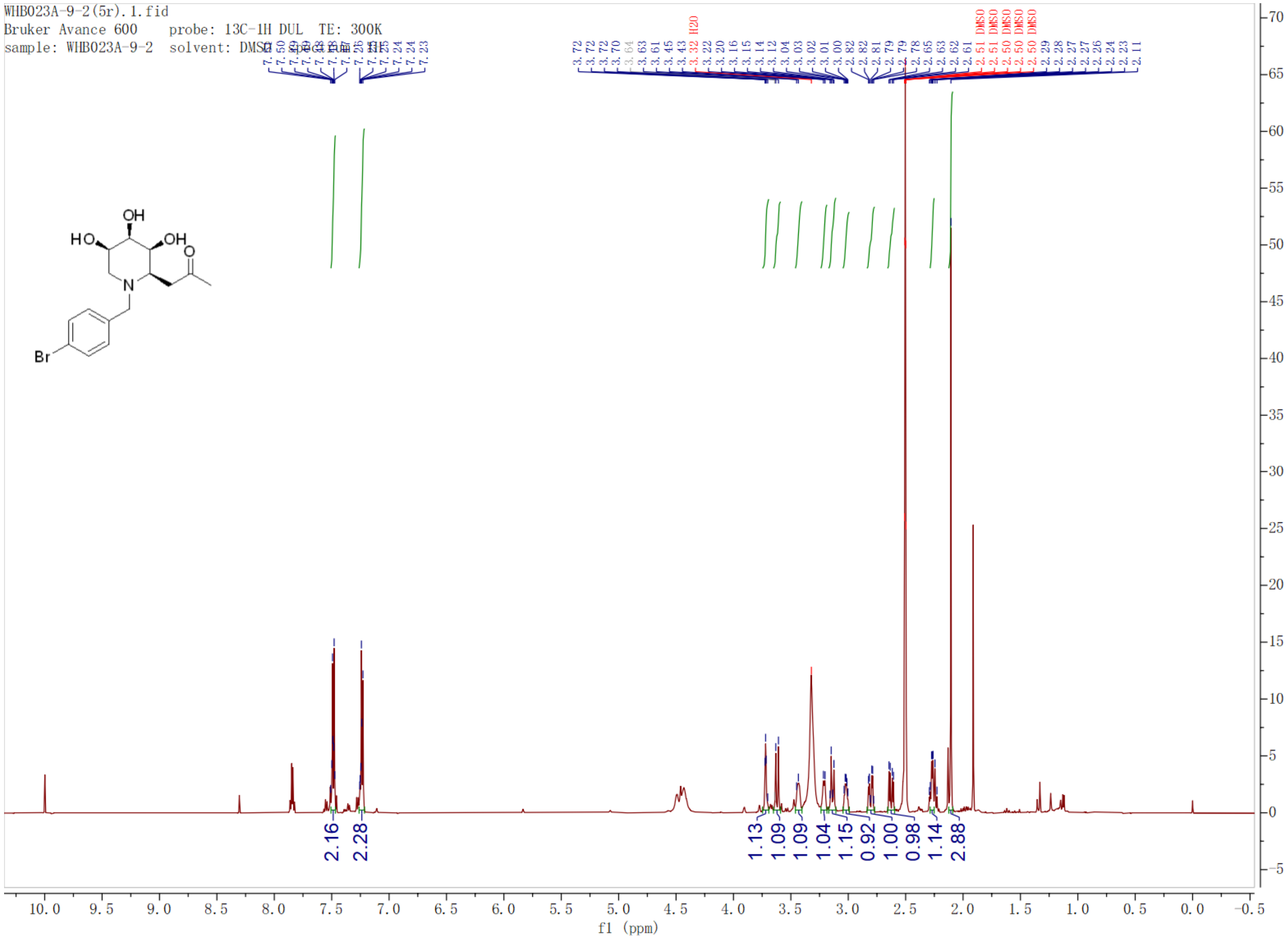

Figure S82. The ¹H NMR of compound **5u**.

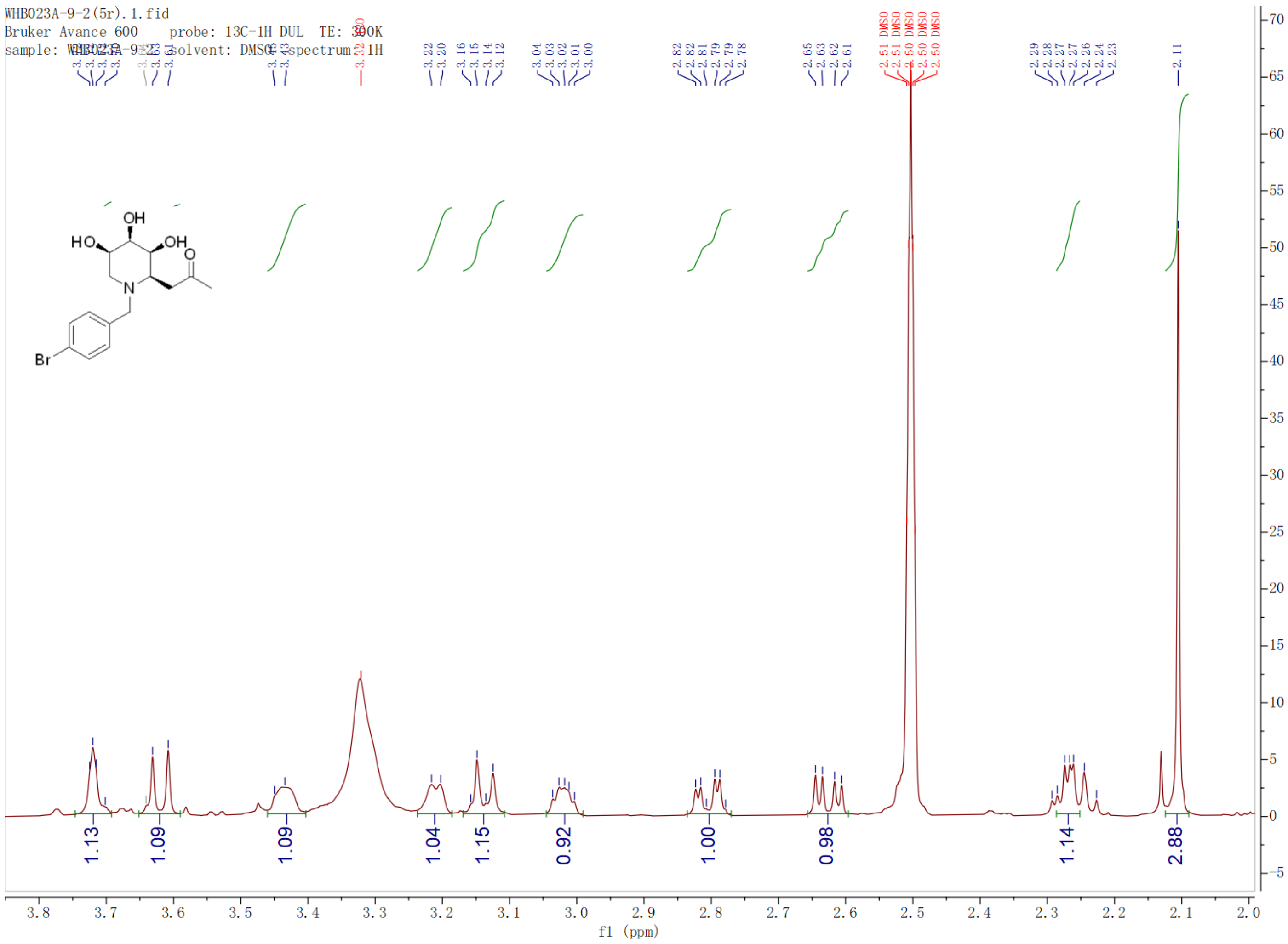

Figure S83. Partial region amplification of the ^{1}H NMR of compound **5u**.

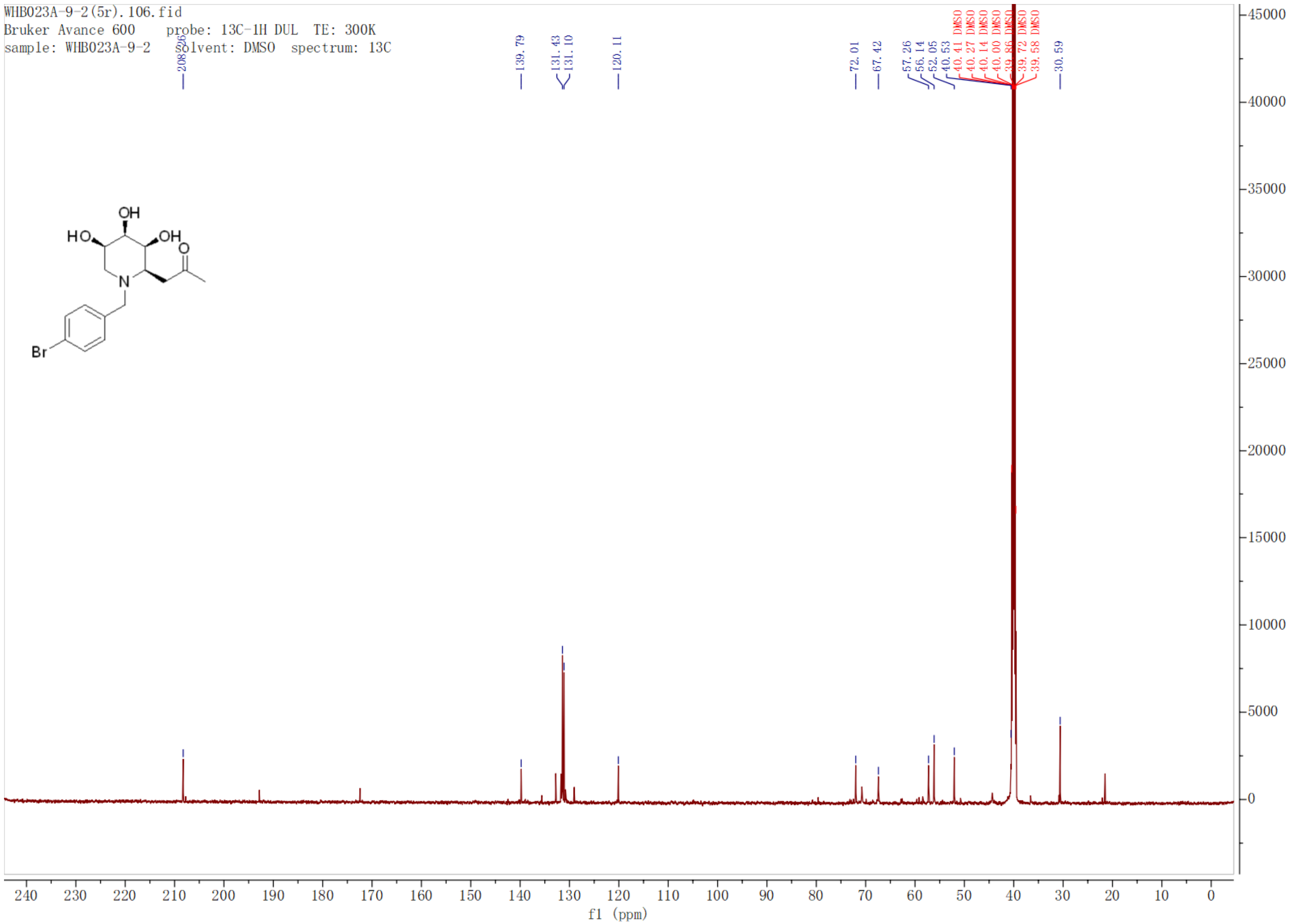

Figure S84. The ^{13}C NMR of compound **5u**.

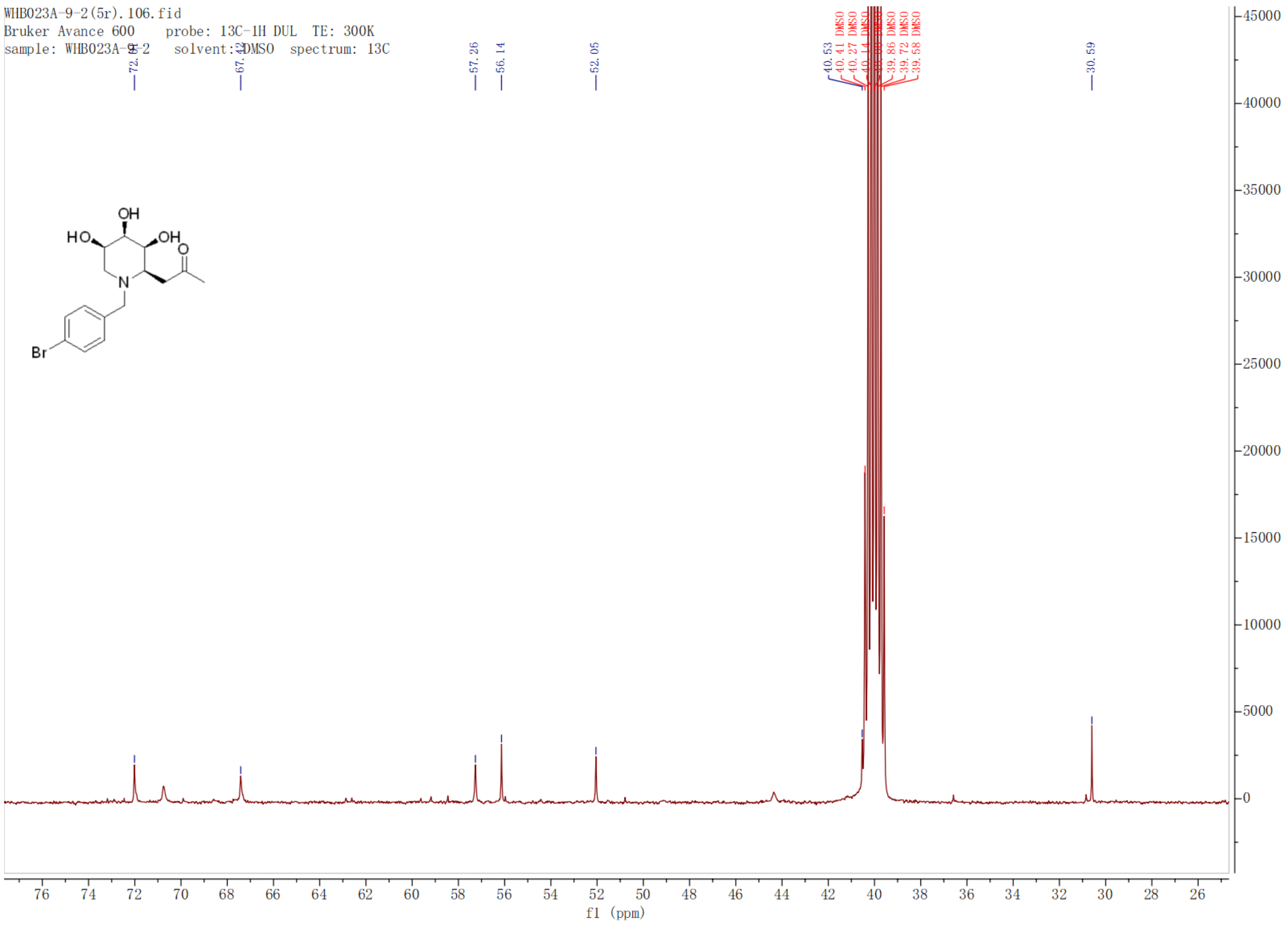

Figure S85. Partial region amplification of the ^{13}C NMR of compound **5u**.

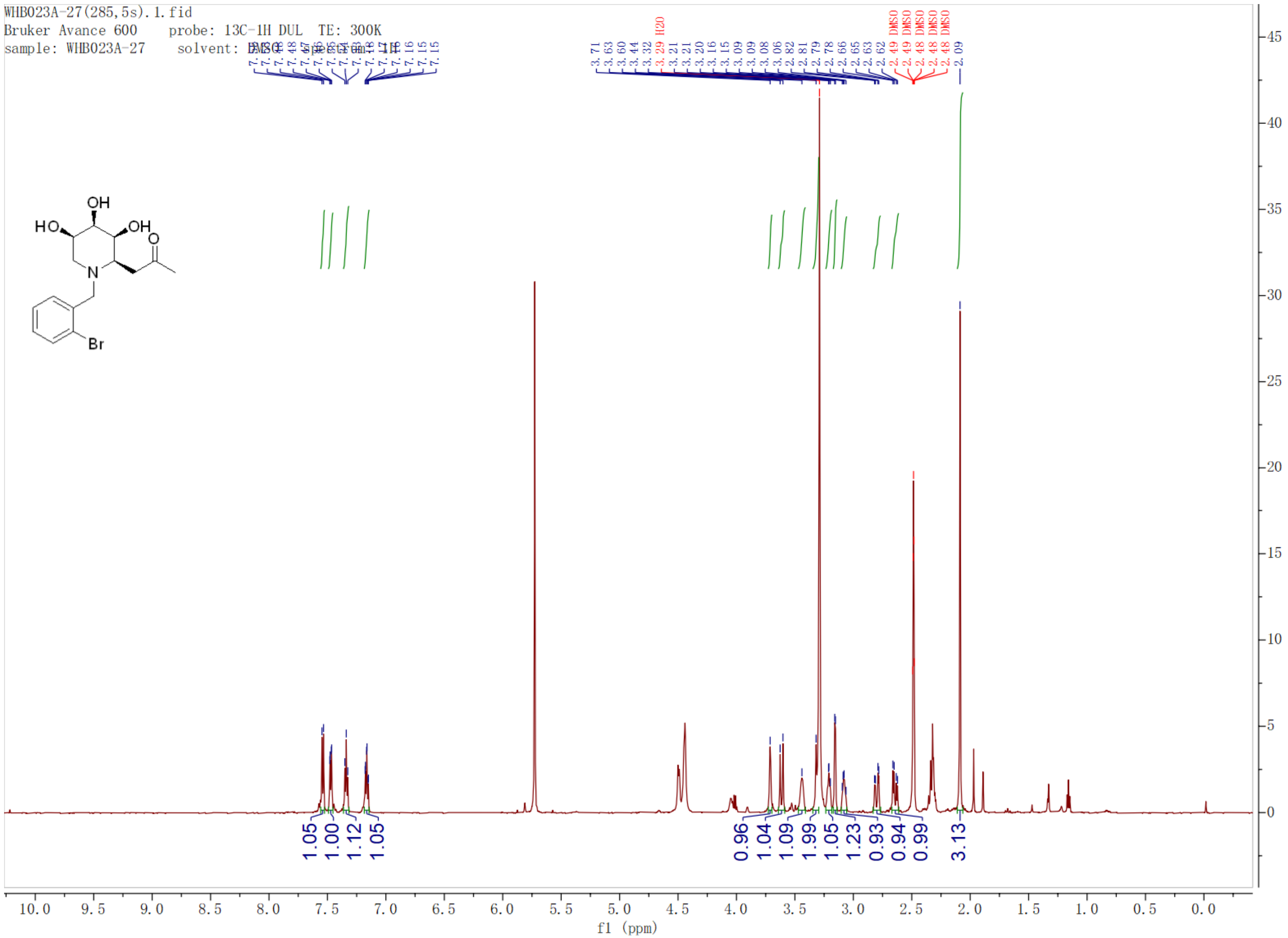

Figure S86. The ^{1}H NMR of compound **5v**.

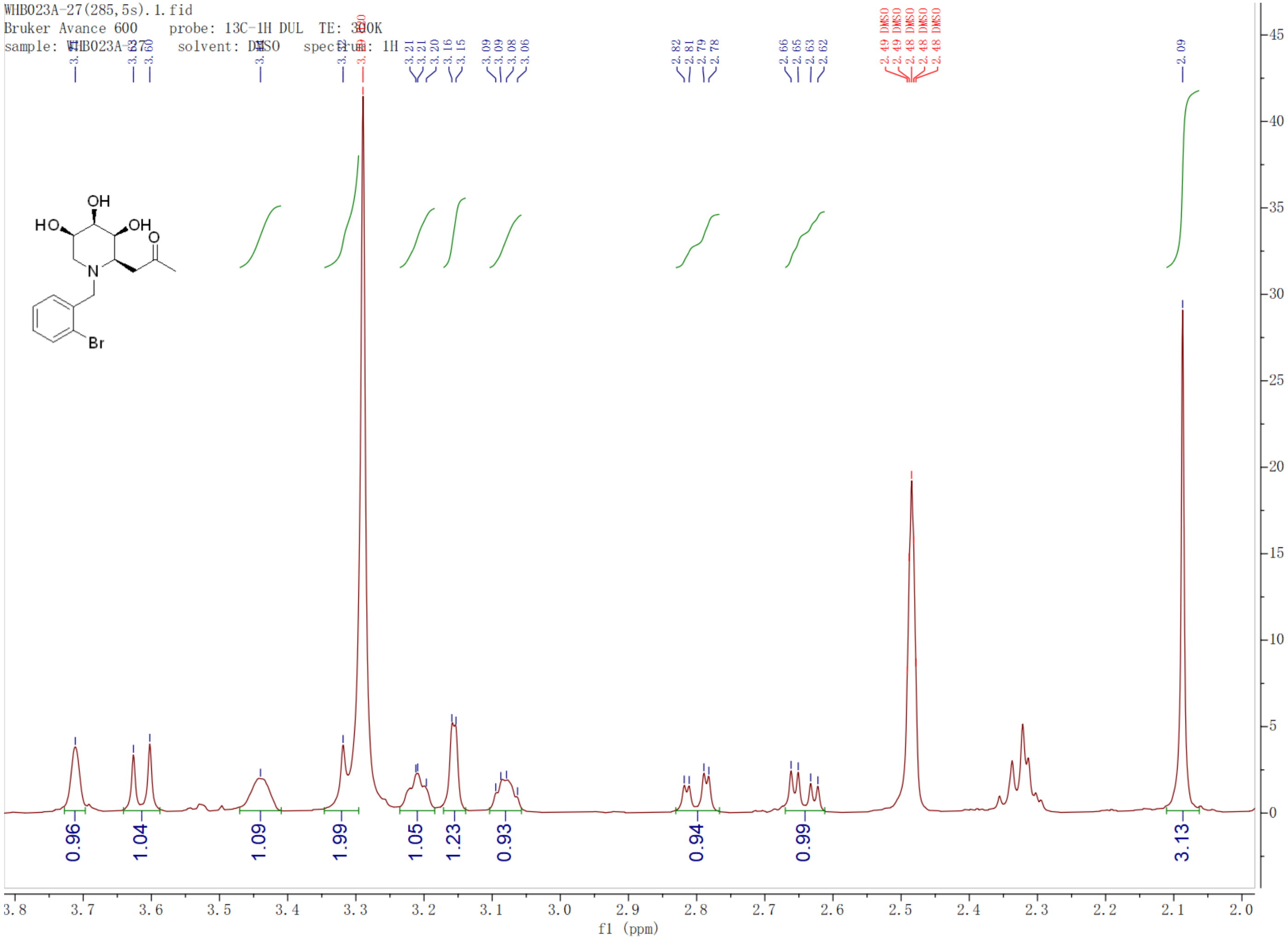

Figure S87. Partial region amplification of the ^{1}H NMR of compound **5v**.

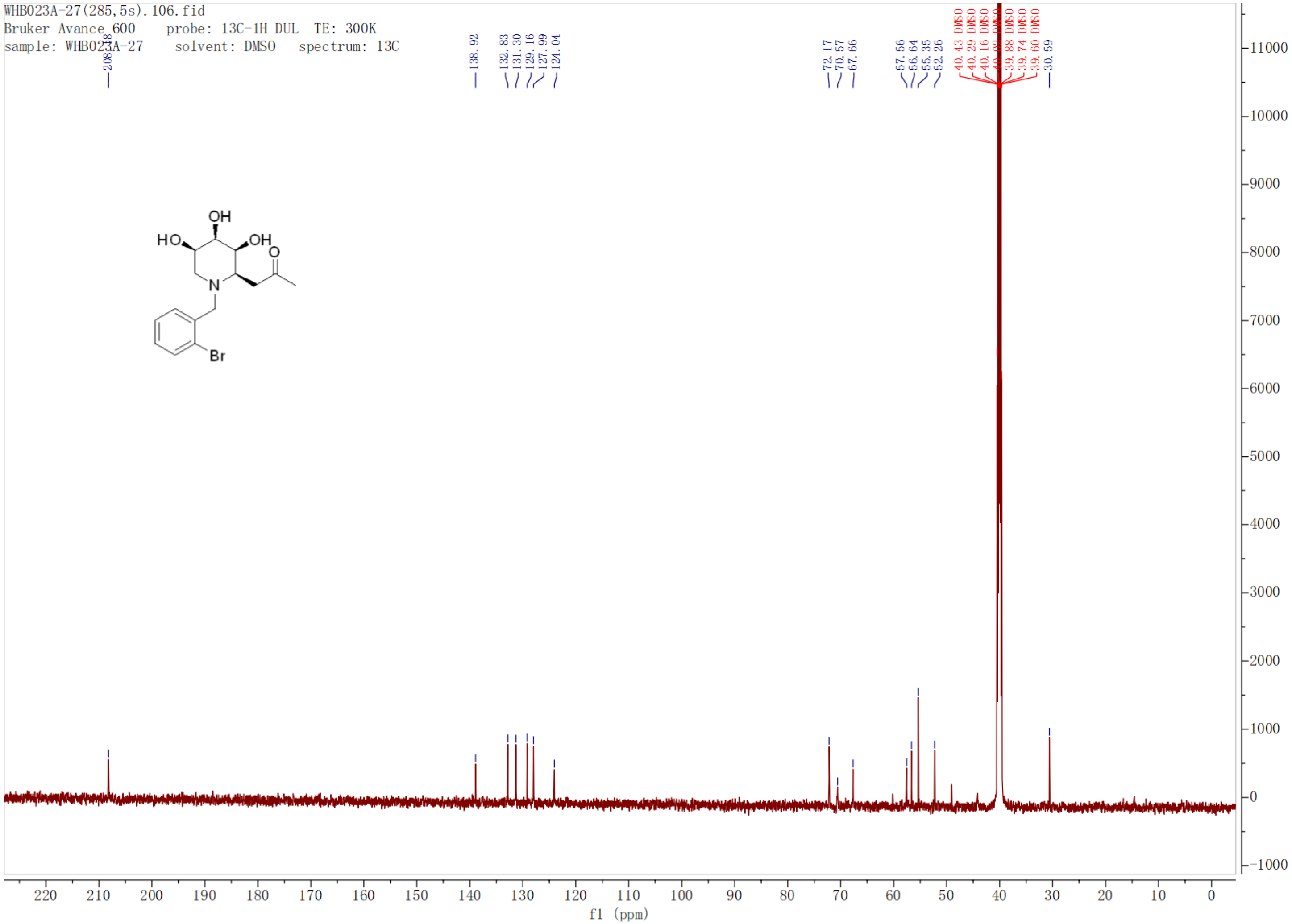

Figure S88. The ^{13}C NMR of compound **5v**.

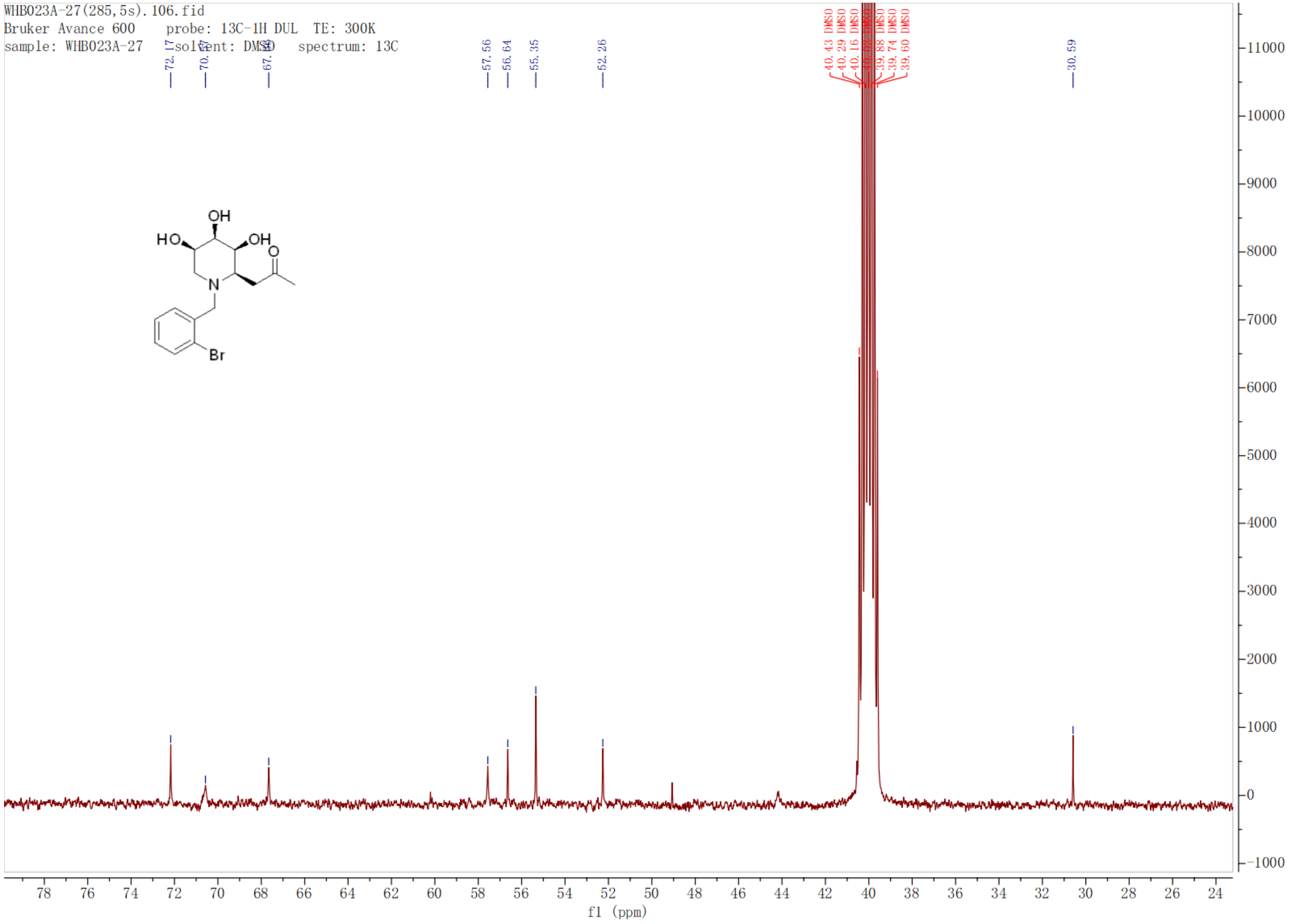

Figure S89. Partial region amplification of the ^{13}C NMR of compound **5v**.

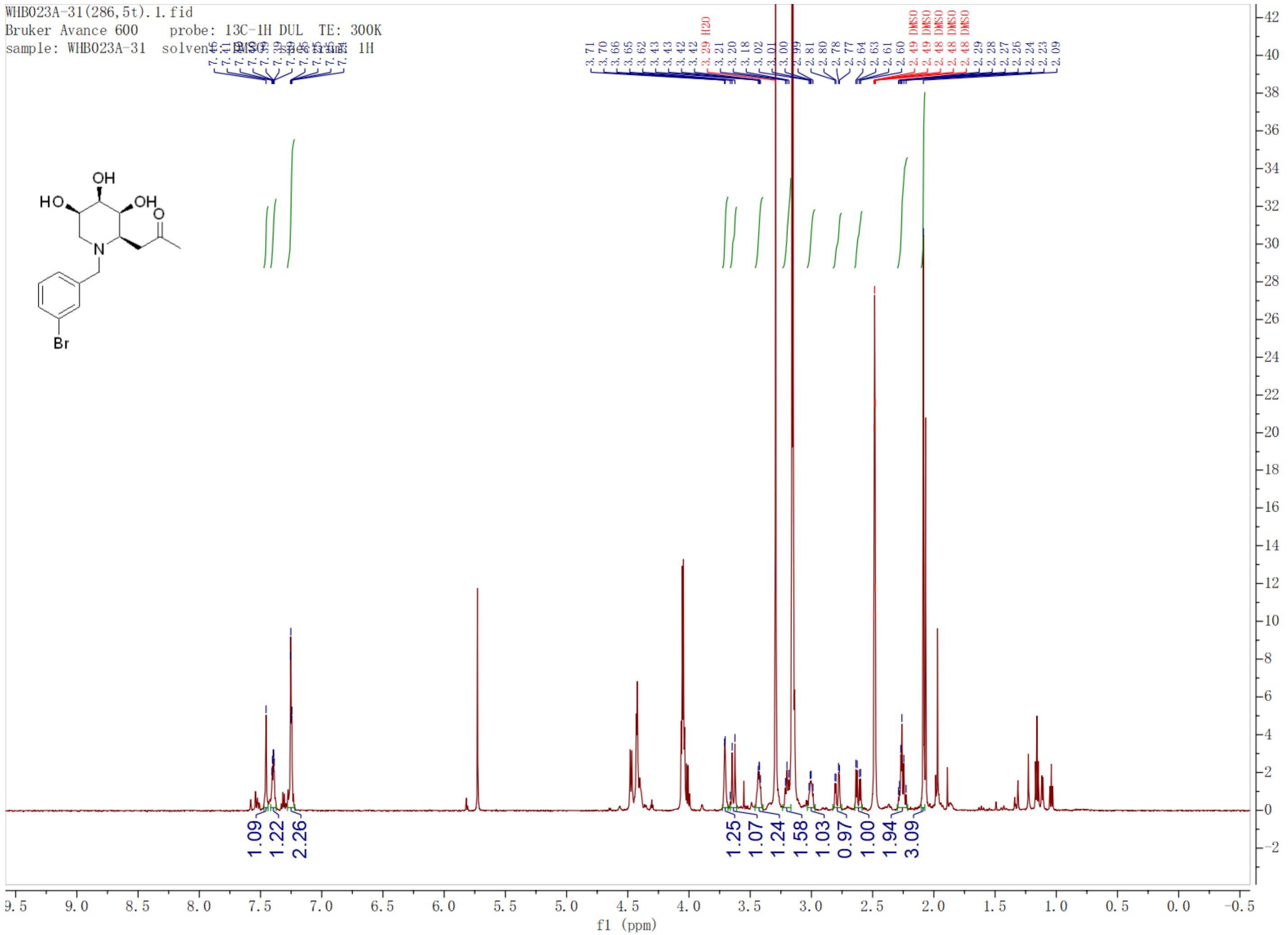

Figure S90. The ^{1}H NMR of compound **5w**.

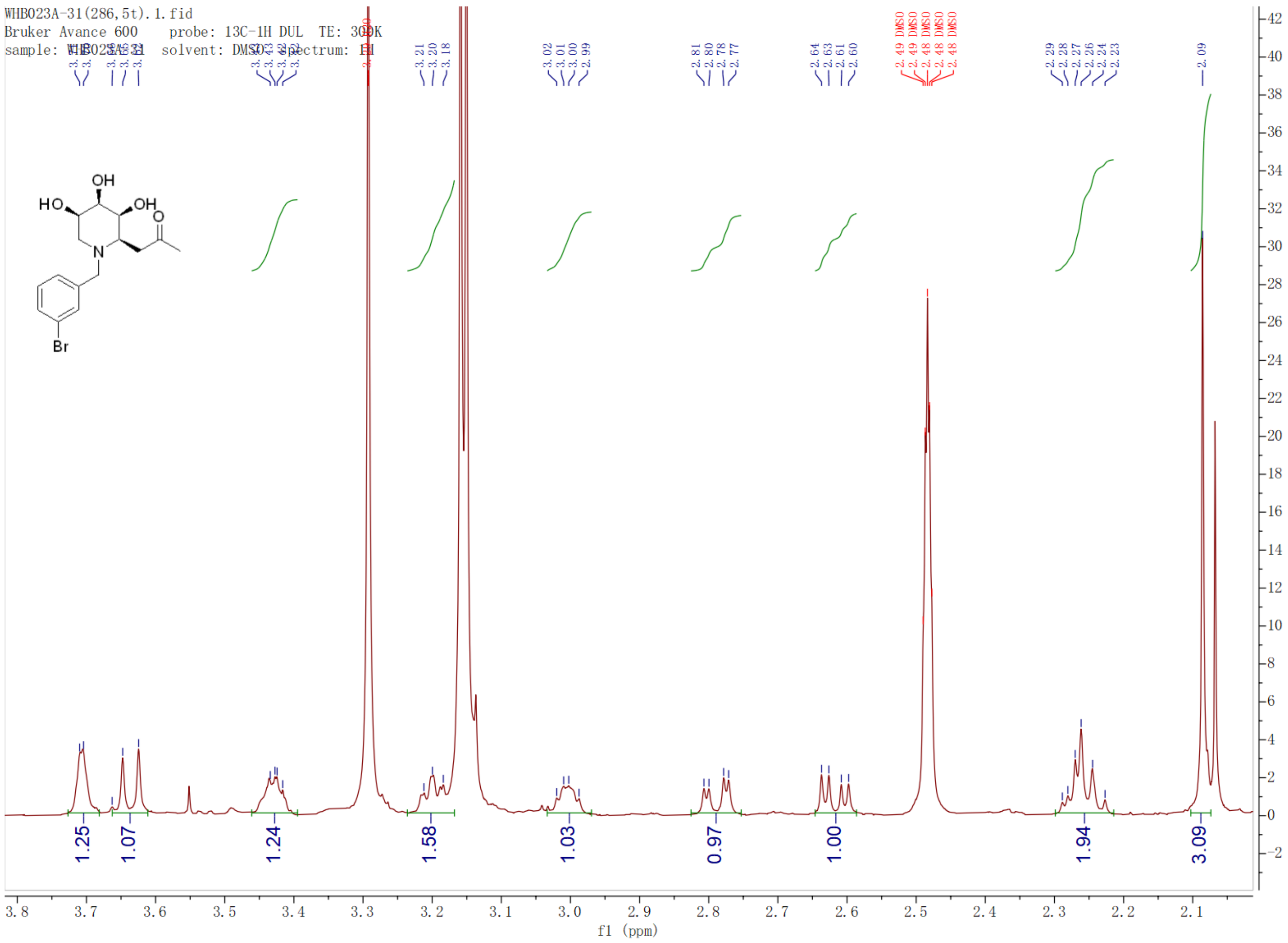

Figure S91. Partial region amplification of the ^{1}H NMR of compound **5w**.

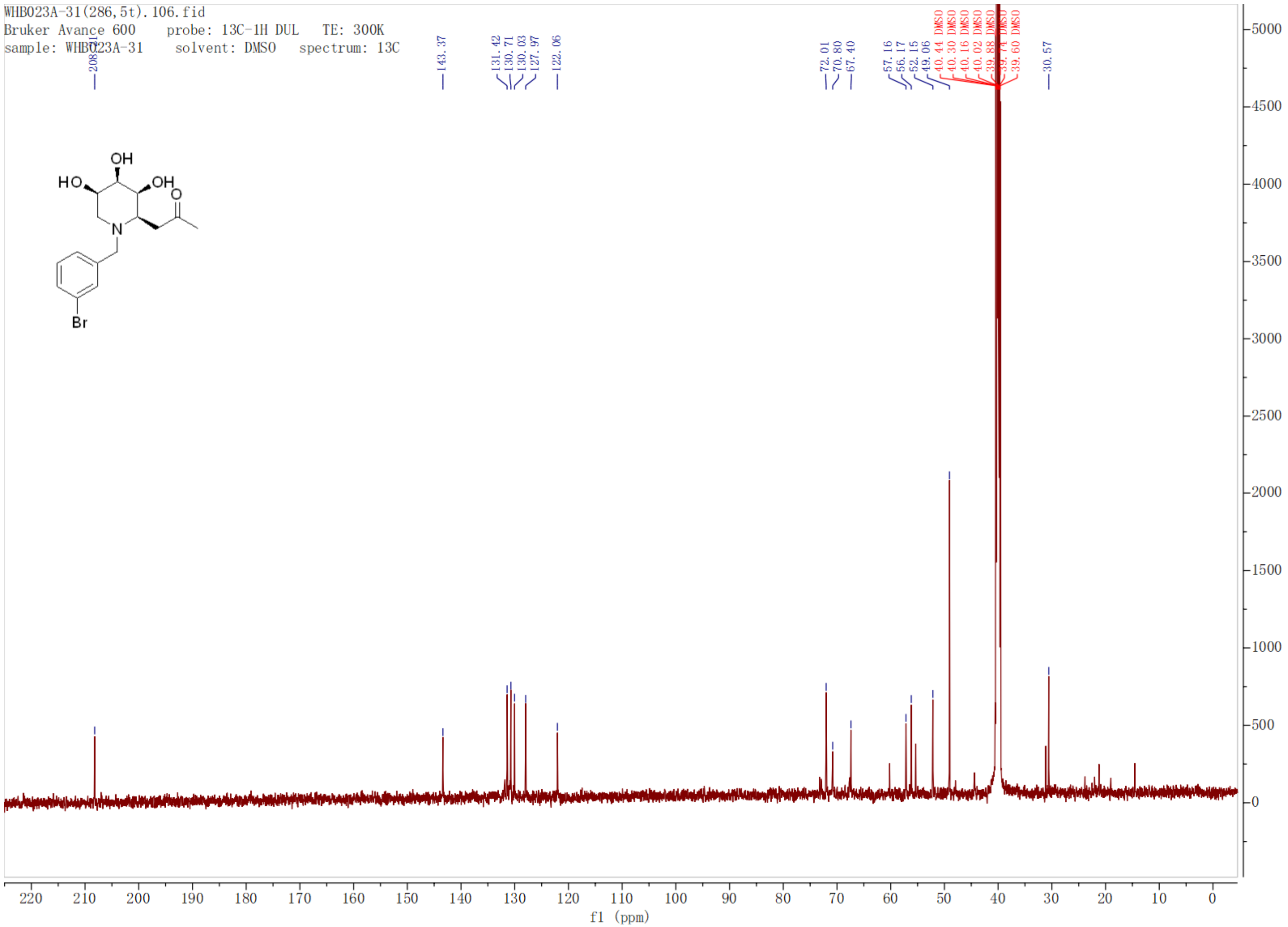

Figure S92. The ^{13}C NMR of compound **5w**.

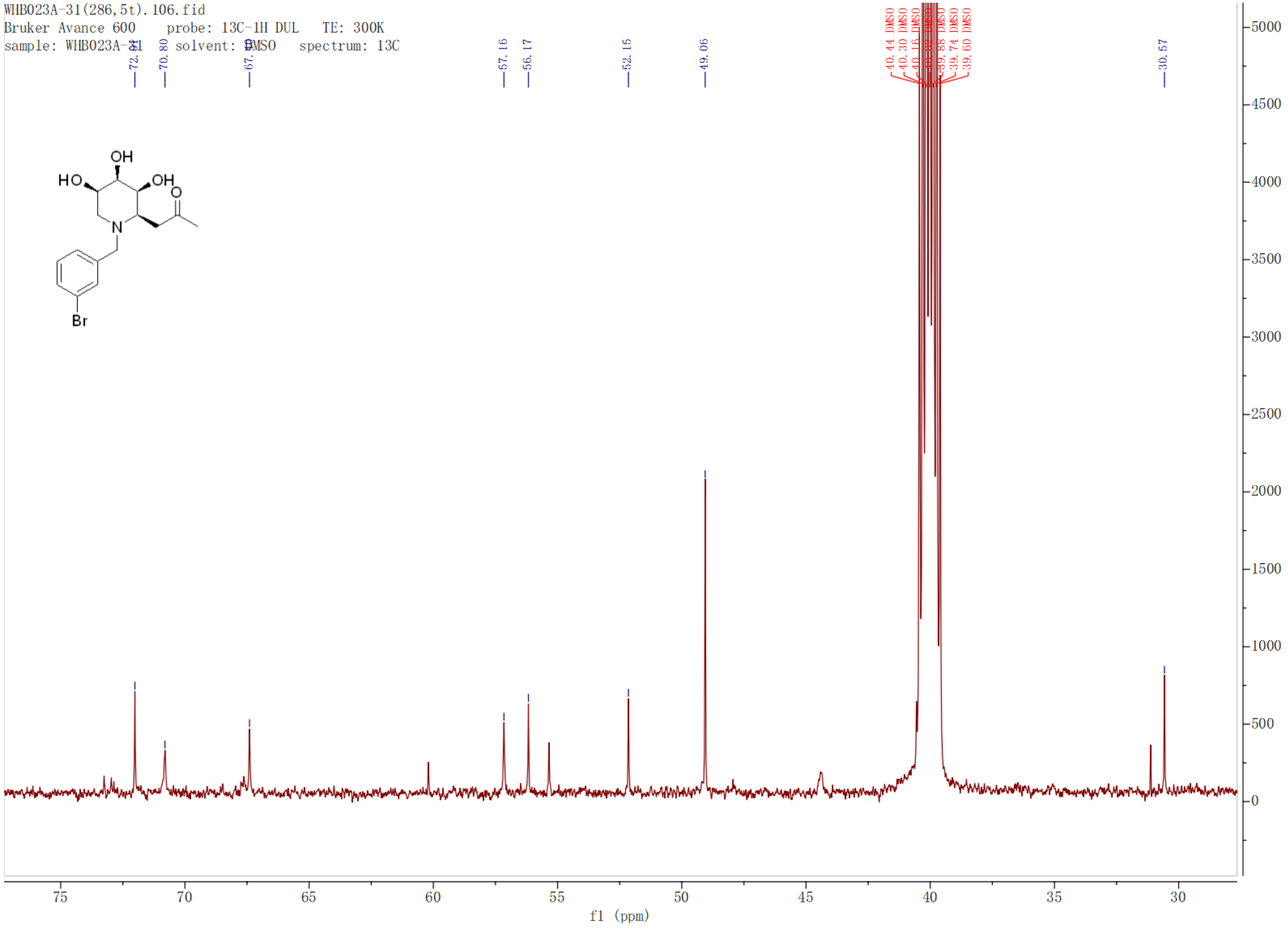

Figure S93. Partial region amplification of the ^{13}C NMR of compound **5w**.

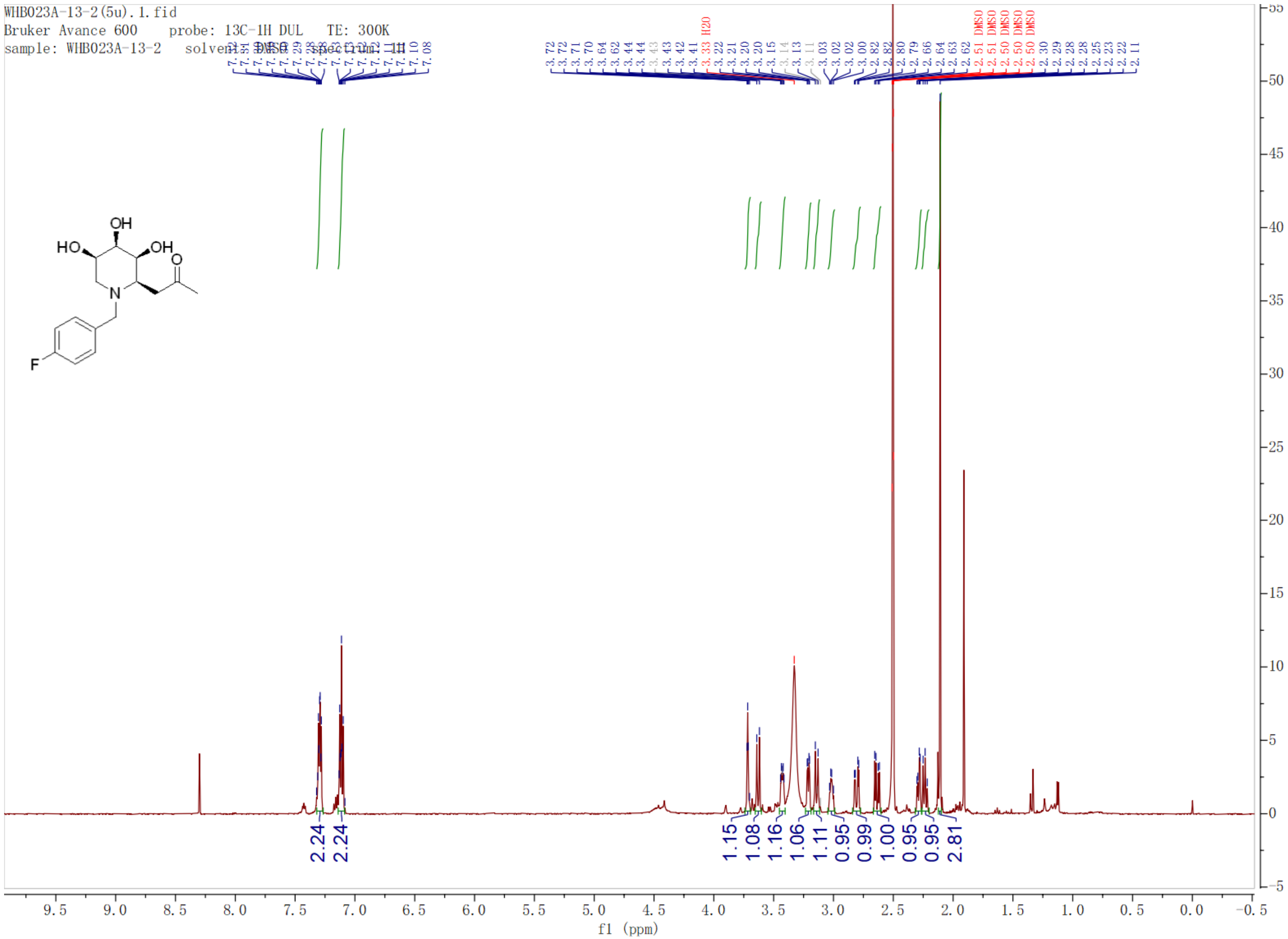

Figure S94. The ^{1}H NMR of compound **5x**.

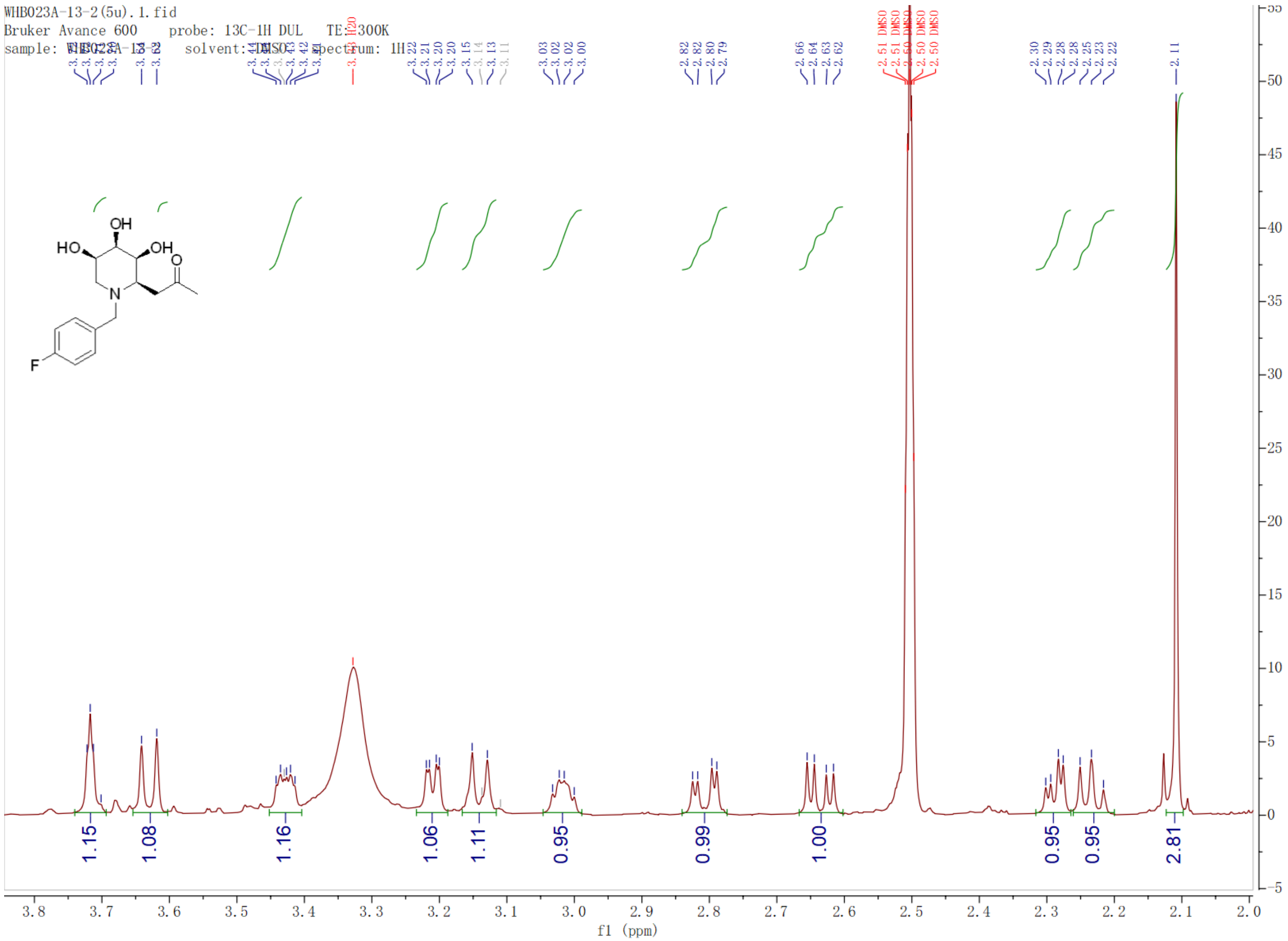

Figure S95. Partial region amplification of the ^{1}H NMR of compound **5x**.

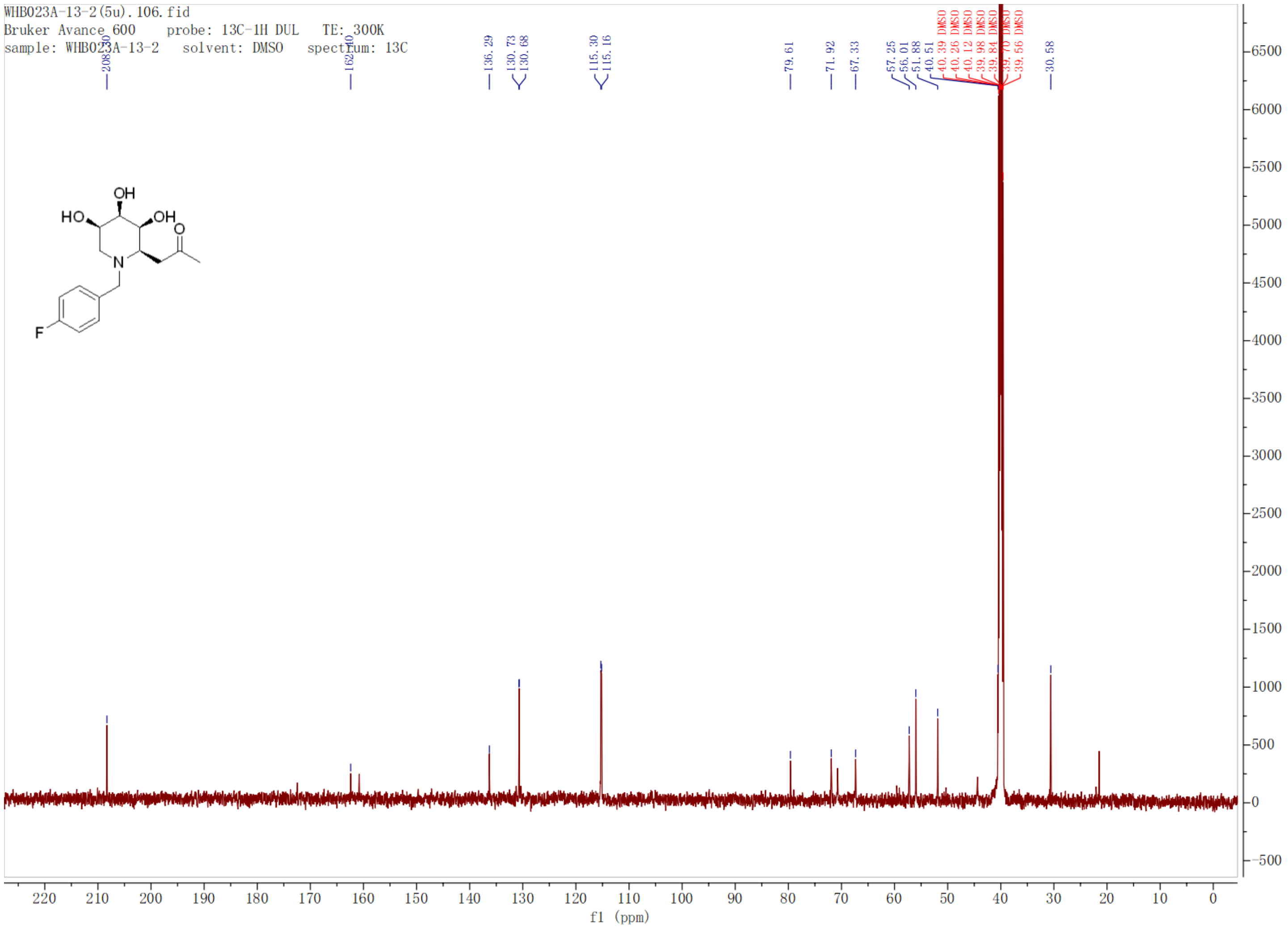

Figure S96. The ^{13}C NMR of compound **5x**.

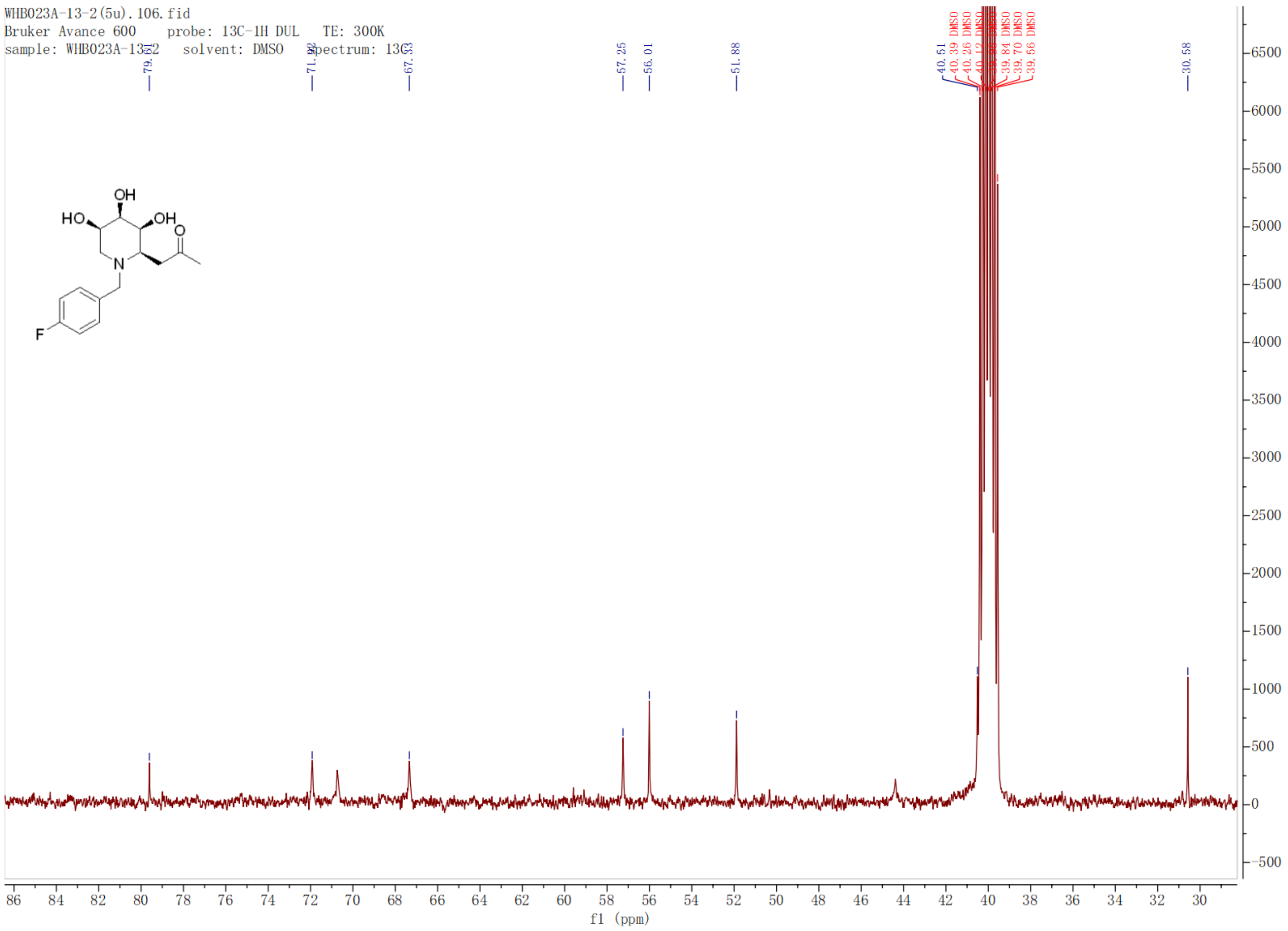

Figure S97. Partial region amplification of the ^{13}C NMR of compound **5x**.

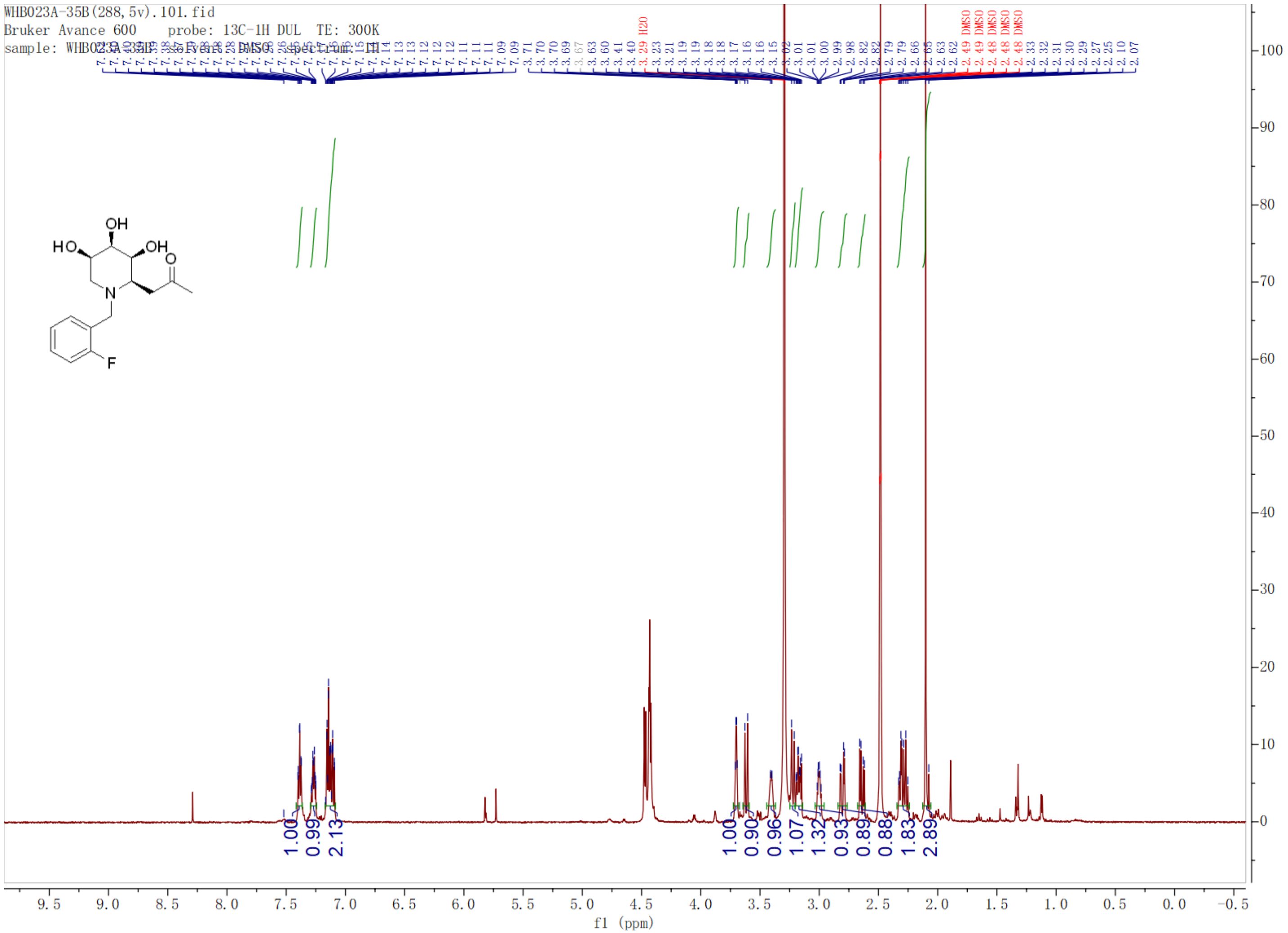

Figure S98. The ^{1}H NMR of compound **5y**.

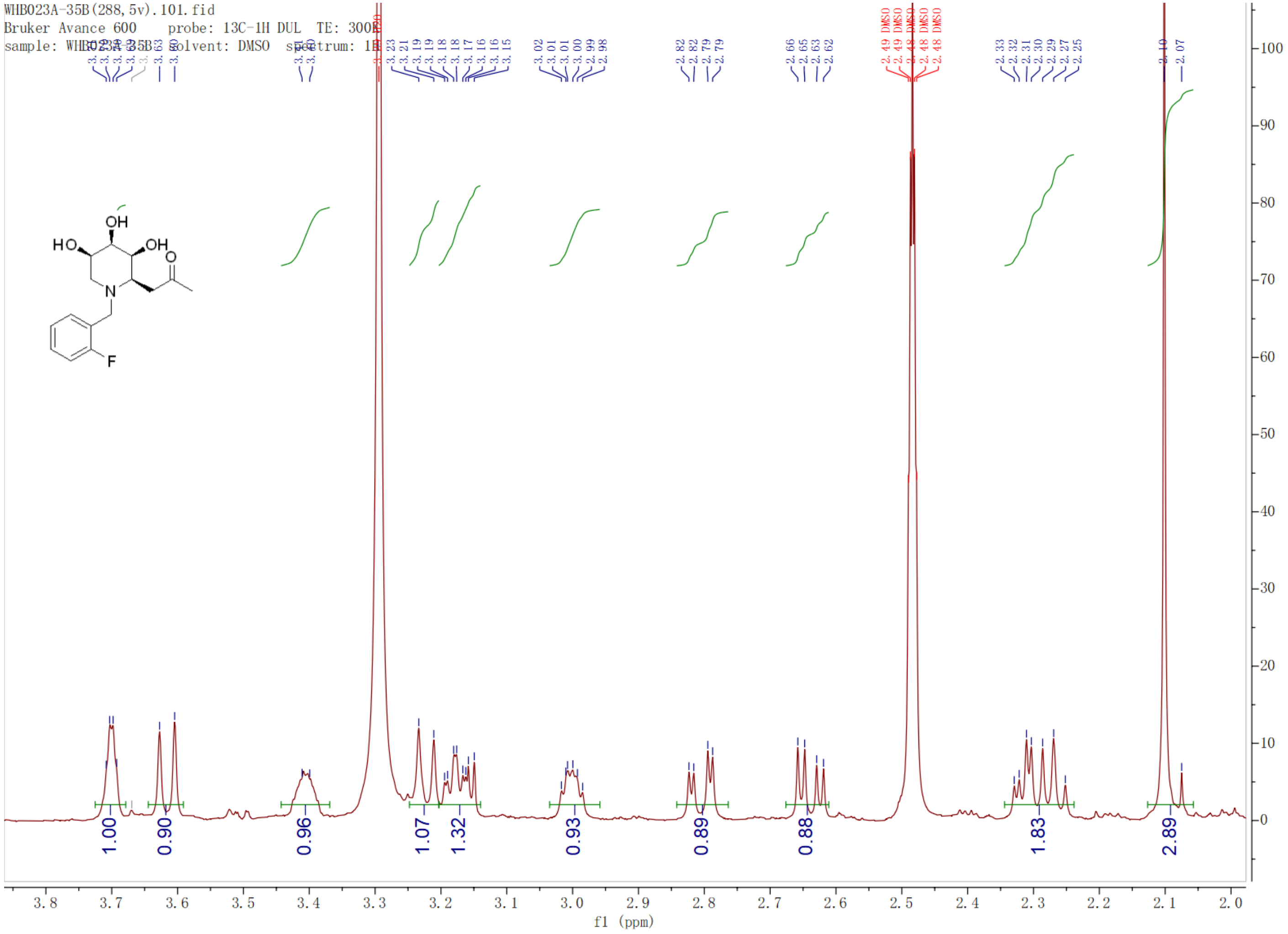

Figure S99. Partial region amplification of the ^{1}H NMR of compound **5y**.

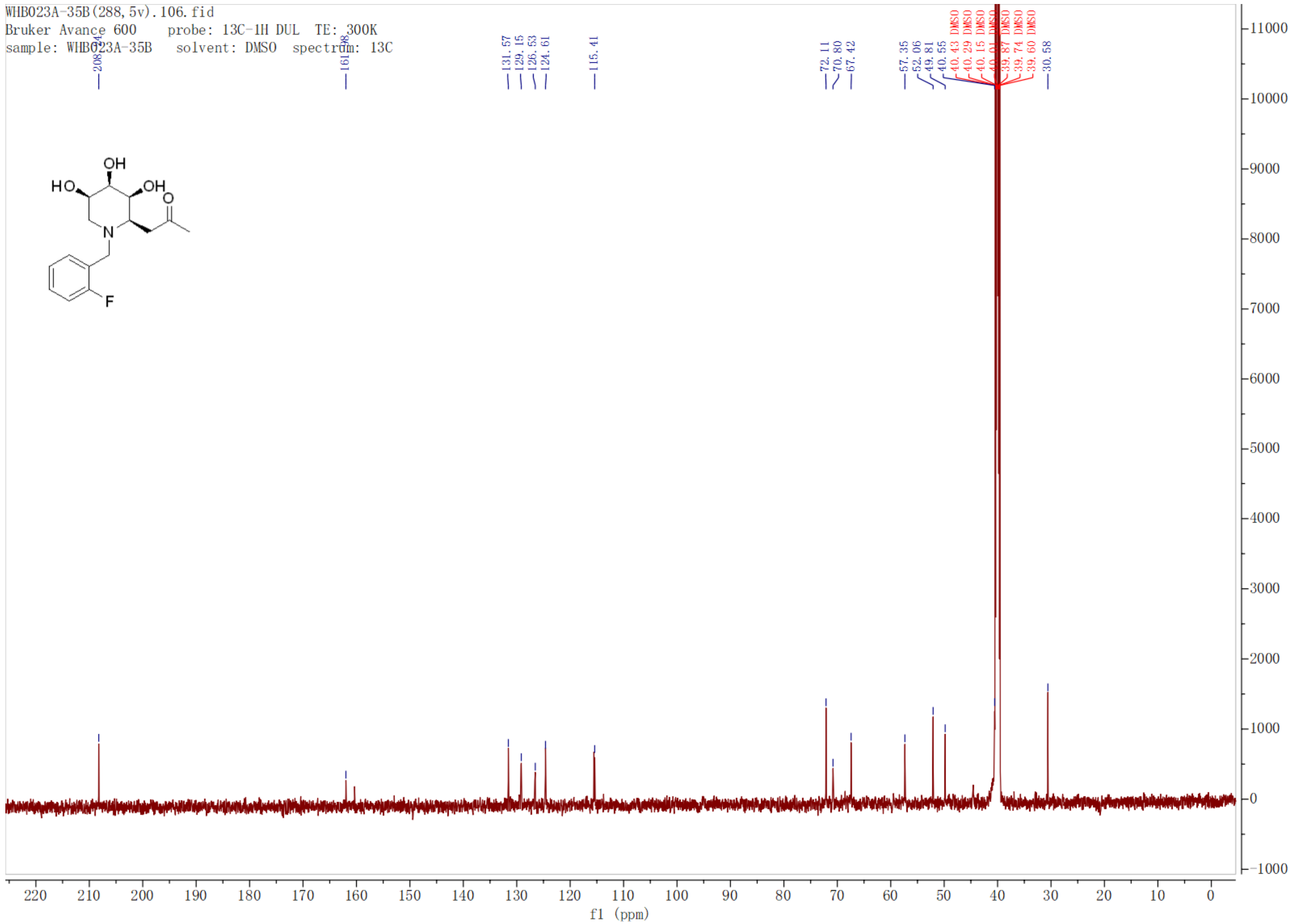

Figure S100. The ^{13}C NMR of compound **5y**.

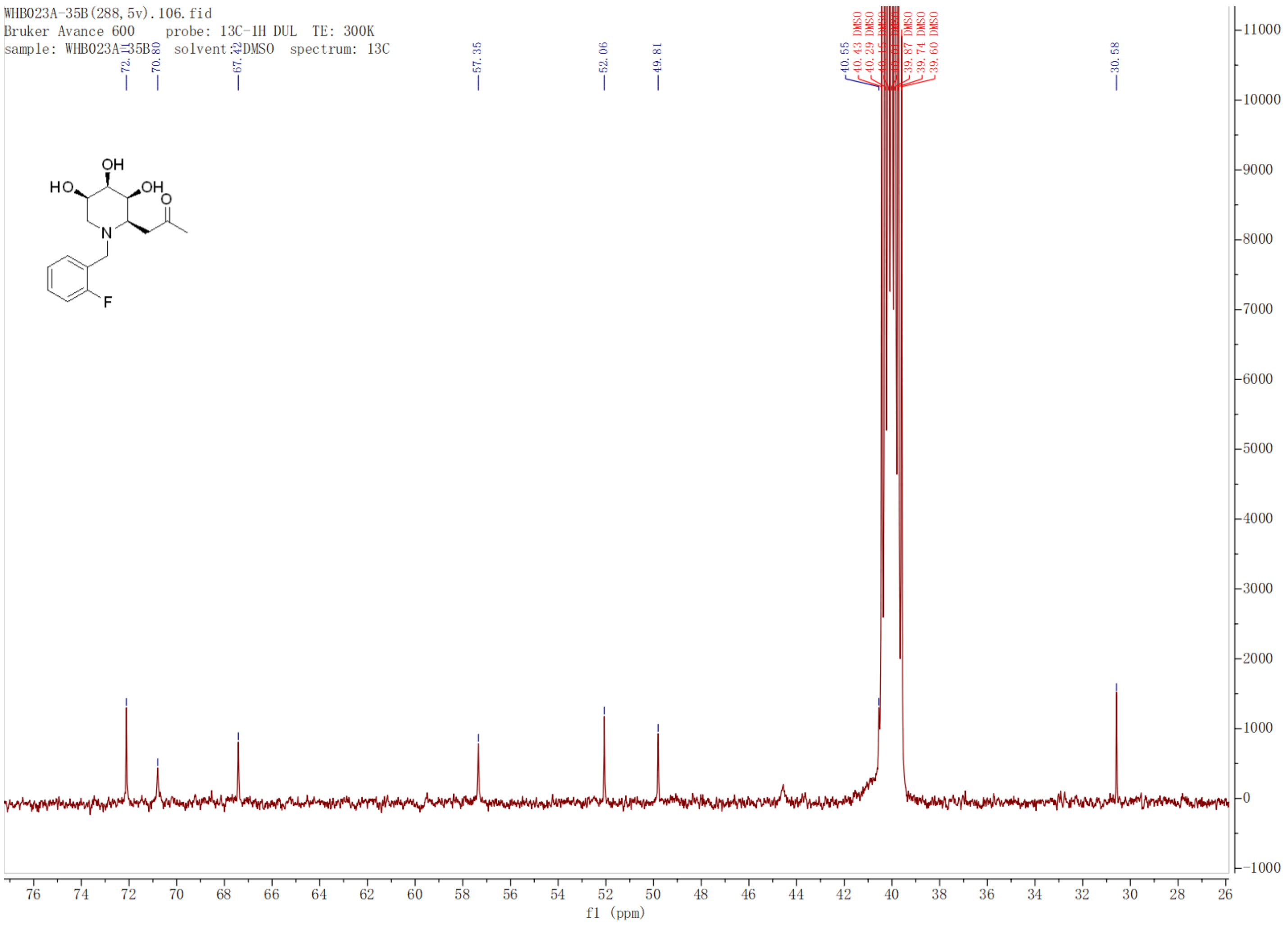

Figure S101. Partial region amplification of the ^{13}C NMR of compound **5y**.

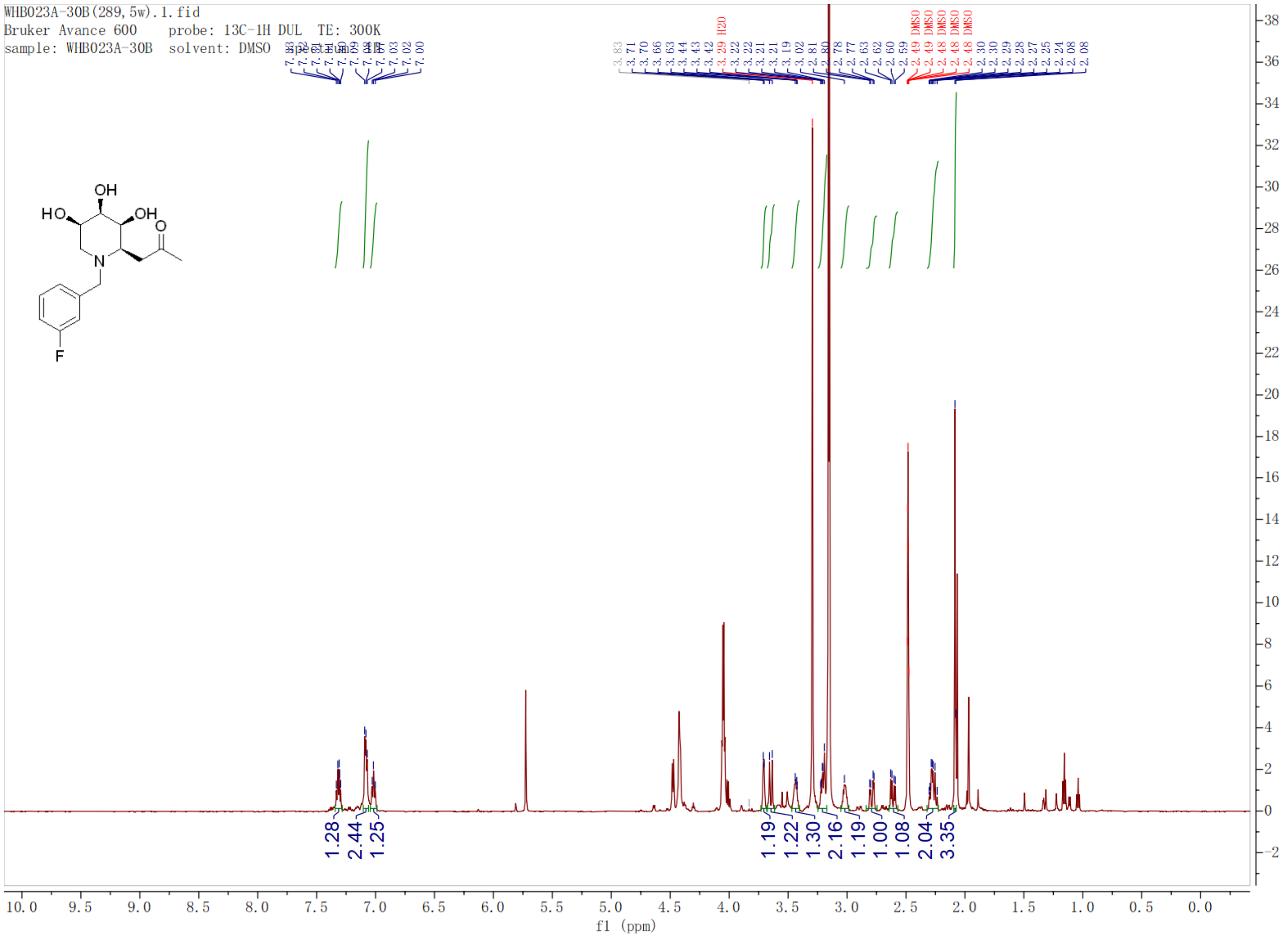

Figure S102. The ^{1}H NMR of compound **5z**.

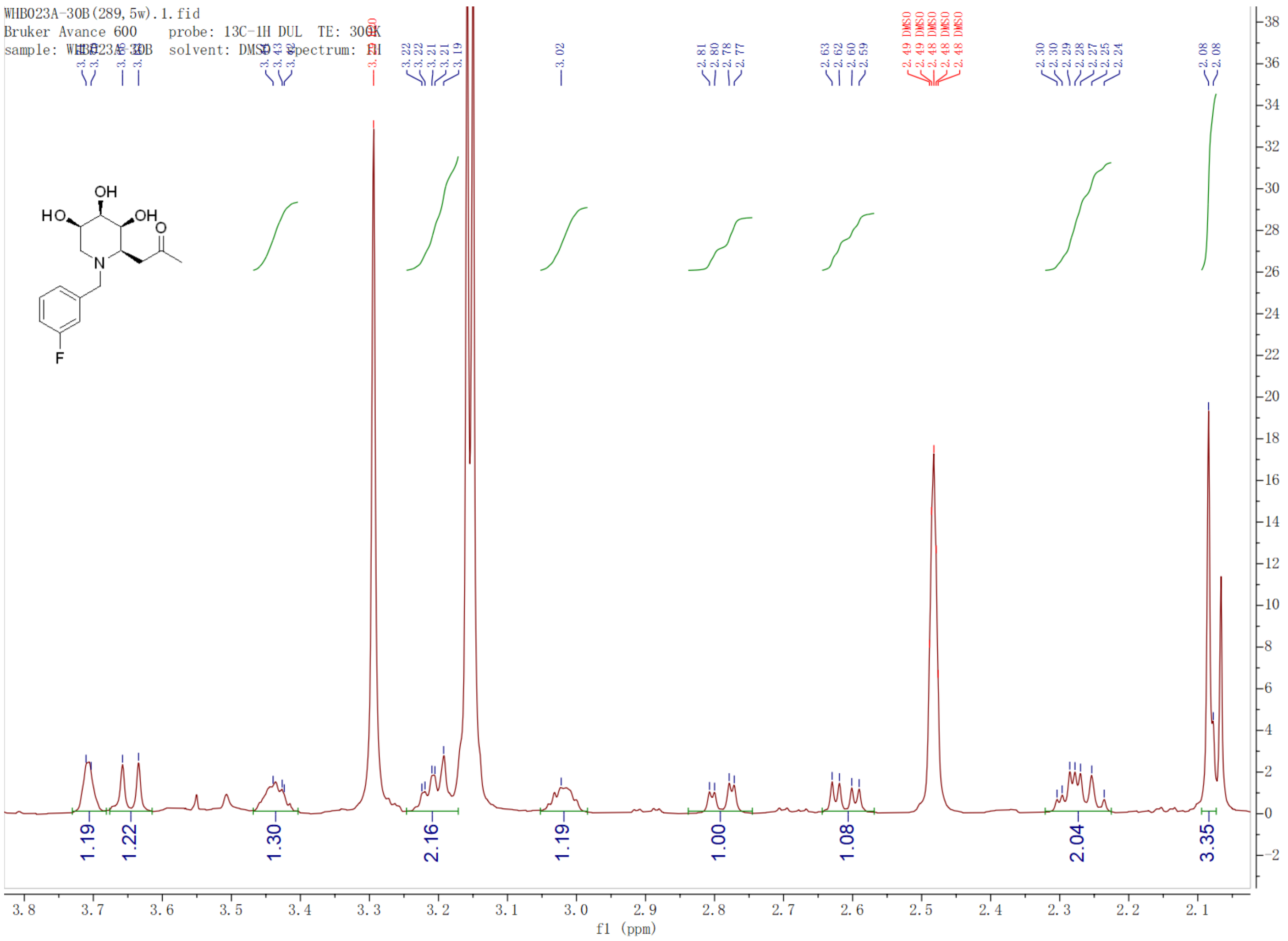

Figure S103. Partial region amplification of the ^1H NMR of compound **5z**.

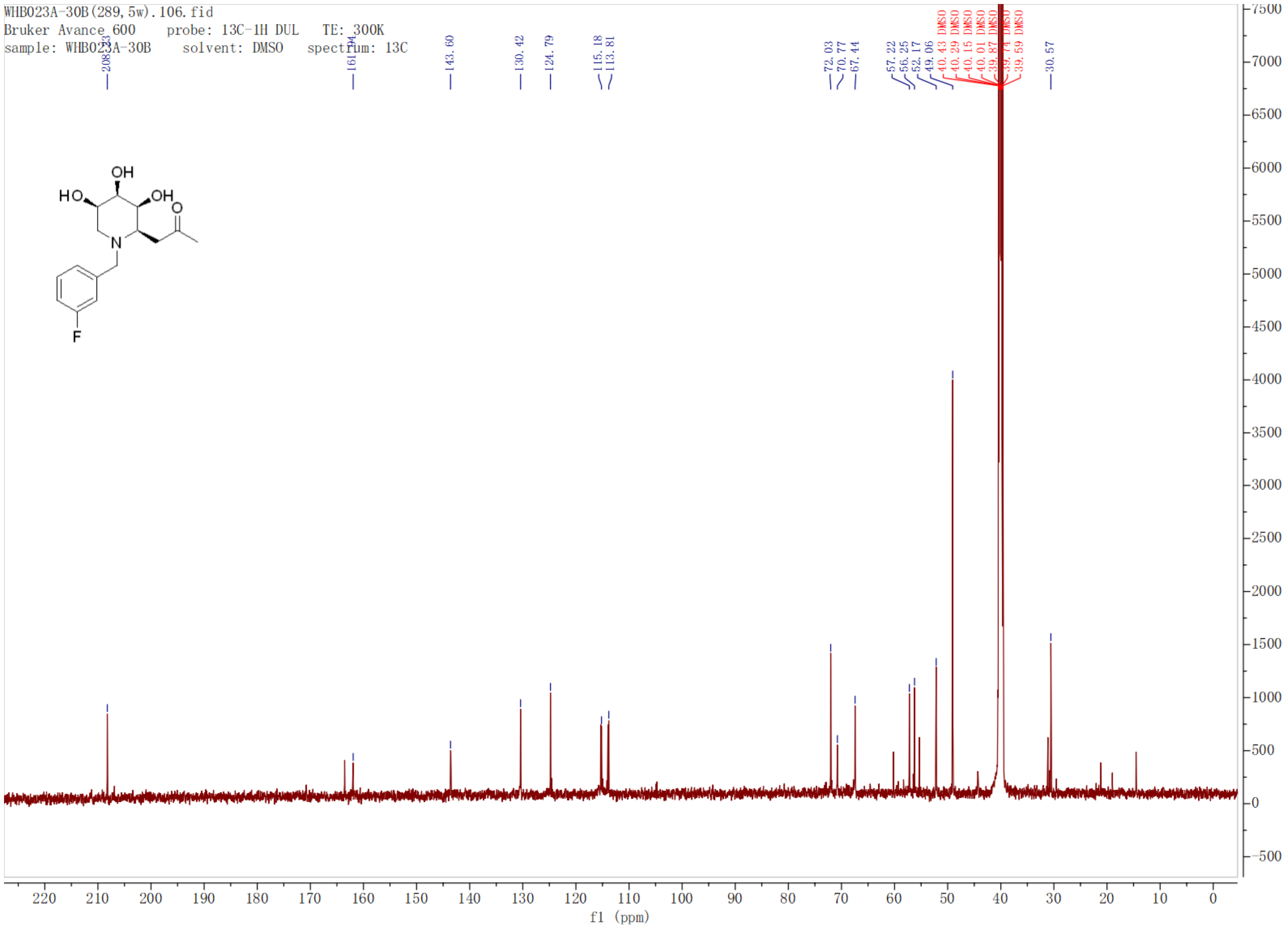

Figure S104. The ^{13}C NMR of compound **5z**.

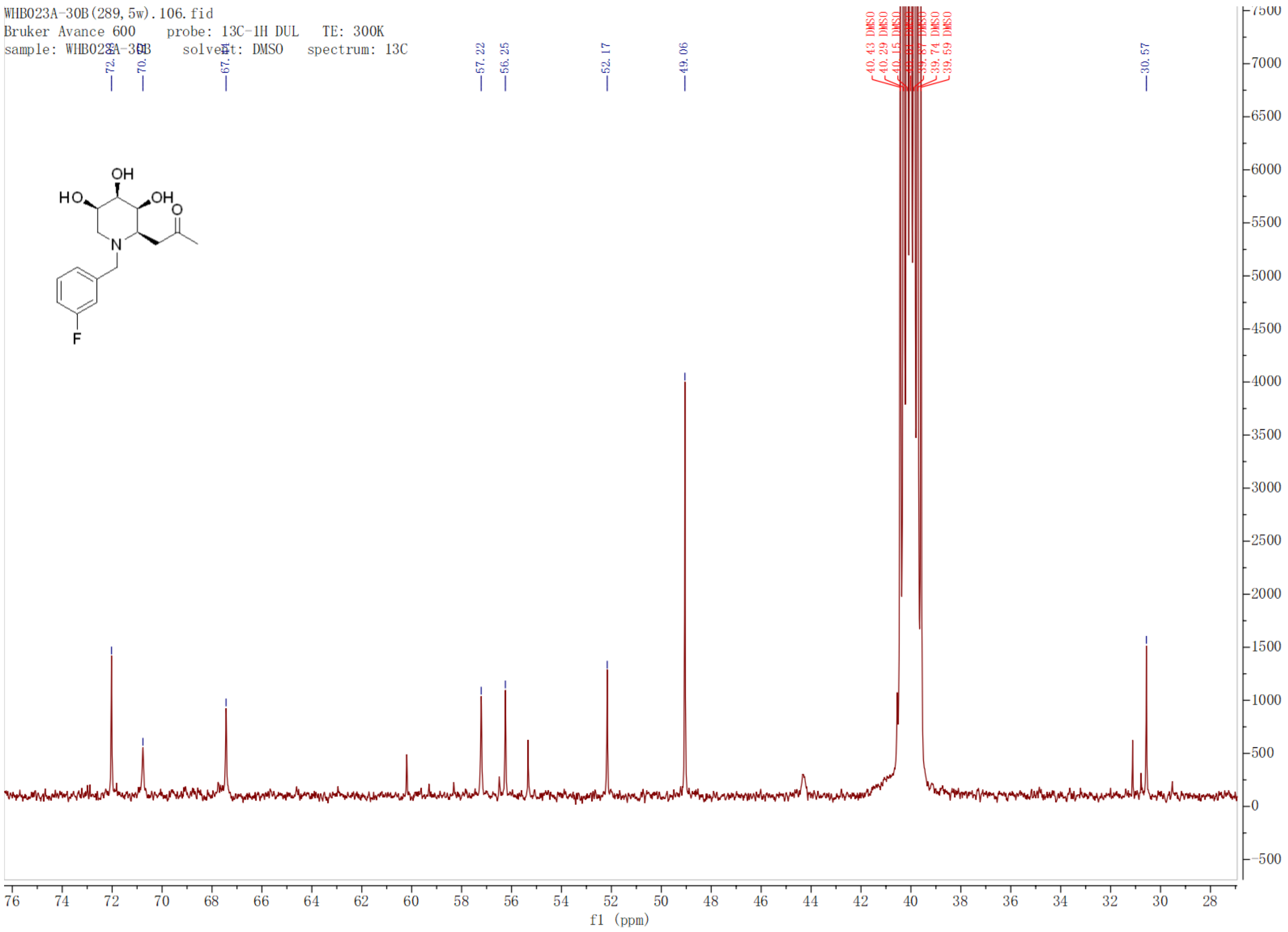

Figure S105. Partial region amplification of the ^{13}C NMR of compound **5z**.

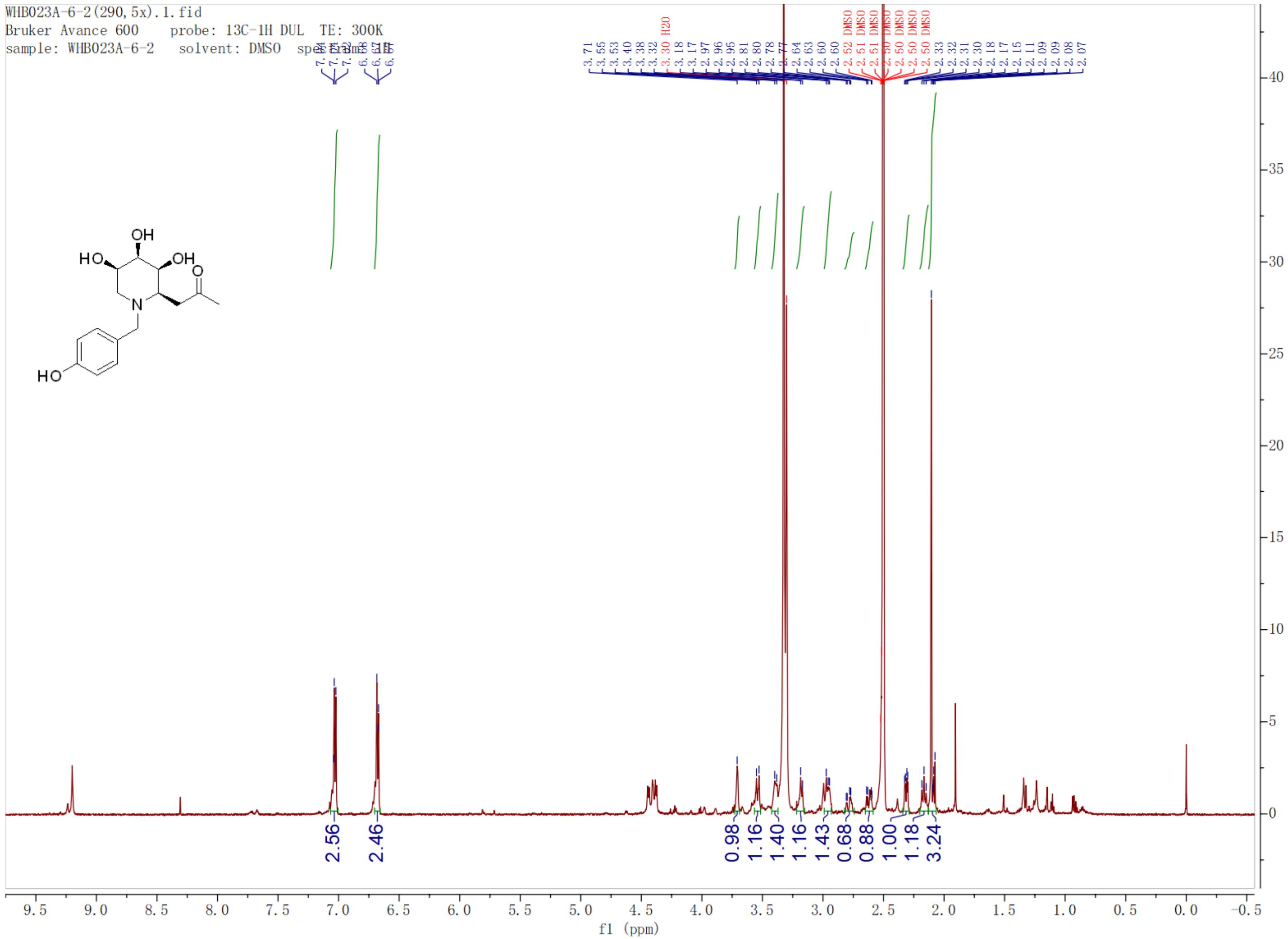

Figure S106. The ^{1}H NMR of compound **5λ**.

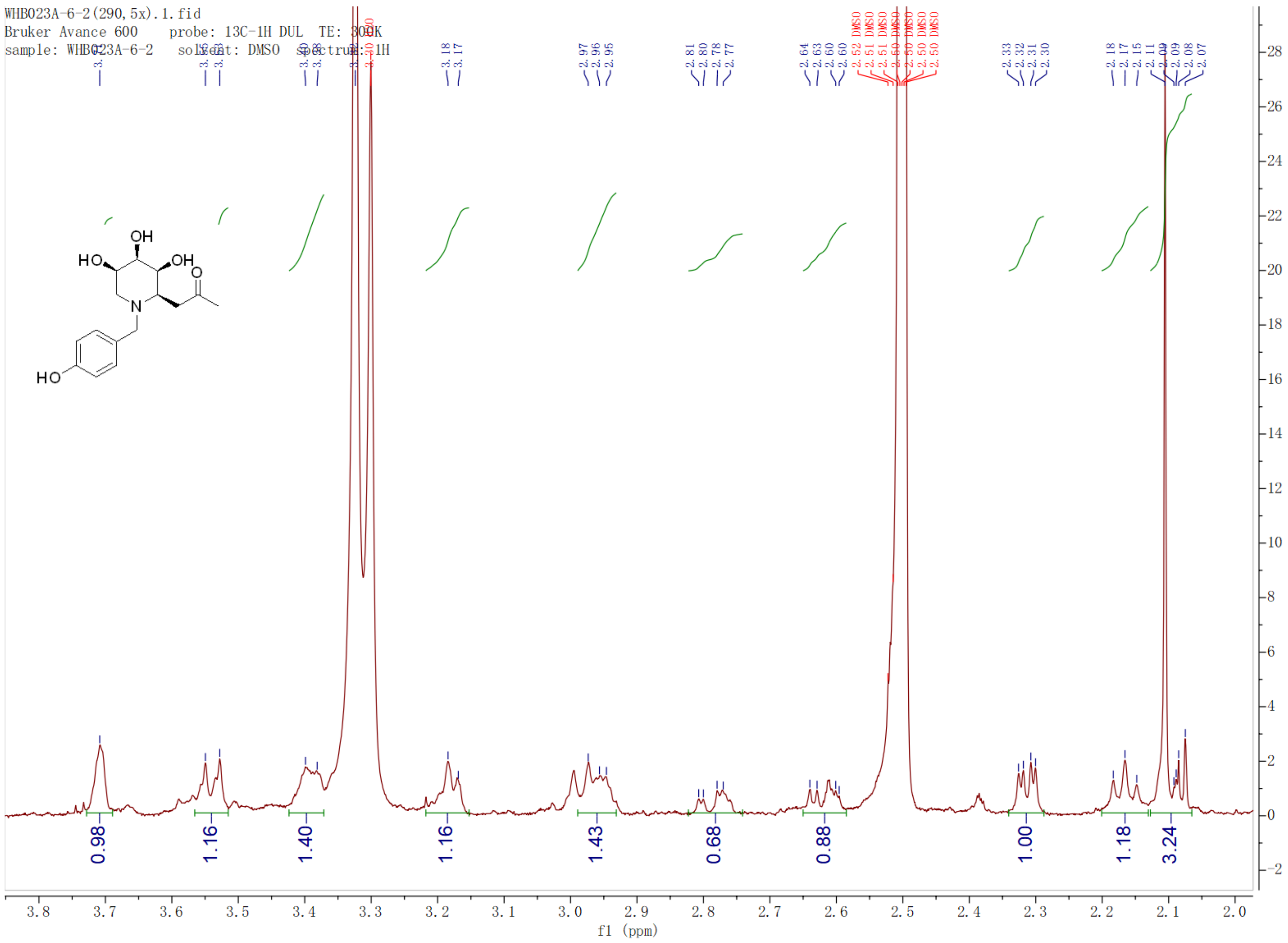

Figure S107. Partial region amplification of the ^{1}H NMR of compound **5λ**.

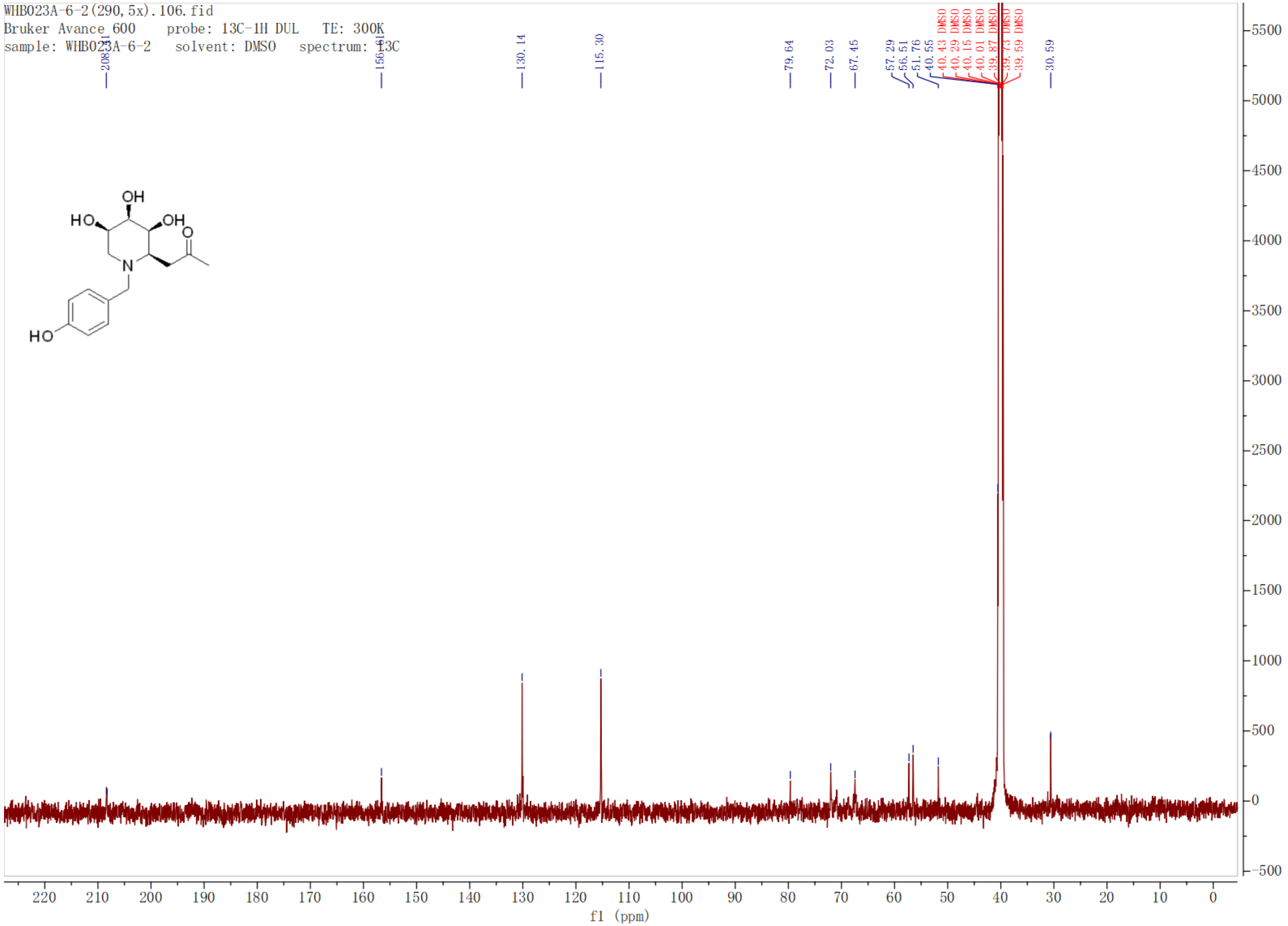

Figure S108. The ^{13}C NMR of compound **5λ**.

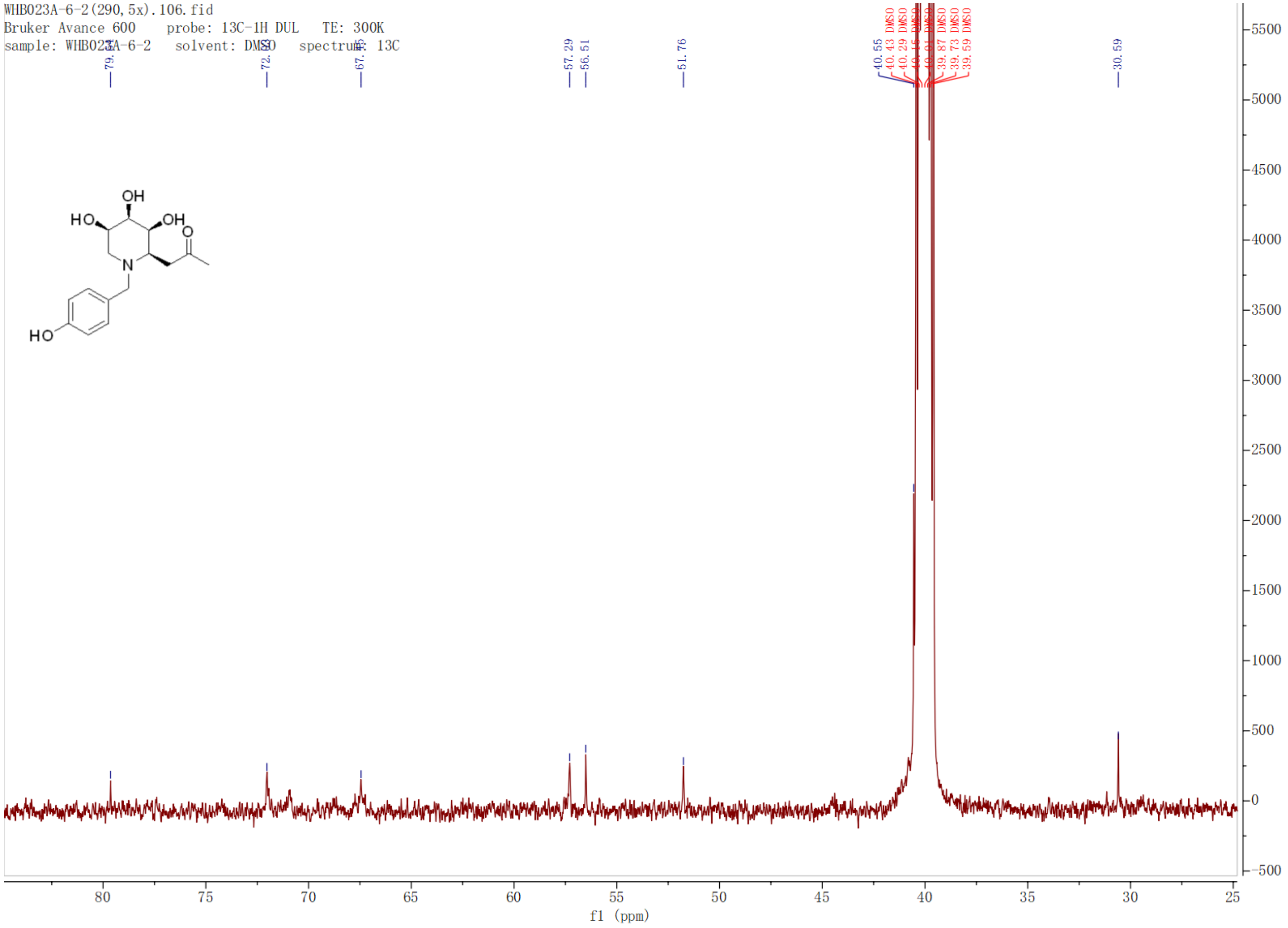

Figure S109. Partial region amplification of the ^{13}C NMR of compound **5λ**.

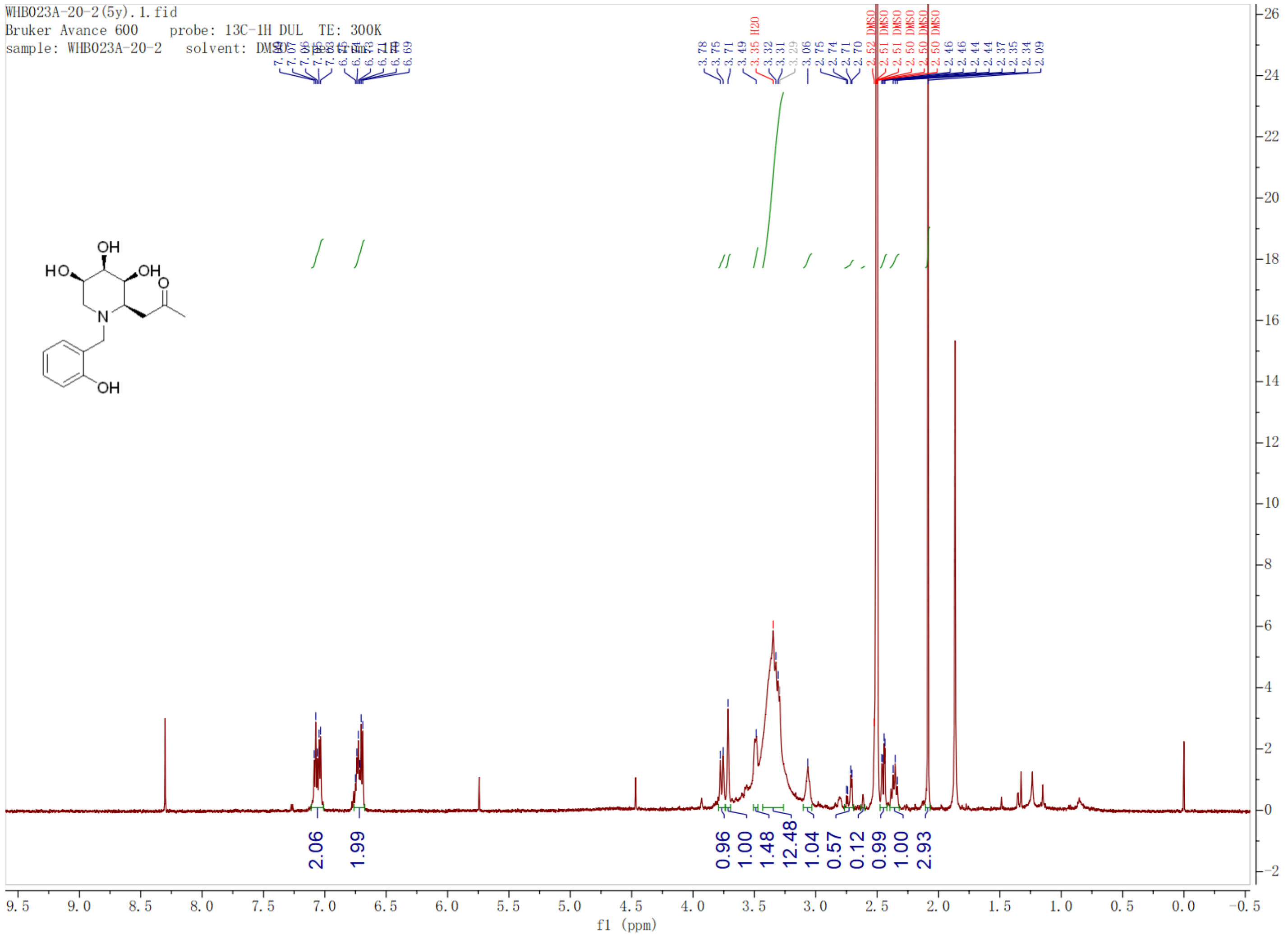

Figure S110. The ¹H NMR of compound **5θ**.

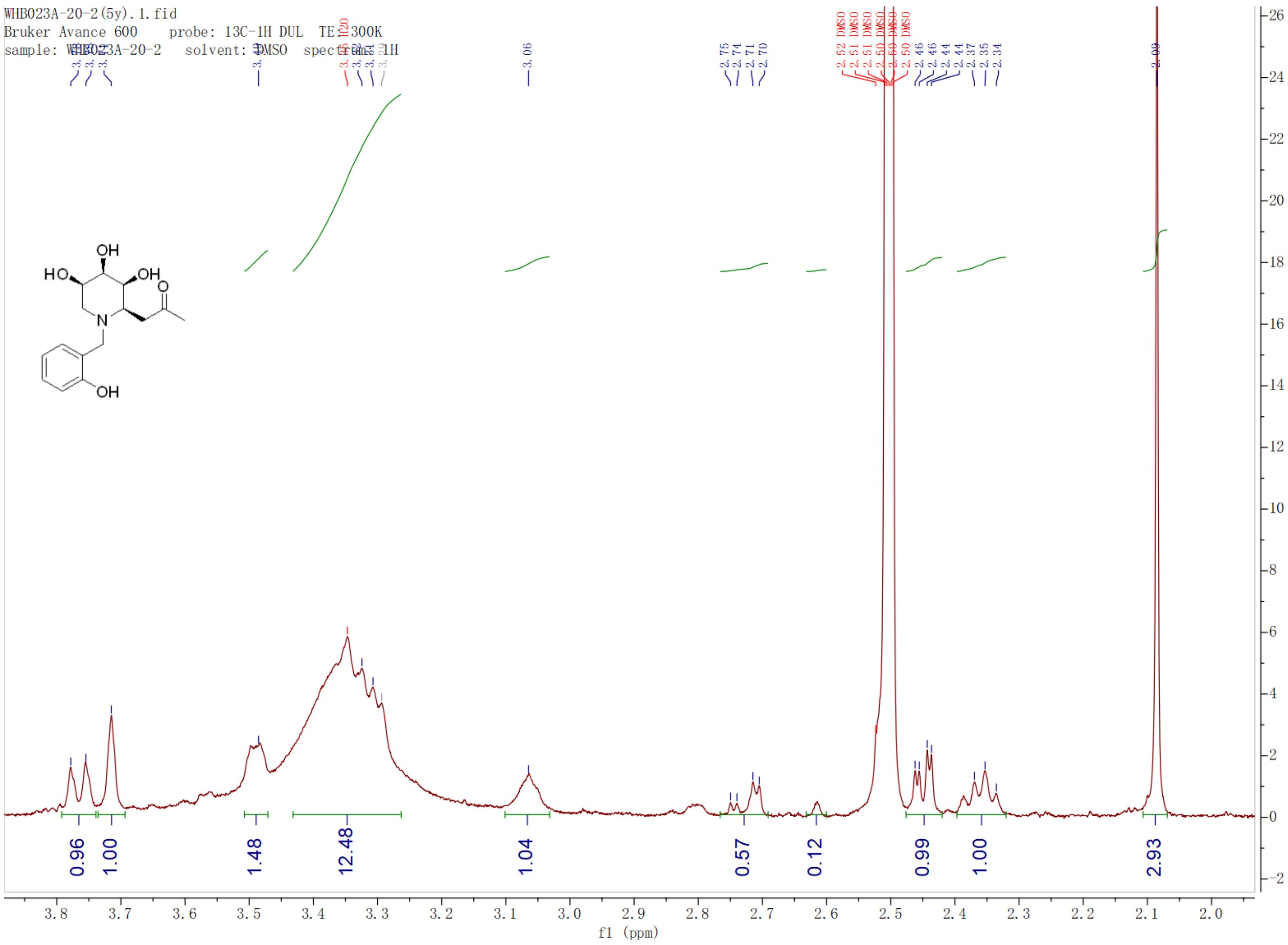

Figure S111. Partial region amplification of the ^{1}H NMR of compound **5θ**.

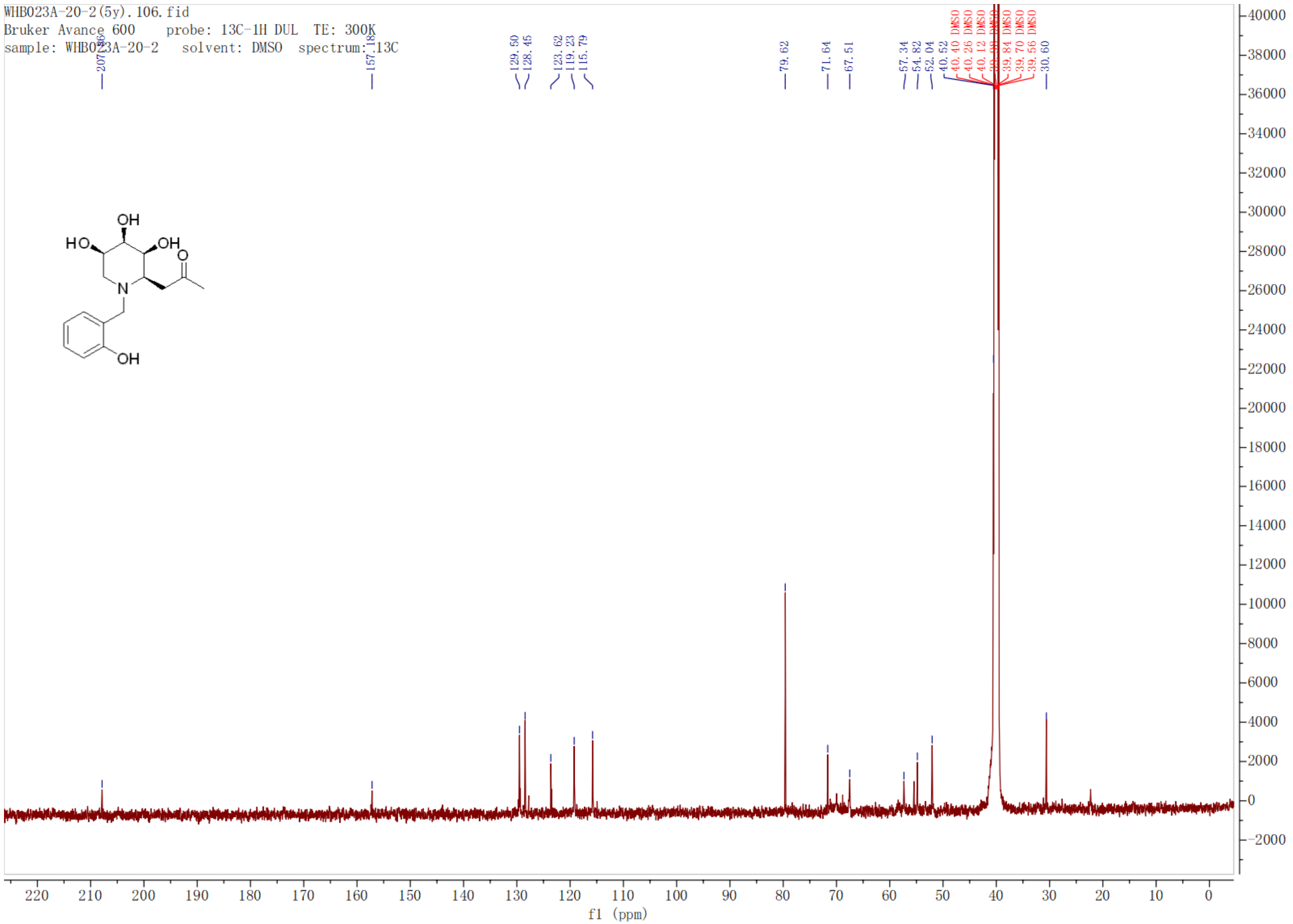

Figure S112. The ^{13}C NMR of compound **50**.

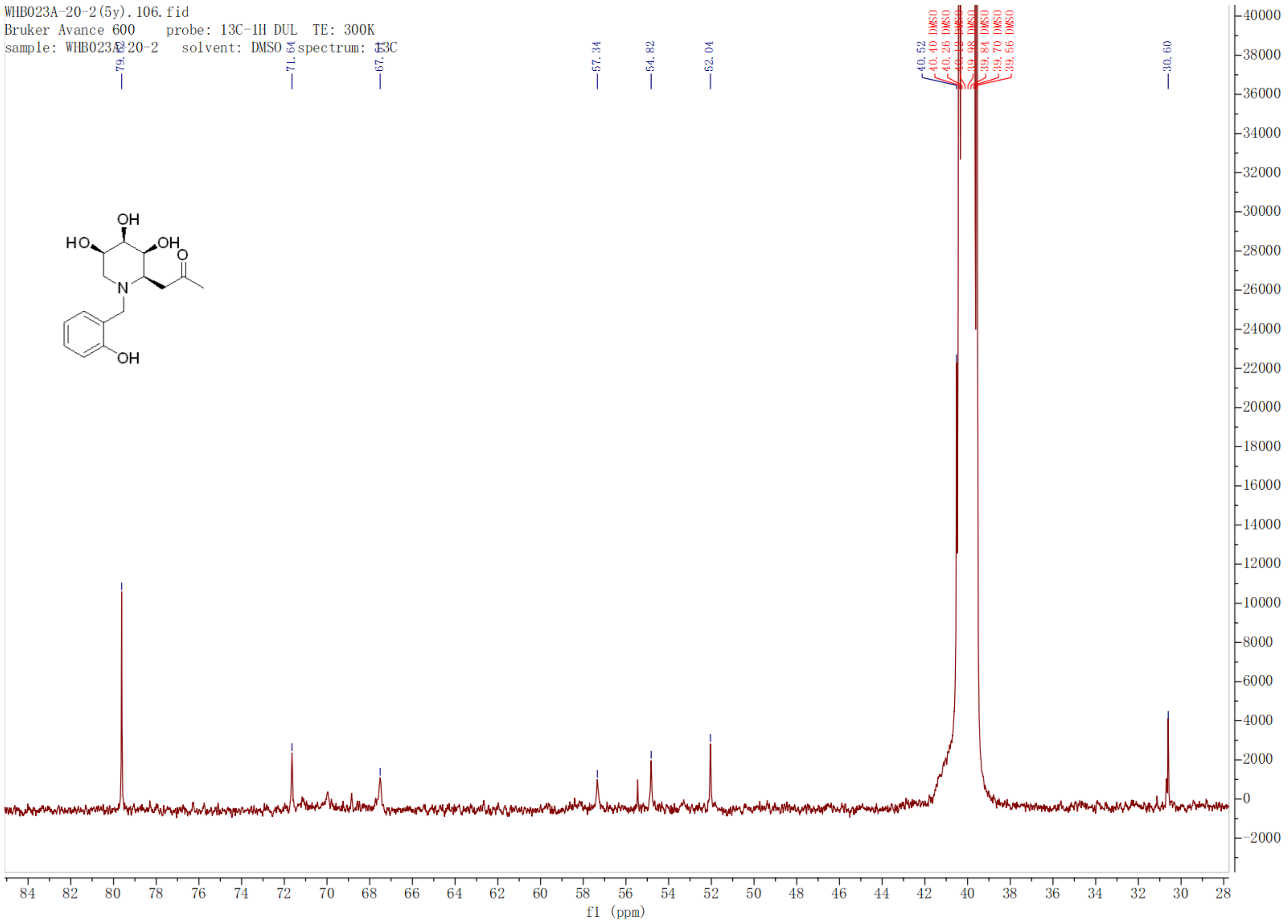

Figure S113. Partial region amplification of the ^{13}C NMR of compound **5θ**.

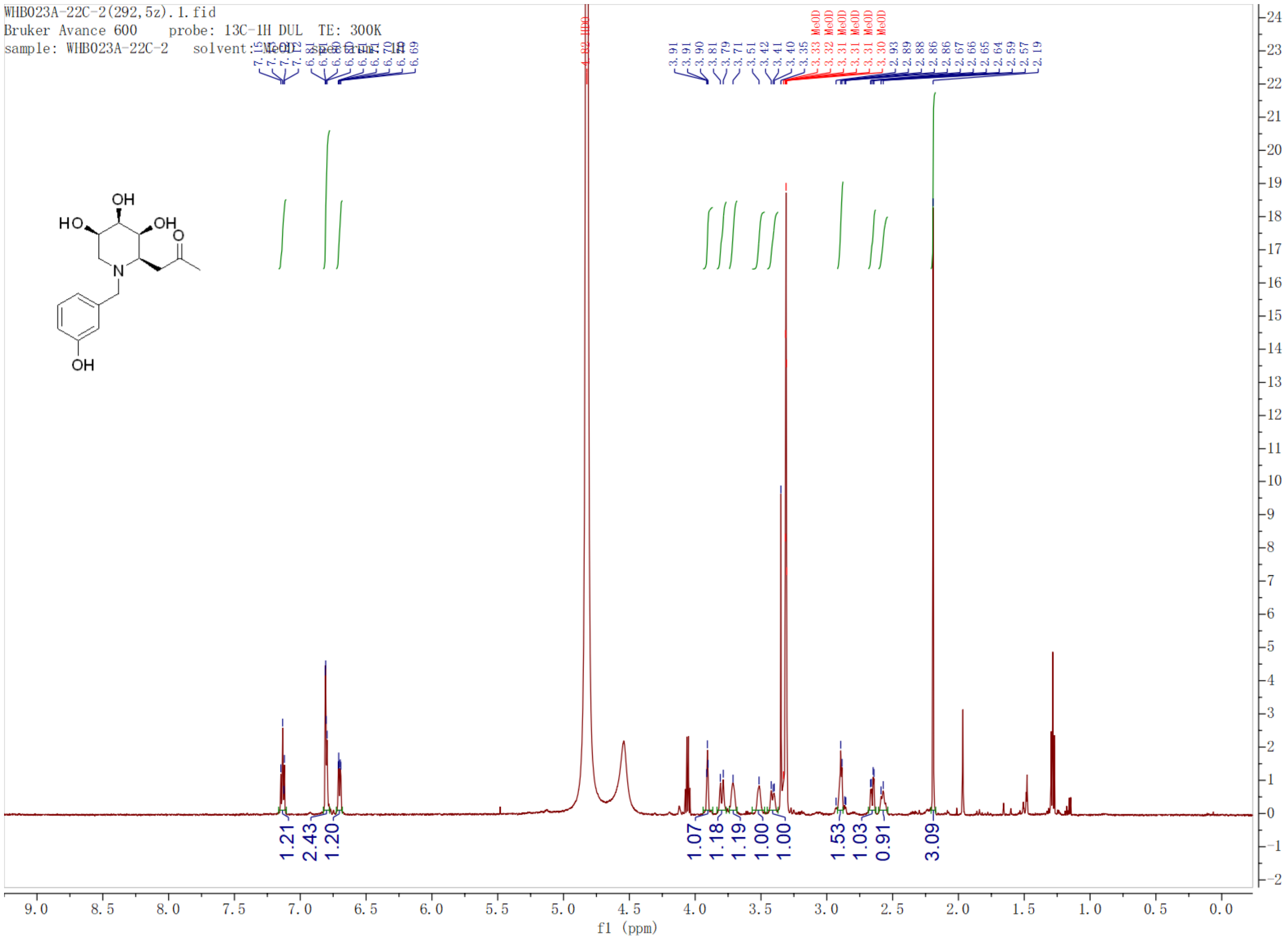

Figure S114. The ^{1}H NMR of compound **5π**.

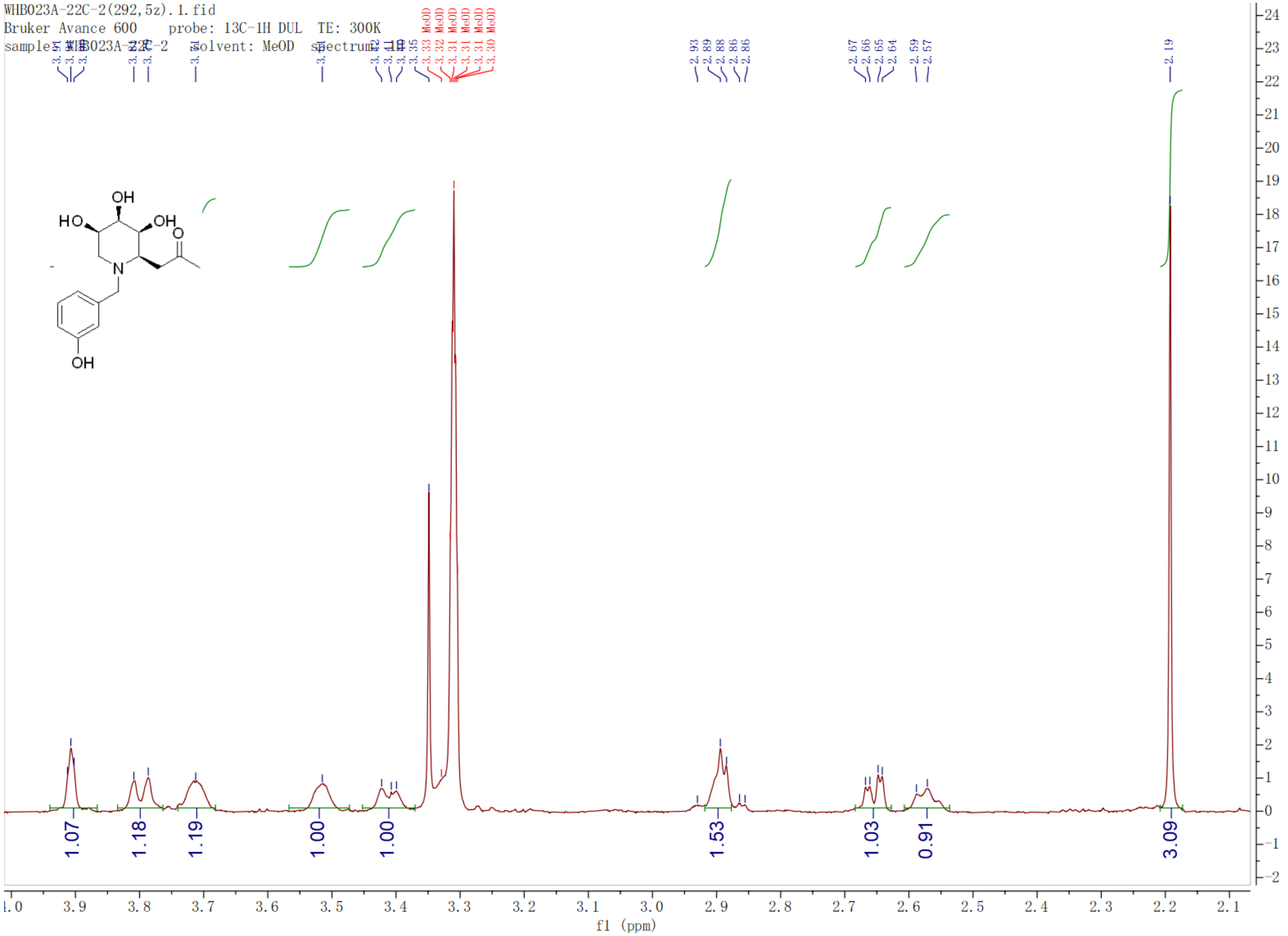

Figure S115. Partial region amplification of the ^{1}H NMR of compound **5π**.

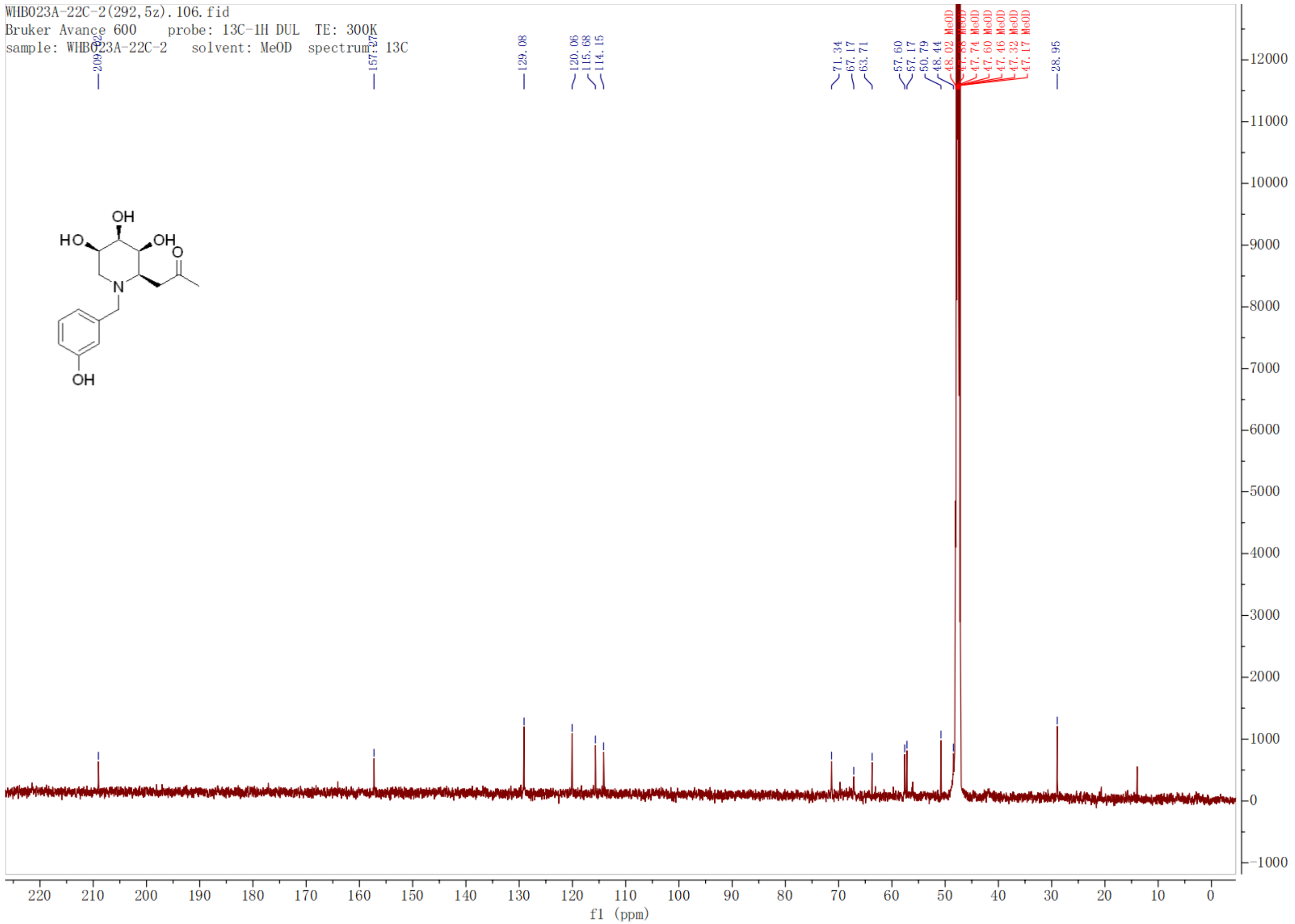

Figure S116. The ^{13}C NMR of compound **5π**.

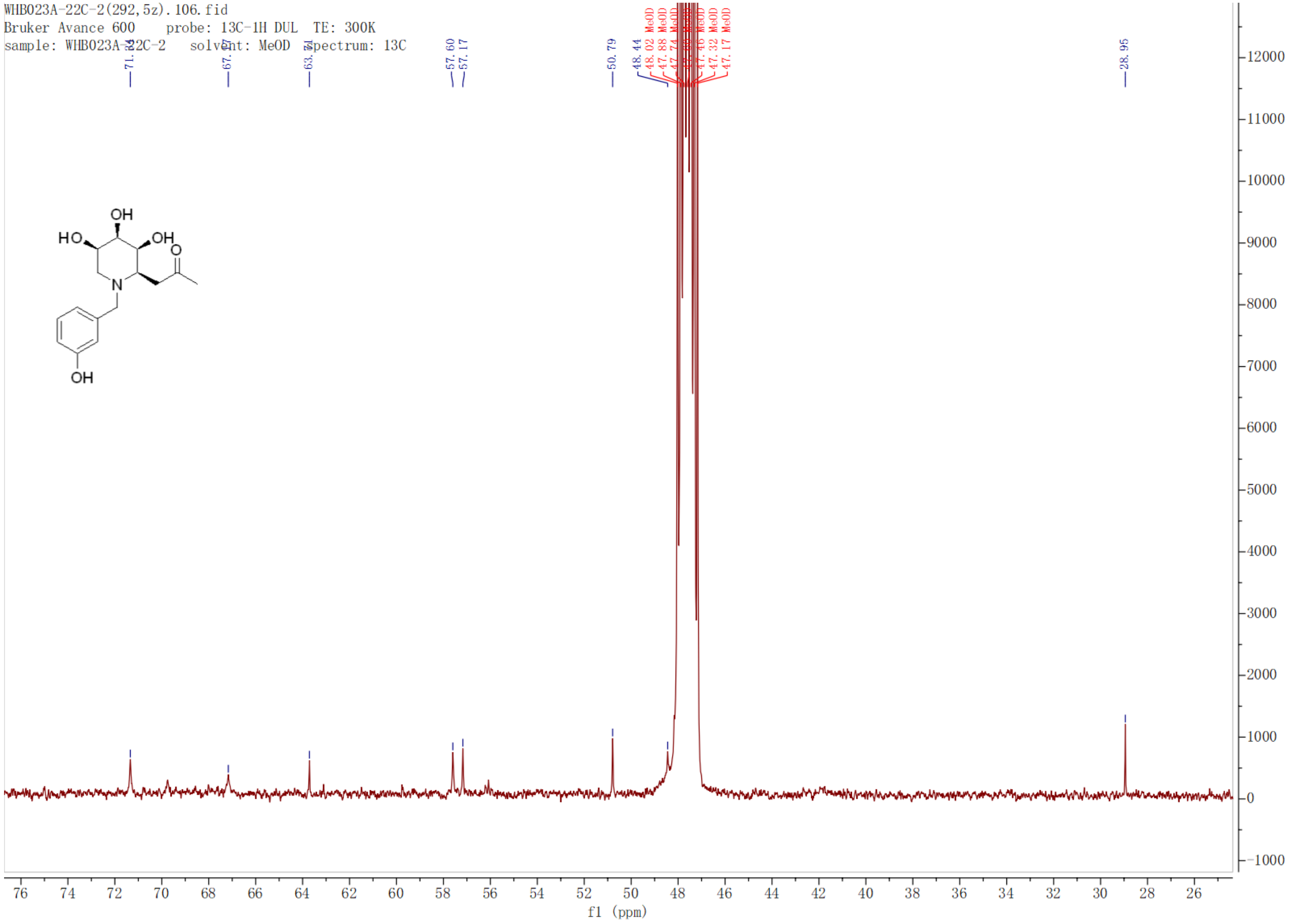

Figure S117. Partial region amplification of the ^{13}C NMR of compound **5π**.

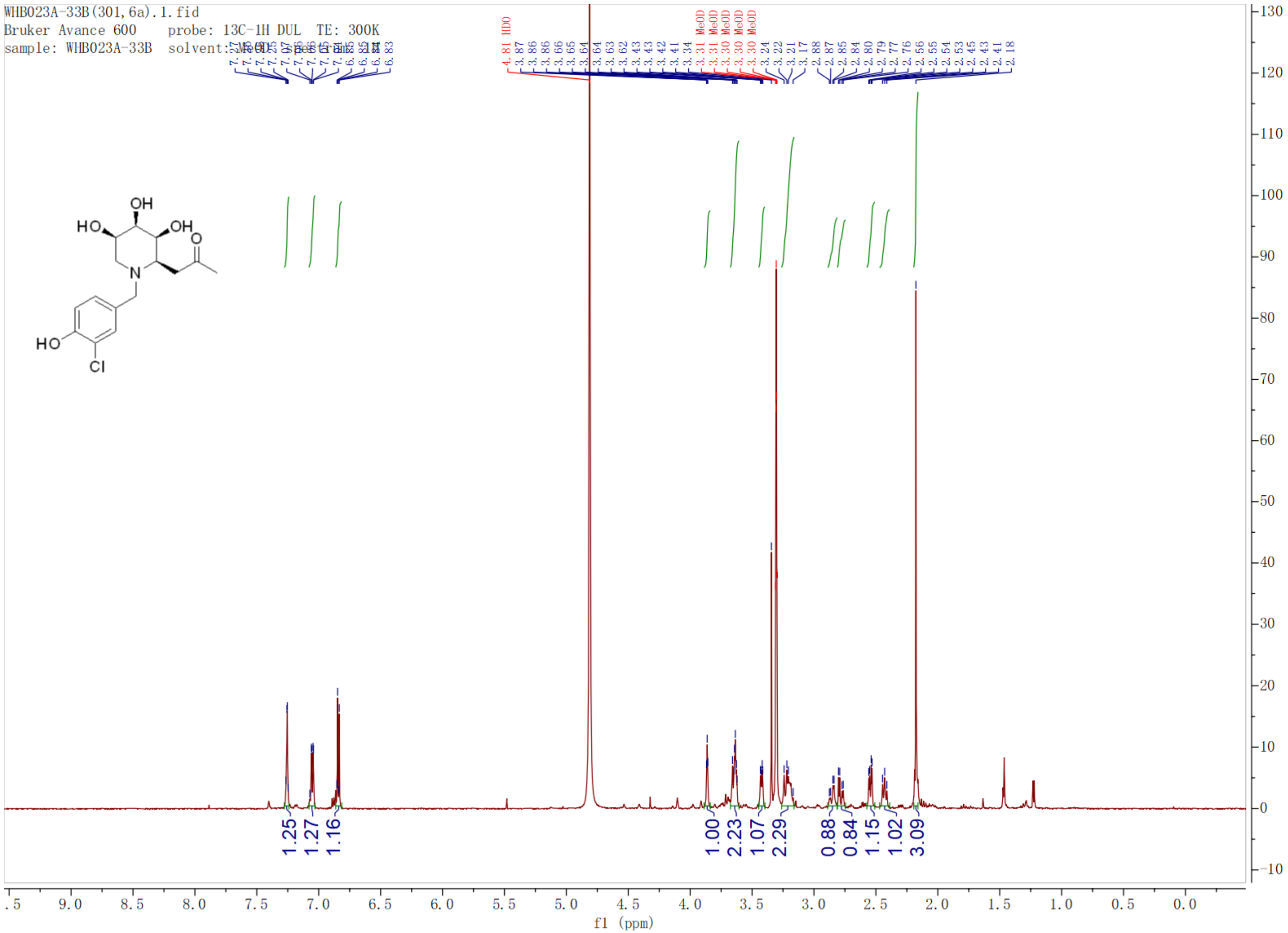

Figure S118. The ^{1}H NMR of compound **6a**.

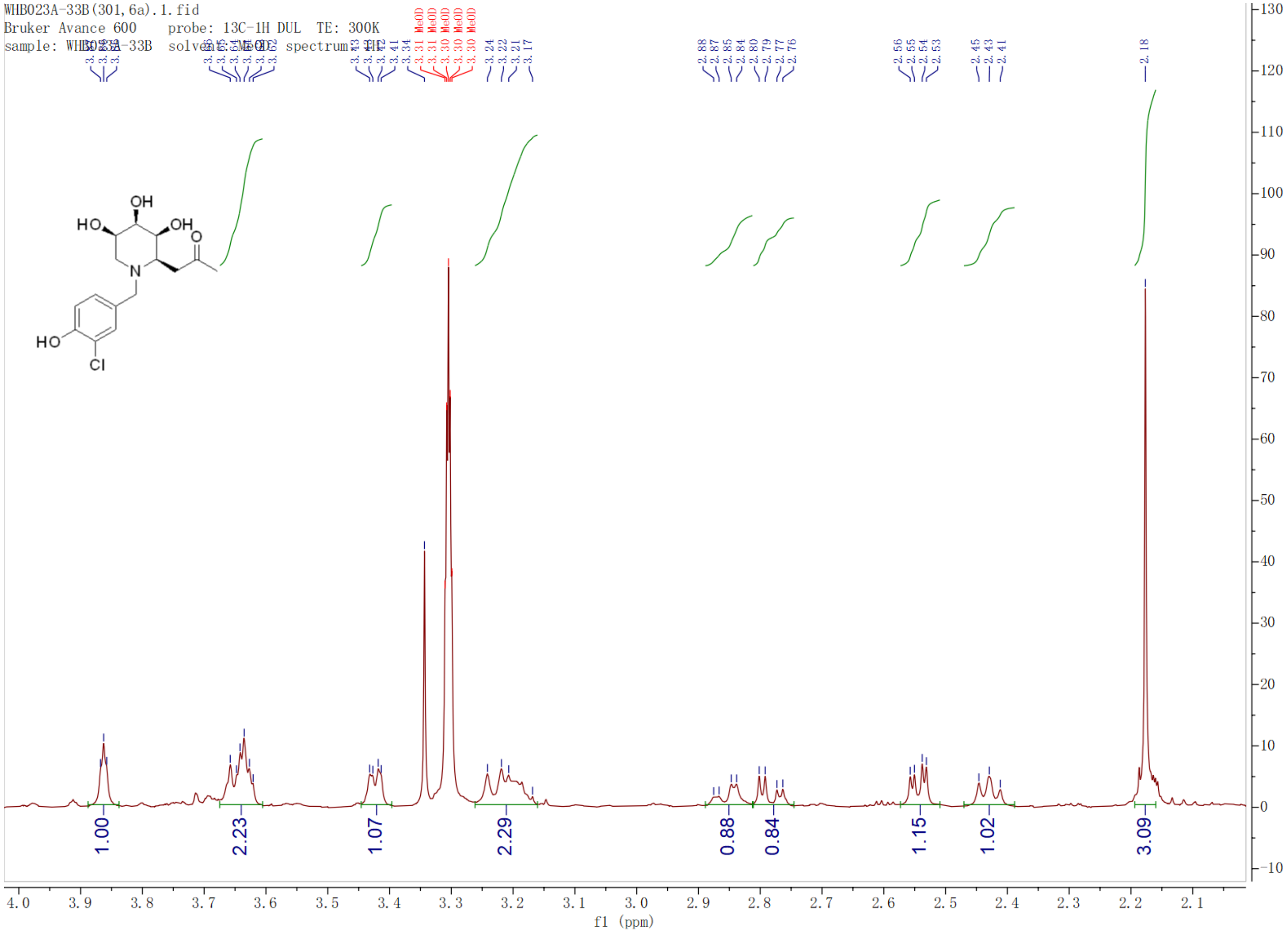

Figure S119. Partial region amplification of the ^{1}H NMR of compound **6a**.

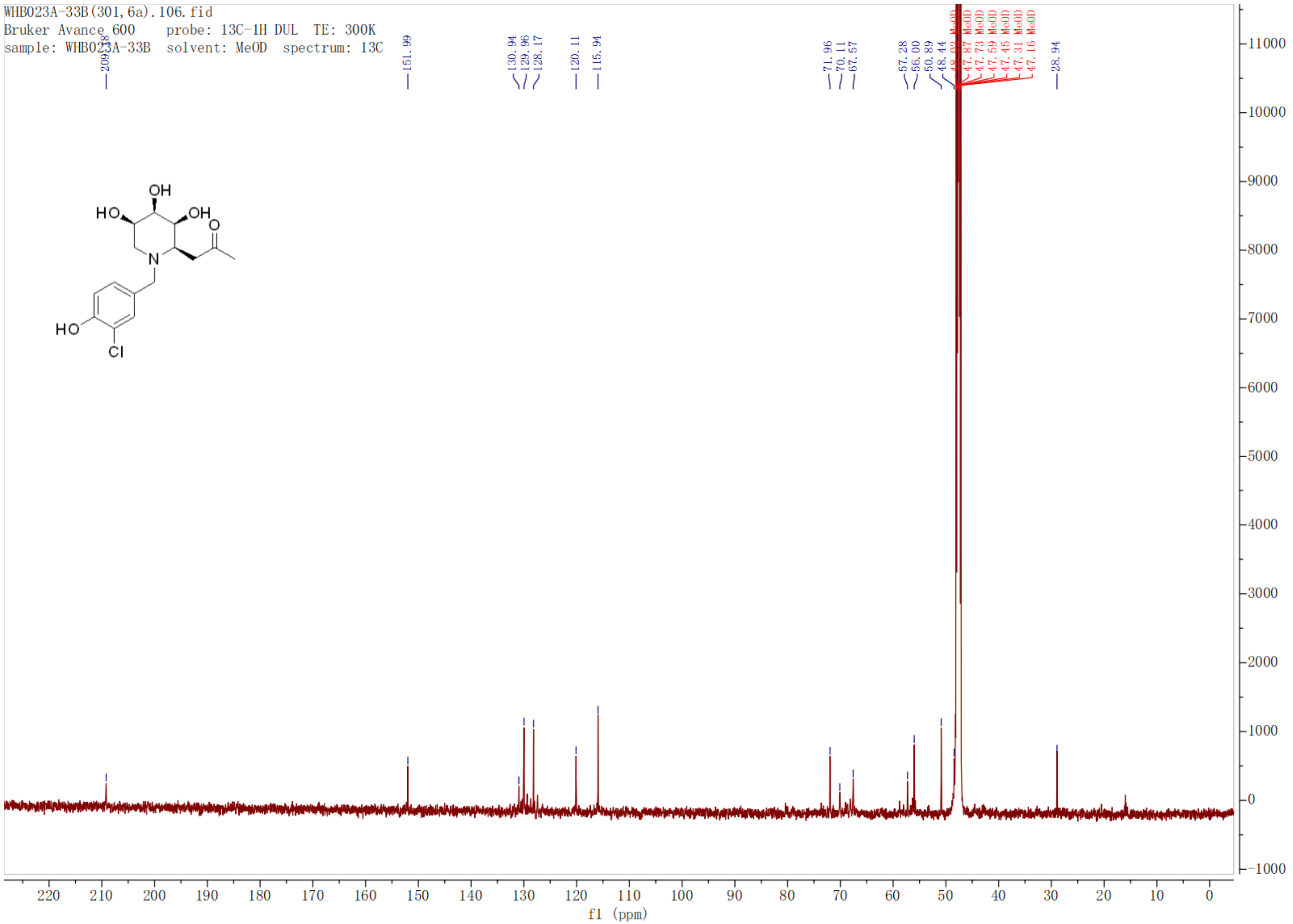

Figure S120. The ^{13}C NMR of compound **6a**.

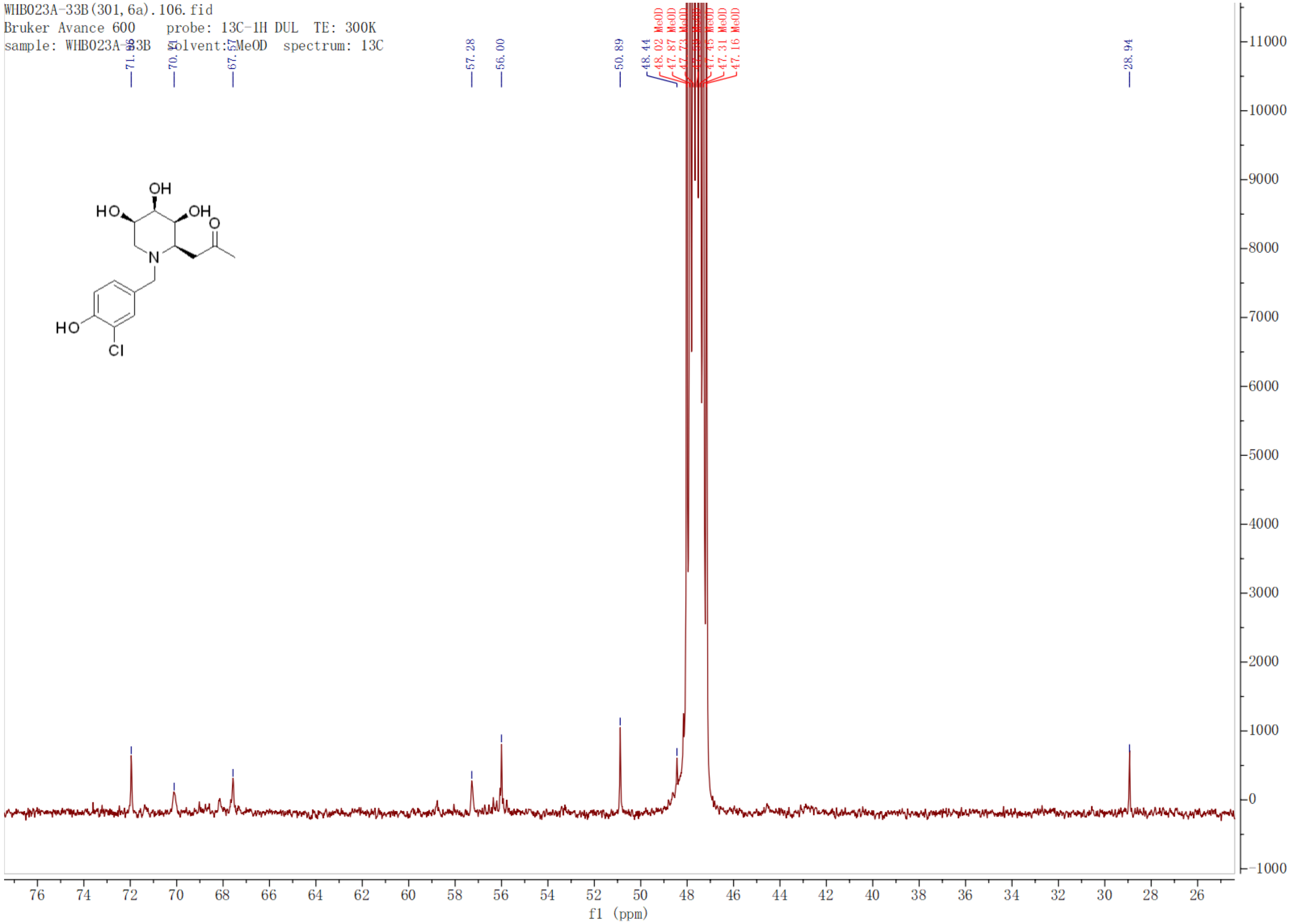

Figure S121. Partial region amplification of the ^{13}C NMR of compound **6a**.

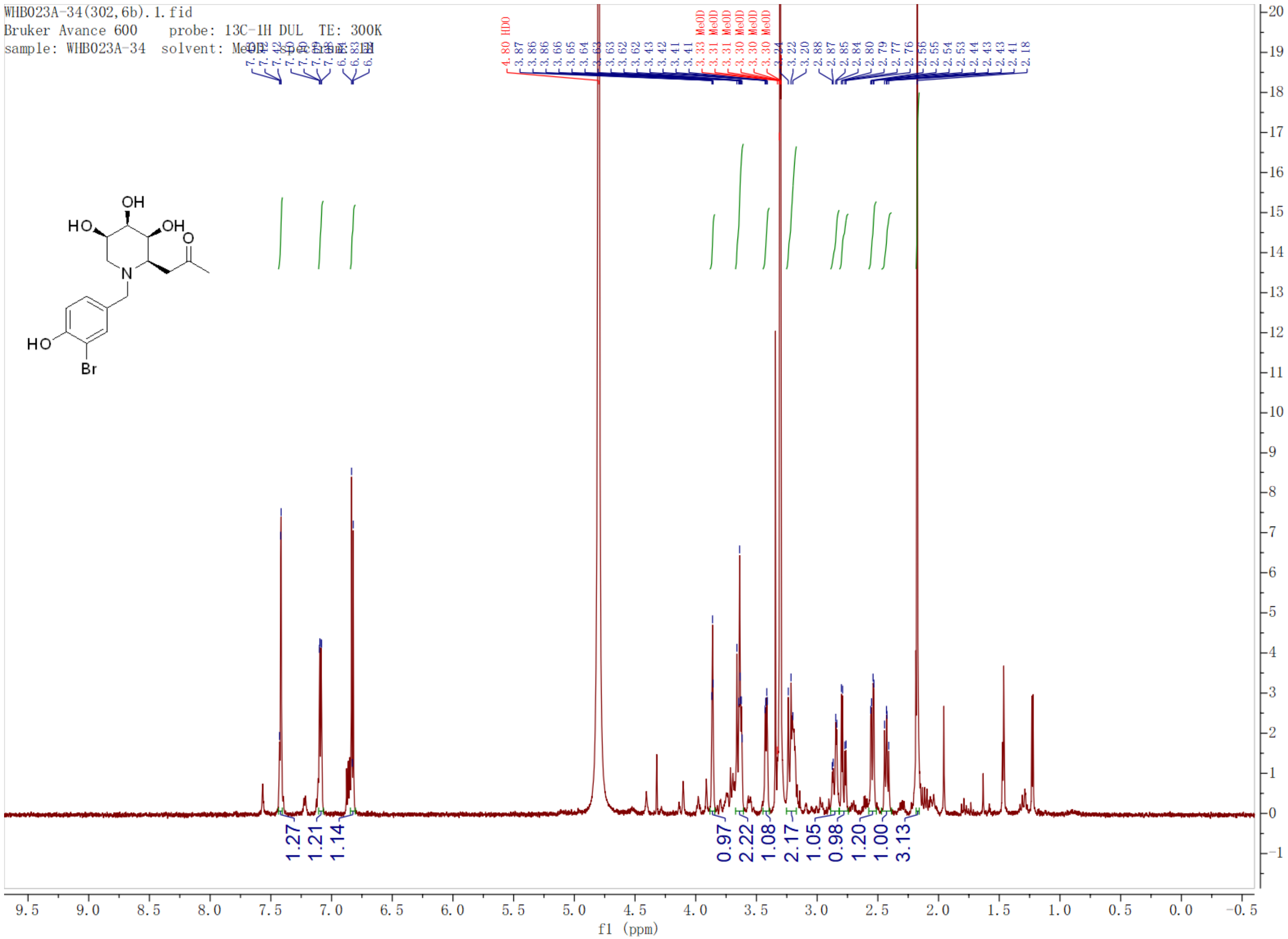

Figure S122. The ¹H NMR of compound **6b**.

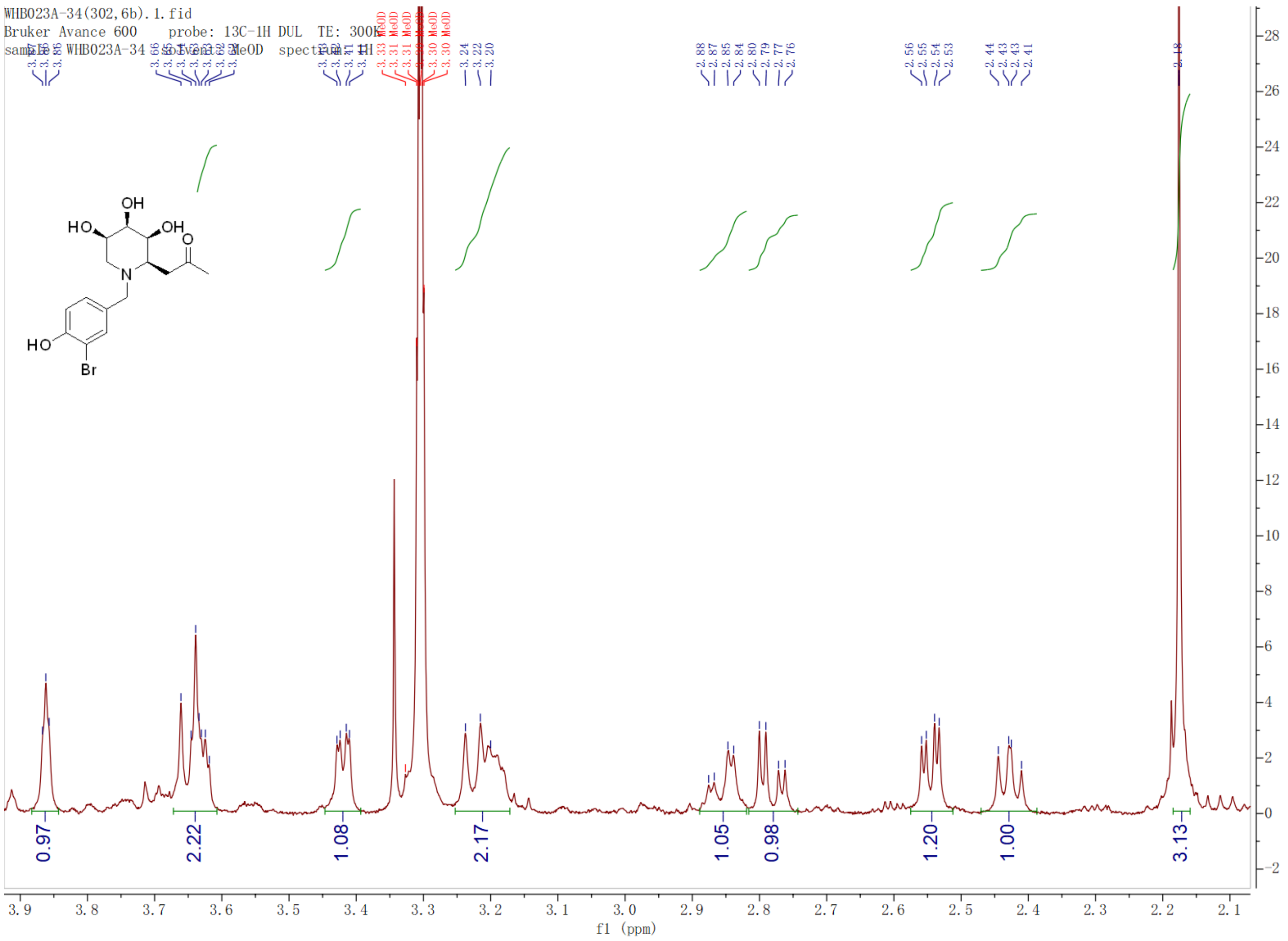

Figure S123. Partial region amplification of the ^{1}H NMR of compound **6b**.

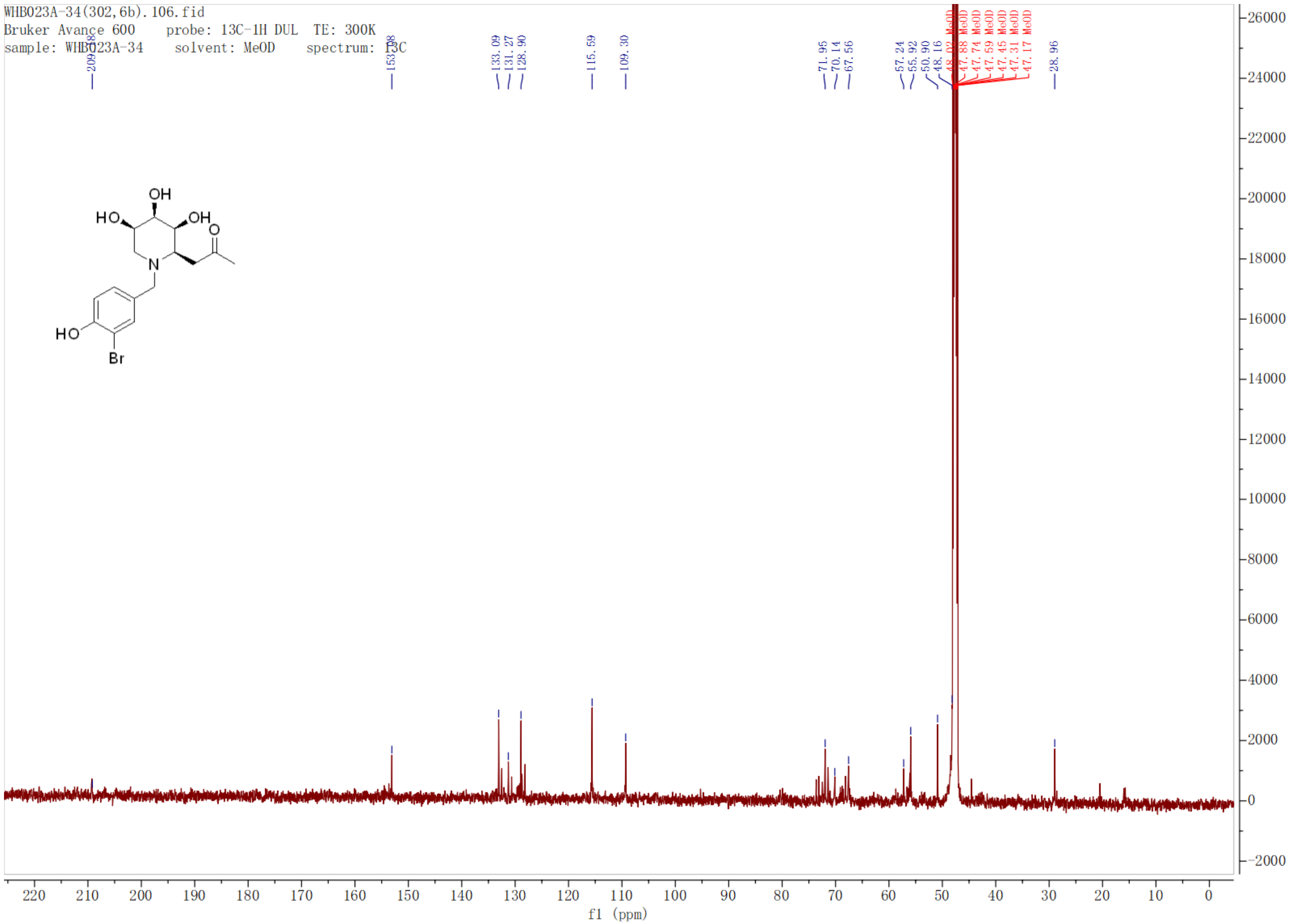

Figure S124. The ^{13}C NMR of compound **6b**.

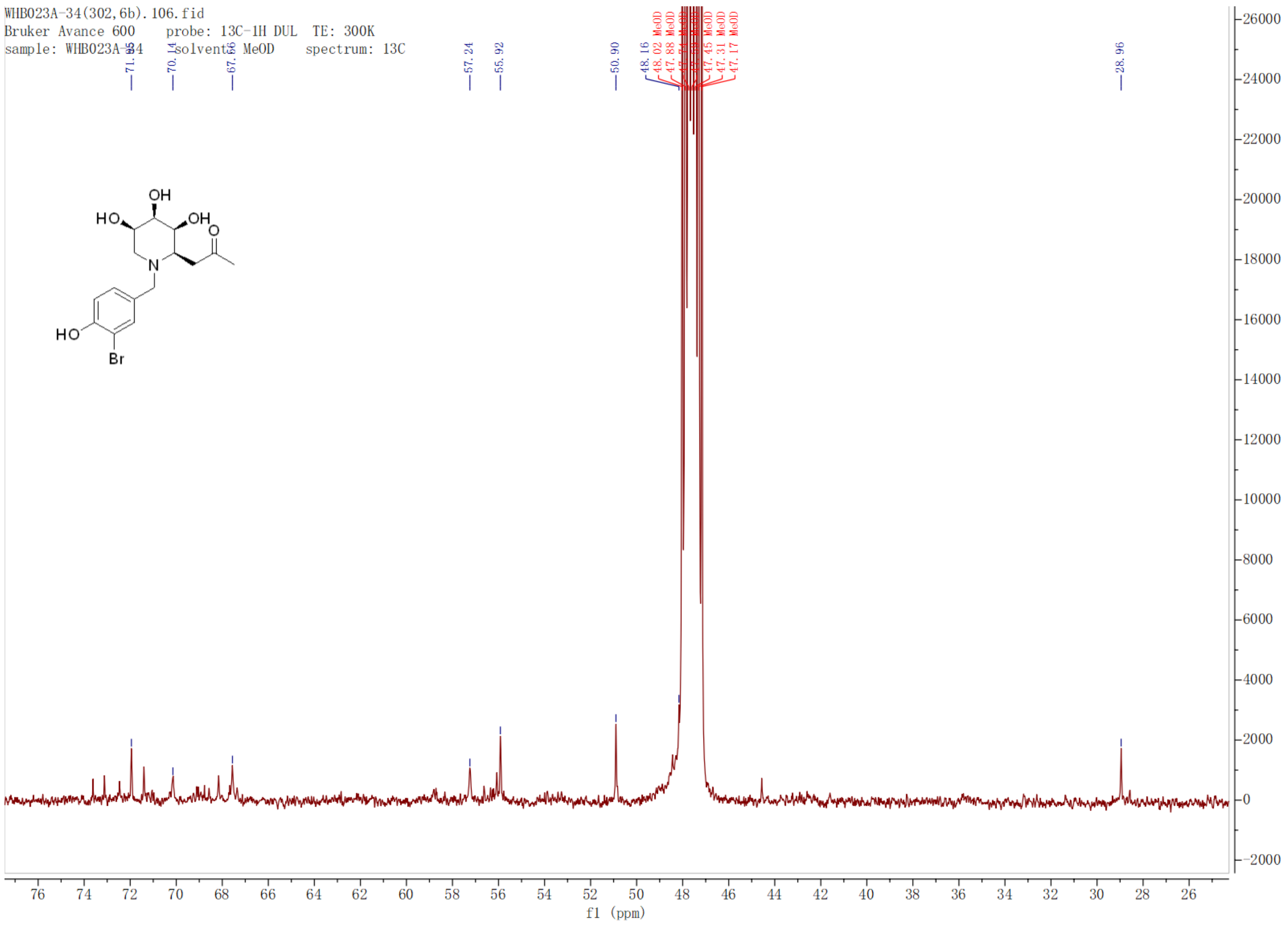

Figure S125. Partial region amplification of the ^{13}C NMR of compound **6b**.

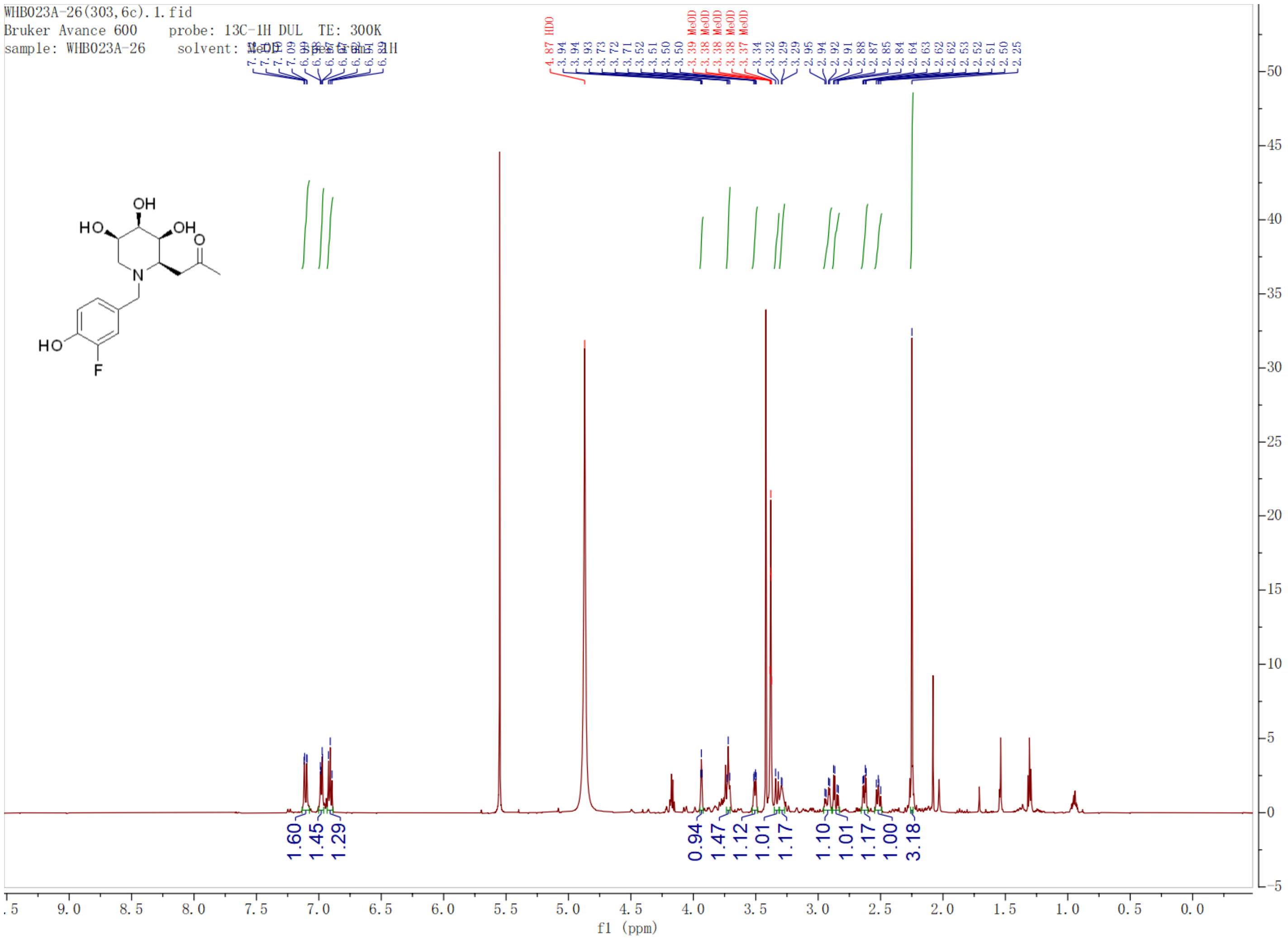

Figure S126. The ^{1}H NMR of compound **6c**.

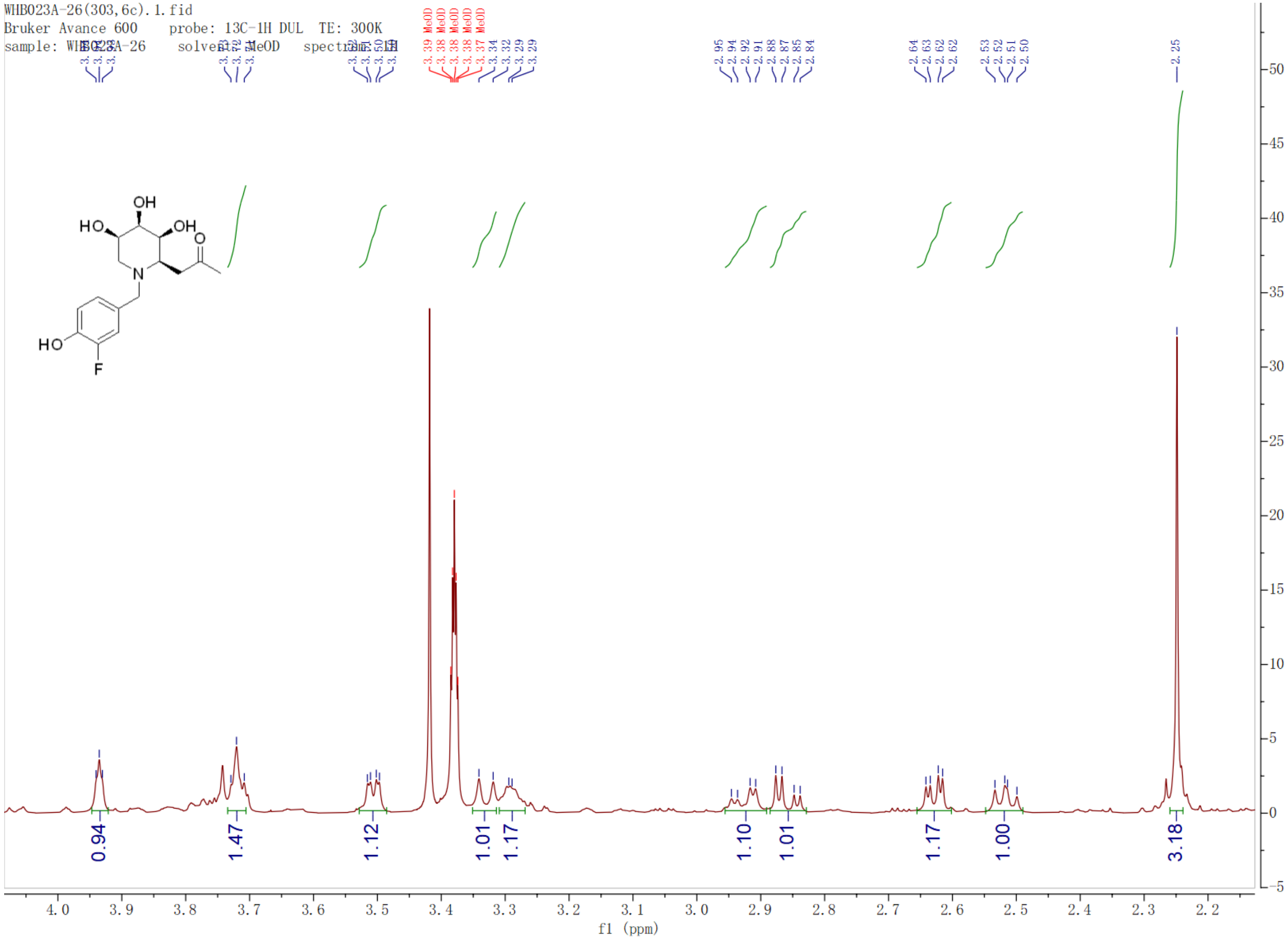

Figure S127. Partial region amplification of the ^{1}H NMR of compound **6c**.

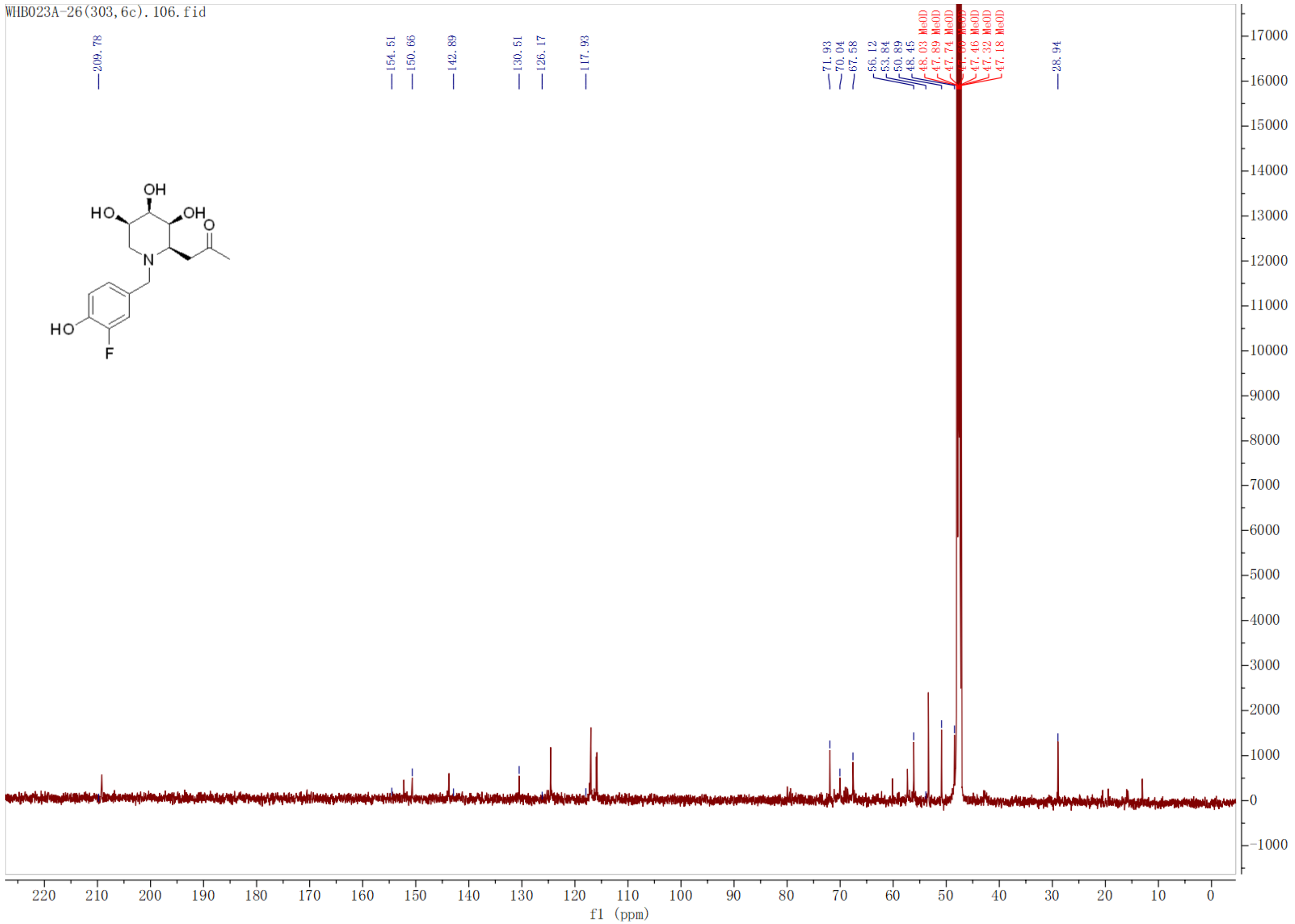

Figure S128. The ^{13}C NMR of compound **6c**.

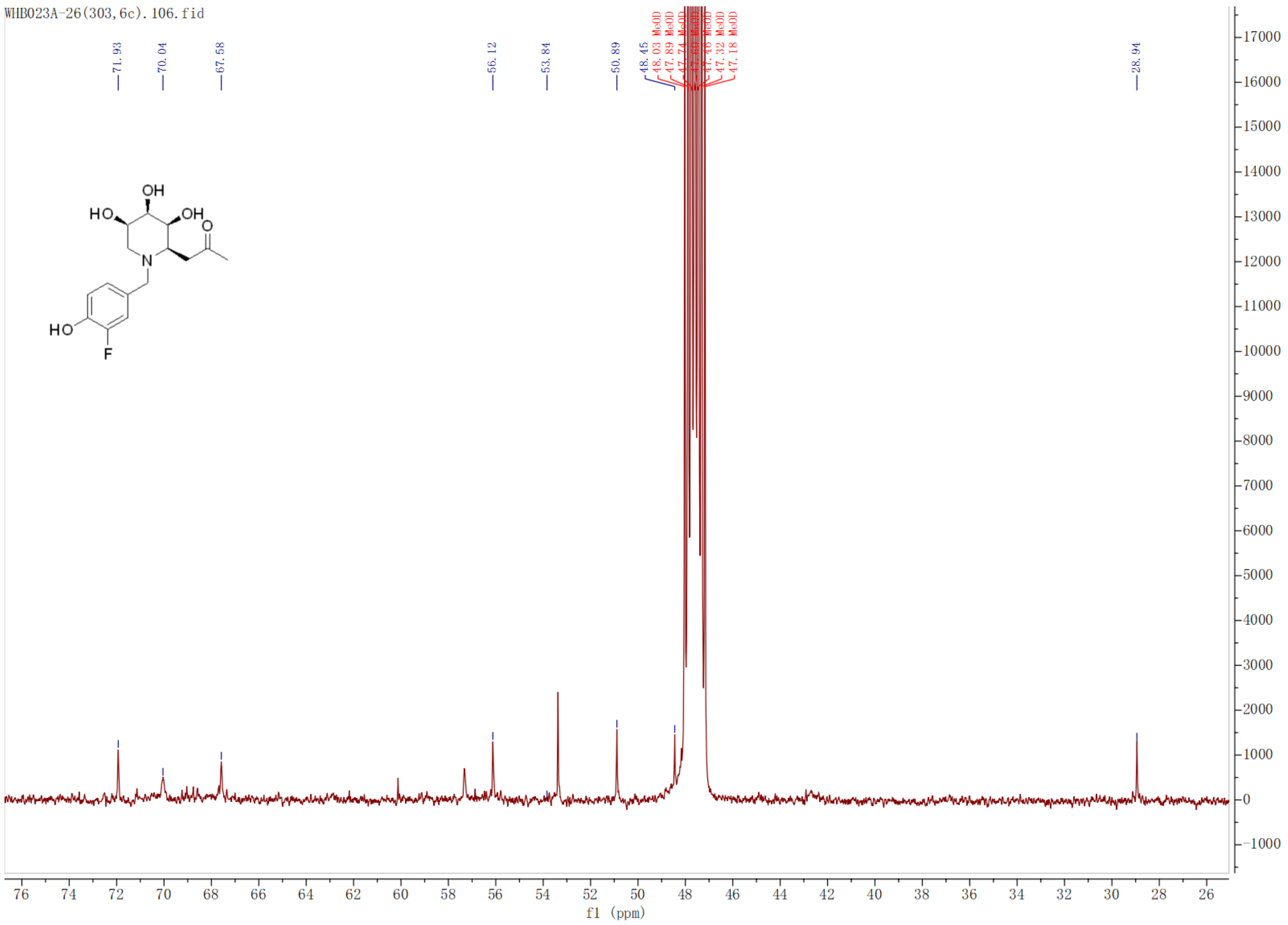

Figure S129. Partial region amplification of the ^{13}C NMR of compound **6c**.

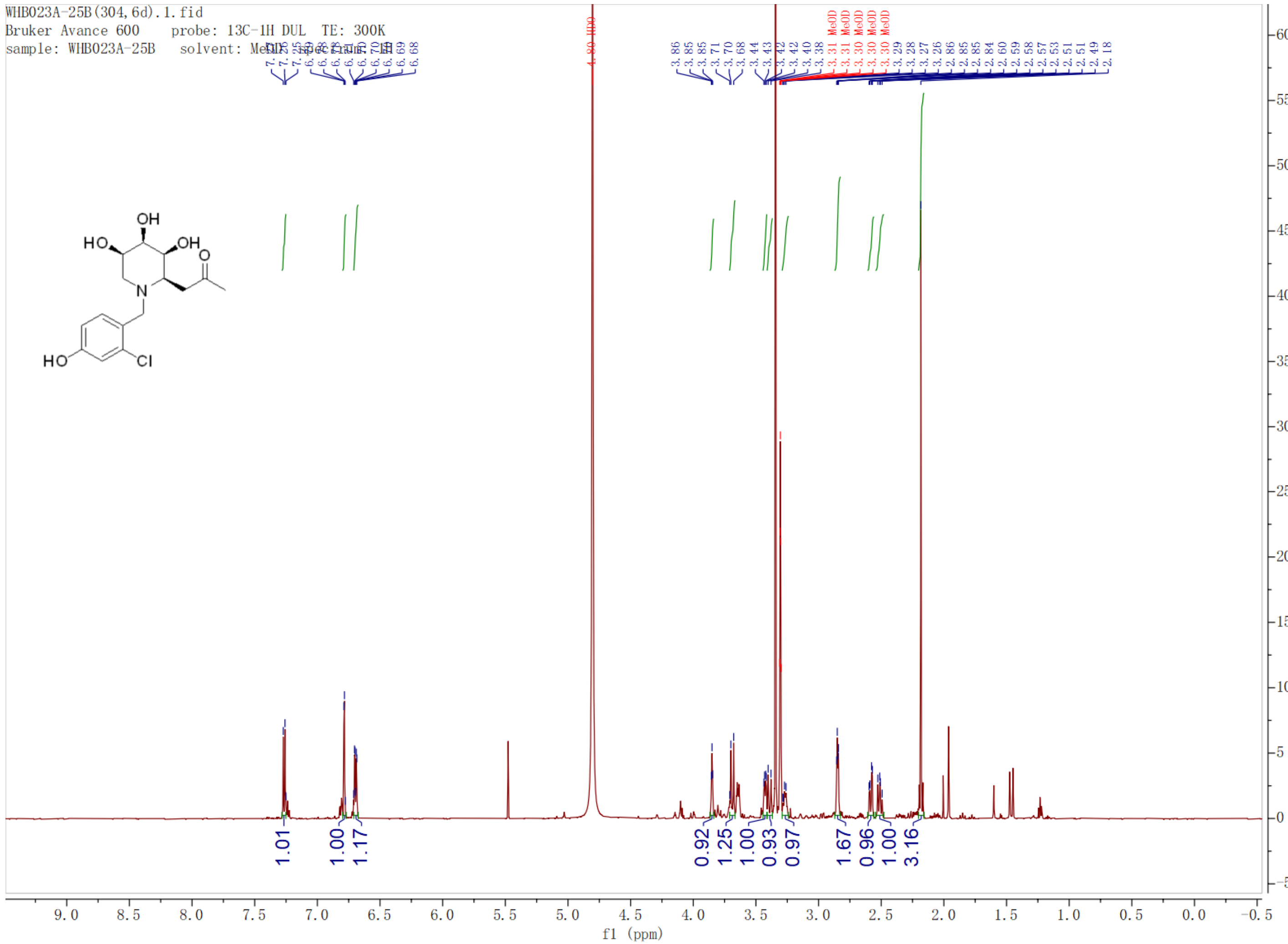

Figure S130. The ¹H NMR of compound **6d**.

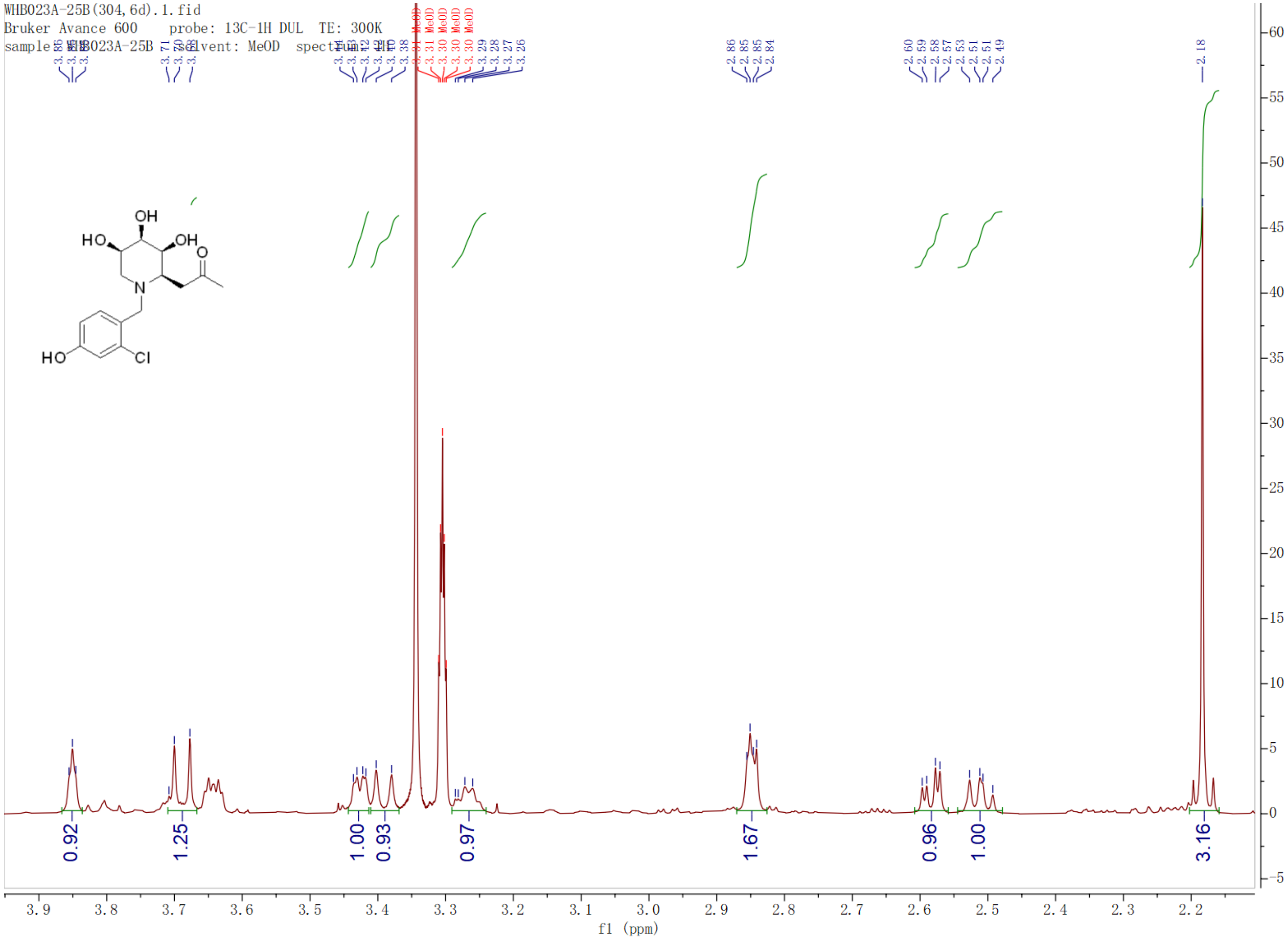

Figure S131. Partial region amplification of the ^{1}H NMR of compound **6d**.

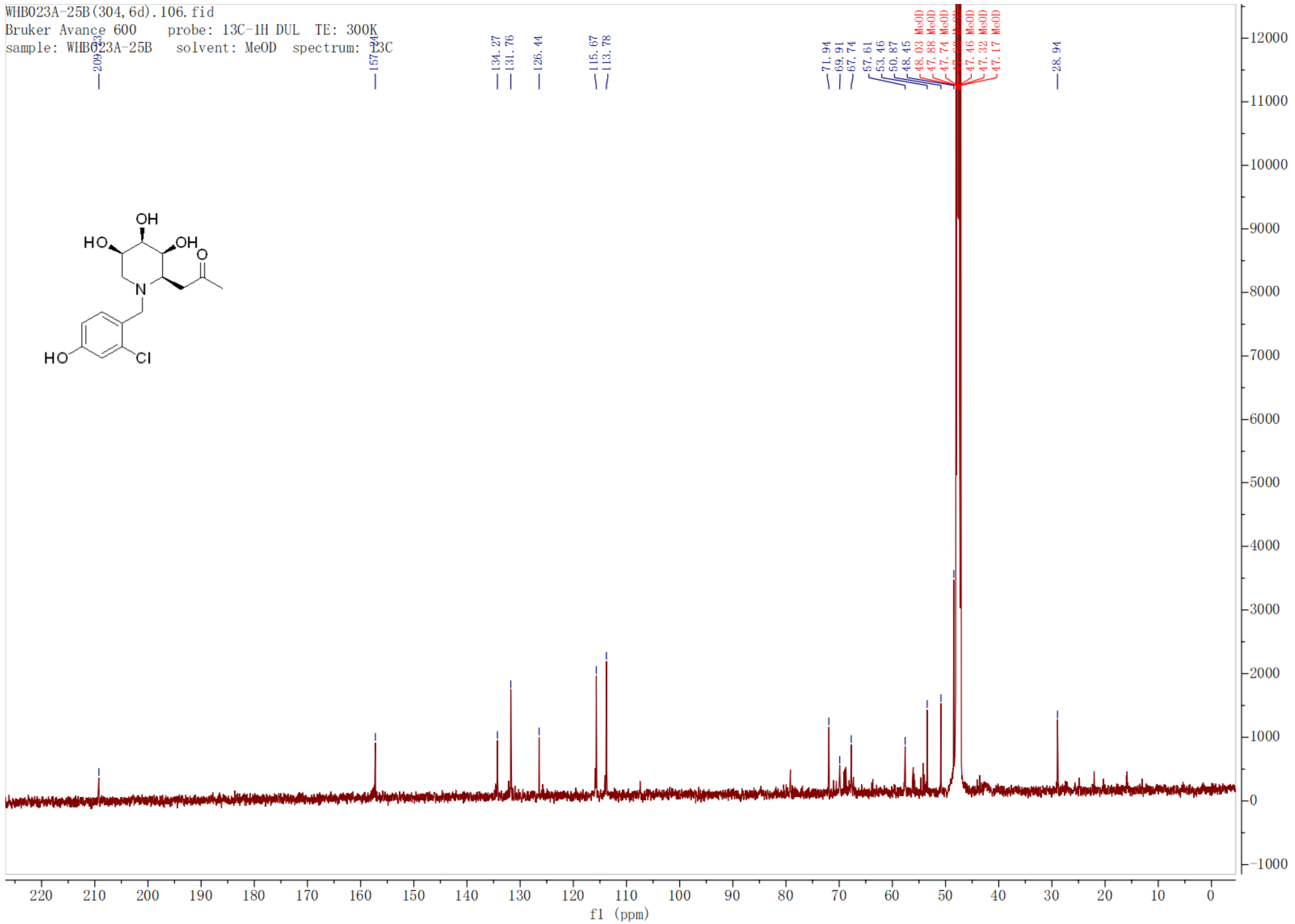

Figure S132. The ^{13}C NMR of compound **6d**.

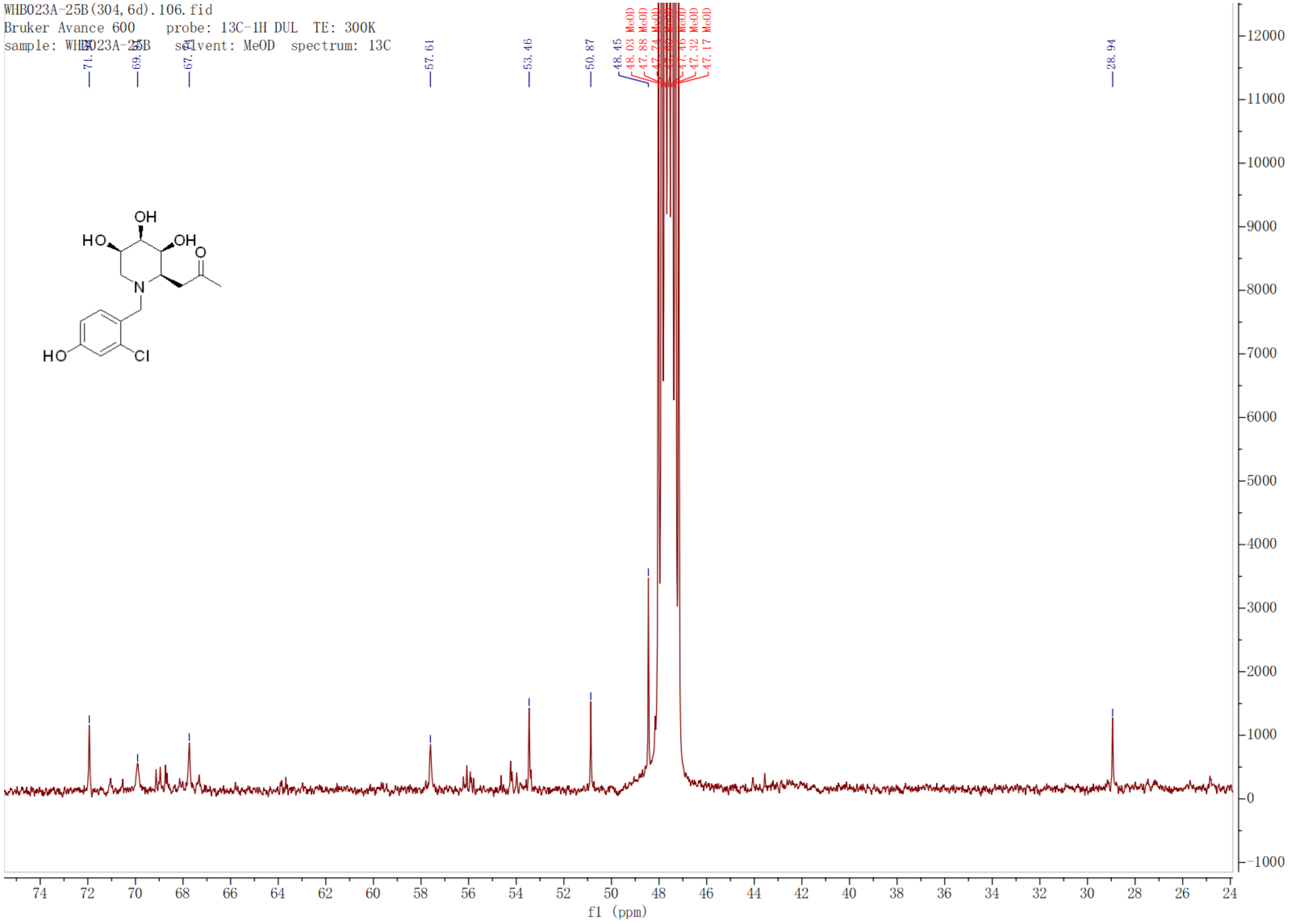

Figure S133. Partial region amplification of the ^{13}C NMR of compound **6d**.

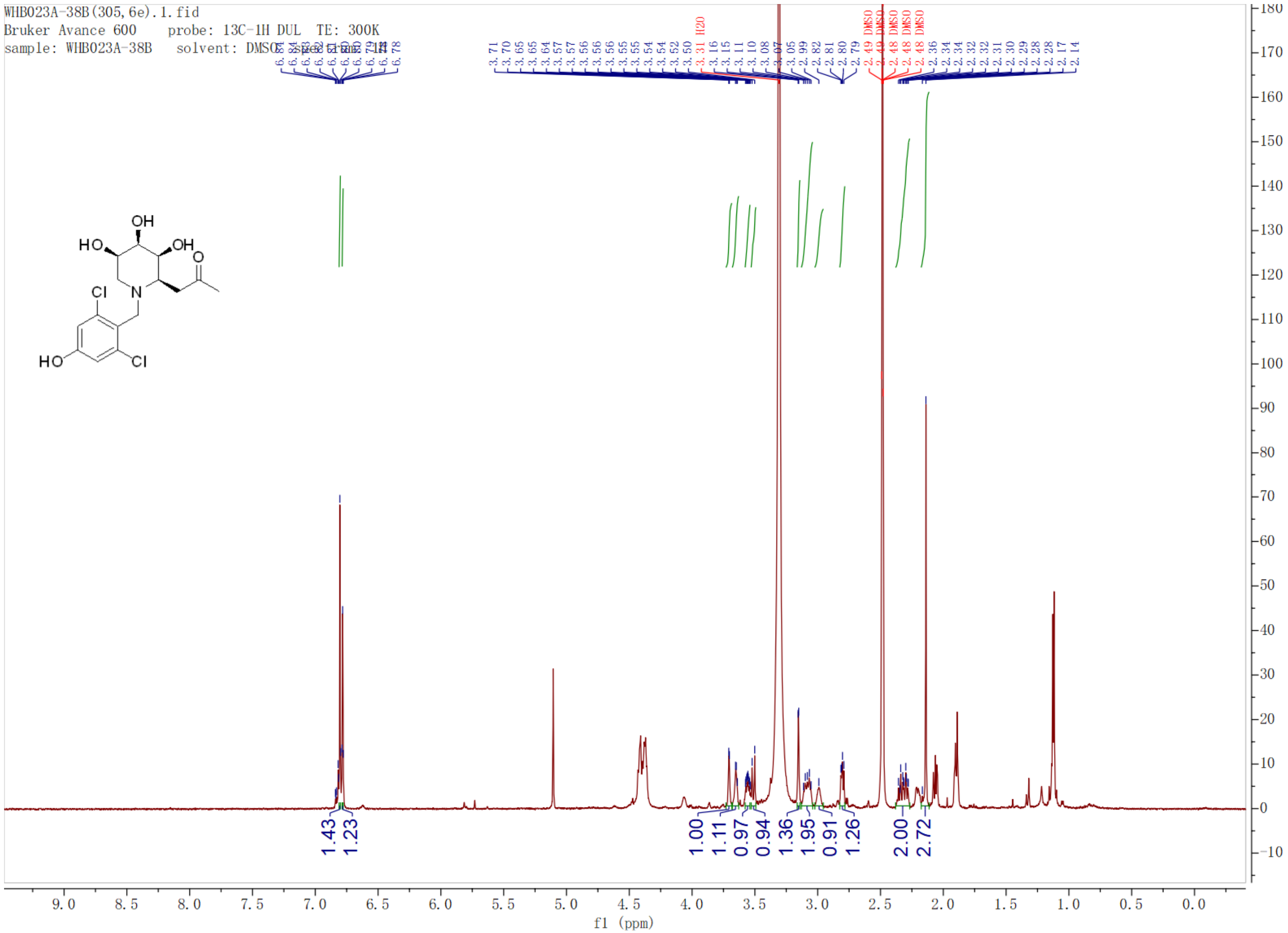

Figure S134. The ^{1}H NMR of compound **6e**.

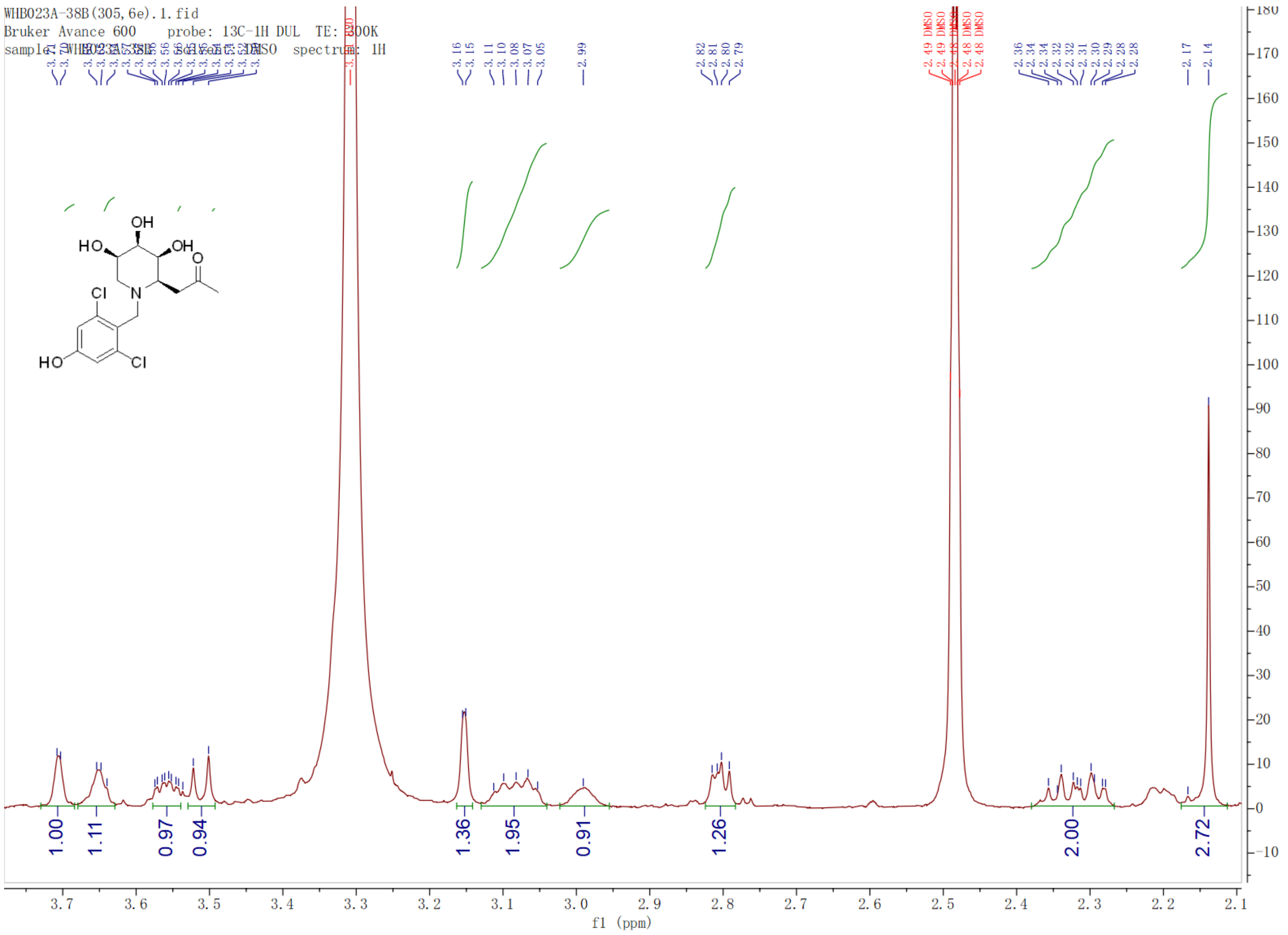

Figure S135. Partial region amplification of the ¹H NMR of compound **6e**.

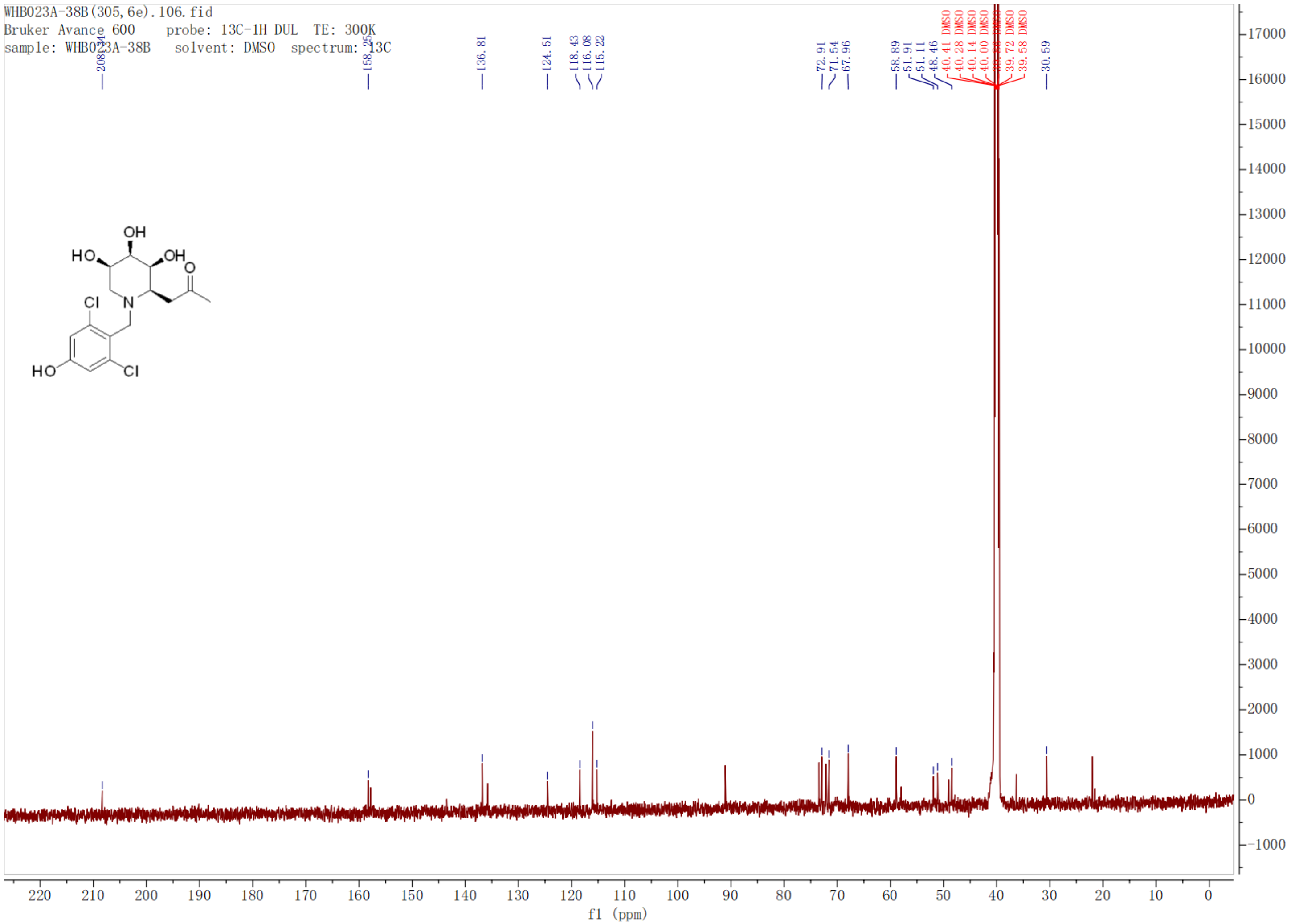

Figure S136. The ^{13}C NMR of compound **6e**.

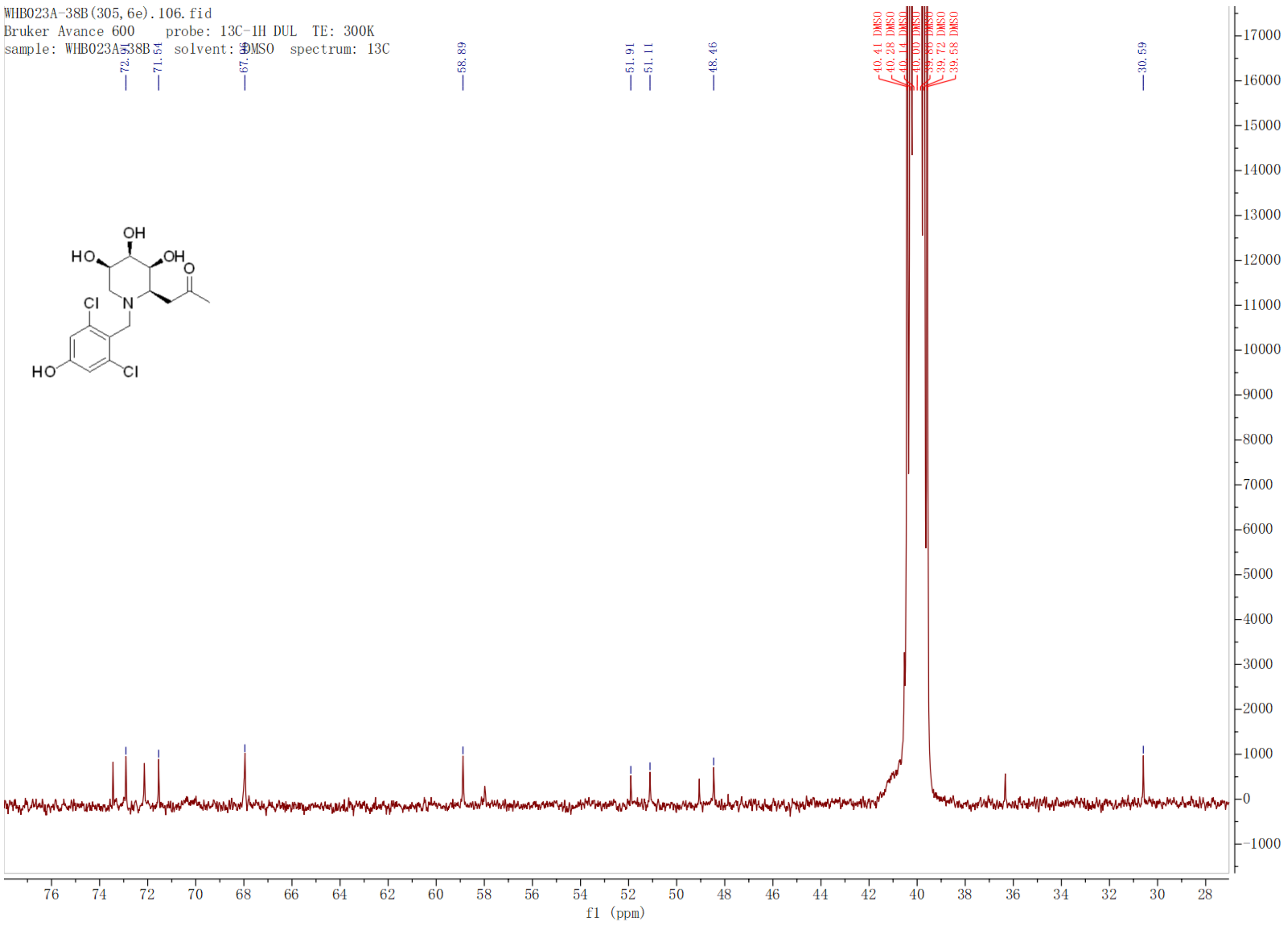

Figure S137. Partial region amplification of the ^{13}C NMR of compound **6e**.

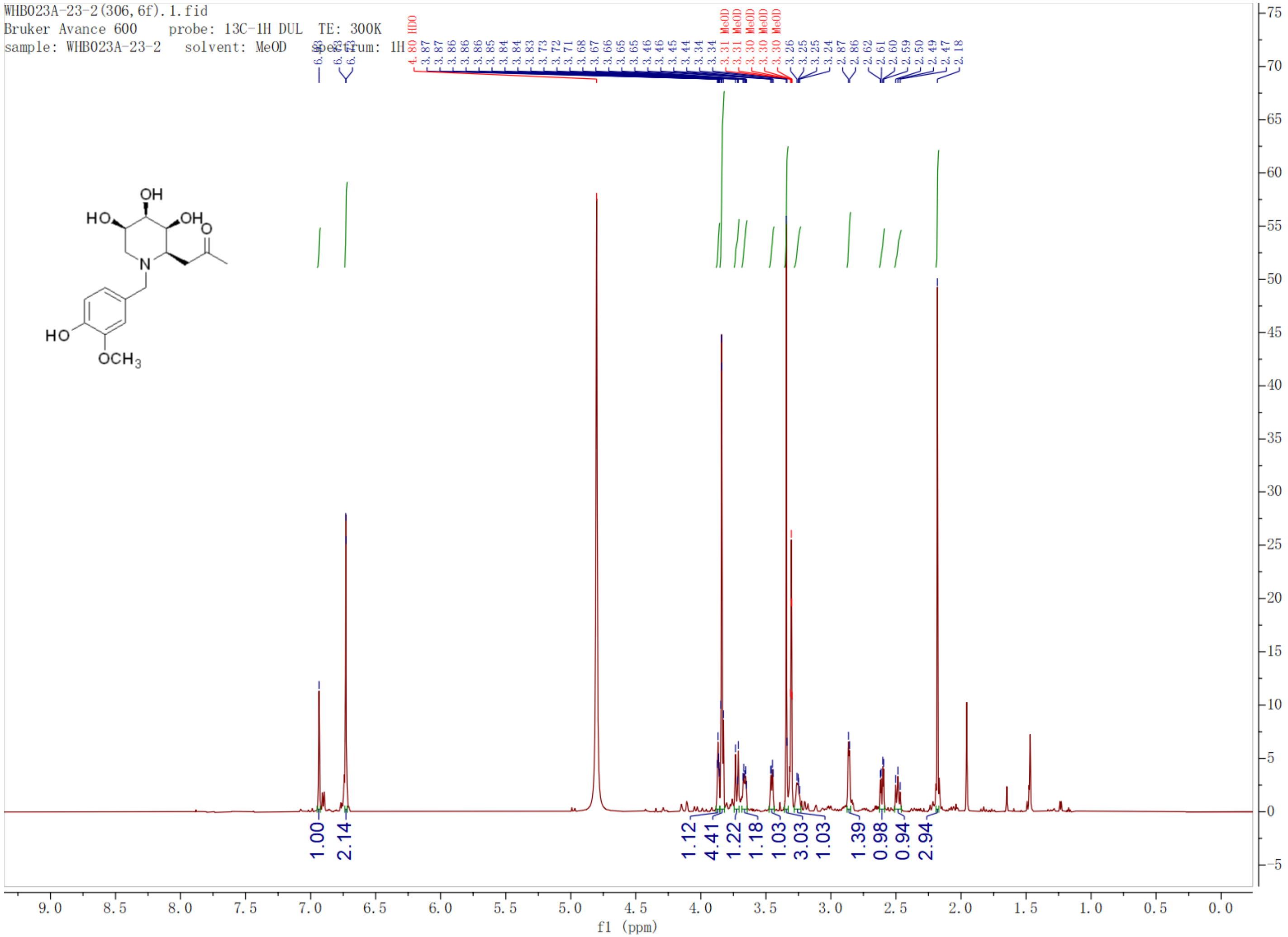

Figure S138. The ^{1}H NMR of compound **6f**.

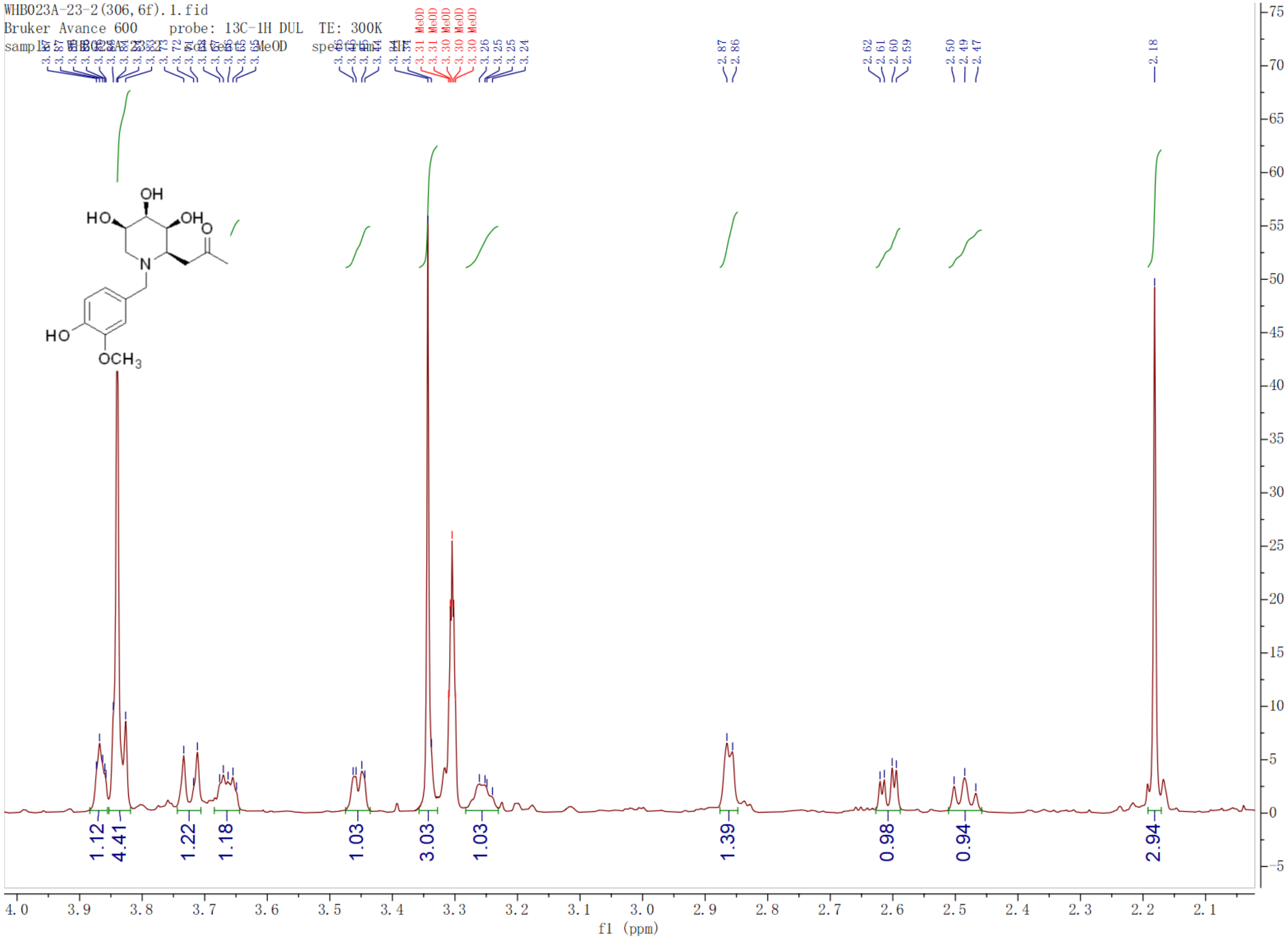

Figure S139. Partial region amplification of the ^{1}H NMR of compound **6f**.

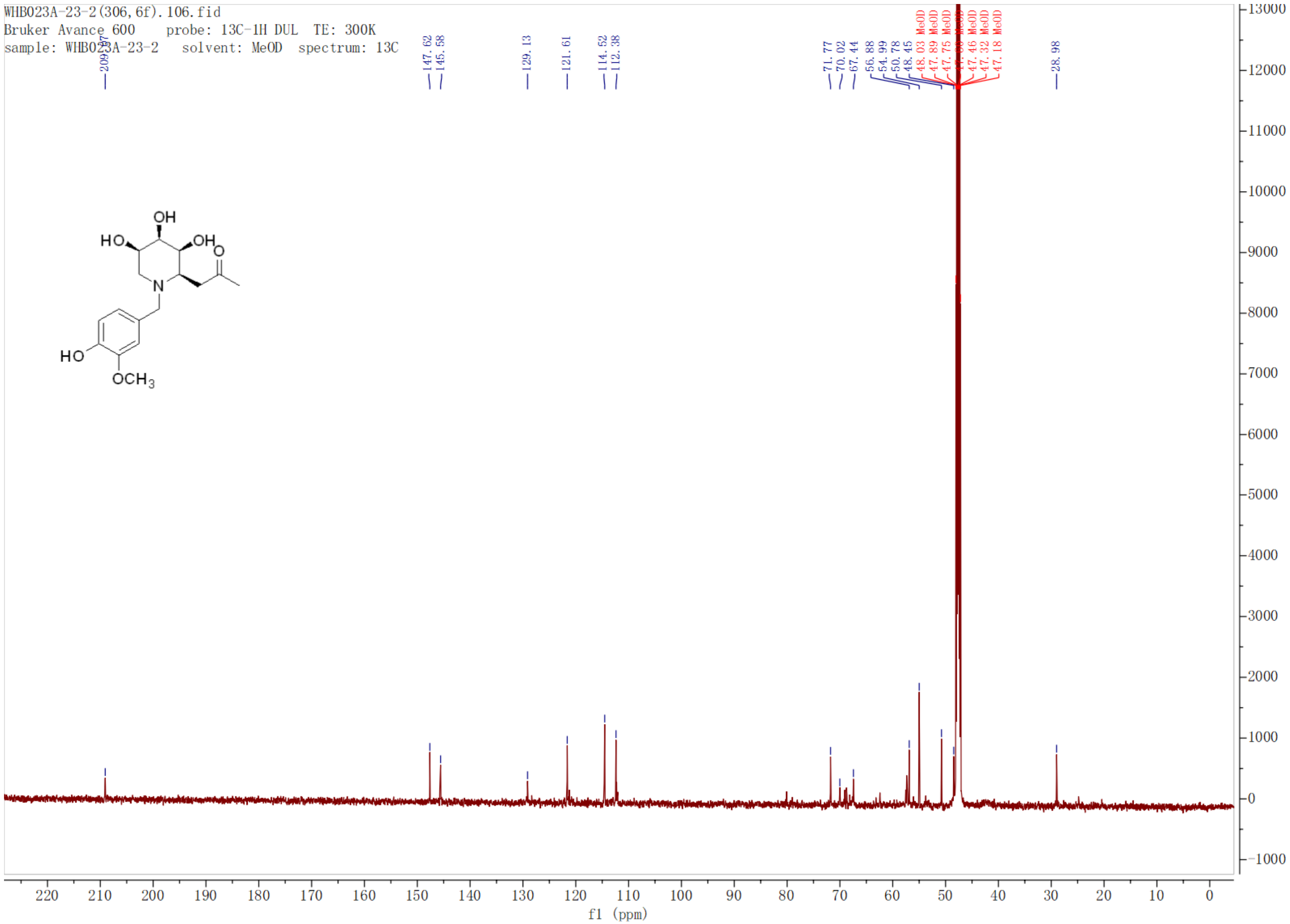

Figure S140. The ^{13}C NMR of compound **6f**.

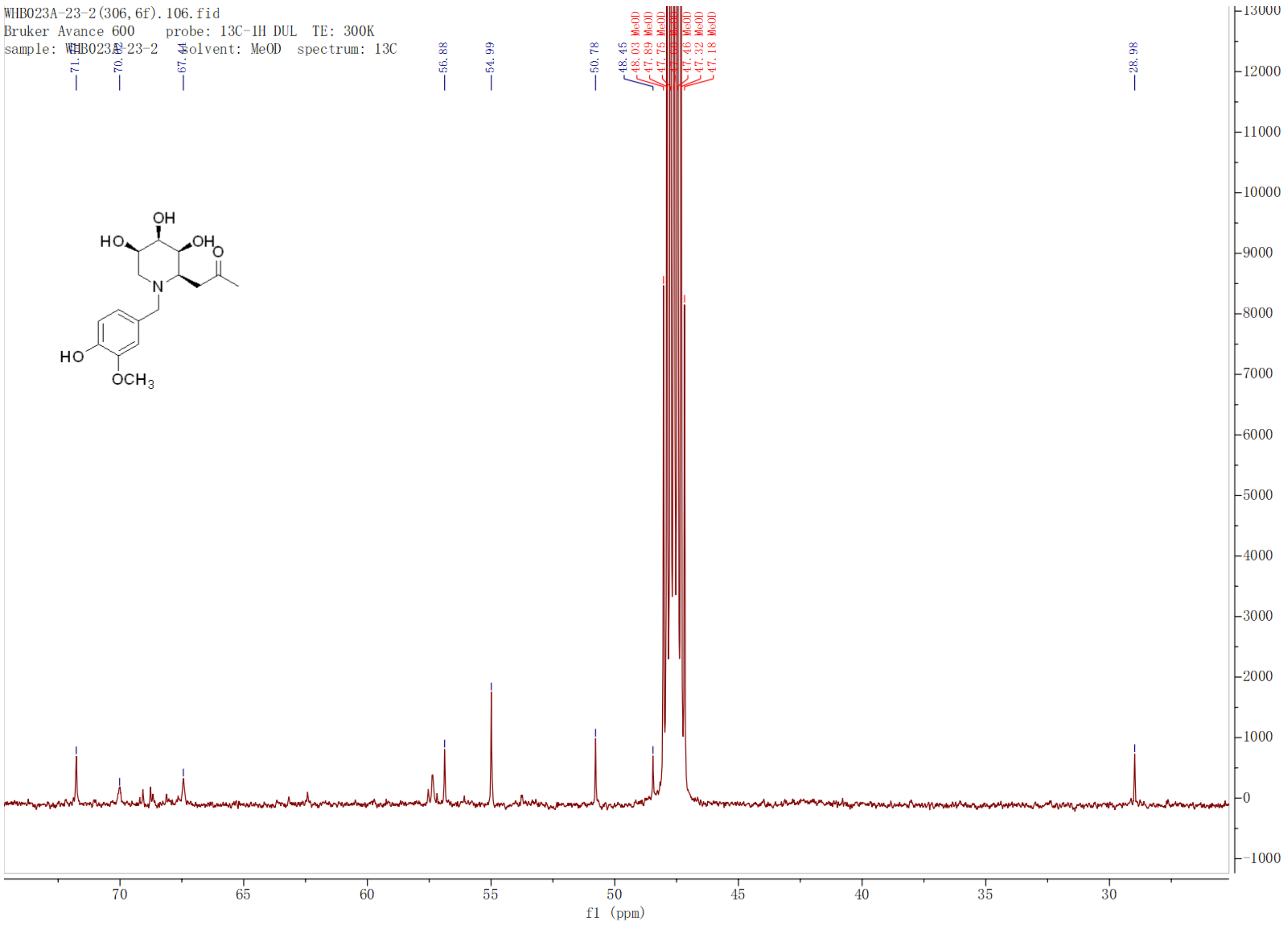

Figure S141. Partial region amplification of the ^{13}C NMR of compound **6f**.

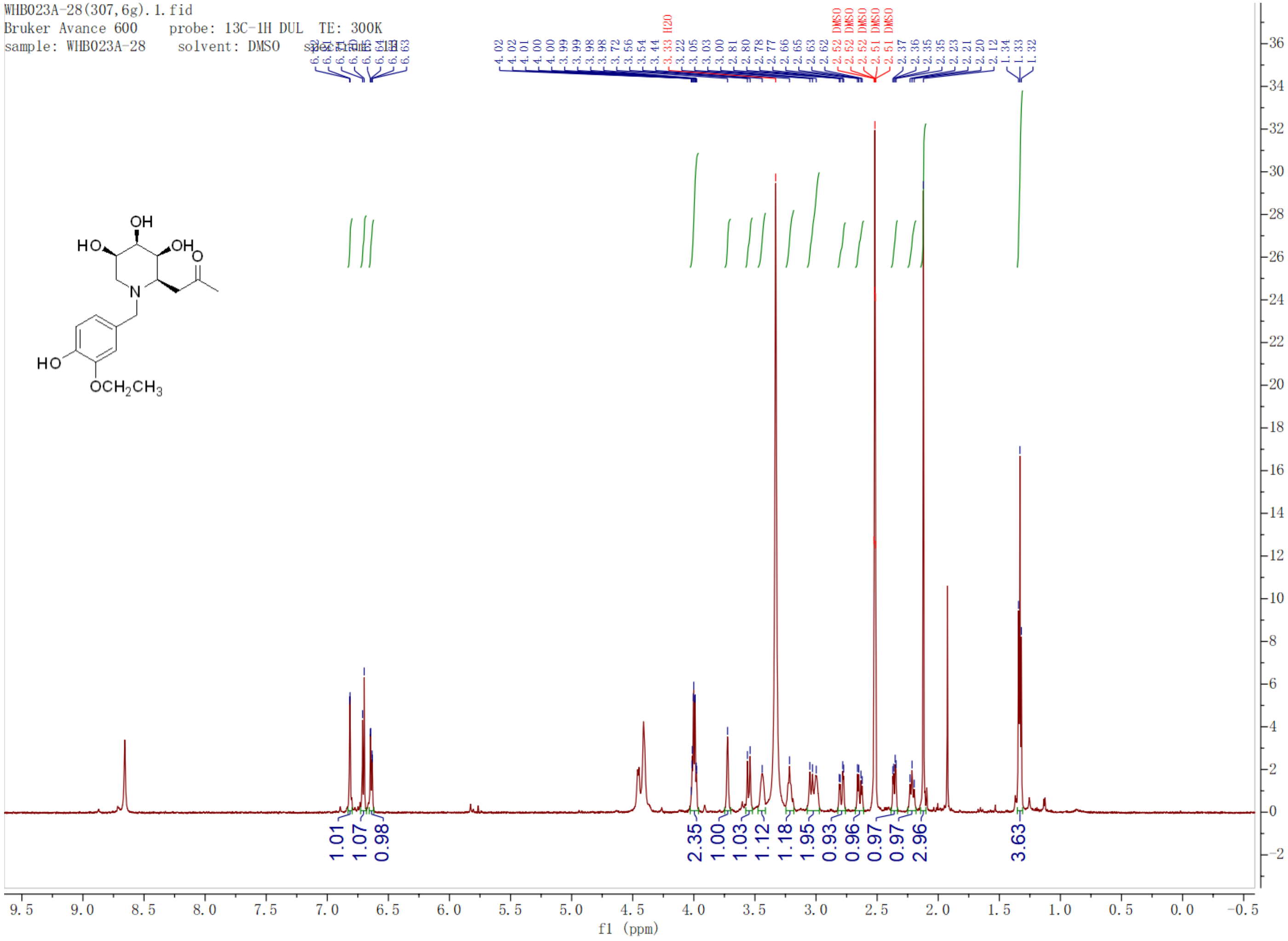

Figure S142. The ^{1}H NMR of compound **6g**.

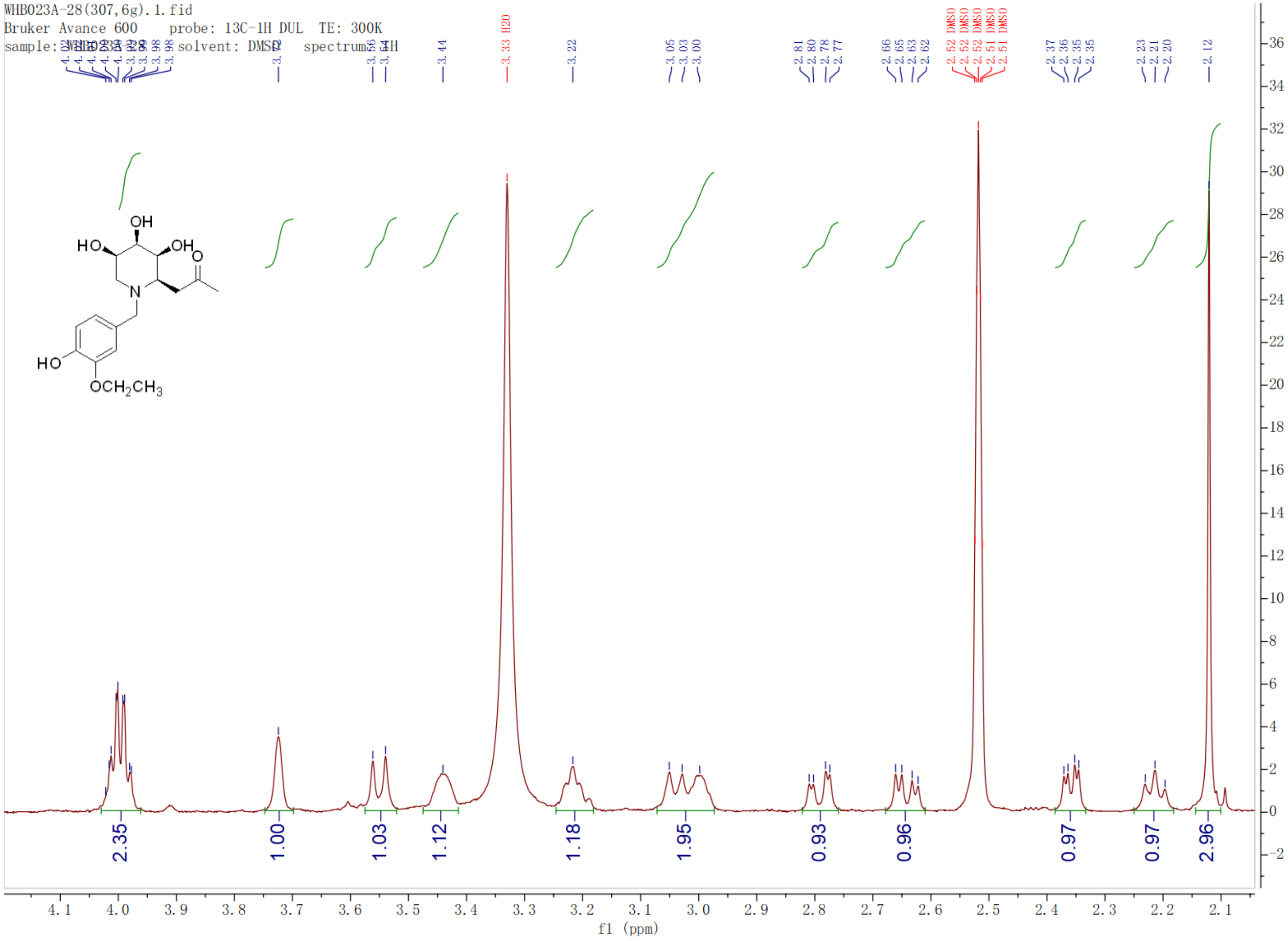

Figure S143. Partial region amplification of the ^{1}H NMR of compound **6g**.

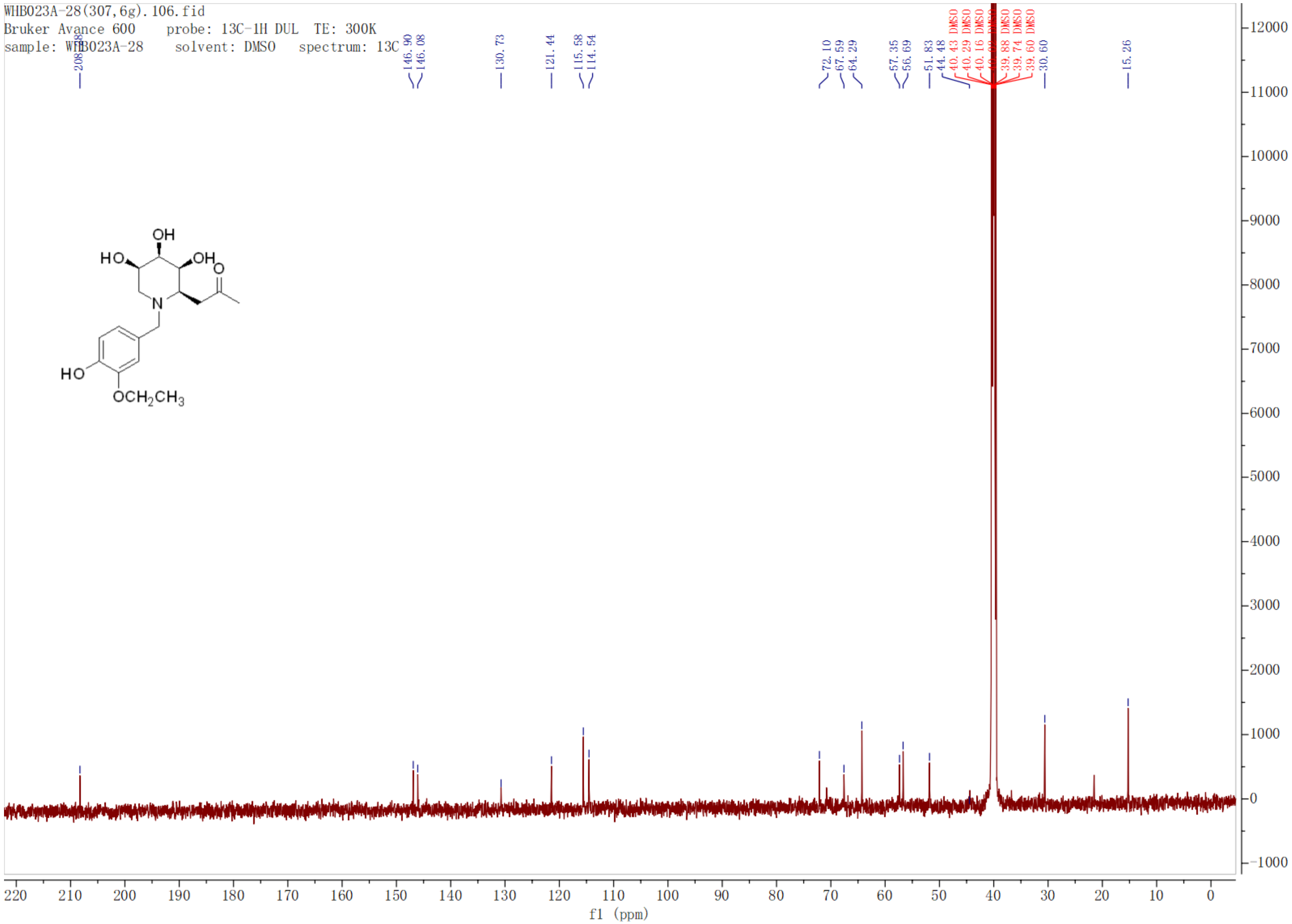

Figure S144. The ^{13}C NMR of compound **6g**.

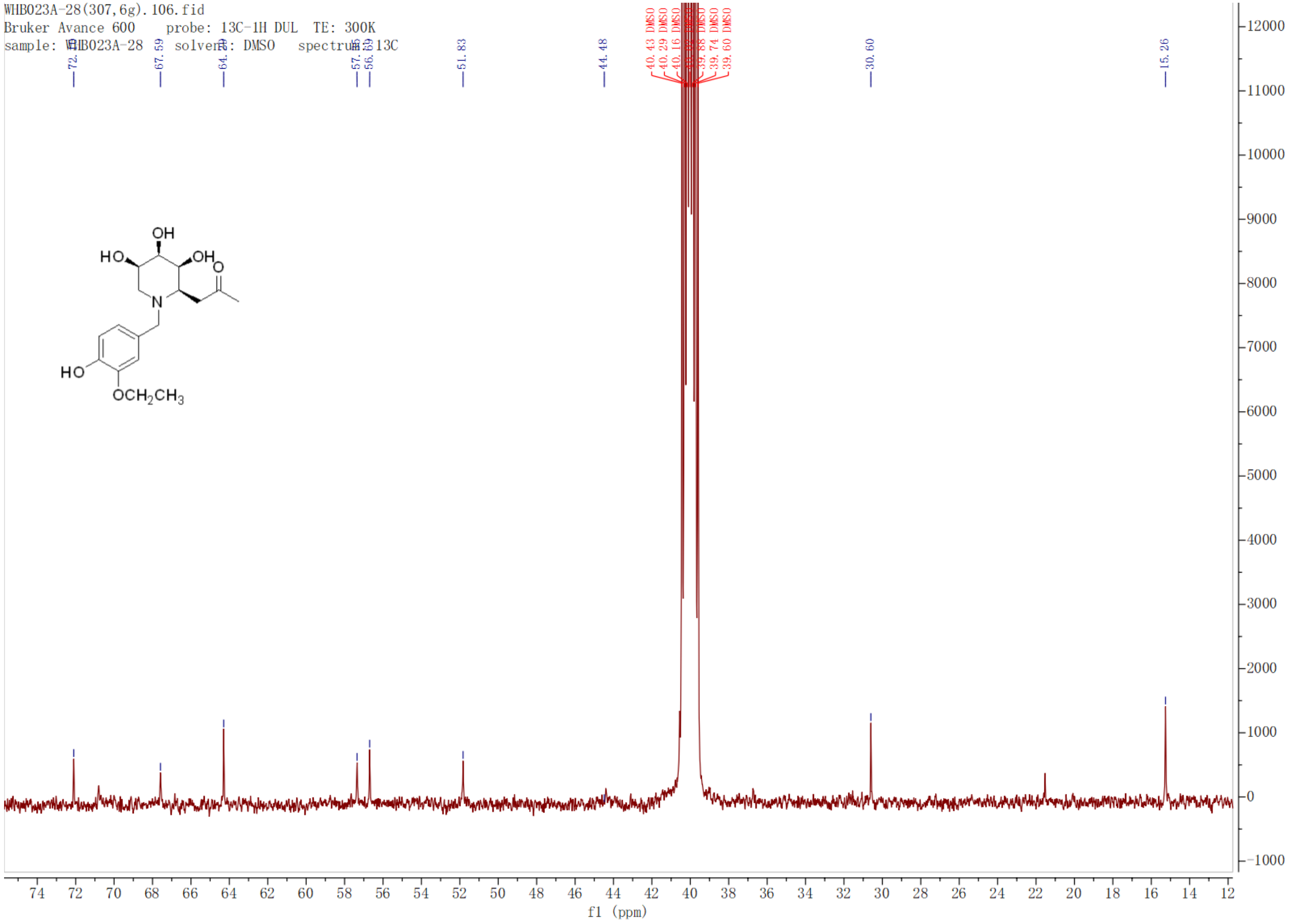

Figure S145. Partial region amplification of the ^{13}C NMR of compound **6g**.

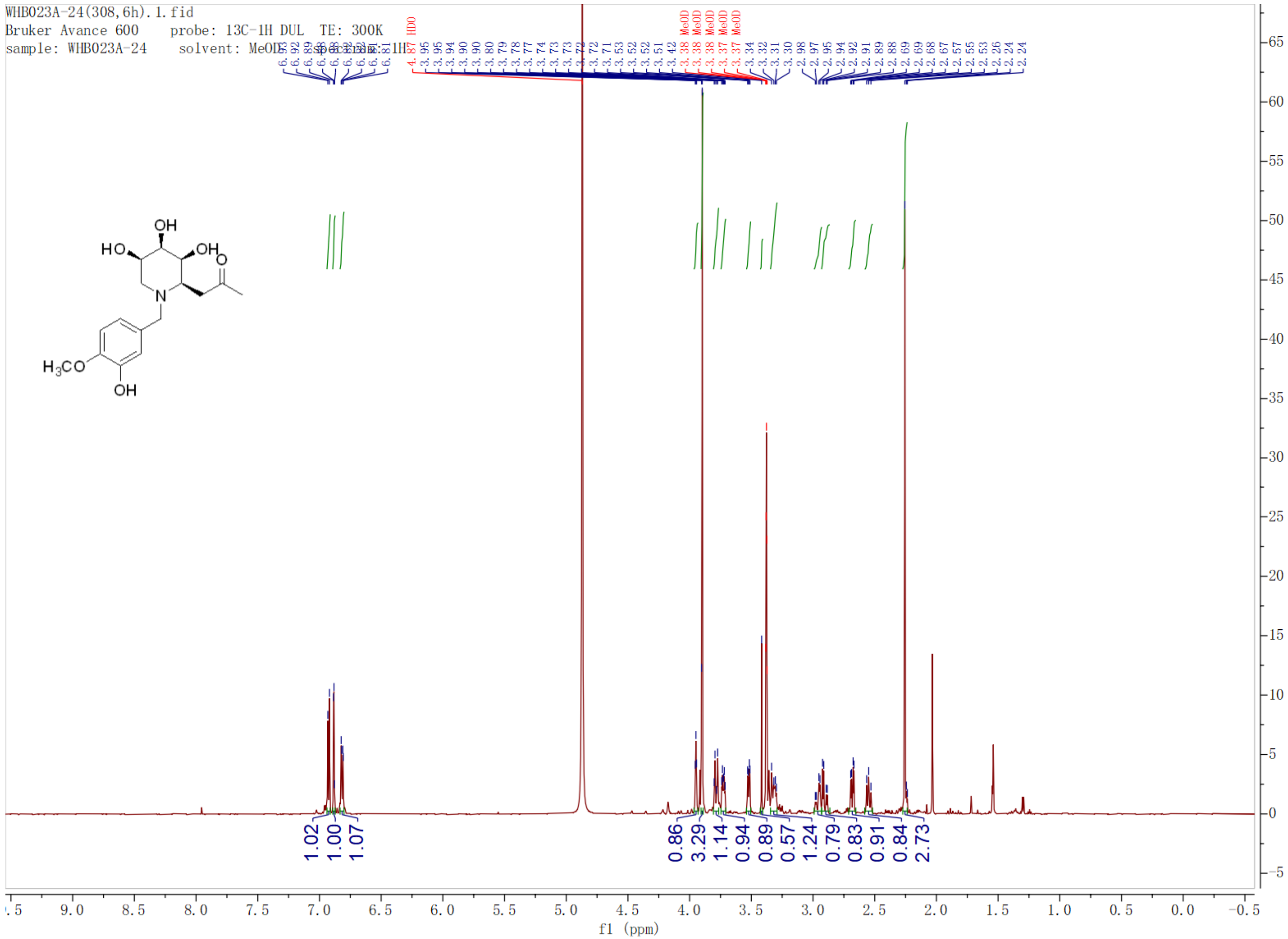

Figure S146. The ¹H NMR of compound **6h**.

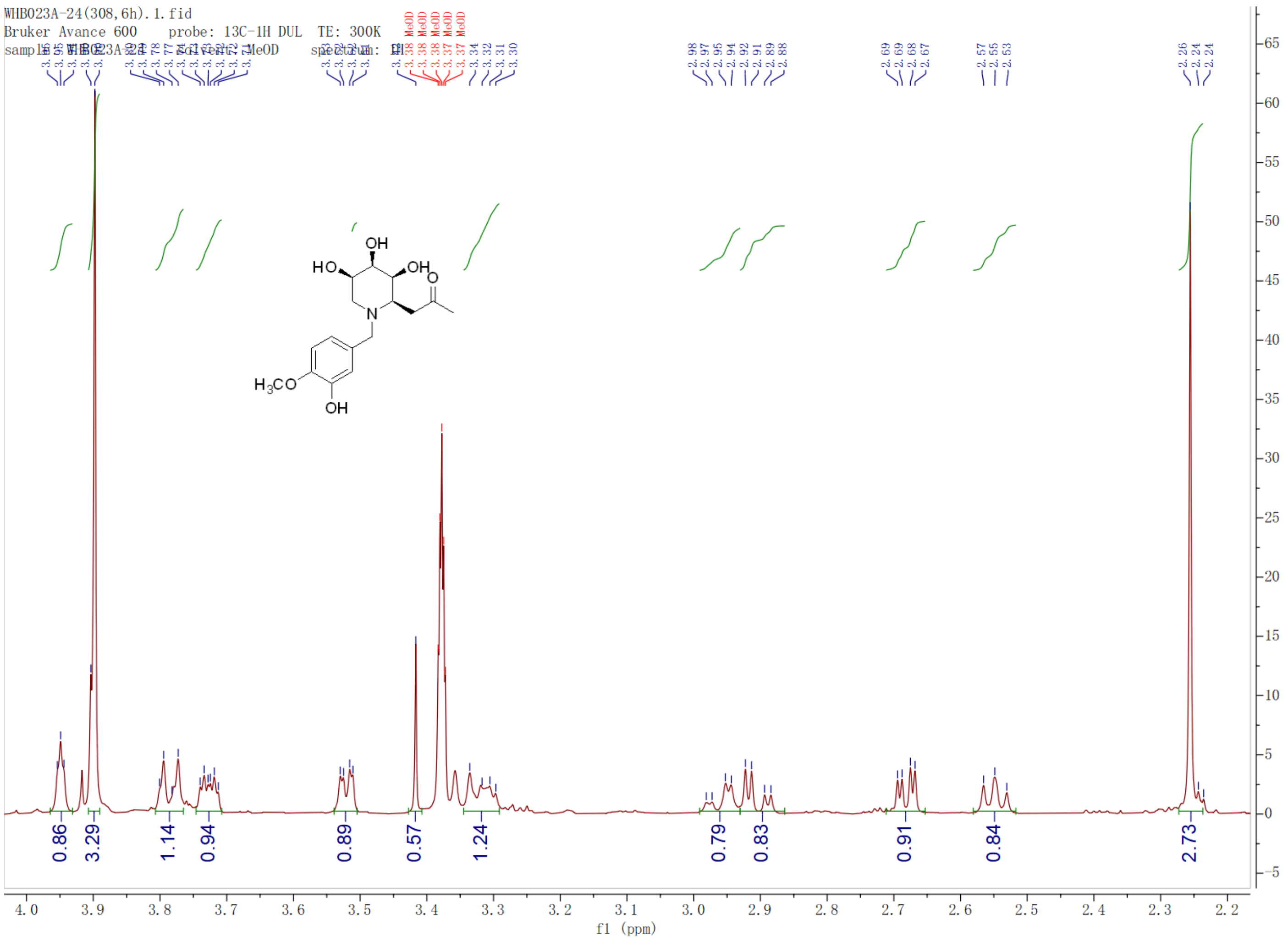

Figure S147. Partial region amplification of the ^{1}H NMR of compound **6h**.

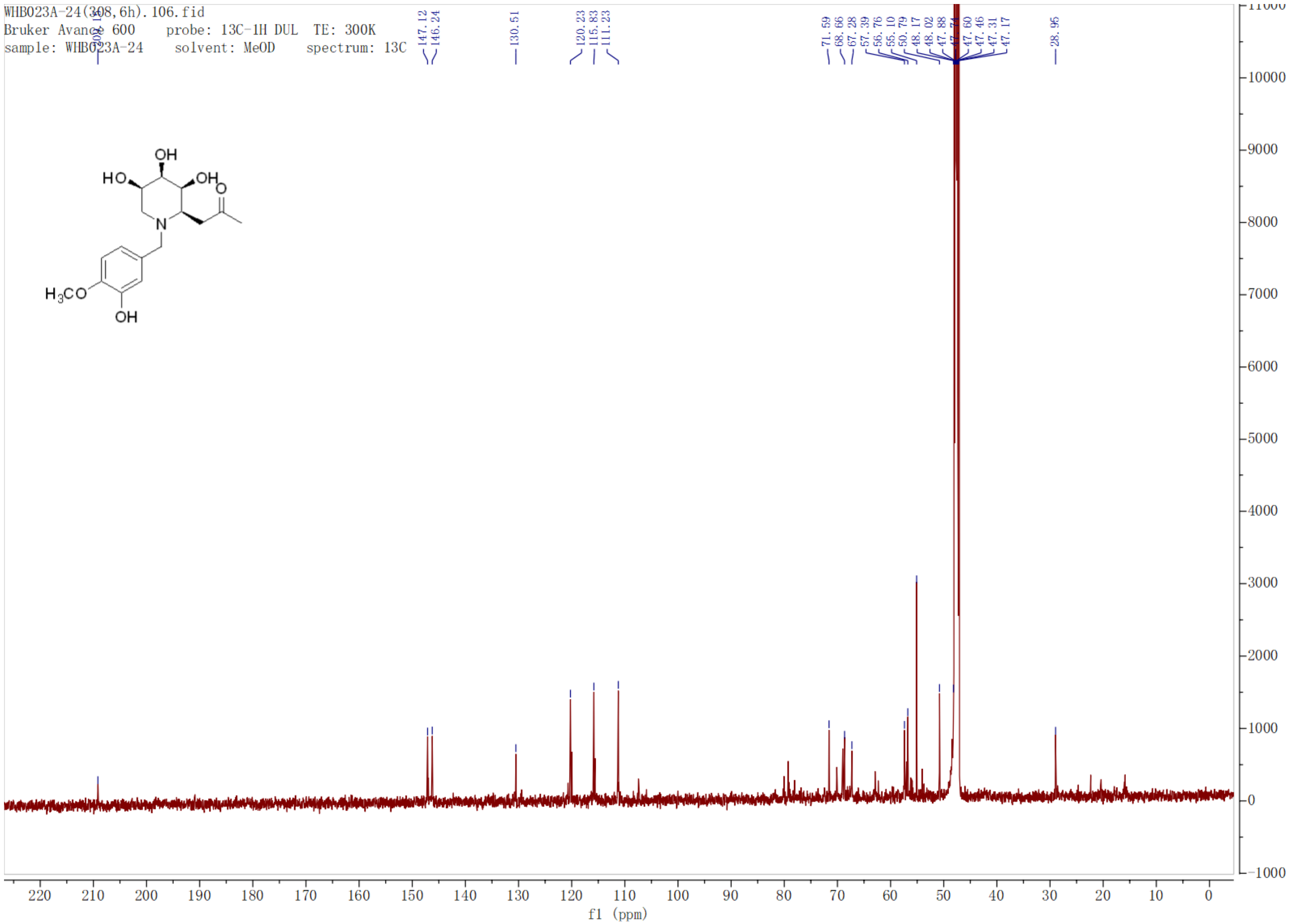

Figure S148. The ^{13}C NMR of compound **6h**.

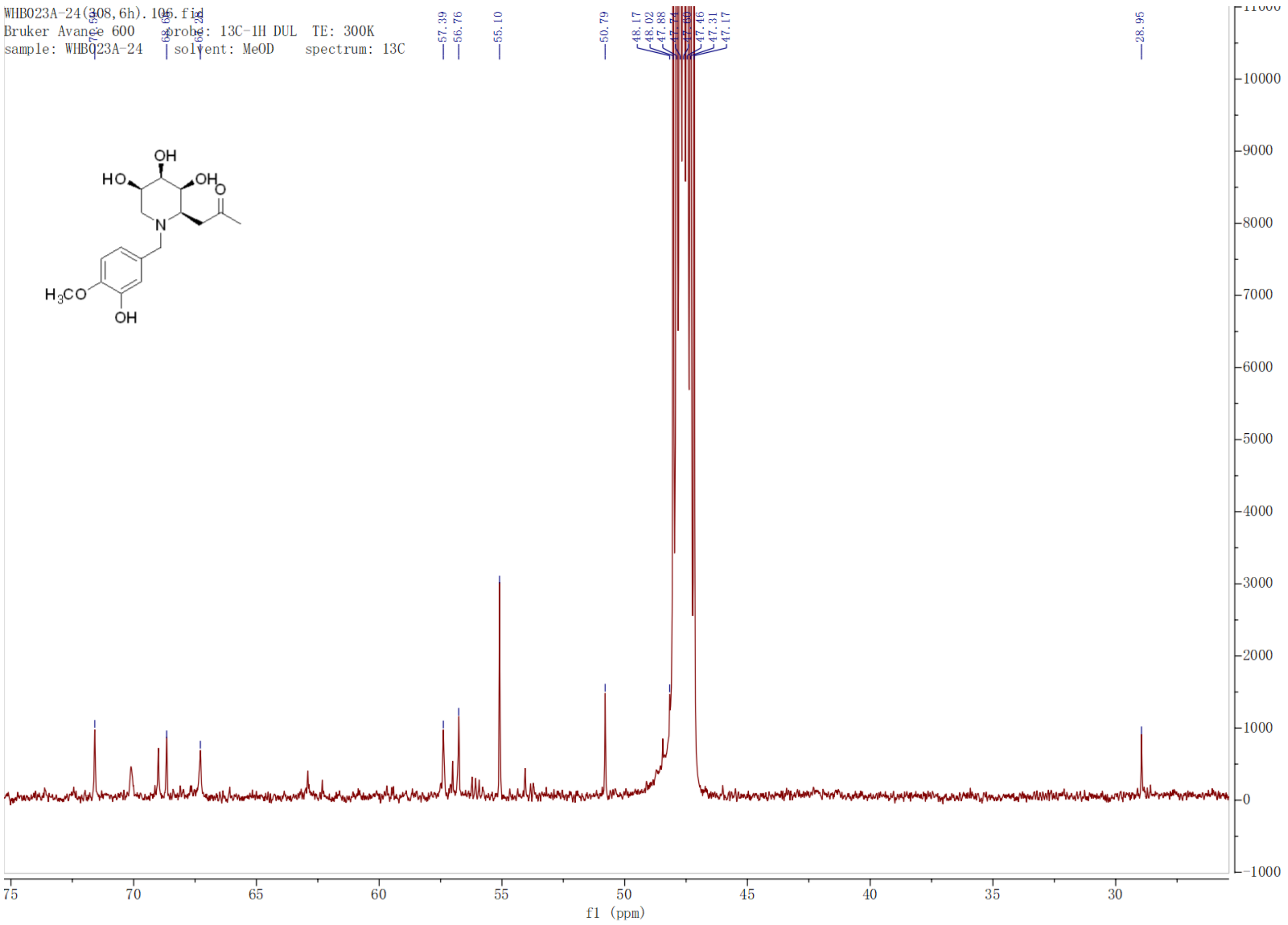

Figure S149. Partial region amplification of the ^{13}C NMR of compound **6h**.

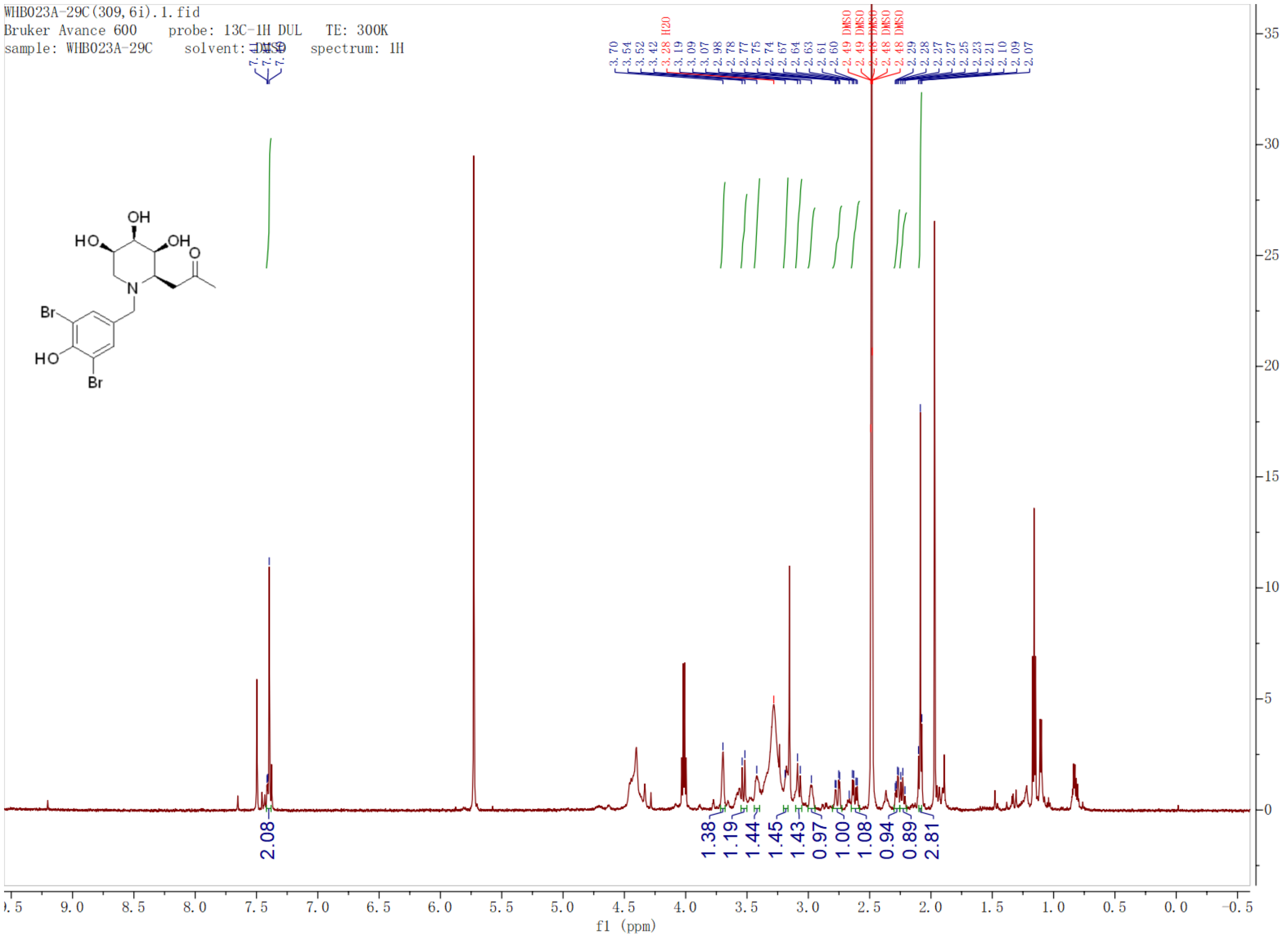

Figure S150. The ^{1}H NMR of compound **6i**.

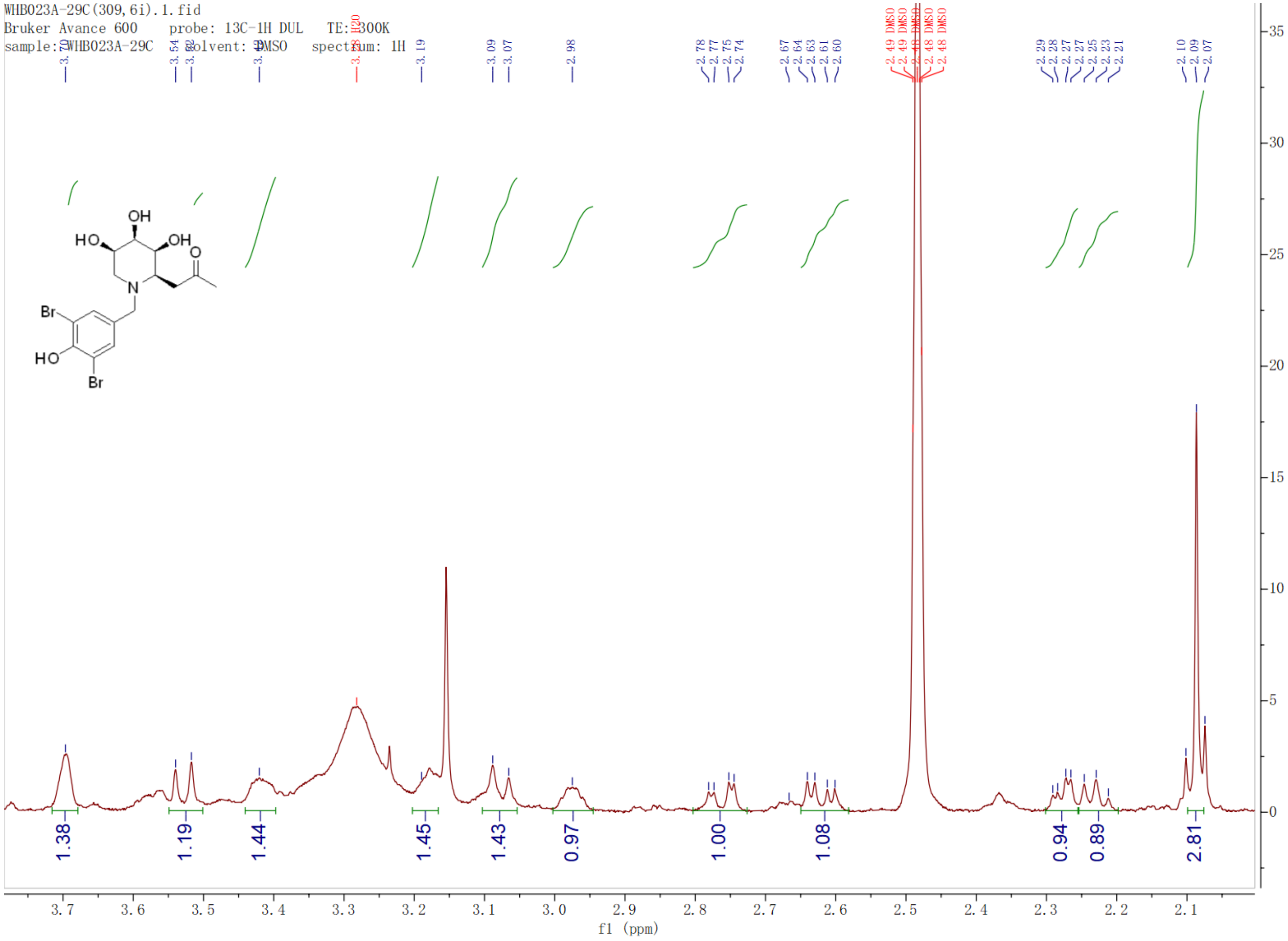

Figure S151. Partial region amplification of the ^{1}H NMR of compound **6i**.

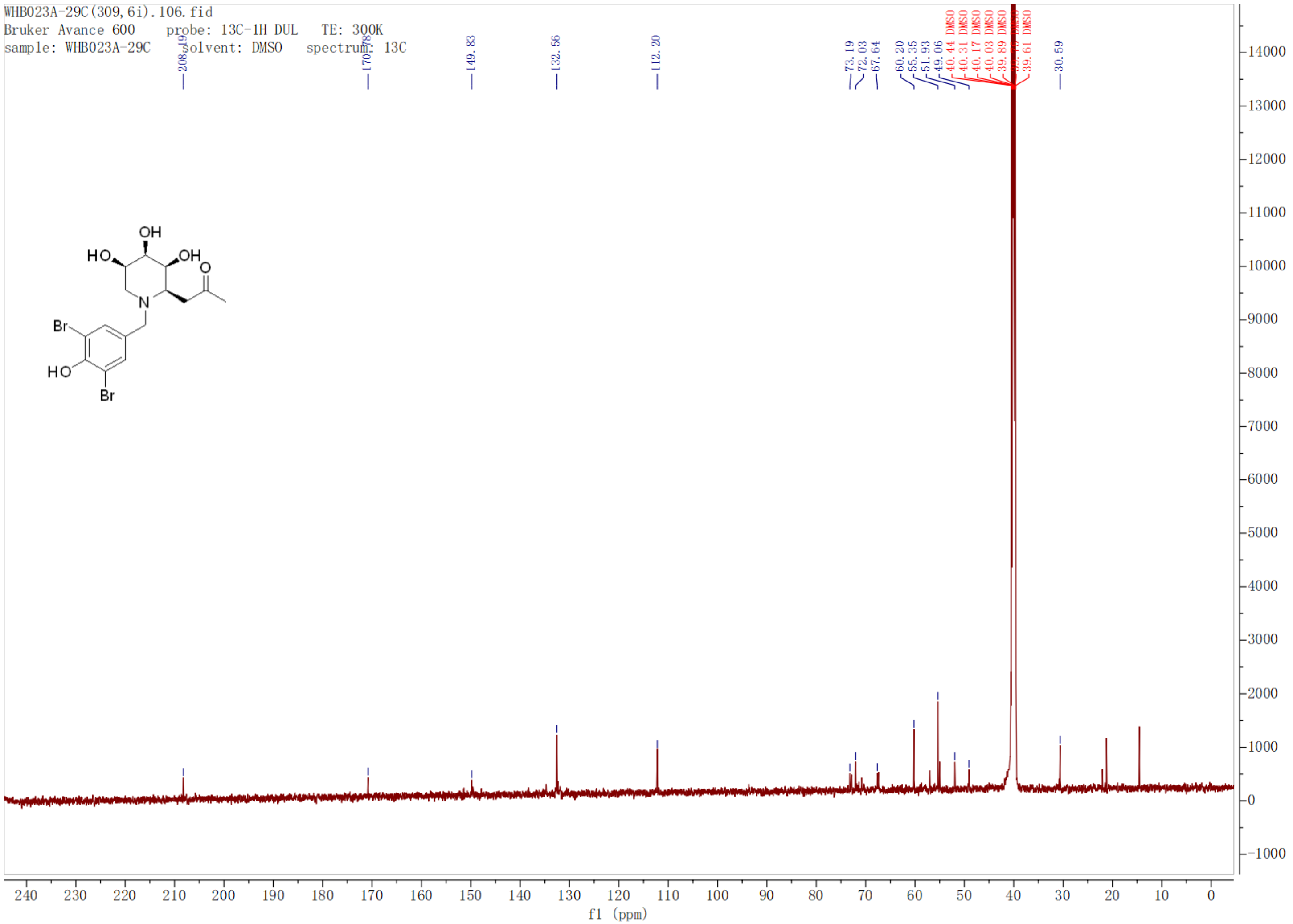

Figure S152. The ^{13}C NMR of compound **6i**.

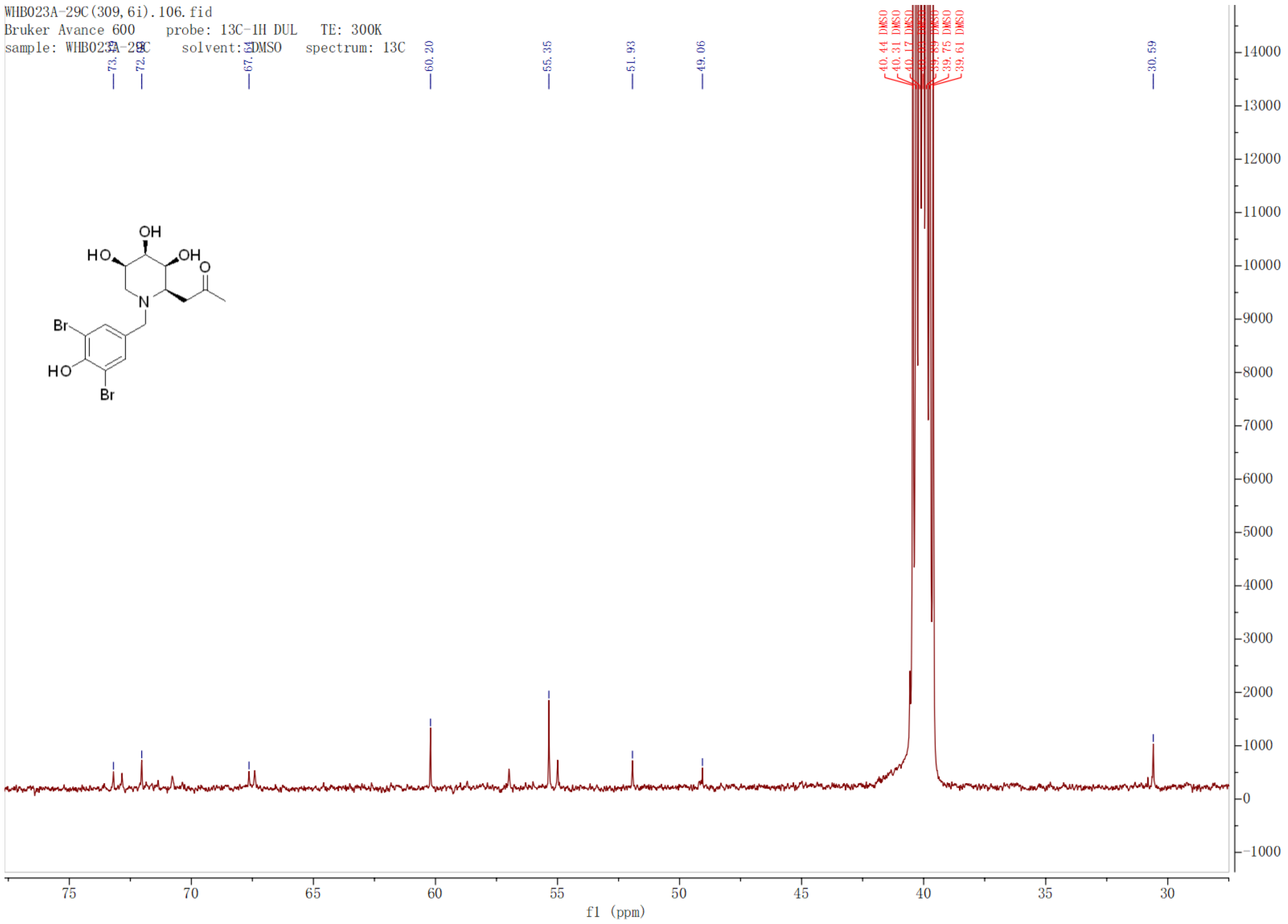

Figure S153. Partial region amplification of the ^{13}C NMR of compound **6i**.
